# One-Pot and Catalyst-Free Transformation of *N*-Protected 1-Amino-1-Ethoxyalkylphosphonates into Bisphosphonic Analogs of Protein and Non-Protein α-Amino Acids

**DOI:** 10.3390/molecules27113571

**Published:** 2022-06-02

**Authors:** Anna Kuźnik, Dominika Kozicka, Wioleta Hawranek, Karolina Socha, Karol Erfurt

**Affiliations:** 1 Department of Organic Chemistry, Bioorganic Chemistry and Biotechnology, Silesian University of Technology, B. Krzywoustego 4, 44-100 Gliwice, Poland; dominika.kozicka@polsl.pl (D.K.); wiolhaw@gmail.com (W.H.); karosoc504@student.polsl.pl (K.S.); 2 Biotechnology Center, Silesian University of Technology, B. Krzywoustego 8, 44-100 Gliwice, Poland; 3 Department of Chemical Organic Technology and Petrochemistry, Silesian University of Technology, B. Krzywoustego 4, 44-100 Gliwice, Poland; karol.erfurt@polsl.pl

**Keywords:** α-aminobisphosphonates, α-ethoxyphosphonates, phosphonium salts, *N*-acylimidates, Michaelis–Becker reaction, Michaelis–Arbuzov reaction

## Abstract

Herein, we describe the development of one-pot transformation of α-ethoxy derivatives of phosphorus analogs of protein and non-protein α-amino acids into biologically important *N*-protected 1-aminobisphosphonates. The proposed strategy, based on the three-component reaction of 1-(*N*-acylamino)-1-ethoxyphosphonates with triphenylphosphonium tetrafluoroborate and triethyl phosphite, facilitates good to excellent yields under mild reaction conditions. The course of the reaction was monitored by ^31^P NMR spectroscopy, allowing the identification of probable intermediate species, thus making it possible to propose a reaction mechanism. In most cases, there is no need to use a catalyst to provide transformation efficiency, which increases its attractiveness both in economic and ecological terms. Furthermore, we demonstrate that the one-pot procedure can be successfully applied for the synthesis of structurally diverse *N*-protected bisphosphonic analogs of α-amino acids. As shown, the indirect formation of the corresponding phosphonium salt as a reactive intermediate during the conversion of 1-(*N*-acylamino)-1-ethoxyphosphonate into a 1-aminobisphosphonate derivative is a crucial component of the developed methodology.

## 1. Introduction

**Figure 1:**
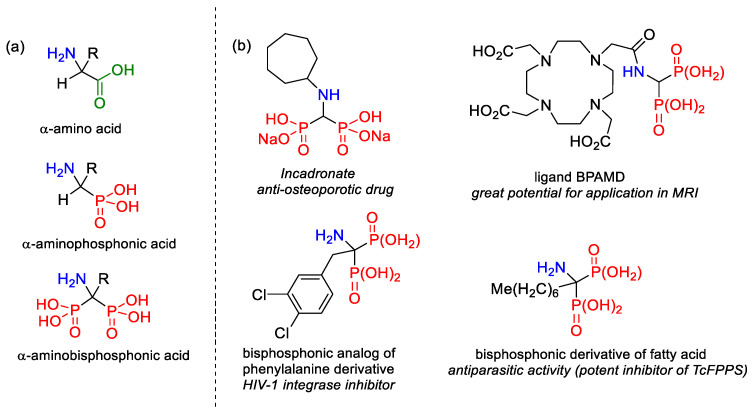
(**a**) Comparison of the structures of α-amino acid, α-aminophosphonic acid and α-aminobisphosphonic acid. (**b**) Representative examples of α-aminobisphosphonates with medical applications.

1-(*N*-Acylamino)alkylene-1,1-bisphosphonates belong to the group of geminal 1-amino-1,1-bisphosphonates (ABPs), and they are characterized by the presence of the P-C(N)-P skeleton. In addition to being synthetic analogs of inorganic pyrophosphate, which is a regulator of calcium metabolism in living organisms, these compounds are considered as structural analogs of α-aminophosphonic acids, which are phosphorus equivalents of α-amino acids [1]. It is known that α-aminophosphonic acids exhibit significant biological activity, including anti-viral, anti-bacterial, anti-inflammatory, and anti-tumor, are potent enzyme inhibitors, and act as herbicides and regulators of plant growth [2,3,4,5]. Their functionalization with an additional phosphonyl group results in the formation of 1 amino-1,1-bisphosphonates (Figure 1a), the activity of which is even stronger, due to the presence of a P-C-P backbone, which has a documented affinity for hydroxyapatite and is resistant to enzymatic hydrolysis [6]. The above factors determine the biological properties of ABPs and the wide range of applications associated therewith.

Along with 1-hydroxy-1,1-bisphosphonates, ABPs are a potent inhibitors of bone resorption used in clinical practice in the treatment of diseases such as osteoporosis, Paget’s disease, or hypercalcemia [7,8]. An example of this is cycloheptylaminomethylene-1,1-bisphosphonate, which is a representative of the latest generation of anti-osteoporotic drugs, commercialized as Incadronate [9]. In addition to anti-resorptive activity, ABPs also show other useful properties, including anti-bacterial [10], anti-viral [11], anti-parasitic [12,13], and herbicidal [14], which provides evidence of their continued development as therapeutics and plant protection agents (Figure 1b). Clinical trials have been conducted on the use of ABPs in oncological therapy, especially immunotherapy [15]. Moreover, thanks to their high affinity for hydroxyapatite and the presence of the amino group in the α position, which enables their further structural modification, they have also become of interest for new drug delivery systems to bone tissue. This is achieved by the formation of conjugates with pharmacological agents, such as radioisotopes, anti-inflammatory drugs, proteins, and agents intended for augmentation of systemic bone mass or antibiotics. The conjugates of ABPs with cytostatics appear to be particularly promising, and they are being tested for their potential use in targeted anti-cancer therapies [16,17]. The high affinity for mineralized tissues is also utilized in the synthesis of new diagnostic agents that enable the imaging of bone tissue by MRI or PET, which is an interesting alternative to the currently used scintigraphy [18]. Great potential for application as a contrast agent in MRI imaging is demonstrated by the complex of the BPAMD ligand with gadolinium, containing a fragment of ABP as a “bone-seeking” moiety (Figure 1b) [19]. The chelating properties of some ABPs are also used to remove radioactive metallic toxins from water or blood [20], as well as for the functionalization of the surface of supermagnetic iron oxide nanoparticles (SPION) used in MRI [18].

Due to the important applications of ABPs, the development of a general and efficient method for their synthesis, or the improvement of previous synthetic methods, still attracts much attention. Currently, there is a range of different methods for the preparation of ABPs. However, they are often utilized for the simplest representatives of this group of compounds, i.e., derivatives of ABPs containing only hydrogen and a variously substituted amino group at the central carbon atom. Among the known methods for the synthesis of ABPs, the following should be mentioned: three-component condensation of amines with dialkyl phosphites in the presence of ethyl orthoformate [21,22,23], Beckmann rearrangement of oximes in the presence of phosphites by using POCl_3_ as a promoter [24], prolonged heating of nitriles with excess phosphoric acid in the presence of phosphorus trichloride and anhydrous benzenesulfonic acid [12], bisphosphonylation of amides using diethyl phosphite in the Tf_2_O-activated reaction in the presence of 2,6-lutidine a base [25], double hydrophosphonylation of nitriles with dialkyl phosphites mediated by titanocene/propylene oxide [26], or ZnCl_2_/Et_3_N system [20], as well as nickel-catalyzed double hydrophosphonylation of aromatic nitriles with trialkyl phosphites assisted by Et_3_B as a reaction promoter [27]. Most of these synthetic strategies, especially those using unreactive nitriles, are carried out under harsh reaction conditions and in the presence of catalytic systems or mediators (often hazardous agents). What is more, as the side chains attached to the α carbon have a significant influence on the biological activities [8,28], it seems that the purposeful synthesis of functionalized α-aminobisphosphonate derivatives is of big importance in the search for compounds with a desirable biomedical profile. This is especially true of such models with the substituents at the α-position identical with those characteristic for natural α-amino acids, both protein and non-protein. The development of a general method for the synthesis of ABPs, providing structural diversity of the product and performed under mild, preferably catalyst-free reaction conditions, is thus highly sought after.

**Scheme 1:**
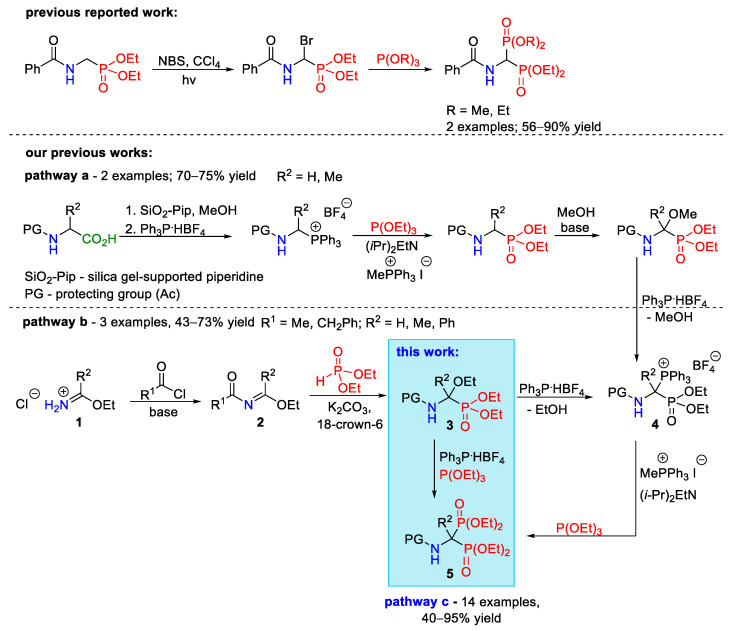
Synthetic routes for the synthesis of tetraethyl 1-(*N*-acylamino)alkylene-1,1-bisphosphonates from α-functionalized derivatives of phosphorus analogs of α-amino acids, such as diethyl 1-(*N*-benzoylamino)bromomethylphosphonate [29] and diethyl 1-(*N*-acetylamino)-1-triphenylphosphoniumalkylphosphonate tetrafluoroborates **4** obtained from α-methoxyphosphonates (pathway **a**) [30] or α-ethoxyphosphonates (pathway **b**) [33].

Recently, we have focused our efforts on developing a new synthetic procedure that allows access to not only α-aminobisphosphonic acids derivatives but also to their asymmetrical analogs. This allows the scope of applicability to be extended and thus the universality of the proposed method for synthesis of bisphosphorus organic compounds. Because each of the phosphorus groups is introduced separately into the final molecule, it has been found that phosphorus analogs of α-amino acids functionalized with a nucleofugal group at the α position are convenient substrates for this type of transformation. To the best of our knowledge, there are only a few reports on the preparation of tetraethyl 1-(*N*-acylamino)alkylene-1,1-bisphosphonates in the Michaelis–Arbuzov-type reaction of triethyl phosphite with diethyl 1-(*N*-acylamino)alkylphosphonates containing a nucleofugal group at the α-position. Despite the presence of the *N*-acylamino group and the dialkoxyphosphoryl group with an electron-withdrawing inductive effect, this type of functionalization of 1-(*N*-acylamino)alkylphosphonates is necessary to display the electrophilicity of their α-carbon required for further reaction. Thus, only one example of this type of transformation has been reported in the literature involving diethyl 1-(*N*-benzoylamino)bromomethylphosphonate synthesized by photochemical bromination of the starting 1-(*N*-benzoylamino)methylphosphonate with NBS [29]. The subsequent Michaelis–Arbuzov-type reaction of diethyl 1-(*N*-benzoylamino)bromomethylphosphonate with trimethyl and triethyl phosphites provided the expected 1-(*N*-benzoylamino)methylene-1,1-bisphosphonates in a yield of 56–90% (Scheme 1).

Other substrates that have been used in this reaction in the presence of Hünig’s base and methyltriphenylphosphonium iodide as catalysts are diethyl 1-(*N*-acetylamino)-1-triphenylphosphoniumalkylphosphonate tetrafluoroborates **4**, which can be considered as α-functionalized triphenylphosphonium derivatives of 1-(*N*-acylamino)alkylphosphonates [30]. The starting phosphonium salts **4** were synthesized here from diethyl 1-aminoalkylphosphonates readily available from *N*-acyl-α-amino acids [31,32] by initially subjecting them to electrochemical oxidation to introduce the nucleofugal methoxy group into the α-position, which was followed by nucleophilic substitution of the obtained diethyl 1-(*N*-acetylamino)-1-methoxyalkylphosphonates with triphenylphosphonium tetrafluoroborate (Scheme 1, pathway a). The biggest limitation of this transformation is the electrochemical oxidation step, which was efficiently performed for only two of the simplest models of phosphorus analogs of α-amino acids, namely for the derivative of glycine and alanine, having at the α-position a hydrogen atom or a methyl group, respectively. Attempts to perform this process for the phosphorus analogs of valine and phenylalanine have failed, possibly due to a steric hindrance of the bulky substituent at the α-position.

To overcome this problem, we looked for a different method of obtaining α-alkoxy derivatives of diethyl α-aminophosphonates with the result of the development of another procedure for the preparation of diethyl 1-(*N*-acylamino)-1-ethoxyalkylphosphonates **3** obtained in a Michaelis–Becker-type reaction of ethyl *N*-acylimidates **2** with diethyl phosphite (Scheme 1, pathway b) [33]. This opened up the wider applicability of this method, since the electrochemical oxidation step required in the previous transformation is omitted in this case, while the starting *N*-acylimidates **2** are readily available from the well-known class of chemical compounds, i.e., ethyl imidate hydrochlorides **1** [34,35]. We then converted 1-(*N*-acylamino)-1-ethoxyalkylphosphonates **3** into diethyl 1-(*N*-acylamino)-1-triphenylphosphoniumalkylphosphonate tetrafluoroborates **4**, whose utility in the synthesis of tetraethyl 1-(*N*-acylamino)alkylene-1,1-bisphosphonates **5** has so far been described for only three models of α-ethoxy derivatives of diethyl alkylphosphonates **3**, such as phosphorus analogs of glycine, alanine and phenylglycine, which have the amino group protected with selected acyl groups (acetyl or phenylacetyl). The target tetraethyl 1-(*N*-acylamino)alkylene-1,1-bisphosphonates **5** were synthesized here in the Michaelis–Arbuzov-type α-amidoalkylation reaction of triethyl phosphite with the previously obtained phosphonium salts **4** in a double catalytic system in the presence of methyltriphenylphosphonium iodide and Hünig’s base.

In a continuation of our efforts to improve the recently developed procedure for the preparation of organobisphosphorus compounds, and hoping that it has the potential to become a general method for the synthesis of ABPs, we report an efficient, catalyst-free one-pot transformation of α-ethoxyaminophosphonate derivatives into tetraethyl 1-(*N*-acylamino)alkylene-1,1-bisphosphonates possessing at the α position a side chain identical with those characteristic for natural α-amino acids, both protein and non-protein (Scheme 1, pathway c).

## 2. Results and Discussion

### 2.1. Optimization of Conditions for the Synthesis of α-Ethoxy Derivatives of Phosphorus Analogs of α-Amino Acids

**Scheme 2:**
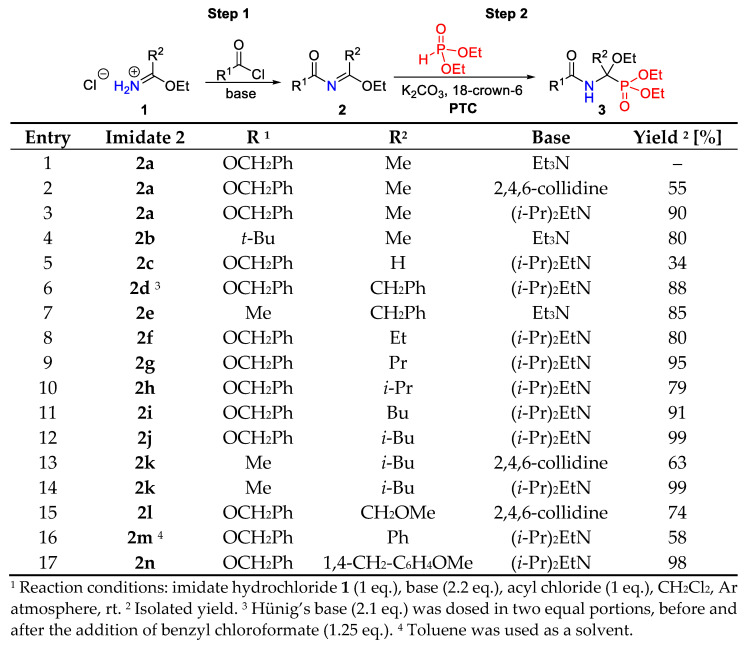
Two-step synthesis of 1-(*N*-acylamino)-1-ethoxyalkylphosphonates from ethyl imidate hydrochlorides and yields of acylation of ethyl imidate hydrochlorides **1** with acid chlorides in a presence of selected bases.

The starting diethyl 1-(*N*-acylamino)-1-ethoxyalkylphosphonates (Table 1, **3a**–**n**) were synthesized according to a previously described two-step protocol with some modifications (Scheme 2) [33]. The general procedure consists of acylation of the imidate hydrochloride **1** with an acyl chloride (Step 1) and the Michaelis–Becker-like addition of diethyl phosphite to ethyl *N*–acylimidate **2** (Step 2).

These modifications in the acylation step of ethyl imidate hydrochlorides **1**, most often with benzyl chloroformate, require a different base for this reaction (hitherto, Et_3_N has been used in the acylation reaction with acetyl chloride). This change was introduced following optimization studies to select an appropriate base to improve the efficiency of acylation, and sometimes even allow it to be carried out, taking into account the key role of the base environment in this reaction. Since the use of Et_3_N in the acylation reaction of ethyl acetimidate hydrochloride **1a** with benzyl chloroformate was unsuccessful (Scheme 2, entry 1), 2,4,6-collidine and (*i*-Pr)_2_EtN (Hünig’s base) were used as bases for this reaction. When the weaker aromatic base such as 2,4,6-collidine was used, the expected reaction took place with the product being isolated in a moderate yield of 55% (entry 2). Therefore, we tried to perform the same reaction using Hünig’s base with comparable strength to Et_3_N but non-nucleophilic in nature to prevent side reactions. This facilitated a higher yield of 90% for the acylation product (entry 3). The lack of nucleophilic character of Hünig’s base can be explained by the presence of two sterically extended isopropyl groups. Based on this successful result, the acylation reactions for all the remaining ethyl imidate hydrochlorides with benzyl chloroformate were carried out with the use of this base (Scheme 2), except for the 2-methoxyacetimidate hydrochloride **1l** which, in the case of 2,4,6-collidine, proved to be much more effective (entry 15).

As for the second step of the synthesis of 1-(*N*-acylamino)-1-ethoxyalkylphosphonates **3**, i.e., the nucleophilic addition of diethyl phosphite to ethyl *N*-acylimidates **2** in the Michaelis–Becker-like reaction, the modification was required to improve its efficiency. For most of the synthesized models, especially those with an amine group protected with a Cbz group, it consisted of reducing the reaction temperature under PTC conditions up to −40 °C and using a two-fold molar excess of the nucleophile in some cases. Therefore, it was possible to obtain the expected products, which were isolated by extraction initially with hexane and then with dichloromethane and subsequent purification of the extracts by column chromatography in satisfactory yields (Table 1).

### 2.2. Development of an Optimized One-Pot Procedure for the Synthesis of Bisphosphonate Analogs of α-Amino Acids

**Figure 2:**
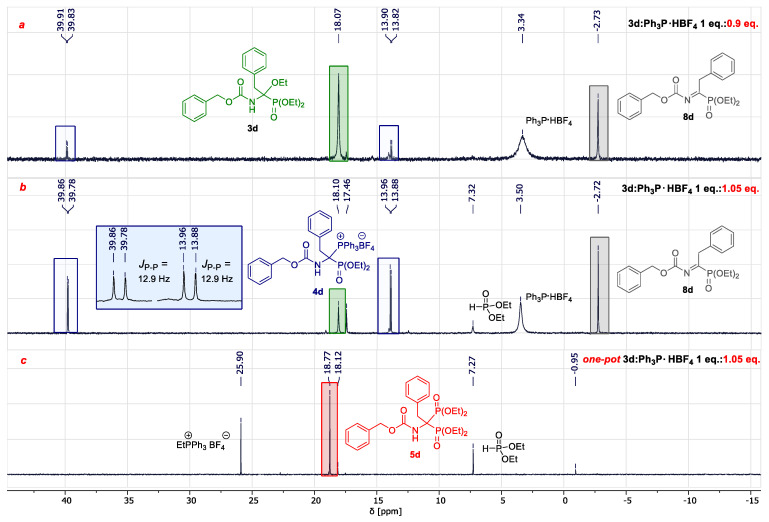
(**a**) ^31^P NMR spectrum of the reaction mixture obtained in the synthesis of phosphonium salt **4d** carried out with a slight molar deficiency of Ph_3_P·HBF_4_ at −40 °C. (**b**) ^31^P NMR spectrum of the reaction mixture obtained in the synthesis of a phosphonium salt **4d** conducted with a slight molar excess of Ph_3_P·HBF_4_ at −40 °C. (**c**) ^31^P NMR spectrum of the reaction mixture obtained in the one-pot synthesis of bisphosphonic derivative **5d** performed with the use of Ph_3_P·HBF_4_ in a molar excess at room temperature.

The electrophilicity of the α-carbon atom of the synthesized α-ethoxyphosphonate derivative **3** is too low to allow its direct transformation into the target bisphosphonate **5** using the Michaelis–Arbuzov-type reaction with triethyl phosphite [30]. Therefore, to increase the electrophilicity of this position, it was necessary to convert α-ethoxyphosphonate **3** to the corresponding phosphonium salt **4**, which is much more reactive and thus susceptible to subsequent reaction with the nucleophilic triethyl phosphite. To better understand the reaction mechanism for the preparation of diethyl 1-(*N*-acylamino)-1-triphenylphosphoniumalkylphosphonate tetrafluoroborates **4** from 1-(*N*-acylamino)-1-ethoxyalkylphosphonates **3**, we attempted the synthesis of another model phosphonium salt from the α-ethoxy derivative of the phosphorus analog of phenylalanine **3d** with the intention of isolating and purifying it. For this purpose, triphenylphosphonium tetrafluoroborate was added to α-ethoxyphosphonate in a slight molar deficiency (0.9 eq. to 1 eq. **3d**). To our surprise, the ^31^P NMR analysis of the reaction mixture taken after 10 and 30 min at room temperature did not confirm the presence of the expected phosphonium salt **4d**. Therefore, this synthesis was repeated according to the same procedure, but modifying the reaction conditions by lowering the temperature to −15 °C. Again, no characteristic signals belonging to the corresponding phosphonium salt were found. Due to the predicted instability of the synthesized diethyl 1-(*N*-benzyloxycarbonylamino)-1-triphenylphosphonium-2-phenylethylphosphonate tetrafluoroborate, it was decided to conduct another experiment at −40 °C, which was analogous to the described procedure above but with a slight molar excess of triphenylphosphonium tetrafluoroborate (5%). After 40 min of reaction, NMR analysis was performed, which provided very promising results with two clearly visible doublets at 13.9 and 39.8 ppm of the same coupling constant (J = 12.9 Hz), confirming the presence of the desired phosphonium salt **4d** (Figure 2b).

In subsequent experiments, the conditions for the synthesis of **4d** were modified in order to assess their impact on the reaction course (Figure 2). Initially, it was assumed that in the synthesis of phosphonium salt, the temperature was a determining factor having an influence on the reaction course. This was based on the results of the first experiments, in which two expected doublets at 13.9 and 39.8 ppm were observed only for the reaction carried out at −40 °C. However, subsequent experiments showed that it is not the temperature but the molar ratio of triphenylphosphonium tetrafluoroborate to substrate **3** that is of key importance for obtaining phosphonium salt **4**. During a detailed analysis of reaction mixture ^31^P NMR spectra, it was observed that in those syntheses in which a slight molar deficiency of Ph_3_P·HBF_4_ was used, the following signals were present in the spectrum, at approximately 18.1 ppm of high intensity together with a weaker signal at −2.8 ppm and broad intense signal at 3.3 ppm corresponding to Ph_3_P·HBF_4_ in the equilibrium (Figure 2a). In contrast, there was a lack of the signal at 18.1 ppm when excess Ph_3_P·HBF_4_ was used, and the equilibrium of the reaction shifted toward an intermediate with the signal at about −2.8 ppm, and phosphonium salt **4d**, appearing in the form of two doublets (Figure 2b).

**Scheme 3:**
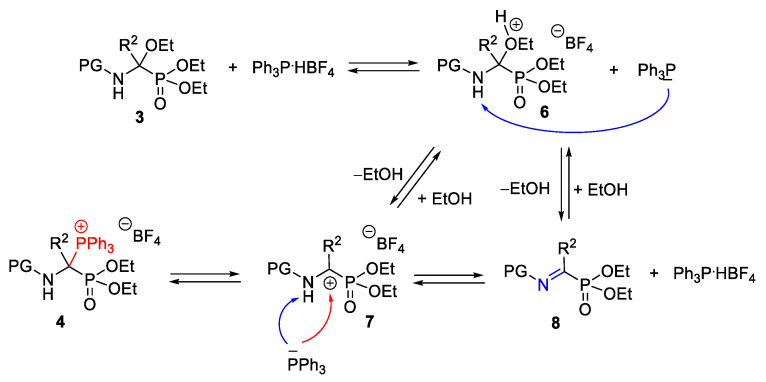
A plausible mechanism for the formation of diethyl 1-(*N*-acylamino)-1-triphenylphosphoniumalkylphosphonate tetrafluoroborate **4** proposed based on the analysis of ^31^P NMR spectra (161.9 MHz/CDCl_3_; ppm) of reaction mixtures obtained in reactions of substrate **3** and Ph_3_P·HBF_4_ used in various molar ratios.

An explanation is presented in Scheme 3, which illustrates the proposed mechanism for the formation of phosphonium salt **4** in an equilibrium reaction via intermediates **6**–**8**. In the first step, α-ethoxyphosphonate **3** reacts with triphenylphosphonium tetrafluoroborate to give salt **6** with a protonated ethoxy group in the α position and liberated triphenylphosphine. When there is a shortage of triphenylphosphonium tetrafluoroborate in relation to reaction substrate, it becomes partially blocked at this stage. Ethanol is cleaved from the resulting salt **6**, the iminophosphonate **8** (which is in equilibrium with iminium type-cation **7**) is formed and Ph_3_P·HBF_4_ is regenerated. This results in an intense ^31^P NMR spectrum signal at approximately 18.1 ppm, belonging to the starting compound **3d**, along with the broad signal of Ph_3_P·HBF_4_ (3.3 ppm) and intermediate iminophosphonate **8** at about −2.8 ppm (which is consistent with the literature data for this type of imines [36]). Conversely, in the case of an excess of triphenylphosphonium tetrafluoroborate (higher acidity of the reaction mixture), iminium-type cation **7** is formed more readily. Finally, the active electrophilic center of iminium-type cation **7** is attacked by triphenylphosphine and the desired phosphonium salt **4** is formed (Scheme 3).

The confirmation of the proposed phosphonium salt **4** formation mechanism is illustrated in the ^31^P NMR spectra of the reaction mixture (Figure 2a,b), with signals belonging to α-ethoxyphosphonate **3d** at approximately 18.1 ppm, Ph_3_P·HBF_4_ at 3.3 ppm, intermediate iminophosphonate **8d** at about −2.8 ppm and expected phosphonium salt **4d** with the corresponding two doublets at 13.9 and 39.8 ppm. The presence of all these signals on the spectra of the reaction mixtures is evidence that this is an equilibrium reaction, in the course of which the acidity of the environment is of critical importance. On the other hand, reducing the temperature allows an unstable reaction intermediate to be observed in the ^31^P NMR spectrum as two intense doublets belonging to the expected phosphonium salt **4d**.

On the basis of the postulated mechanism, it was concluded that diethyl 1-(*N*-benzyloxycarbonylamino)-1-triphenylphosphonium-2-phenylethylphosphonate tetrafluoroborate **4d**, which is a reactive intermediate in this synthetic route, has to be used in situ in the subsequent transformation into α-aminobisphosphonate derivative **5d**. Hence, we attempted to transform diethyl 1-(*N*-acylamino)-1-ethoxyalkylphosphonates **3** into 1-(*N*-acylamino)alkylene-1,1-bisphosphonates **5** via the corresponding phosphonium salts using a one-pot method (Figure 2c). Our screening tests, described above with the use of the model **3d** as substrate, showed that during the synthesis of phosphonium salt **4d**, iminophosphonate **8d** is spontaneously formed in the equilibrium mixture. From this, we concluded that the addition of Hünig’s base as a catalyst in the synthesis of the bisphosphonates is redundant. Indeed, carrying out the one-pot synthesis of tetraethyl 1-(*N*-benzyloxycarbonylamino)-2-phenylethylene-1,1-bisphosphonate **5d** by dissolving all the reactants, namely substrate **3d**, Ph_3_P·HBF_4_ and triethylphosphite, used in a molar ratio of 1:1.05:1.5, in dichloromethane at 0–5 °C in the presence of methyltriphenylphosphonium iodide as a catalyst (0.25 eq.) and left at this temperature for 45 min, then at room temperature overnight, resulted in the expected product **5d** with an estimated yield of 52% (Table 2, entry 1). This success inspired further optimization of the transformation conditions. This included the question of whether the presence of methyltriphenylphosphonium iodide is necessary here, since the function of the dealkylating agent for triethoxyphosphonium salt, obtained as an intermediate in the Michaelis–Arbuzov reaction, could potentially be performed by triphenylphosphine that is present in the reaction mixture [37,38]. The next experiment was therefore carried out in an analogous manner but without the addition of any catalysts, providing an estimate yield of bisphosphonate **5d** of 73% (Table 2, entry 2). This result provided unequivocal evidence that the catalyst methyltriphenylphosphonium iodide is not required for this reaction to proceed efficiently, and thus, the reaction takes place in an autocatalytic system. It was also considered whether the dosing of the reactants at a reduced temperature is required and what molar ratio of Ph_3_P·HBF_4_ to α-ethoxyphosphonate **3d** will be the most favorable. Table 2 shows the molar ratios of these reagents used together with the bisphosphonate yields afforded in the given experiments when performed at room temperature. It was found that it is sufficient to use a slight molar excess of Ph_3_P·HBF_4_ at the level of 5–8% in the synthesis of α-aminobisphosphonate, and that room temperature is optimal for this transformation (entries 3 and 4). The progress of the reaction was monitored by NMR spectroscopy, and finally, it was concluded that 6 h was sufficient time for the substrate to completely react (entry 5). The product was isolated from the reaction mixture by extraction with toluene and subsequent purification of the extract by column chromatography to afford the target bisphosphonate in a yield of 86%.

### 2.3. Scope of the Reaction

**Scheme 4:**
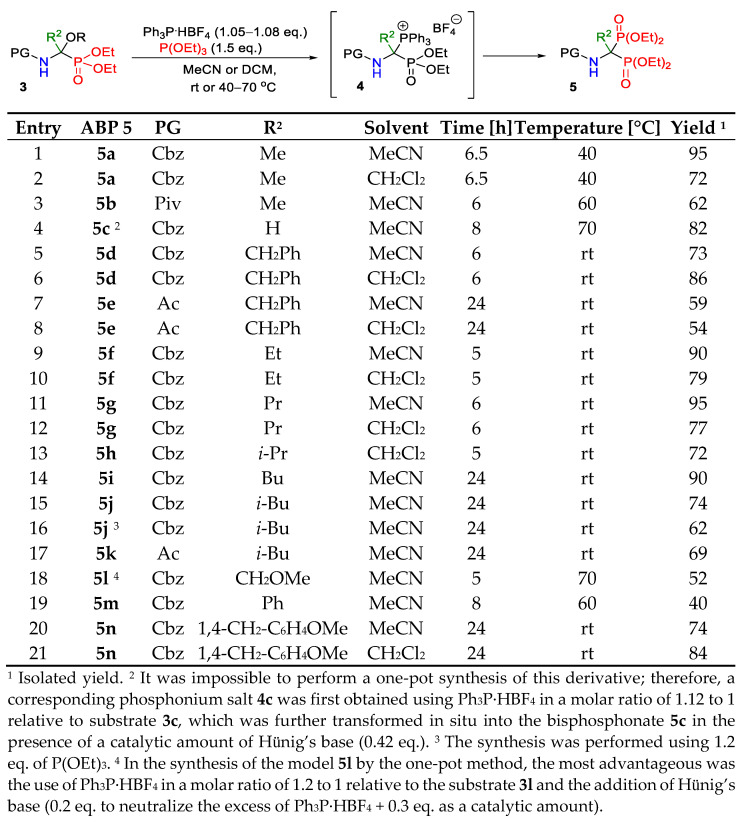
Conditions and yields for the synthesis of tetraethyl 1-(*N*-acylamino)alkylene-1,1-bisphosphonates in the Michaelis–Arbuzov-like reaction.

We then studied the scope of the one-pot reaction for the synthesis of various models of bisphosphonic analogs of protein and non-protein α-amino acids **5** according to the previous optimal conditions (Scheme 4).

These optimized conditions were tested on other α-ethoxyphosphonates **3** containing either aromatic or aliphatic substituents at the α position with straight or branched chains, e.g., for valine and nor-valine or leucine and nor-leucine analogs. For most models, these conditions were well matched, and it was enough to combine all reagents without any catalyst in a chosen solvent and leave it for 5 to 24 h at room temperature to successfully perform the reaction. Subsequent isolation of the obtained product initially by extraction with toluene and then column chromatography provided target compounds **5** with good to excellent yields (72–95%; Cbz-protected amino group). However, in some cases, the use of elevated temperature (40–70 °C) was required to conduct the reaction under catalyst-free conditions (Scheme 4, entries 1–3, 19).

For the majority of the synthesized bisphosphonic derivatives of α-amino acids, the amino group was protected with an easily removable benzyloxycarbonyl group. However, the tested procedure also worked well for models with the amino group protected with other acyl groups, such as acetyl or pivaloyl, leading to expected product yields in a moderate range of 54–62% (entries 3, 7, 8, 17).

We also tested the influence of the solvent on the yield of ABPs during synthesis, finding a general relationship that acetonitrile is a better solvent for this transformation. However, for some bisphosphonates, a higher reaction yield was noted with dichloromethane (cf. entries 5, 6 and 20, 21).

For one model, namely the *N*-Cbz-protected α-ethoxy derivative of phosphorus analog of leucine, the impact of the excess of triethyl phosphite on the efficiency of the synthesis of the corresponding bisphosphonate **5j** was also evaluated. We noted that the reaction yield was higher with the use of 1.5 eq. of P(OEt)_3_ (74%) than for 1.2 eq. of the nucleophile used (62%) (entries 15, 16).

**Figure 3:**
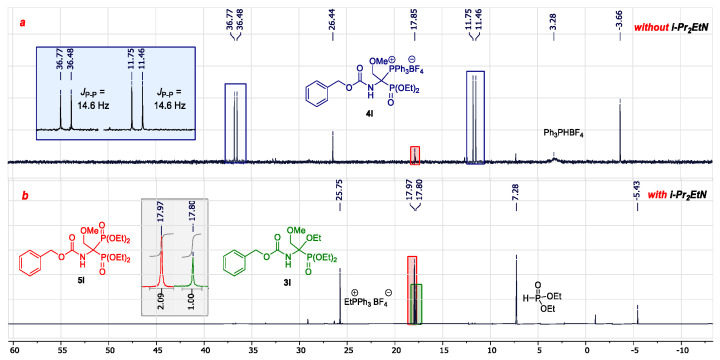
(**a**) ^31^P NMR spectrum of the reaction mixture obtained from the synthesis of the bisphosphonic derivative of serine **5l** without the use of Hünig’s base catalyst at 70 °C. (**b**) ^31^P NMR spectrum of the reaction mixture obtained in the synthesis of bisphosphonic derivative of serine **5l** performed in the presence of catalytic Hünig’s base at 70 °C.

It should be noted that in the case of the bisphosphonic derivative of serine **5l**, the final one-pot reaction was successfully carried out only when the Hünig’s base was also used as a catalyst and when the reaction temperature was raised to 70 °C. The selection of such parameters undoubtedly facilitated the transformation of the indirectly formed phosphonium salt **4l** into the target product **5l** (Figure 3b), which was obtained with a yield of 52% (Scheme 4, entry 18). The course of the reaction at a lower temperature or without the use of a catalyst ended at the stage of phosphonium salt generation, which can be seen in the ^31^P NMR spectrum of the reaction mixture in the form of two doublets with the same coupling constant, which was accompanied by the very small signal of the desired product **5l** (δ = 17.9 ppm) (Figure 3a).

**Scheme 5:**
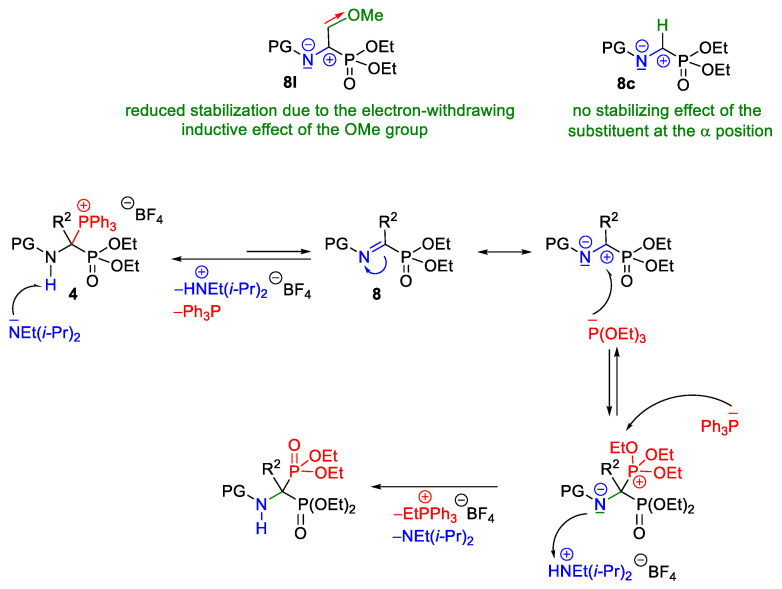
The role of Hünig’s base as a catalyst for the Michaelis–Arbuzow-type reaction in the synthesis of bisphosphonic derivatives of serine **5l** (R^2^ = CH_2_OMe) and glycine **5c** (R^2^ = H) models.

The functional role of Hünig’s base as a catalyst in the synthesis of a bisphosphonic serine derivative relies on assisting the generation of the corresponding *N*-acyliminophosphonate **8l** from the resulting phosphonium salt **4l** during the final Michaelis–Arbuzow-type reaction. This is the rate-determining step of the transformation. For the serine model, due to the presence of an electron-withdrawing methoxymethyl group at the α position, the stabilization of the iminophosphonate **8l** is reduced (Scheme 5), so that the reaction equilibrium is strongly shifted toward the phosphonium salt **4l**. Hence, the addition of Hünig’s base is necessary to allow the Michaelis–Arbuzov-type reaction to be performed, as it is shown in the postulated mechanism for this transformation (Scheme 5).

Moreover, in the case of the bisphosphonic derivative of glycine **5c**, not only was the Hünig’s base catalyst and an increased temperature (70 °C) required, but the intended one-pot transformation was not achieved. This is likely due to the reversible nature of the transformation being carried out, during which the recovery of the starting α-ethoxyphosphonate **3c** in the equilibrium reaction was privileged (Scheme 3), due to the presence of ethanol in the reaction mixture. To overcome this problem, the synthesis of the bisphosphonic derivative **5c** was carried out according to a two-step procedure. First, the phosphonium salt **4c** was obtained by heating the residue after evaporation of the solvent from a homogeneous mixture, which was prepared by dissolving α-ethoxyphosphonate **3c** and triphenylphosphonium tetrafluoroborate at 85 °C under reduced pressure for 5 h. Next, the crude phosphonium salt **4c** was subjected to the Michaelis–Arbuzow-type reaction after dissolving in acetonitrile by treatment with triethylphosphite in the presence of Hünig’s base, resulting in a very good yield of the target product **5c** (82%) (Scheme 4, entry 4).

Regarding the prospects for the further use of synthesized bisphosphonate models **5**, we will carry out structural modifications to increase their application potential in medical chemistry. One future research direction involves the synthesis of conjugates by combining compounds with proven biological activity (e.g., anti-cancer) with α-aminobisphosphonates that can be used as drug carriers. Their functional role in these complexes includes not only the targeted delivery of pharmaceuticals to the bone tissue but also synergistic action with anti-cancer drugs. Minor modifications to the structure of α-aminobisphosphonates will also be of interest. The acylation of α-aminobisphosphonates with the use of appropriate chloroacyl chlorides will allow the production of building blocks that are useful in the synthesis of a ligands, which can be used as potential contrast agents for imaging of bone mineral by MRI after complexing with paramagnetic ions.

## 3. Materials and Methods

### 3.1. General Information

Melting points were determined in capillaries in a Stuart Scientific SMP3 melting point apparatus and were uncorrected. ^1^H-NMR spectra were acquired on a Varian 400 spectrometer at an operating frequency of 400 MHz using tetramethylsilane (TMS) as the resonance shift standard. ^13^C-NMR spectra were recorded on a Varian 400 at 100 MHz, using solvent resonance as the internal standard. ^31^P-NMR spectra were recorded on a Varian 400 at 161.9 MHz without the resonance shift standard, with respect to H_3_PO_4_ as 0 ppm. All chemical shifts (δ) are reported in ppm and coupling constants (*J*) in Hz. IR-spectra were measured on a Nicolet 6700 FT-IR spectrophotometer, Thermo Scientific (attenuated total reflectance method; ATR). The high-resolution mass spectra (HRMS) were obtained by electrospray ionization (ESI) using a Waters Corporation Xevo G2 QTOF instrument. The reactions of Michaelis–Becker-like addition of diethyl phosphite to ethyl *N*-acylimidates were performed at reduced temperatures using Julabo ultra-low refrigerated-circulator F81-ME. For TLC analysis, Merck TLC silica gel 60 F_254_ plates were used. The plates were visualized by UV light (254 nm) and/or dipped in a solution of cerium sulfate and tetrahydrate of ammonium heptamolybdate in H_2_SO_4aq_ and heated. Kieselgel 60 (Merck, 0.040–0.063 mm) was used for column chromatography.

Materials. All solvents and common reagents were obtained from commercial suppliers. Diethyl phosphite and triethyl phosphite were purchased from Acros Organics.

^1^H, ^13^C, ^31^P NMR spectrum of all new compounds **3**, **4c** and **5** are available in Supplementary Materials.

### 3.2. Substrate Synthesis

Commercially available ethyl acetimidate and ethyl benzimidate hydrochlorides (**1a** and **1m**) were used. Ethyl formimidate hydrochloride **1c** was synthesized according to the procedure described by Schmitz and Ohme [39]. The rest of ethyl imidate hydrochlorides **1** were obtained according to the protocol given by Yadav and Babu [40].

General procedure for the synthesis of diethyl 1-(*N*-acylamino)-1-ethoxyalkylphosphonates **3**.

Step 1: *N*-Acylation reactions of ethyl imidate hydrochlorides **1** were carried out according to the modified procedure described by Kuźnik et al. [33]. The appropriate base (17.6 mmol, 2.2 eq.) (Scheme 2) was added to a solution of ethyl imidate hydrochloride **1** (8.0 mmol, 1.0 eq.) in dry CH_2_Cl_2_ (25 mL) and cooled in an ice bath. Then, acid chloride (8.0 mmol, 1.0 eq.) was added to the reaction mixture dropwise. The ice bath was removed, and the mixture was stirred under argon atmosphere, at room temperature, for 24 h, and the solvent was evaporated under reduced pressure. To separate the product from base hydrochloride, hexane (15 mL) was added to the residue. The precipitate was filtered over celite, and the filtrate was concentrated to give ethyl *N*-acylimidate **2**.

Due to the instability of the ethyl *N*-(benzyloxycarbonyl)formimidate **2c**, *N*-acylation of ethyl formimidate hydrochloride **1c** was carried out in an ice bath, and the reaction time was reduced to 2 h. The obtained compound was immediately used in the next step.

Synthesis of ethyl *N*-(benzyloxycarbonyl)phenylacetimidate **2d** was performed using Hünig’s base (2.1 eq.), added in two equal portions, before and after the addition of benzyl chloroformate (1.25 eq.). *N*-Acylation of ethyl benzimidate hydrochloride **1m** was carried out using toluene as a solvent.

*Ethyl N-(benzyloxycarbonyl)acetimidate* (**2a**). Pale yellow oil; 90% yield (1.592 g). ^1^H-NMR (400 MHz, CDCl_3_): δ 7.40–7.32 (m, 5H), 5.19 (s, 2H), 4.16 (q, *J =* 7.2 Hz, 2H), 2.05 (s, 3H) 1.28 (t, *J* = 7.2 Hz, 3H). ^13^C-NMR (100 MHz, CDCl_3_): δ 167.9, 161.5, 135.9, 128.5, 128.4, 128.3, 68.1, 63.2, 18.4, 13.8. HRMS (ESI) *m/z*: calcd for C_12_H_15_NO_3_Na [M + Na]^+^ 244.0950, found 244.0951.
*Ethyl N-(pivaloyl)acetimidate* (**2b**) [41]. Pale yellow oil; 80% yield (1.095 g). ^1^H-NMR (400 MHz, CDCl_3_): δ 4.12 (q, *J* = 7.0 Hz, 2H), 1.98 (s, 3H), 1.29 (t, *J* = 7.2 Hz, 3H), 1.18 (s, 9H). ^13^C-NMR (100 MHz, CDCl_3_): δ 191.7, 161.3, 62.5, 41.4, 27.1, 18.0, 14.0. HMRS (ESI) *m/z*: calcd for C_9_H_18_NO_2_ [M + H]^+^ 172.1338, found 172.1343.
*EthylN-(benzyloxycarbonyl)formimidate* (**2c**). Pale yellow oil; 34% yield (565 mg). ^1^H-NMR (400 MHz, CDCl_3_): δ 8.45 (s, 1H), 7.54–7.28 (m, 5H), 5.12 (s, 2H), 4.34 (q, *J* = 7.0 Hz, 2H), 1.34 (t, *J* = 7.0 Hz, 3H). ^13^C-NMR (100 MHz, CDCl_3_): δ 167.1, 161.9, 135.6, 128.53, 128.51, 128.3, 68.4, 64.5, 13.8. HRMS (ESI) *m/z*: calcd for C_11_H_14_NO_3_ [M + H]^+^ 208.0974, found 208.0977.
*EthylN-(benzyloxycarbonyl)-2-phenylacetimidate* (**2d**). Pale yellow oil; 88% yield (2.095 g). ^1^H-NMR (400 MHz, CDCl_3_): δ 7.35–7.19 (m, 10H), 5.13 (s, 2H), 4.16 (q, *J =* 7.2 Hz, 2H), 3.68 (s, 2H), 1.25 (t, *J =* 7.2 Hz, 3H). ^13^C-NMR (100 MHz, CDCl_3_): δ 167.9, 161.1, 135.7, 134.2, 129.2, 128.6, 128.5, 128.3, 127.0, 68.2, 63.5, 38.8, 13.8. HRMS (ESI) *m/z*: calcd for C_18_H_20_NO_3_ [M + H]^+^ 298.1443, found 298.1443.
*Ethyl N-(acetyl)-2-phenylacetimidate* (**2e**) [42]. Pale yellow oil; 85% yield (1.389 g). ^1^H-NMR (400 MHz, CDCl_3_): δ 7.26–7.53 (m, 5H), 4.10 (q, *J =* 7.0 Hz, 2H), 3.66 (s, 2H), 1.96 (s, 3H), 1.17 (t, *J* = 7.2 Hz, 3H). ^13^C-NMR (100 MHz, CDCl_3_): δ183.4, 161.1, 134.5, 129.3, 128.6, 127.1, 62.9, 38.3, 26.3, 13.8. HRMS (ESI) *m/z*: calcd for C_12_H_16_NO_2_ [M + H]^+^ 206.1181, found 206.1184.
*Ethyl N-(benzyloxycarbonyl)propanimidate* (**2f**). Pale yellow oil; 80% yield (1.507 g). ^1^H-NMR (400 MHz, CDCl_3_): δ 7.42–7.30 (m, 5H), 5.19 (s, 2H), 4.15 (q, *J* = 7.2 Hz, 2H), 2.36 (q, *J* = 7.6 Hz, 2H), 1.28 (t, *J* = 7.2 Hz, 3H), 1.12 (t, *J* = 7.6 Hz, 3H). ^13^C-NMR (100 MHz, CDCl_3_): δ 170.7, 161.4, 135.9, 128.5, 128.4, 128.3, 68.1, 63.0, 26.1, 13.8, 10.5. HRMS (ESI) *m/z*: calcd for C_13_H_17_NO_3_Na [M + Na]^+^ 258.1106, found 258.1108.
*Ethyl N-(benzyloxycarbonyl)butanimidate* (**2g**) [43]. Pale yellow oil; 95% yield (1.899 g). ^1^H-NMR: (400 MHz, CDCl_3_): δ 7.42–7.28 (m, 5H), 5.19 (s, 2H), 4.15 (q, *J* = 7.2 Hz, 2H), 2.30 (t, *J* = 7.6 Hz, 2H), 1.57 (sext, *J* = 7.6 Hz, 2H), 1.28 (t, *J* = 7.2 Hz, 3H), 0.86 (t, *J* = 7.4 Hz, 3H). ^13^C-NMR: (100 MHz, CDCl_3_): δ 169.8, 161.3, 135.9, 128.5, 128.3, 68.0, 62.9, 34.4, 19.5, 13.8, 13.6. HMRS (ESI) *m/z*: calcd for C_14_H_19_NO_3_Na [M + Na]^+^ 272.1263, found 272.1264.
*Ethyl N-(benzyloxycarbonyl)-2-methylpropanimidate* (**2h**). Pale yellow oil; 79% yield (1.580 g). ^1^H-NMR (400 MHz, CDCl_3_): δ 7.41–7.30 (m, 5H), 5.18 (s, 2H), 4.12 (q, *J* = 7.2 Hz, 2H), 2.72 (sept, *J* = 7.0 Hz, 1H), 1.27 (t, *J* = 7.2 Hz, 3H), 1.12 (d, *J* = 6.8 Hz, 6H).^13^C-NMR (100 MHz, CDCl_3_): δ 172.2, 161.2, 136.0, 128.5, 128.4, 128.2, 68.1, 62.9, 32.8, 19.5, 13.7. HMRS (ESI) *m/z*: calcd for C_14_H_20_NO_3_ [M *+* H]^+^ 250.1443 found 250.1445.
*Ethyl N-(benzyloxycarbonyl)pentanimidate* (**2i**) [43]. Yellow oil; 91% yield (1.912 g). ^1^H-NMR (400 MHz, CDCl_3_): δ 7.42–7.29 (m, 5H), 5.18 (s, 2H), 4.14 (q, *J* = 7.2 Hz, 2H), 2.32 (t, *J* = 7.8 Hz, 2H), 1.53 (qu, *J* = 7.6 Hz, 2H), 1.28 (t, *J* = 7.2 Hz, 3H), 1.25 (sext, *J* = 7.2 Hz, 2H) 0.85 (t, *J* = 7.2 Hz, 3H) ^13^C-NMR (100 MHz, CDCl_3_): δ 170.0, 161.4, 135.9, 128.5, 128.3, 68.1, 63.0, 32.4, 28.2, 22.3, 13.9, 13.6. HMRS (ESI) *m/z*: calcd for C_15_H_21_NO_3_Na [M + Na]^+^ 286.1419, found 286.1417.
*Ethyl N-(benzyloxycarbonyl)-3-methylbutanimidate* (**2j**). Yellow oil; 99% yield (2.083 g). ^1^H-NMR (400 MHz, CDCl_3_): δ 7.42–7.26 (m, 5H), 5.18 (s, 2H), 4.15 (q, *J* = 7.0 Hz, 2H), 2.02 (d, *J* = 7.2 Hz, 2H), 1.97 (sept, *J* = 6.8 Hz, 1H). 1.28 (t, *J* = 7.2 Hz, 3H), 0.85 (d, *J* = 6.8 Hz, 6H). ^13^C-NMR (100 MHz, CDCl_3_): δ 169.1, 161.3, 135.9, 128.6, 128.5, 128.4, 68.0, 62.9, 41.1, 26.3, 22.2, 13.9. HMRS (ESI) *m/z*: calcd for C_15_H_22_NO_3_ [M + H]^+^ 264.1600, found 264.1599.
*Ethyl N-(acetyl)-3-methylbutanimidate* (**2k**). Pale yellow oil; 99% yield (1.358 g). ^1^H-NMR (400 MHz, CDCl_3_): δ 4.09 (q, *J =* 7.1 Hz, 2H), 2.20 (d, *J =* 7.2 Hz, 2H), 2.16 (s, 3H) 2.05 (m, 1H), 1.28 (t, *J =* 7.0 Hz, 3H), 0.95 (d, *J =* 6.8 Hz, 6H). ^13^C-NMR (100 MHz, CDCl_3_): δ 183.4, 162.2, 62.4, 41.0, 26.7, 26.1, 22.3, 13.9. HRMS (ESI) *m/z*: calcd for C_9_H_18_NO_2_ [M + H]^+^ 172.1338, found 172.1343.
*Ethyl N-(benzyloxycarbonyl)-2-methoxyacetimidate* (**2l**). Pale yellow oil; 74% yield (1.481 g). ^1^H-NMR (400 MHz, CDCl_3_): δ 7.42–7.30 (m, 5H), 5,17 (s, 2H), 4.20 (q, *J* = 7.2 Hz, 2H), 4.10 (s, 2H), 3.24 (s, 3H), 1.30 (t, *J* = 7.2 Hz, 3H). ^13^C-NMR (100 MHz, CDCl_3_): δ 164.4, 160.6, 135.9, 128.7, 128.4, 128.2, 69.7, 68.0, 63.5, 59.6, 13.8. HMRS (ESI) *m/z*: calcd for C_13_H_17_NO_4_Na [M + Na]^+^ 274.1055, found 274.1057.
*EthylN-(benzyloxycarbonyl)benzimidate* (**2m**). Pale yellow oil; 58% yield (1.316 g). ^1^H-NMR (400 MHz, CDCl_3_): δ 7.55–7.50 (m, 5H), 5.12 (s, 2H), 4.34 (q, *J* = 7.0 Hz, 2H), 1.34 (t, *J* = 7.0 Hz, 3H). ^13^C-NMR (100 MHz, CDCl_3_): δ 167.1, 161.9, 135.6, 128.53, 128.51, 128.3, 68.4, 64.5, 13.8. HRMS (ESI) *m/z*: calcd for C_17_H_18_NO_3_ [M + H]^+^ 284.1287, found 284.1290.
*Ethyl N-(benzyloxycarbonyl)-2-(4-methoxyphenyl)acetimidate* (**2n**). Yellow oil; 96% yield (2.519 g). ^1^H-NMR (400 MHz, CDCl_3_): δ 7.36–7.32 (m, 5H), 7.11–7.09 (m, 2H), 6.80–6.76 (m, 2H), 5.13 (s, 2H), 4.15 (q, *J* = 7.2 Hz, 2H), 3.77 (s, 3H), 3.61 (s, 2H), 1.25 (t, *J* = 7.2 Hz, 3H). ^13^C-NMR (100 MHz, CDCl_3_): δ 168.06, 161.19, 158.62, 135.76, 130.26, 128.57, 128.51, 128.31, 113.90, 68.17, 63.42, 55.20, 37.96, 13.79. HMRS (ESI) *m/z*: calcd for C_19_H_22_NO_4_ [M + H]^+^ 328.1549, found 328.1546.

Step 2: Transformation of ethyl *N*-acylimidates **2** into diethyl 1-(*N*-acylamino)-1-ethoxyalkylphosphonates **3** was carried out according to the modified protocol given by Kuźnik et al. [33]. Potassium carbonate (4 wt % H_2_O) (2.7 mmol, 373 mg, 1.35 eq.) and 18-crown-6 (0.24 mmol, 63 mg, 0.12 eq.) were added to the solution of ethyl *N*-acylimidate **2** (2 mmol, 1 eq.) in hexane (6.4 mL). Then, diethyl phosphite (2.4 mmol, 331 mg, 0.31 mL, 1.2 eq. or 4.0 mmol, 552 mg, 0.51 mL, 2 eq. or 6.0 mmol, 829 mg, 0.77 mL, 3 eq.) was added dropwise. The reaction mixture was stirred vigorously at room or reduced temperature for the appropriate time period (Table 1). Then, K_2_CO_3_ was filtered off, and crude product was isolated by washing first with hexane and then with CH_2_Cl_2_. The crude product was further purified by column chromatography on silica gel using the mixture of CH_2_Cl_2_/MeOH/Et_3_N (100:1:1) as the eluent.

*Diethyl 1-(N-benzyloxycarbonylamino)-1-ethoxyethylphosphonate* (**3a**). White solid; 94% yield (338 mg); mp 69.4 to 71.0 °C. ^1^H-NMR (400 MHz, CDCl_3_): δ 7.39–7.30 (m, 5H), 5.71 (br d, *J =* 7.9 Hz, 1H), 5.09 (ABq, *J =* 12.2 Hz, 2H), 4.24–4.12 (m, 4H)^a^, 3.67–3.60 (m, 2H), 1.90 (d, *J =* 15.0 Hz, 3H), 1.34 (t, *J* = 7.2 Hz, 3H) and 1.32 (t, *J =* 7.2 Hz, 3H)^b^, 1.17 (t, *J* = 7.0, 3H). ^13^C-NMR (100 MHz, CDCl_3_): δ 154.6 (d, *J* = 16.4 Hz), 136.2, 128.5, 128.2, 128.1, 84.4 (d, *J* = 196.8 Hz), 66.7, 63.8 (d, *J* = 6.9 Hz), 63.4 (d, *J* = 6.9 Hz), 58.5 (d, *J* = 8.0 Hz), 18.9, 16.4 (d, *J* = 5.3 Hz), 15.4. ^31^P-NMR (162 MHz, CDCl_3_): δ 18.5. IR (ATR): 3203, 1717, 1541, 1224, 1047, 960, 750 cm^−1^. HMRS (ESI) *m/z*: calcd for C_16_H_27_NO_6_P [M + H]^+^ 360.1576, found 360.1578. ^a^Overlapping signals of P(O)(O**CH_2_**CH_3_)_2_ groups. ^b^Overlapping signals of P(O)(OCH_2_**CH_3_**)_2_ groups.
*Diethyl 1-(N-pivaloylamino)-1-ethoxyethylphosphonate* (**3b**). Colorless oil; 74% yield (230 mg). ^1^H-NMR (400 MHz, CDCl_3_): δ 6.41 (br d, *J* = 7.5 Hz, 1H), 4.26–4.14 (m, 4H)^a^, 3.67–3.59 (m, 2H), 1.95 (d, *J* = 15.4 Hz, 3H), 1.35 (t, *J* = 7.0 Hz, 3H) and 1.34 (t, *J* = 7.0 Hz, 3H)^b^, 1.22 (s, 9H), 1.19 (t, *J* = 7.0 Hz, 3H). ^13^C-NMR (100 MHz, CDCl_3_): δ 178.8 (d, *J* = 9.9 Hz), 84.9 (d, *J* = 194.5 Hz), 63.9 (d, *J* = 6.9 Hz), 63.1 (d, *J* = 6.9 Hz), 58.6 (d, *J* = 9.5 Hz), 39.9, 27.5, 18.7, 16.5 (d, *J* = 5.3 Hz), 16.4 (d, *J* = 5.4 Hz), 15.5. ^31^P-NMR (162 MHz, CDCl_3_): δ 19.1. IR (ATR): 3283, 1676, 1519, 1244, 1021, 958 cm^−1^. HMRS (ESI) *m/z*: calcd for C_13_H_29_NO_5_P [M + H]^+^ 310.1783, found 310.1790. ^a^Overlapping signals of P(O)(O**CH_2_**CH_3_)_2_ groups. ^b^Overlapping signals of P(O)(OCH_2_**CH_3_**)_2_ groups.
*Diethyl 1-(N-benzyloxycarbonylamino)-1-ethoxymethylphosphonate* (**3c**). Colorless oil; 93% yield (321 mg). ^1^H-NMR (400 MHz, CDCl_3_): δ 7.39–7.32 (m, 5H), 5.65 (dd, *J =* 10.8, 4.3 Hz, 1H), 5.24 (dd, *J*_1_ = 10.8 Hz, *J*_2_ = 9.3 Hz, 1H), 5.15 (ABq, *J =* 12.2 Hz, 2H), 4.24–4.11 (m, 4H)^a^, 3.81–3.74 (m, 1H), 3.65–3.57 (m, 1H), 1.33 (t, *J* = 7.2 Hz, 3H) and 1.29 (t, *J* = 7.0 Hz, 3H)^b^, 1.22 (t, *J* = 7.2 Hz, 3H). ^13^C-NMR (100 MHz, CDCl_3_): δ 156.0 (d, *J* = 12.2 Hz), 135.9, 128.6, 128.3, 128.1, 77.4 (d, *J =* 201.1 Hz), 67.4, 65.4 (d, *J* = 12.9 Hz), 63.7 (d, *J* = 6.5 Hz), 63.2 (d, *J* = 6.9 Hz), 16.38 (d, *J* = 5.3 Hz) and 16.36 (d, *J* = 5.3 Hz)^a^, 14.9. ^31^P-NMR (162 MHz, CDCl_3_): δ 15.9. IR (ATR): 3221, 1720, 1526, 1229, 1026, 977, 752 cm^−1^. HMRS (ESI) *m/z*: calcd for C_15_H_25_NO_6_P [M + H]^+^ 346.1419, found 346.1426. ^a^Overlapping signals of P(O)(O**CH_2_**CH_3_)_2_ groups. ^b^Overlapping signals of P(O)(OCH_2_**CH_3_**)_2_ groups.
*Diethyl 1-(N-benzyloxycarbonylamino)-1-ethoxy-2-phenylethylphosphonate* (**3d**). White solid; 88% yield (383 mg); mp 78.2 to 79.5 °C. ^1^H-NMR (400 MHz, CDCl_3_): δ 7.38–7.17 (m, 10H), 5.81 (br d, *J* = 10.6 Hz, 1H), 5.14 (ABq, *J* = 12 Hz, 2H), 4.09–3.68 (m, 7H)^a^, 3.41 (dd, *J*_1_ = 14.4 Hz, *J*_2_ = 11.1 Hz, 2H), 1.23 (t, *J =* 7.2 Hz, 3H) and 1.21 (t, *J* = 7.2 Hz, 3H)^b^, 1.05 (t, *J =* 7.0 Hz, 3H). ^13^C-NMR (100 MHz, CDCl_3_): δ 154.6 (d, *J* = 16.4 Hz), 136.1, 135.6 (d, *J* = 3.6 Hz), 131.2, 128.5, 128.24, 128.19, 127.6, 126.5, 87.1 (d, *J* = 186.5 Hz), 66.8, 63.2 (d, *J* = 7.2 Hz), 62.9 (d, *J* = 7.2 Hz), 59.4 (d, *J =* 4.6 Hz), 39.1 (d, *J* = 2.9 Hz), 16.3 (d, *J* = 5.8 Hz), 16.0 (d, *J =* 6.1 Hz), 15.2. ^31^P-NMR (162 MHz, CDCl_3_): δ 17.5. IR (ATR): 3204, 1726, 1548, 1245, 1019, 959, 754, 701 cm^−1^. HMRS (ESI) *m/z*: calcd for C_22_H_30_NO_6_NaP [M + Na]^+^ 458.1708, found 458.1704. ^a^Overlapping signals of C_α_C**H**_2_C_6_H_5_ and P(O)(O**CH_2_**CH_3_)_2_ groups. ^b^Overlapping signals of P(O)(OCH_2_**CH_3_**)_2_ groups.
*Diethyl 1-(N-acetylamino)-1-ethoxy-2-phenylethylphosphonate* (**3e**). White solid; 53% yield (183 mg); mp 92.8 to 94.3 °C. ^1^H-NMR (400 MHz, CDCl_3_): δ 7.33–7.20 (m, 5H), 6.12 (br d, *J* = 11.4 Hz, 1H), 4.12–3.92 (m, 5H)^a^, 3.87–3.71 (m, 2H), 3.39 (dd, *J*_1_ = 14.5 Hz, *J*_2_ = 9.1 Hz, 1H), 2.04 (s, 3H), 1.29 (t, *J* = 7.0 Hz, 3H), 1.23 (t, *J* = 7.0 Hz, 3H), 1.10 (t, *J* = 7.0 Hz, 3H). ^13^C-NMR (100 MHz, CDCl_3_): δ 170.3 (d, *J* = 9.2 Hz), 135.6 (d, *J* = 4.0 Hz), 131.1, 127.7, 126.6, 87.6 (d, *J* = 185.9 Hz), 63.4 (d, *J* = 7.2 Hz), 62.8 (d, *J* = 7.2 Hz), 59.9 (d, *J =* 5.3 Hz), 38.9, 24.5, 16.4 (d, *J =* 6.1 Hz), 16.1 (d, *J* = 6.1 Hz), 15.2. ^31^P-NMR (162 MHz, CDCl_3_): δ 17.9. IR (ATR): 3185, 1670, 1548, 1218, 1029, 962, 749, 696 cm^−1^. HMRS (ESI) *m/z*: calcd for C_16_H_27_NO_5_P [M + H]^+^ 344.1627, found 344.1627. ^a^Overlapping signals of C_α_C**H**_2_C_6_H_5_ and P(O)(O**CH_2_**CH_3_)_2_ groups.
*Diethyl 1-(N-benzyloxycarbonylamino)-1-ethoxypropylphosphonate* (**3f**). White solid; 82% yield (306 mg); mp 59.4 to 60.8 °C. ^1^H-NMR (400 MHz, CDCl_3_): δ 7.38–7.30 (m, 5H), 5.77 (d, *J* = 8.0 Hz, 1H), 5.09 (ABq, *J* = 10.0 Hz, 2H), 4.24–4.11 (m, 4H)^a^, 3.68–3.56 (m, 2H), 2.61 (ddq, *J*_1_ = 23.4 Hz, *J*_2_ = 15.0 Hz, *J*_3_ = 7.5 Hz, 1H), 2.25 (tq, *J*_1_ = 14.9 Hz, *J*_2_ = 7.5 Hz, 1H), 1.33 (t, *J* = 7.0 Hz, 3H) and 1.32 (t, *J* = 7.0 Hz, 3H)^b^, 1.17 (t, *J* = 7.0 Hz, 3H), 1.02 (t, *J* = 7.6 Hz, 3H), ^13^C-NMR (100 MHz, CDCl_3_): δ 154.4 (d, *J =* 16.2), 136.2, 128.5, 128.3, 128.1, 87.7 (d, *J* = 189.5 Hz), 66.7, 63.6 (d, *J* = 7.2 Hz), 63.1 (d, *J* = 7.1 Hz), 58.4 (d, *J* = 7.2 Hz), 25.2, 16.4 (d, *J* = 5.7 Hz), 15.3, 8.5 (d, *J* = 2.1 Hz). ^31^P-NMR (162 MHz, CDCl_3_): δ 19.0. IR (ATR): 3253, 1231, 1024, 773 cm^−1^. HMRS (ESI) *m/z*: calcd for C_17_H_28_NO_6_NaP [M + Na]^+^ 396.1552, found 396.1545. ^a^Overlapping signals of P(O)(O**CH_2_**CH_3_)_2_ groups. ^b^Overlapping signals of P(O)(OCH_2_**CH_3_**)_2_ groups.
*Diethyl 1-(N-benzyloxycarbonylamino)-1-ethoxybutylphosphonate* (**3g**). Colorless oil; 68% yield (263 mg). ^1^H-NMR (400 MHz, CDCl_3_): δ 7.39–7.31 (m, 5H), 5.78 (br d, *J* = 8.0 Hz, 1H), 5.08 (ABq, *J* = 12.2 Hz, 2H), 4.23–4.11 (m, 4H)^a^, 3.68–3.55 (m, 2H), 2.61–2.47 (m, 1H), 2.23–2.11 (m, 1H), 1.54–1.44 (m, 2H), 1.33 (t, *J* = 7.0 Hz, 3H) and 1.32 (t, *J*_1_ = 7.0 Hz, 3H)^b^, 1.16 (t, *J* = 7.0 Hz, 3H), 0.93 (t, *J* = 7.4 Hz, 3H). ^13^C-NMR (100 MHz, CDCl_3_): δ 154.4 (d, *J* = 16.1 Hz), 136.2, 128.5, 128.1, 128.0, 87.3 (d, *J* = 189.7 Hz), 66.6, 63.6 (d, *J =* 7.1 Hz), 63.1 (d, *J =* 7.0 Hz), 58.4 (d, *J* = 7.4 Hz), 34.4, 17.2 (d, *J =* 2.0 Hz), 16.4 (d, *J =* 5.5 Hz), 15.3, 14.4. ^31^P-NMR (162 MHz, CDCl_3_): δ 19.0. IR (ATR): 2976, 1737, 1499, 1240, 1019, 969, 742 cm^−1^. HMRS (ESI) *m/z*: calcd for C_18_H_30_NO_6_NaP [M + Na]^+^ 410.1708, found 410.1706. ^a^Overlapping signals of P(O)(O**CH_2_**CH_3_)_2_ groups. ^b^Overlapping signals of P(O)(OCH_2_**CH_3_**)_2_ groups.
*Diethyl 1-(N-benzyloxycarbonylamino)-1-ethoxy-2-methylpropylphosphonate* (**3h**). White solid; 53% yield (205 mg); mp 54.2 to 55.5 °C. ^1^H-NMR (400 MHz, CDCl_3_): δ 7.37–7.31 (m, 5H), 5.91 (br d, *J =* 10.8 Hz, 1H), 5.09 (ABq, *J* = 12.2 Hz, 2H), 4.21–4.12 (m, 4H)^a^, 3.68–3.56 (m, 2H), 3.19 (dsept, *J*_1_ = 32.6 Hz, *J*_2_ = 7.0 Hz 1H), 1.33 (t, *J* = 7.0 Hz, 3H), 1.15 (t, *J* = 7.0 Hz, 3H), 1.12 (d, *J* = 6.8 Hz, 3H), 1.06 (d, *J* = 6.8 Hz, 3H). ^13^C-NMR (100 MHz, CDCl_3_): δ 154.6 (d, *J* = 18.2 Hz), 136.3, 128.5, 128.2, 128.1, 90.2 (d, *J* = 185.8 Hz), 66.7, 63.4 (d, *J =* 7.2 Hz), 62.9 (d, *J =* 7.6 Hz), 58.6 (d, *J* = 6.5 Hz), 31.4, 17.7 (d, *J* = 3.1 Hz), 17.5, 16.4 (d, *J* = 5.3 Hz), 15.3. ^31^P-NMR (162 MHz, CDCl_3_): δ 19.7. IR (ATR): 3218, 1723, 1544, 1239, 1023, 976, 745 cm^−1^. HMRS (ESI) *m/z*: calcd for C_18_H_31_NO_6_P [M + H]^+^ 388.1889, found 388.1890. ^a^Overlapping signals of P(O)(O**CH_2_**CH_3_)_2_ groups.
*Diethyl 1-(N-benzyloxycarbonylamino)-1-ethoxypentylphosphonate* (**3i**). White solid; 54% yield (217 mg); mp 53.1 to 54.7 °C. ^1^H-NMR (400 MHz, CDCl_3_): δ 7.36–7.28 (m, 5H), 5.78 (br d, *J* = 8.4 Hz, 1H), 5.08 (ABq, *J* = 12.2 Hz, 2H), 4.23–4.11 (m, 4H)^a^, 3.68–3.56 (m, 2H), 2.61–2.47 (m, 1H), 2.26–2.14 (m, 1H), 1.49–1.41 (m, 2H), 1.35–1.26 (m, 2H), 1.33 (t, *J* = 7.0 Hz, 3H), 1.32 (t, *J* = 7.0 Hz, 3H), 1.16 (t, *J* = 7.0 Hz, 3H), 0.91 (t, *J* = 7.2 Hz, 3H). ^13^C-NMR (100 MHz, CDCl_3_): δ 154.3 (d, *J* = 16.1 Hz), 136.2, 128.4, 128.1, 128.0, 87.3 (d, *J* = 189.5 Hz), 66.6, 63.5 (d, *J =* 7.2 Hz) and 63.1 (d, *J =* 7.2 Hz), 58.3 (d, *J* = 7.2 Hz), 31.9, 25.8 (d, *J* = 1.9 Hz), 22.9, 16.3 (d, *J =* 5.6 Hz), 15.2, 13.9. ^31^P-NMR (162 MHz, CDCl_3_): δ 19.0. IR (ATR): 2973, 1722, 1545, 1240, 1022, 985, 754 cm^−1^. HMRS (ESI) *m/z*: calcd for C_19_H_32_NO_6_NaP [M + Na]^+^ 424.1865, found 424.1863. ^a^Overlapping signals of C_α_CH_2_CH_2_**CH_2_**CH_3_ and P(O)(O**CH_2_**CH_3_)_2_ groups.
*Diethyl 1-(N-benzyloxycarbonylamino)-1-ethoxy-3-methylbutylphosphonate* (**3j**). Colorless oil; 32% yield (135.7 mg). ^1^H-NMR (400 MHz, CDCl_3_): δ 7.36–7.31 (m, 5H), 5.88 (br d, *J =* 8.8 Hz, 1H), 5.08 (ABq, *J* = 12.2 Hz, 2H), 4.25–4.11 (m, 4H)^a^, 3.61 (qd, *J* = 7.0, 1.0 Hz, 2H), 2.62 (ddd, *J*_1_ = 26.3 Hz, *J*_2_ = 15.0 Hz, *J*_3_ = 7.9 Hz, 1H), 2.09–2.03 (m, 1H), 1.96 (ddd, *J*_1_ = 15.0 Hz, *J*_2_ = 9.1 Hz, *J*_3_ = 4.3 Hz, 1H), 1.34 (t, *J* = 7.2 Hz, 3H) and 1.32 (t, *J* = 7.2 Hz, 3H)^b^, 1.16 (t, *J* = 7.0 MHz, 3H), 1.00 (d, *J* = 6.8 Hz, 3H), 0.94 (d, *J* = 6.8 Hz, 3H). ^13^C-NMR (100 MHz, CDCl_3_): δ 154.4 (d, *J* = 16.7 Hz), 136.2, 128.5, 128.14, 128.06, 87.9 (d, *J* = 188.2 Hz), 66.6, 63.8 (d, *J =* 7.2 Hz), 62.9 (d, *J =* 7.2 Hz), 58.5 (d, *J* = 7.2 Hz), 40.3, 24.5 (d, *J* = 4.6 Hz), 23.2, 16.40 (d, *J =* 5.7 Hz) and 16.38 (d, *J =* 5.7 Hz)^b^, 15.1. ^31^P-NMR (162 MHz, CDCl_3_): δ 19.2. IR (ATR): 3248, 1739, 1499, 1242, 1021, 967, 749 cm^−1^. HMRS (ESI) *m/z*: calcd for C_19_H_32_NO_6_NaP [M + Na]^+^ 424.1865, found 424.1862. ^a^Overlapping signals of P(O)(OCH_2_**CH_3_**)_2_ groups. ^a^Overlapping signals of P(O)(O**CH_2_**CH_3_)_2_ groups. ^b^Overlapping signals of P(O)(OCH_2_**CH_3_**)_2_ groups.
*Diethyl 1-(N-acetylamino)-1-ethoxy-3-methylbutylphosphonate* (**3k**). White solid; 64% yield (198 mg); mp 58.6 to 59.7 °C. ^1^H-NMR (400 MHz, CDCl_3_): δ 6.30 (br d, *J* = 7.6 Hz, 1H), 4.28–4.13 (m, 4H)^a^, 3.70–3.58 (m, 2H), 2.81–2.70 (m, 1H), 1.98–1.92 (m, 2H), 2.02 (s, 3H), 1.35 (t, *J =* 7.0 Hz, 3H) and 1.34 (t, *J* = 7.0 Hz, 3H)^b^, 1.18 (t, *J* = 7.2 Hz, 3H), 1.00 (d, *J* = 6.7 Hz, 3H), 0.95 (d, *J* = 6.7 Hz, 3H). ^13^C-NMR (100 MHz, CDCl_3_): δ 170.1 (d, *J* = 12.9 Hz), 88.8 (d, *J* = 187.3 Hz), 64.1 (d, *J* = 7.2 Hz), 62.7 (d, *J* = 7.2 Hz), 59.0 (d, *J* = 8.0 Hz), 39.7, 24.9, 24.7 (d, *J* = 3.0 Hz), 24.5, 23.1, 16.43 (d, *J* = 6.1 Hz) and 16.40 (d, *J =* 5.3 Hz)^b^, 15.1. ^31^P-NMR (162 MHz, CDCl_3_): δ 19.6. IR (ATR): 3197, 1670, 1541, 1224, 1070, 956, 759 cm^−1^. HMRS (ESI) *m/z*: calcd for C_13_H_29_NO_5_P [M + H]^+^ 310.1783, found 310.1776. ^a^Overlapping signals of P(O)(OCH_2_**CH_3_**)_2_ groups. ^a^Overlapping signals of P(O)(O**CH_2_**CH_3_)_2_ groups. ^b^Overlapping signals of P(O)(OCH_2_**CH_3_**)_2_ groups.
*Diethyl 1-(N-benzyloxycarbonylamino)-1-ethoxy-2-methoxyethylphosphonate* (**3l**). White solid; 91% yield (354 mg); mp 60.1 to 62.1 °C. ^1^H-NMR (400 MHz, CDCl_3_): δ 7.37–7.30 (m, 5H), 5.97 (br d, *J =* 11.0 Hz, 1H), 5.10 (ABq, *J* = 12.0 Hz, 2H), 4.26–4.15 (m, 5H)^a^, 3.92 (dd, *J*_1_ = 10.6 Hz, *J*_2_ = 9.3 Hz, 2H), 3.75–3.61 (m, 2H), 3.41 (s, 3H), 1.34 (td, *J*_1_ = 7.2 Hz, *J*_2_ = 0.4 Hz, 3H) and 1.33 (td, *J*_1_ = 7.2 Hz, *J*_2_ = 0.4 Hz, 3H)^b^, 1.18 (t, *J* = 7.2 Hz, 3H). ^13^C-NMR (100 MHz, CDCl_3_): δ 154.4 (d, *J* = 14.3 Hz), 136.1, 128.5, 128.2, 128.1, 86.0 (d, *J* = 188.8 Hz), 72.5, 67.0, 63.6 (d, *J =* 7.2 Hz), 63.5 (d, *J =* 6.9 Hz), 59.4, 59.3 (d, *J* = 6.0 Hz), 16.4 (d, *J =* 5.7 Hz), 15.4. ^31^P-NMR (162 MHz, CDCl_3_): δ 17.9. IR (ATR): 3227, 2985, 1733, 1528, 1245, 1027, 987, 758 cm^−1^. HMRS (ESI) *m/z*: calcd for C_17_H_28_NO_7_NaP [M + Na]^+^ 412.1501, found 412.1494. ^a^Overlapping signals of C_α_C**H**_2_OMe and P(O)(O**CH_2_**CH_3_)_2_ groups.
*Diethyl 1-(N-benzyloxycarbonylamino)-1-ethoxy-1-phenylmethylphosphonate* (**3m**). White solid; 82% yield (345 mg); mp 96.6 to 97.6 °C. ^1^H-NMR (400 MHz, CDCl_3_): δ 7.55–7.52 (m, 2H) and 7.35–7.28 (m, 8H)^a^, 6.23 (d, *J* = 10. Hz, 1H), 5.04 (ABq, *J* = 12.4 Hz, 2H), 4.14–3.67 (m, 6H)^b^, 1.26 (t, *J* = 7.0 Hz, 3H) and 1.25 (td, *J*_1_ = 7.0 Hz, *J*_2_ = 0.8 Hz, 3H)^c^, 1.17 (t, *J* = 7.2 Hz, 3H). ^13^C-NMR (100 MHz, CDCl_3_): δ 154.4 (d, *J* = 20.9 Hz), 136.0, 128.4, 128.14, 128.11, 127.75, 127.72, 127.41, 127.37, 87.3 (d, *J* = 185.8 Hz), 67.0, 64.5 (d, *J* = 7.2 Hz), 63.8 (d, *J* = 7.7 Hz), 59.8 (d, *J* = 6.1 Hz), 16.3 (d, *J* = 5.7 Hz), 16.2 (d, *J* = 5.6 Hz), 15.3. ^31^P-NMR (162 MHz, CDCl_3_): δ 15.4. IR (ATR): 3195, 1729, 1541, 1234,1027, 957, 737 cm^−1^. HMRS (ESI) *m/z*: calcd for C_21_H_28_NO_6_NaP [M + Na]^+^ 445.1552, found 444.1546. ^a^Overlapping signals of **Ph**CH_2_O and C_α_**Ph** groups. ^a^Overlapping signals of Ph**CH_2_**O and P(O)(O**CH_2_**CH_3_)_2_ groups. ^c^Overlapping signals of P(O)(OCH_2_**CH_3_**)_2_ groups.
*Diethyl 1-(N-benzyloxycarbonylamino)-1-ethoxy-2-(4-methoxyphenyl)ethylphosphonate* (**3n**). White solid; 70% yield (326 mg); mp 80.1 to 81.6 °C. ^1^H-NMR (400 MHz, CD_3_CN): δ 7.40–7.33 (m, 5H), 7.20–7.16 (m, 2H), 6.79–6.75 (m, 2H), 5.91 (br d, *J* = 10.0 Hz, 1H), 5.11 (s, 2H), 4.05–3.87 (m, 4H)^a^, 3.80–3.72 (m, 1H)^b^, 3.74 (s, 3H)^b^, 3.67–3.57 (m, 2H), 1.17 (td, *J*_1_ = 7.0, *J*_2_ = 0.5 Hz, 3H), 1.13 (t, *J* = 7.0 Hz, 3H), 1.10 (td, *J*_1_ = 7.0 Hz, *J*_2_ = 0.5 Hz, 3H). ^13^C-NMR (100 MHz, CDCl_3_): δ 159.5, 155.4 (d, *J* = 14.1 Hz), 138.0, 133.1, 129.5, 129.1, 129.0, 128.5 (d, *J* = 3.8 Hz), 114.0, 88.3 (d, *J* = 187.3 Hz), 67.2, 63.78 (d, *J* = 7.2 Hz) and 62.73 (d, *J* = 7.2 Hz)^a^, 60.1 (d, *J* = 4.2 Hz), 55.8, 39.1 (d, *J* = 5.0 Hz), 15.7 (d, *J* = 5.7 Hz) and 15.6 (d, *J* = 5.7 Hz), 14.6. ^31^P-NMR (162 MHz, CD_3_CN): δ 17.29. IR (ATR): 3197, 2973, 1720, 1514, 1255, 1019, 972, 738, 697 cm^−1^. HMRS (ESI) *m/z*: calcd for C_23_H_32_NO_7_NaP [M + Na]^+^ 488.1814, found 488.1812. ^a^Overlapping signals of P(O)(O**CH_2_**CH_3_)_2_ groups. ^b^Overlapping signals of C_6_H_4_O**CH_3_** and C_α_C**H_2_**C_6_H_4_CH_3_ groups.
Synthesis of diethyl 1-(*N*-benzyloxycarbonylamino)-1-triphenylphosphonium-methylphosphonate tetrafluoroborate **4c**.

Diethyl 1-(*N*-benzyloxycarbonylamino)-1-triphenylphosphoniummethylphosphonate tetrafluoroborate **4c** was synthesized as previously described by Kuźnik et al. [33]. In brief, triphenylphosphonium tetrafluoroborate (1.12 mmol, 392 mg, 1.12 eq.) and diethyl 1-(*N*-benzyloxycarbonylamino)-1-ethoxymethylphosphonate **3c** (1.0 mmol, 345 mg, 1 eq.) were dissolved in dry CH_2_Cl_2_ (5 mL) for homogenization. The solvent was evaporated, and the residue was heated in an oil bath at 85 °C under reduced pressure for 5 h. The resulting phosphonium salt **4c** was used in the next step without further purification.

*Diethyl 1-(N-benzyloxycarbonylamino)-1-triphenylphosphoniummethylphosphonate* (**4c**) Colorless crystals; 95% yield (615 mg), mp 163.7 to 164.9 °C. ^1^H-NMR (400 MHz, CDCl_3_): δ 7.85–7.58 (m, 16H)^a^ 7.33–7.25 (m, 5H), 5.96 (ddd, *J* = 22.7, 16.6, 9.9 Hz, 1H), 4.96 (ABq, *J* = 12.6 Hz, 2H), 4.17–4.07 (m, 2H), 3.95–3.84 (m, 2H), 1.23 (t, *J* = 7.1 Hz, 3H), 1.15 (t, *J* = 7.1 Hz, 3H). ^13^C-NMR (100 MHz, CDCl_3_): δ 156.3, 135.7, 135.1 (d, *J* = 3.1 Hz), 134.8 (d, *J* = 10.3 Hz), 130.1 (d, *J* = 13.0 Hz), 128.4, 128.1, 128.0, 116.9 (d, *J* = 84.7 Hz), 67.9, 65.1 (d, *J* = 7.6 Hz), 64.9 (d, *J* = 6.9 Hz), 48.1 (dd, *J* = 152.8, 48.5 Hz), 16.1 (d, *J* = 6.1 Hz), 16.0 (d, *J* = 5.0 Hz). ^31^P-NMR (162 MHz, CDCl_3_): 27.5 (d, *J* = 37.5 Hz), 11.2 (d, *J* = 37.5 Hz). IR (ATR) 3213, 1712, 1522, 1273, 1008, 747, 688. HRMS (ESI) *m/z*: calcd for C_31_H_34_NO_5_P_2_ [M + H]^+^ 562.1912, found 562.1912. ^a^Overlapping signals of ^+^P**Ph**_3_ and N**H** groups.

### 3.3. General Procedure for the One-Pot Synthesis of Tetraethyl 1-(N-acylamino)alkylene-1,1-bisphosphonates **5**

Triethyl phosphite (1.5 mmol, 249 mg, 0.26 mL, 1.5 eq.) was added to a solution of diethyl 1-(*N*-acylamino)-1-ethoxyalkylphosphonate **3** (1.0 mmol) and triphenylphosphonium tetrafluoroborate (1.05–1.08 mmol, 368 mg–378 mg, 1.05–1.08 eq.) in dry MeCN or CH_2_Cl_2_ (4 mL). The reaction mixture was heated or left at room temperature for the appropriate time period (Scheme 4). Then, the solvent was evaporated under reduced pressure, and the residue was extracted with toluene (3 to 5 times). After evaporation of the toluene, the crude product **5** was purified by column chromatography on silica gel using the mixture of CH_2_Cl_2_/MeOH (20:1) as the eluent.

The synthesis of compound **5l** was carried out in an analogous manner but with larger excess of triphenylphosphonium tetrafluoroborate (1.2 mmol, 420 mg, 1.2 eq.) and the addition of Hünig’s base (0.5 mmol, 65 mg, 87 μL, 0.5 eq.).

Synthesis of tetraethyl 1-(*N*-benzyloxycarbonylamino)methylene-1,1-bisphosphonate **5c**.

Triethyl phosphite (1.5 mmol, 249 mg, 258 μL, 1.5 eq.) and Hünig’s base (0.42 mmol, 54 mg, 73 μL, 0.42 eq.) were added to a solution of crude diethyl 1-(*N*-benzyloxycarbonylamino)-1-triphenylphosphoniummethylphosphonate tetrafluoroborate **4c** (1.0 mmol, 649 mg, 1 eq.) in dry MeCN (4 mL). The mixture was heated at 70 °C for 8 h. The product **5c** was isolated and purified in an analogous manner as described in the procedure above.

*Tetraethyl 1-(N-benzyloxycarbonylamino)ethylene-1,1-bisphosphonate* (**5a**). Colorless crystals; 95% yield (430 mg), mp 47.1 to 48.7 °C. ^1^H-NMR (400 MHz, CDCl_3_): δ 7.36–7.31 (m, 5H), 5.40 (br t, *J* = 3.4 Hz, 1H), 5.07 (s, 2H), 4.25–4.12 (m, 8H)^a^, 1.98 (t, *J* = 17.0 Hz, 3H), 1.33 (t, *J* = 7.2 Hz, 6H) and 1.31 (t, *J* = 7.2 Hz, 6H)^b^. ^13^C-NMR (100 MHz, CDCl_3_): 154.3, 136.3, 128.4, 128.11, 128.09, 66.7, 63.83 (d, *J =* 3.4 Hz) and 63.80 (d, *J =* 3.4 Hz) and 63.75 (d, *J* = 3.4 Hz) and 63.72 (d, *J* = 3.4 Hz)^a^, 55.8 (t, *J* = 146.9 Hz), 16.5–16.3 (m)^b^, 16.2 (br t, *J* = 4.1 Hz). ^31^P-NMR (162 MHz, CDCl_3_): 19.6. IR (ATR) 3218, 1714, 1537, 1229, 1016, 958, 750. HRMS (ESI) *m/z*: calcd for C_18_H_32_NO_8_P_2_ [M + H]^+^ 452.1603, found 452.1610. ^a^Overlapping signals of P(O)(O**CH_2_**CH_3_)_2_ groups. ^b^Overlapping signals of P(O)(OCH_2_**CH_3_**)_2_ groups.
*Tetraethyl 1-(N-pivaloylamino)ethylene-1,1-bisphosphonate* (**5b**). Colorless crystals; 62% yield (247 mg), mp 50.8 to 52.3 °C. ^1^H-NMR (400 MHz, CDCl_3_): δ 6.19 (br t, *J* = 4.6 Hz, 1H), 4.28–4.19 (m, 8H)^a^, 2.01 (t, *J* = 17.0 Hz, 3H), 1.35 (t, *J* = 7.0 Hz, 12H), 1.20 (s, 9H). ^13^C-NMR (100 MHz, CDCl_3_): δ 177.7 (t, *J* = 5.1 Hz), 63.76 (d, *J* = 3.4 Hz) and 63.73 (d, *J* = 3.4 Hz) and 63.67 (d, *J* = 3.4 Hz) and 63.64 (d, *J* = 3.4 Hz)^a^, 56.7 (t, *J* = 144.9 Hz), 39.8, 27.4, 16.7 (t, *J* = 4.5 Hz), 16.5–16.4 (m)^b^. ^31^P-NMR (162 MHz, CDCl_3_): 20.0. IR (ATR) 3276, 1677, 1515, 1233, 1016, 945. HRMS (ESI) *m/z*: calcd for C_15_H_34_NO_7_P_2_ [M + H]^+^ 402.1811, found 402.1813. ^a^Overlapping signals of P(O)(O**CH_2_**CH_3_)_2_ groups. ^b^Overlapping signals of P(O)(OCaH_2_**CH_3_**)_2_ groups.
*Tetraethyl 1-(N-benzyloxycarbonylamino)methylene-1,1-bisphosphonate* (**5c**). Colorless crystals; 82% yield (357 mg), mp 59.8 to 60.7 °C. ^1^H-NMR (400 MHz, CDCl_3_): δ 7.36–7.31 (m, 5H), 5.32 (br d, *J* = 10.4 Hz, 1H), 5.15 (s, 2H), 4.59 (td *J*_1_ = 21.9, *J*_2_ *=* 10.4 Hz), 4.25–4.12 (m, 8H)^a^, 1.32 (t, *J =* 7.0 Hz, 6H) and 1.29 (t, *J =* 7.0 Hz, 6H)^b^. ^13^C-NMR (100 MHz, CDCl_3_): δ 155.5 (t, *J* = 4.9 Hz), 135.9, 128.5, 128.3, 128.1, 67.6, 63.5, 46.0 (t, *J* = 146.8 Hz), 16.3–16.2 (m)^b^. ^31^P-NMR (162 MHz, CDCl_3_): 16.3. IR (ATR) 3354, 1717, 1528, 1266, 1019, 977, 736. HRMS (ESI) *m/z*: calcd for C_17_H_29_NO_8_NaP_2_ [M + Na]^+^ 460.1266, found 460.1261. ^a^Overlapping signals of P(O)(O**CH_2_**CH_3_)_2_ groups. ^b^Overlapping signals of P(O)(OCH_2_**CH_3_**)_2_ groups.
*Tetraethyl 1-(N-benzyloxycarbonylamino)-2-phenylethylene-1,1-bisphosphonate* (**5d**). Colorless crystals; 86% yield (455 mg), mp 60.2 to 61.5 °C. ^1^H-NMR (400 MHz, CDCl_3_) δ 7.44–7.34 (m, 5H), 7.26–7.16 (m, 5H), 5.44 (t, *J* = 12.7 Hz, 1H), 5.18 (s, 2H), 4.30–4.15 (m, 4H)^a^, 4.13–4.03 (m, 2H), 3.98–3.88 (m, 2H), 3.58 (dd, *J* = 15.3, 11.7 Hz, 2H), 1.30 (t, *J* = 7.2 Hz, 6H), 1.19 (t, *J* = 7.2 Hz, 6H). ^13^C-NMR (100 MHz, CDCl_3_) δ 154.9 (t, *J* = 8.8 Hz), 136.4, 135.3 (t, *J* = 8.6 Hz), 131.2, 128.49, 128.45, 128.2, 127.7, 126.7, 67.1, 63.9 (d, *J* = 7.5 Hz), 63.0 (d, *J* = 7.4 Hz), 61.2 (t, *J* = 143.1 Hz), 35.5, 16.3 (d, *J* = 6.3 Hz), 16.2 (d, *J* = 6.2 Hz). ^31^P-NMR (162 MHz, CDCl_3_): 18.8. IR (ATR) 3224, 1711, 1534, 1266, 1022, 963, 752. HRMS (ESI) *m/z*: calcd for C_24_H_35_NO_8_NaP_2_ [M + Na]^+^ 550.1736, found 550.1732. ^a^Overlapping signals of P(O)(O**CH_2_**CH_3_)_2_ groups.
*Tetraethyl 1-(N-acetylamino)-2-phenylethylene-1,1-bisphosphonate* (**5e**). Colorless crystals; 59% yield (255 mg); mp 91.0 to 92.3 °C. ^1^H-NMR (400 MHz, CDCl_3_): δ 7.28–7.19 (m, 5H), 6.04 (br t, *J* = 13.3 Hz, 1H), 4.33–4.25 (m, 4H)^a^, 4.16–4.06 (m, 2H), 4.04–3.94 (m, 2H), 3.57 (dd, *J* = 15.3, 12.0 Hz, 2H), 2.05 (s, 3H), 1.35 (t, *J* = 7.1, Hz, 6H), 1.23 (t, *J* = 7.1 Hz, 6H). ^13^C-NMR (100 MHz, CDCl_3_): δ 169.9 (t, *J* = 7.3 Hz), 135.5 (t, *J* = 8.2 Hz), 131.1, 127.7, 126.9, 64.1 (d, *J* = 7.3 Hz), 62.9 (d, *J* = 7.6 Hz), 61.4 (t, *J* = 143.1 Hz), 35.2, 23.9, 16.4 (d, *J* = 6.2 Hz), 16.1 (d, *J* = 6.5 Hz). ^31^P-NMR (162 MHz, CDCl_3_): 19.2. IR (ATR) 3305, 2989, 1684, 1537, 1245, 1065, 1008, 962. HRMS (ESI) *m/z*: calcd for C_18_H_31_NO_7_NaP_2_ [M + Na]^+^ 458.1473, found 458.1467. ^a^Overlapping signals of P(O)(O**CH_2_**CH_3_)_2_ groups.
*Tetraethyl 1-(N-benzyloxycarbonylamino)propylene-1,1-bisphosphonate* (**5f**). Colorless crystals; 90% yield (417 mg), mp 61.6 to 62.8 °C. ^1^H-NMR (400 MHz, CDCl_3_) δ 7.37–7.31 (m, 5H), 5.47 (br t, *J* = 8.3 Hz, 1H), 5.08 (s, 2H), 4.27–4.15 (m, 8H)^a^, 2.42 (tq, *J*_1_ *=* 16.0 Hz, *J*_2_ *=* 7.7 Hz, 2H), 1.34 (t, *J =* 7.0 Hz, 6H) and 1.32 (t, *J =* 7.0 Hz, 6H)^b^, 1.11 (t, *J* = 7.4 Hz, 3H). ^13^C-NMR (100 MHz, CDCl_3_) δ 154.3 (t, *J* = 8.0 Hz), 136.4, 128.4, 128.1, 66.8, 63.67 (d, *J =* 3.5 Hz) and 63.64 (d, *J =* 3.5 Hz)^b^, 63.42 (d, *J =* 3.5 Hz) and 63.38 (d, *J =* 3.5 Hz)^a^, 60.4 (t, *J* = 144.4 Hz), 23.8 (t, *J* = 3.0 Hz), 16.5–16.3 (m)^b^, 9.1(t, *J* = 6.5 Hz). ^31^P-NMR (162 MHz, CDCl_3_): 20.0. IR (ATR) 3422, 1737, 1503, 1248, 1022, 968, 770. HRMS (ESI) *m/z*: calcd for C_19_H_33_NO_8_NaP_2_ [M + Na]^+^ 488.1579, found 488.1577. ^a^Overlapping signals of P(O)(O**CH_2_**CH_3_)_2_ groups. ^b^Overlapping signals of P(O)(OCH_2_**CH_3_**)_2_ groups.
*Tetraethyl 1-(N-benzyloxycarbonylamino)butylene-1,1-bisphosphonate* (**5g**). Colorless crystals; 95% yield (457 mg), mp 66.7 to 68.5 °C. ^1^H-NMR (400 MHz, CDCl_3_) δ 7.37–7.30 (m, 5H), 5.46 (br t, *J* = 8.2 Hz, 1H), 5.08 (s, 2H), 4.26–4.14 (m, 8H)^a^, 2.36–2.24 (m, 2H), 1.61–1.55 (m, 2H), 1.33 (t, *J* = 7.0 Hz, 6H) and 1.32 (t, *J* = 7.0 Hz, 6H)^b^, 0.93 (t, *J* = 7.3 Hz, 3H). ^13^C-NMR (100 MHz, CDCl_3_): δ 154.3 (t, *J* = 7.6 Hz), 136.4, 128.4, 128.1, 66.8, 63.66 (d, *J* = 3.5 Hz) and 63.63 (d, *J* = 3.5 Hz)^a^, 63.43 (d, *J* = 3.5 Hz) and 63.40 (d, *J =* 3.5 Hz)^a^, 60.1 (t, *J =* 143.5 Hz), 32.7 (t, *J =* 3.0 Hz), 17.7 (t, *J =* 6.2 Hz), 16.45 (d, *J* = 2.9 Hz) and 16.43 (d, *J =* 2.6 Hz) and 16.40 (d, *J* = 2.7 Hz) and 16.37 (d, *J* = 2.9 Hz)^b^, 14.5. ^31^P-NMR (162 MHz, CDCl_3_): 20.1. IR (ATR) 1735, 1499, 1243, 1017, 958, 740. HRMS (ESI) *m/z*: calcd for C_20_H_35_NO_8_NaP_2_ [M + Na]^+^ 502.1736, found 502.1731. ^a^Overlapping signals of P(O)(O**CH_2_**CH_3_)_2_ groups. ^b^Overlapping signals of P(O)(OCH_2_**CH_3_**)_2_ groups.
*Tetraethyl 1-(N-benzyloxycarbonylamino)-2-methylpropylene-1,1-bisphosphonate* (**5h**). Colorless oil; 72% yield (344 mg). ^1^H-NMR (400 MHz, CDCl_3_): δ 7.37–7.28 (m, 5H), 5.71 (t, *J =* 10.1 Hz, 1H), 5.09 (s, 2H), 4.28–4.13 (m, 8H)^a^, 3.06 (tsept, *J*_1_ = 23.6, *J*_2_ = 7.0 Hz 1H), 1.33 (t, *J =* 7.0 Hz, 6H) and 1.32 (t, *J =* 7.0 Hz, 6H)^b^, 1.22 (d, *J =* 6.9 Hz, 6H). ^13^C-NMR (100 MHz, CDCl_3_): δ 154.3 (t, *J =* 8.2 Hz), 136.4, 128.4, 128.0, 66.8, 64.6 (t, *J* = 139.2 Hz), 63.40 (d, *J* = 3.6 Hz) and 63.36 (d, *J* = 3.6 Hz)^a^, 63.11 (d, *J =* 3.5 Hz) and 63.08 (d, *J* = 3.6 Hz)^a^, 30.7, 18.8 (t, *J =* 4.3 Hz), 16.40 (d, *J =* 3.0 Hz) and 16.37 (d, *J =* 2.9 Hz) and 16.34 (d, *J =* 2.9 Hz) and 16.31 (d, *J =* 3.0 Hz)^b^. ^31^P-NMR (162 MHz, CDCl_3_): 20.7. IR (ATR) 3433, 1743, 1500, 1244, 1019, 966, 741. HRMS (ESI) *m/z*: calcd for C_20_H_36_NO_8_P_2_ [M + H]^+^ 480.1916, found 480.1917. ^a^Overlapping signals of P(O)(O**CH_2_**CH_3_)_2_ groups. ^b^Overlapping signals of P(O)(OCH_2_**CH_3_**)_2_ groups.
*Tetraethyl 1-(N-benzyloxycarbonylamino)pentylene-1,1-bisphosphonate* (**5i**). Colorless crystals; 90% yield (444 mg), mp 56.0 to 57.2 °C. ^1^H-NMR (400 MHz, CDCl_3_) δ 7.37–7.29 (m, 5H), 5.47 (br t, *J =* 8.5 Hz, 1H), 5.08 (s, 2H), 4.26–4.14 (m, 8H)^a^, 2.38–2.26 (m, 2H), 1.57–1.49 (m, 2H), 1.37–1.25 (m, 2H) and 1.33 (t, *J* = 7.0 Hz, 6H) and 1.32 (t, *J* = 7.0 Hz, 6H)^b^, 0.90 (t, *J* = 7.3 Hz, 3H). ^13^C-NMR (100 MHz, CDCl_3_) δ 154.3 (t, *J =* 7.3 Hz), 136.4, 128.4, 128.0, 66.7, 63.59 (d, *J =* 3.5 Hz) and 63.55 (d, *J =* 3.5 Hz)^b^, 63.38 (d, *J =* 3.4 Hz) and 63.34 (d, *J =* 3.5 Hz)^b^, 60.0 (t, *J =* 144.4 Hz), 30.4 (t, *J =* 3.0 Hz), 26.2 (t, *J =* 6.0 Hz), 23.0, 16.39 (d, *J =* 2.7 Hz) and 16.36 (d, *J =* 2.6 Hz) and 16.33 (d, *J =* 2.6 Hz) and 16.31 (d, *J =* 2.7 Hz)^a^, 13.9. ^31^P-NMR (162 MHz, CDCl_3_): 20.1. IR (ATR) 3224, 1736, 1498, 1233, 1019, 953, 772. HRMS (ESI) *m/z*: calcd for C_21_H_37_NO_8_NaP_2_ [M + Na]^+^ 516.1892, found 516.1889. ^a^Overlapping signals of P(O)(O**CH_2_**CH_3_)_2_ groups. ^b^Overlapping signals of C_α_CH_2_CH_2_**CH_2_**CH_3_ and P(O)(OCH_2_**CH_3_**)_2_ groups. ^c^Overlapping signals of P(O)(OCH_2_**CH_3_**)_2_ groups.
*Tetraethyl 1-(N-benzyloxycarbonylamino)-3-methylbutylene-1,1-bisphosphonate* (**5j**). Colorless crystals; 74% yield (367 mg), mp 54.9 to 55.5 °C. ^1^H-NMR (400 MHz, CDCl_3_) δ 7.35–7.30 (m, 5H), 5.55 (br t, *J* = 12.0 Hz, 1H), 5.09 (s, 2H), 4.28–4.16 (m, 8H)^a^, 2.19–2.11 (m, 3H), 1.33 (t, *J* = 7.1 Hz, 12H), 0.95 (d, *J* = 6.2 Hz, 6H). ^13^C-NMR (100 MHz, CDCl_3_): 154.4 (t, *J =* 8.0 Hz), 136.4, 128.4, 128.14, 128.06, 66.9, 63.56 (d, *J* = 3.5 Hz) and 63.52 (d, *J* = 3.5 Hz)^a^, 63.34 (d, *J* = 3.5 Hz) and 63.30 (d, *J* = 3.5 Hz)^a^, 60.7 (t, *J =* 142.9 Hz), 38.6 (t, *J =* 2.2 Hz), 25.2 (t, *J =* 7.8 Hz), 24.2, 16.44–16.26 (m)^b^. ^31^P-NMR (162 MHz, CDCl_3_): 20.4. IR (ATR) 3231, 1716, 1528, 1250, 1026, 967, 749. HRMS (ESI) *m/z*: calcd for C_21_H_37_NO_8_NaP_2_ [M + Na]^+^ 516.1892, found 516.1891. ^a^Overlapping signals of P(O)(O**CH_2_**CH_3_)_2_ groups. ^b^Overlapping signals of P(O)(OCH_2_**CH_3_**)_2_ groups.
*Tetraethyl 1-(N-acetylamino)-3-methylbutylene-1,1-bisphosphonate* (**5k**). Colorless crystals; 69% yield (278 mg), mp 108.8 to 110.3 °C. ^1^H-NMR (400 MHz, CDCl_3_): δ 6.25 (br t, *J =* 13.0 Hz, 1H), 4.28–4.17 (m, 8H)^a^, 2.18–2.07 (m, 3H), 2.02 (s, 3H), 1.349 (t, *J =* 7.0 Hz, 6H) and 1.345 (t, *J =* 7.0 Hz, 6H)^b^, 0.96 (d, *J =* 6.3 Hz, 6H). ^13^C-NMR (100 MHz, CDCl_3_): δ 169.2 (t, *J =* 6.9 Hz), 63.6 (d, *J =* 7.3 Hz), 63.1 (d, *J =* 7.2 Hz), 60.9 (t, *J =* 143.2 Hz), 38.4 (t, *J =* 2.6 Hz), 25.4 (t, *J =* 8.0 Hz), 24.2, 23.9, 16.4 (d, *J =* 6.0 Hz) and 16.3 (d, *J =* 6.3 Hz)^b^. ^31^P-NMR (162 MHz, CDCl_3_): 20.9. IR (ATR) 3441, 1682, 1541, 1234, 1023, 971. HRMS (ESI) *m/z*: calcd for C_15_H_34_NO_7_P_2_ [M + H]^+^ 402.1811, found 402.1811. ^a^Overlapping signals of P(O)(O**CH_2_**CH_3_)_2_ groups. ^b^Overlapping signals of P(O)(OCH_2_**CH_3_**)_2_ groups.
*Tetraethyl 1-(N-benzyloxycarbonylamino)-2-methoxyethylene-1,1-bisphosphonate* (**5l**). Colorless oil; 52% yield (248 mg), ^1^H-NMR (400 MHz, CDCl_3_) δ 7.36–7.28 (m, 5H), 5.56 (br t, *J =* 8.0 Hz, 1H), 5.09 (s, 2H), 4.26–4.12 (m, 10H)^a^, 3.39 (s, 3H), 1.32 (t, *J =* 7.0 Hz, 12H). ^13^C-NMR (100 MHz, CDCl_3_): δ 154.4 (t, *J =* 6.2 Hz), 136.3, 128.4, 128.1, 128.0, 70.1, 66.9, 63.59 (d, *J =* 3.5 Hz) and 63.55 (d, *J =* 3.6 Hz) and 63.52 (d, *J =* 3.6 Hz)^b^, 60.5 (t, *J =* 142.7 Hz), 59.1, 16.4–16.3 (m)^c^. ^31^P-NMR (162 MHz, CDCl_3_): 18.0. IR (ATR) 1741, 1498, 1254, 1023, 973, 733. HRMS (ESI) *m/z*: calcd for C_19_H_33_NO_9_NaP_2_ [M + Na]^+^ 504.1528, found 504.1526. ^a^Overlapping signals of and C_α_C**H_2_**OCH_3_ and P(O)(O**CH_2_**CH_3_)_2_ groups. ^a^Overlapping signals of P(O)(O**CH_2_**CH_3_)_2_ groups. ^b^Overlapping signals of P(O)(OCH_2_**CH_3_**)_2_ groups.
*Tetraethyl 1-(N-benzyloxycarbonylamino)phenylmethylene-1,1-bisphosphonate* (**5m**). Colorless crystals; 40% yield (204 mg); mp 66.1 to 68.0 °C. ^1^H-NMR (400 MHz, CDCl_3_): δ 7.70–7.66 (m, 2H) and 7.38–7.25 (m, 8H)^a^, 5.93 (br s, 1H), 5.12 (s, 2H), 4.16–3.94 (m, 8H)^b^, 1.21 (t, *J =* 7.0 Hz, 12H). ^13^C-NMR (100 MHz, CDCl_3_) δ 154.4 (br s), 136.3, 132.0, 128.4, 128.1 (t, *J =* 5.0 Hz), 127.6 (t, *J =* 2.6 Hz), 127.5 (t, *J =* 2.3 Hz), 67.1, 64.25 (d, *J =* 3.7 Hz) and 64.21 (d, *J =* 3.6 Hz) and 64.08 (d, *J =* 3.6 Hz) and 64.05 (d, *J =* 3.8 Hz)^b^, 64.15 (t, *J =* 140.4 Hz), 16.26 (d, *J* = 3.1 Hz) and 16.23 (d, *J* = 3.0 Hz)^c^. ^31^P-NMR (162 MHz, CDCl_3_): 17.1. IR (ATR) 3218, 1723, 1530, 1014, 949, 750, 697. HRMS (ESI) *m/z*: calcd for C_23_H_33_NO_8_NaP_2_ [M + Na]^+^ 536.1579, found 536.1570. ^a^Overlapping signals of **Ph**CH_2_O and C_α_**Ph** groups. ^a^Overlapping signals of P(O)(O**CH_2_**CH_3_)_2_ groups. ^c^Overlapping signals of P(O)(OCH_2_**CH_3_**)_2_ groups.
*Tetraethyl 1-(N-benzyloxycarbonylamino)-2-(4-methoxyphenyl)ethylene-1,1-bisphosphonate* (**5n**). Colorless crystals; 74% yield (412 mg), mp 71.7 to 73.5 °C. ^1^H-NMR (400 MHz, CDCl_3_) δ 7.44–7.34 (m, 5H), 7.14–7.09 (m, 2H), 6.71–6.69 (m, 2H), 5.43 (br t, *J =* 12.8 Hz), 5.17 (s, 2H), 4.29–4.17 (m, 4H), 4.13–4.08 (m, 2H), 4.05–3.91 (m, 2H), 3.75 (s, 3H), 3.52 (dd, *J_1_* = 15.3, *J_2_* = 11.8 Hz, 2H) 1.31 (t, *J =* 7.1 Hz, 6H), 1.21 (t, *J =* 7.1 Hz, 6H). ^13^C-NMR (100 MHz, CDCl_3_) δ 158.5, 154.9 (t, *J =* 8.8 Hz), 136.3, 132.1, 128.46, 128.45, 128.2, 127.2 (t, *J =* 8.6 Hz), 113.1, 67.0, 63.9 (d, *J =* 7.4 Hz), 63.0 (d, *J =* 7.6 Hz), 61.1 (t, *J =* 143.0 Hz), 55.2, 34.7, 16.3 (d, *J* = 6.3 Hz), 16.2 (d, *J =* 6.2 Hz). ^31^P-NMR (162 MHz, CDCl_3_): 19.0. IR (ATR) 3251, 1715, 1513, 1247, 1028, 953, 774. HRMS (ESI) *m/z*: calcd for C_25_H_37_NO_9_NaP_2_ [M + Na]^+^ 580.1841, found 580.1839.

## 4. Conclusions

In conclusion, we developed a simple and efficient methodology for the preparation of *N*-protected bisphosphonic analogs of protein and non-protein α-amino acids. The optimization of our procedure, consisting of the reaction of 1-(*N*-acylamino)-1-ethoxyphosphonates **3** with triphenylphosphonium tetrafluoroborate and triethyl phosphite, highlights the one-pot synthesis conducted in mild conditions. In most cases, there is no need to use any catalyst as it is autocatalytic in nature. Relatively easy access to the starting α-ethoxyphosphonates **3**, obtained from ethyl *N*-acylimidates **2**, simple work-up of the reaction mixture and good to excellent yields of the target products **5** are additional advantages of the proposed protocol. The methodology provided constitutes a convenient approach for the synthesis of structurally diverse *N*-protected 1-aminobisphosphonate derivatives **5** and can be considered a new universal strategy for the construction of bisphosphorus organic compounds containing the P-C(N)-P skeleton.

It is worth emphasizing that the one-pot method reported here proceeds through the indirect transformation of α-ethoxyphosphonates, non-reactive in the Michaelis–Arbuzow-type reaction, into the corresponding phosphonium salts **4** of high reactivity and thus susceptibility to further reaction with phosphorus nucleophiles. This is another confirmation of the synthetic potential of phosphonium salts, which are increasingly gaining in importance. As reactive intermediates, they often enable transformations that are difficult to perform with other methods.

## Figures and Tables

**Table 1:** Yields of the Michaelis–Becker-like nucleophilic addition of diethyl phosphite to ethyl *N*-acylimidates ^1^.

Entry	Comp. 3	PG	R^2^	Nu (eq.)	Time [days]	Temperature [°C]	Yield ^2^
1	**3a**	Cbz	Me	1.2	2	rt	94
2	**3b**	Piv	Me	1.2	3	rt	74
3	**3c**	Cbz	H	1.2	2	−10	93
4	**3d**	Cbz	CH_2_Ph	2	4	−20	88
5	**3e**	Ac	CH_2_Ph	1.2	4	rt	53
6	**3f**	Cbz	Et	1.2	3	−5	82
7	**3g**	Cbz	Pr	2	4	−10	68
8	**3h**	Cbz	*i*-Pr	2	7	−40	53
9	**3i**	Cbz	Bu	2	4	−10	54
10	**3j**	Cbz	*i*-Bu	3	7	−40	32
11	**3k**	Ac	*i*-Bu	2	7	−40	65
12	**3l**	Cbz	CH_2_OMe	1.2	4	−10	91
13	**3m**	Cbz	Ph	1.2	3	rt	82
14	**3n**	Cbz	1,4-CH_2_-C_6_H_4_OMe	3	3	−25	70

^1^ Reaction conditions: diethyl *N*-acylimidate **2** (1 eq.), diethyl phosphite (1.2–3.0 eq.), 18-crown-6 (0.12 eq.), K_2_CO_3_ (1.35 eq.), hexane. ^2^ Isolated yield.

**Table 2:** Screening of reaction conditions in the synthesis of bisphosphonate model **5d** from α-ethoxyphosphonate **3d** by the one-pot method.

Entry	Molar Ratios		MePh_3_P^+^ I^−^	Temperature [°C]	Time [h]	Yield [%] ^1^
	3d	Ph_3_P·HBF_4_				
1	1	1.06	+	0–5 for 45 min. then rt	24	52
2	1	1.08	−	0–5 for 45 min. then rt	24	73
3	1	1.05	−	rt	24	77
4	1	1.27	−	rt	24	75
5	1	1.06	−	rt	6	86 ^2^

^1^ Yield estimated from the ^1^H NMR spectrum of the reaction mixture relative to the known mass of the internal standard (diphenyldimethylsilane). ^2^ Isolated yield. ^+^ Denotes the addition of MePh_3_P^+^ I^−^ as a catalyst. ^−^ Denotes that the experiment was performed without the addition of MePh_3_P^+^ I^−^.

## Data Availability

Not applicable.

## Supplementary Materials

The following can be downloaded at: https://www.mdpi.com/article/10.3390/molecules27113571/s1. Supporting information includes ^1^H, ^13^C, ^31^P NMR of all new compounds **3**, **4c** and **5**, as well as a tabulated summary of the characteristic ^13^C NMR data of these compounds.

## Sample Availability

Samples of all described compounds are available from the authors.
