# Supplementary material for: One-Pot and Catalyst-Free Transformation of N-Protected 1-Amino-1-Ethoxyalkylphosphonates into Bisphosphonic Analogs of Protein and Non-Protein α-Amino Acids
Source: Molecules. 2022 Jun 2;27(11):3571. doi: 10.3390/molecules27113571 (PMC9182278; doi:10.3390/molecules27113571)
Supplement: Supplementary file 1 [file molecules-27-03571-s001.zip › molecules-1755804-SI.pdf]

One-pot and catalyst-free transformation of *N*-protected 1-amino-1-ethoxyalkylphosphonates into bisphosphonic analogs of protein and non-protein α-amino acids

Anna Kuźnik [1,2,*], Dominika Kozicka [1,2], Wioleta Hawranek [1], Karolina Socha [1] and Karol Erfurt [3]

[1] *Department of Organic Chemistry, Bioorganic Chemistry and Biotechnology, Silesian University of Technology, B. Krzywoustego 4, 44-100 Gliwice, Poland.*

[2] *Biotechnology Center of Silesian University of Technology, B. Krzywoustego 8, 44-100 Gliwice, Poland.*

[3] *Department of Chemical Organic Technology and Petrochemistry, Silesian University of Technology, B. Krzywoustego 4, 44-100 Gliwice, Poland*

[*] *Corresponding author. e-mail: anna.kuznik@polsl.pl; Tel: +48 32 237 16 13; Fax: +48 32 237 20 94.*

Table of contents

1. ^{1}H, ^{13}C and ^{31}P NMR spectra of all 1-(*N*-acylamino)-1-ethoxyalkylphosphonates **3** (S2-S43);
2. ^{1}H, ^{13}C and ^{31}P NMR spectra of diethyl 1-(*N*-benzyloxycarbonylamino)-1-triphenylphosphoniummethylphosphonate **4c** (S44-S46);
3. ^{1}H, ^{13}C and ^{31}P NMR spectra of all 1-(*N*-acylamino)alkylene-1,1-bisphosphonates **5** (S44-S88).
4. Summary of characteristic ^{13}C NMR data for all 1-(*N*-acylamino)-1-ethoxyalkylphosphonates **3**, diethyl 1-(*N*-benzyloxycarbonylamino)-1-triphenylphosphoniummethylphosphonate **4c** and all 1-(*N*-acylamino)alkylene-1,1-bisphosphonates **5** (S47-S92).

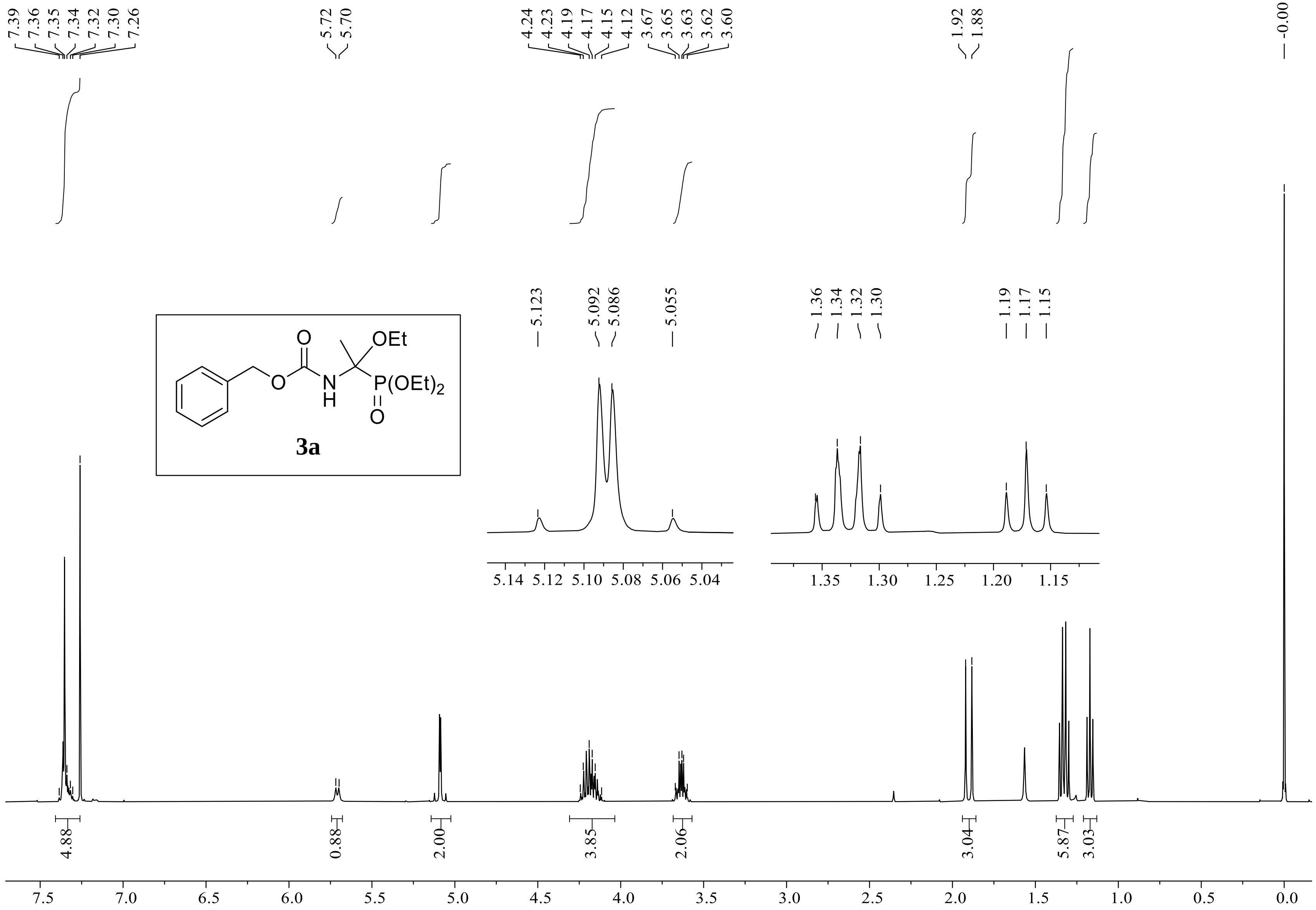

^{1}H NMR spectrum of *diethyl 1-(N-benzyloxycarbonylamino)-1-ethoxyethylphosphonate* (**3a**); 400 MHz/$CDCl_3$/TMS; δ (ppm).

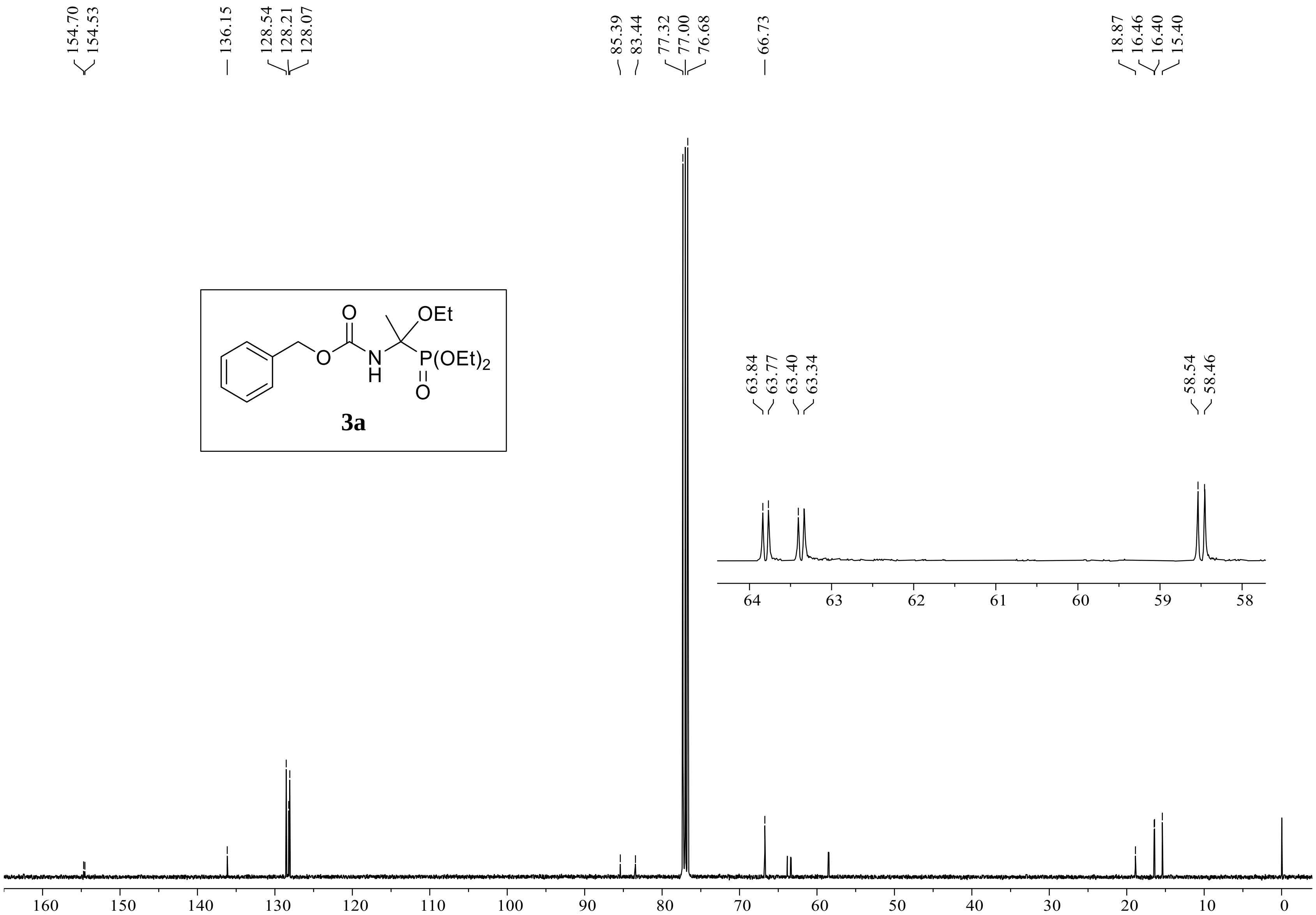

^{13}C NMR spectrum of *diethyl 1-(N-benzyloxycarbonylamino)-1-ethoxyethylphosphonate* (**3a**); 100 MHz/$CDCl_3$/TMS; δ (ppm).

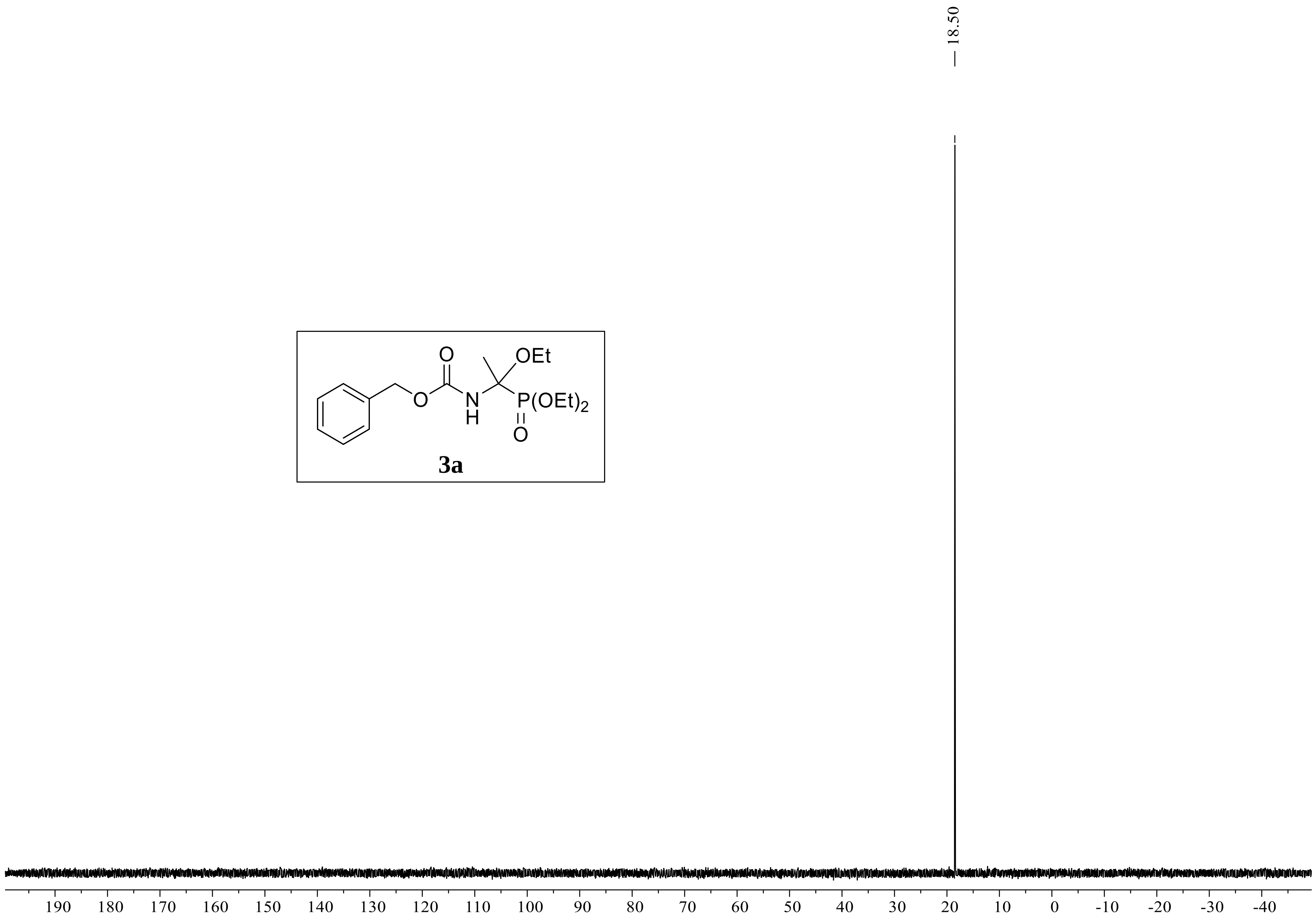

3a

^{31}P NMR spectrum of *diethyl 1-(N-benzyloxycarbonylamino)-1-ethoxyethylphosphonate* (**3a**); 162 MHz/$CDCl_3$/TMS; δ (ppm).

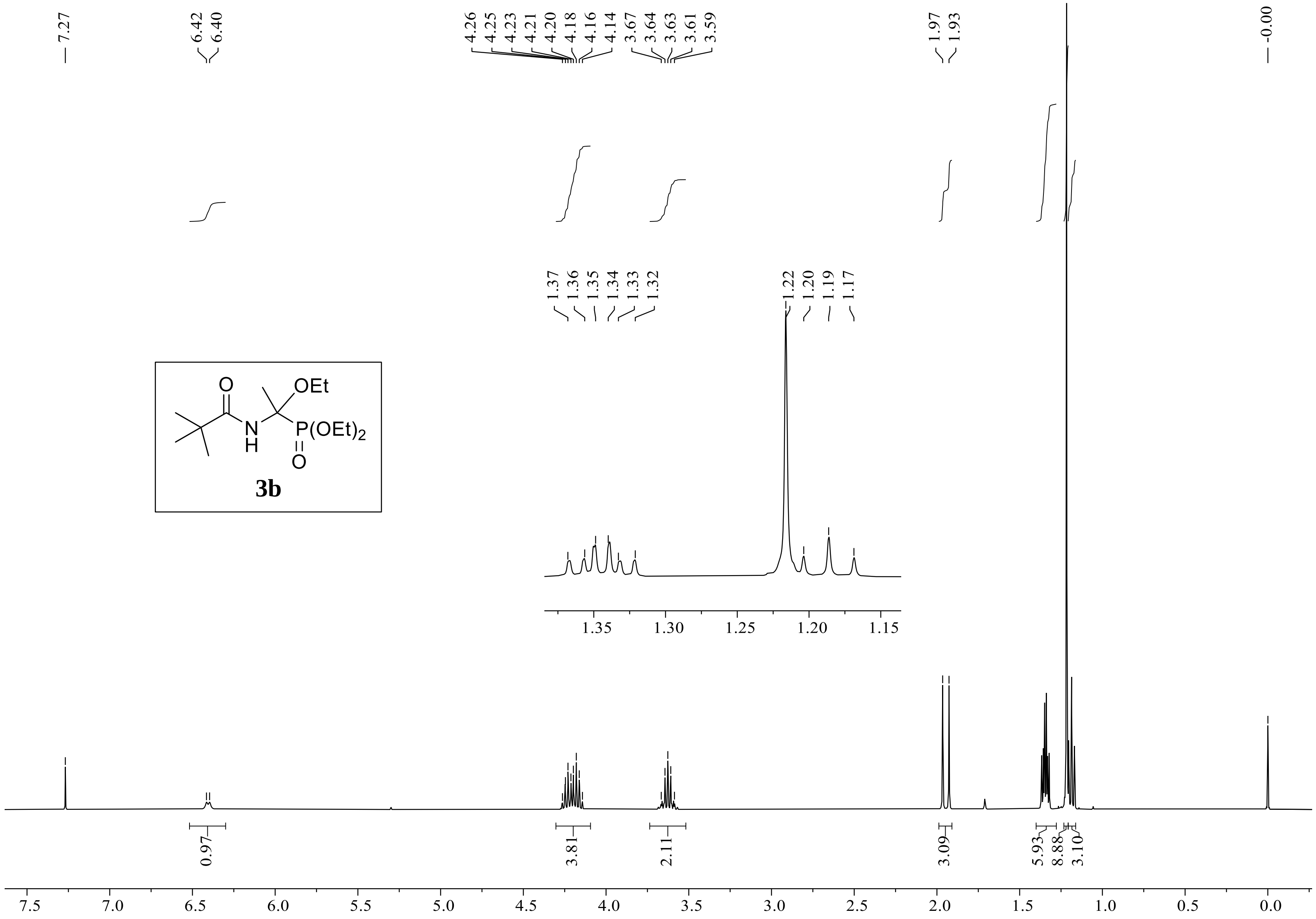

^{1}H NMR spectrum of *diethyl 1-(N-pivaloylamino)-1-ethoxyethylphosphonate* (**3b**); 400 MHz/$CDCl_3$/TMS; δ (ppm).

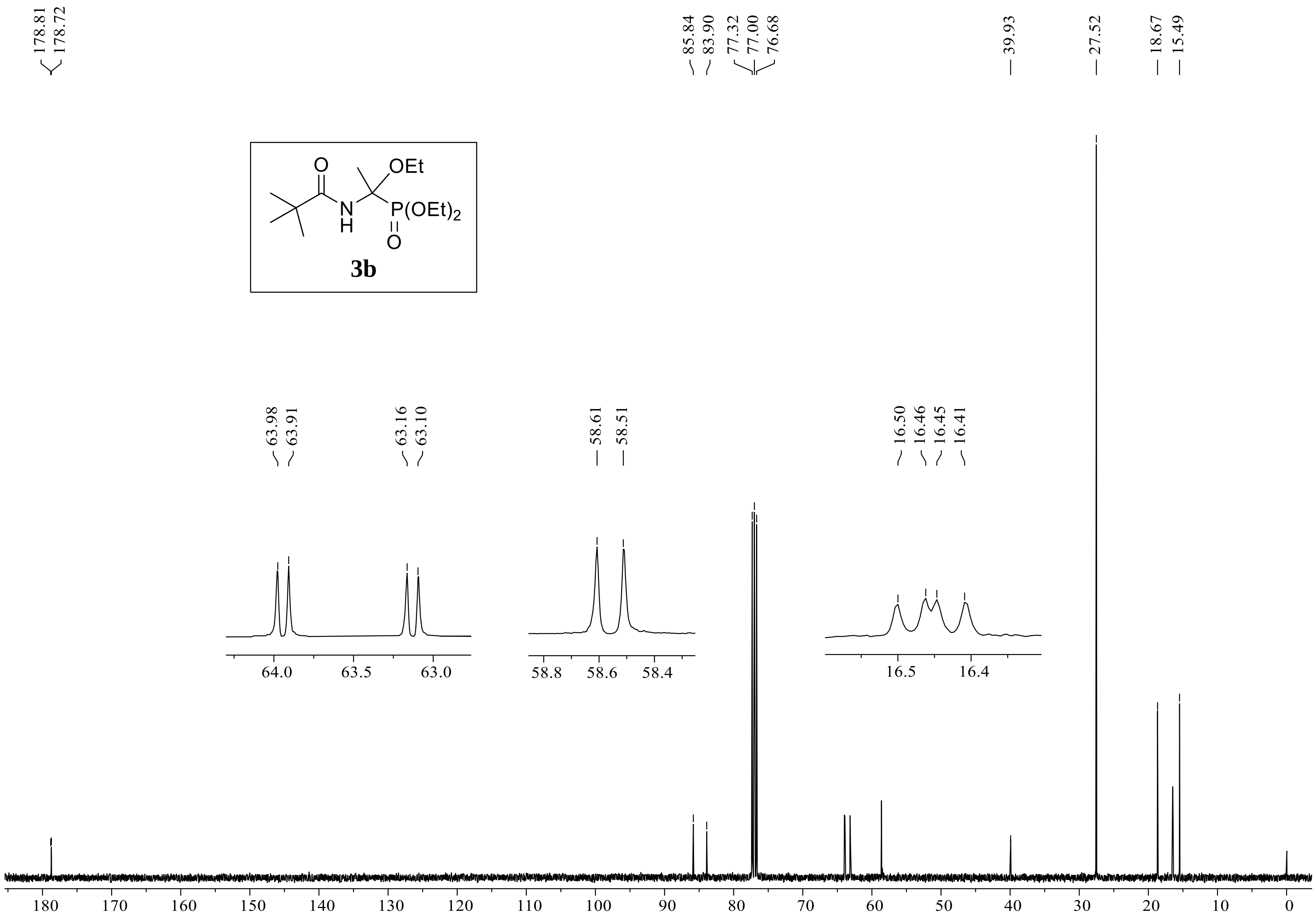

^{13}C NMR spectrum of *diethyl 1-(N-pivaloylamino)-1-ethoxyethylphosphonate* (**3b**); 100 MHz/$CDCl_3$/TMS; δ (ppm).

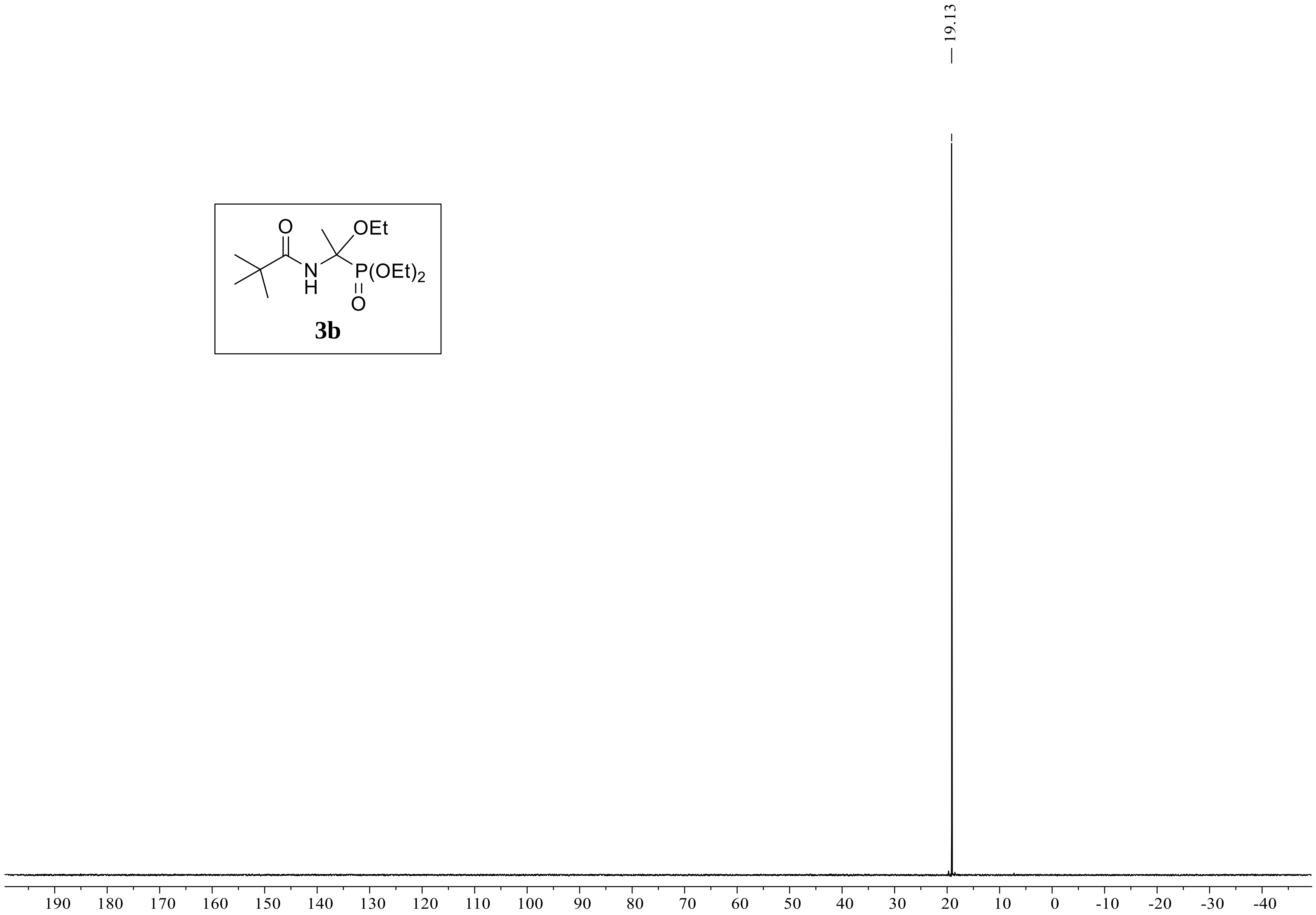

^{31}P NMR spectrum of *diethyl 1-(N-pivaloylamino)-1-ethoxyethylphosphonate* (**3b**); 162 MHz/$CDCl_3$/TMS; δ (ppm).

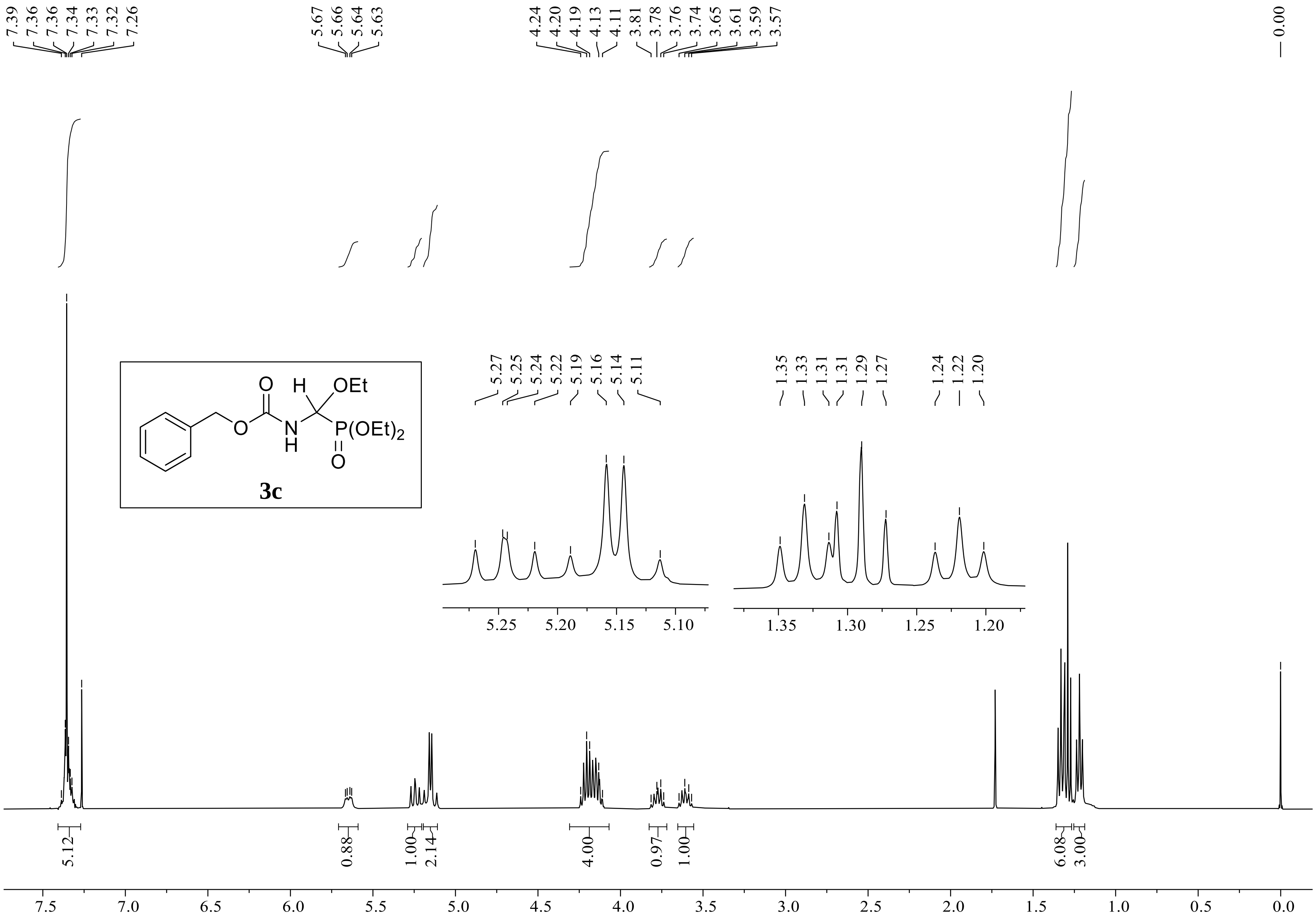

^{1}H NMR spectrum of *diethyl 1-(N-benzyloxycarbonylamino)-1-ethoxymethylphosphonate* (**3c**); 400 MHz/$CDCl_3$/TMS; δ (ppm).

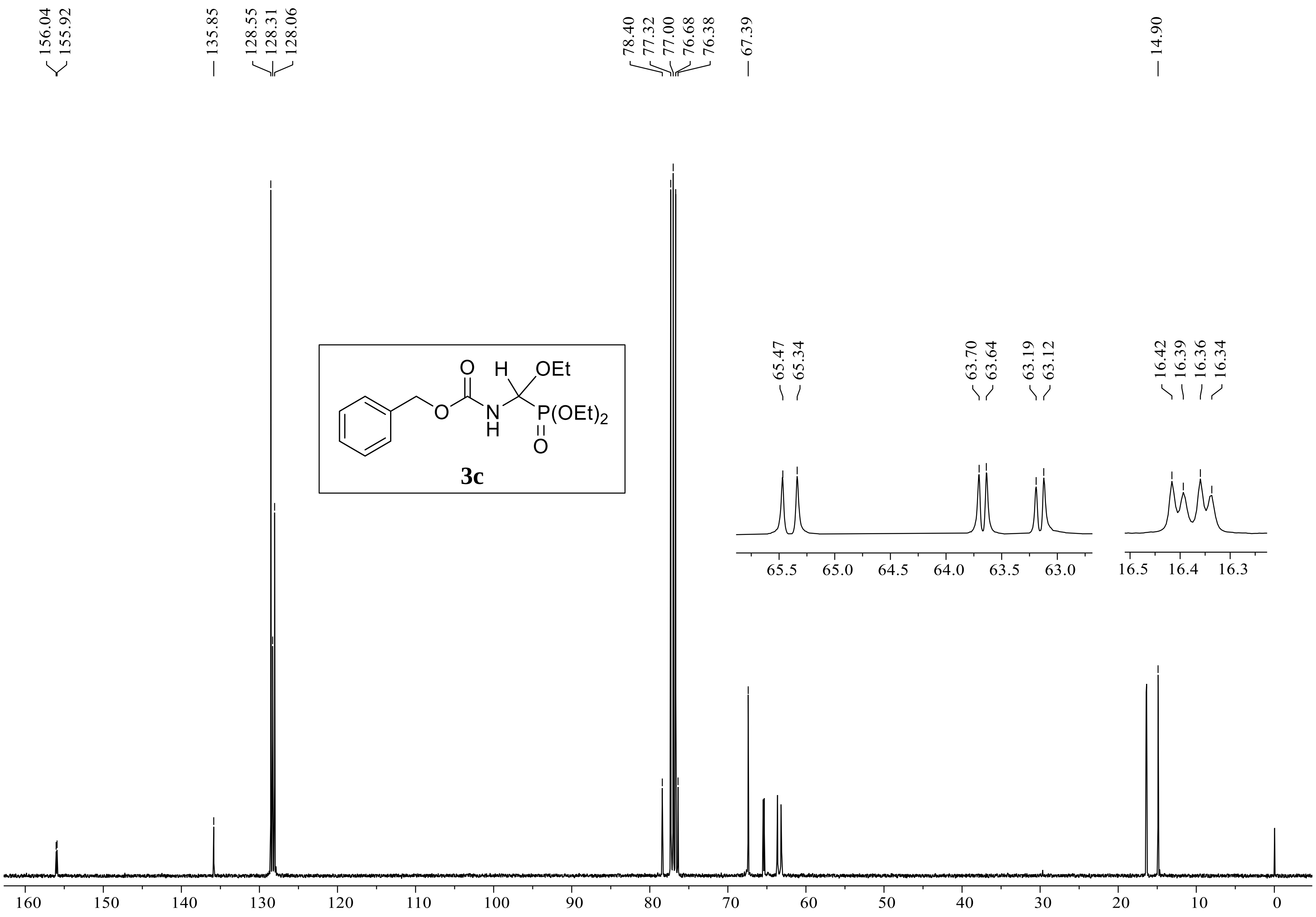

^{13}C NMR spectrum of *diethyl 1-(N-benzyloxycarbonylamino)-1-ethoxymethylphosphonate* (**3c**); 100 MHz/$CDCl_3$/TMS; δ (ppm).

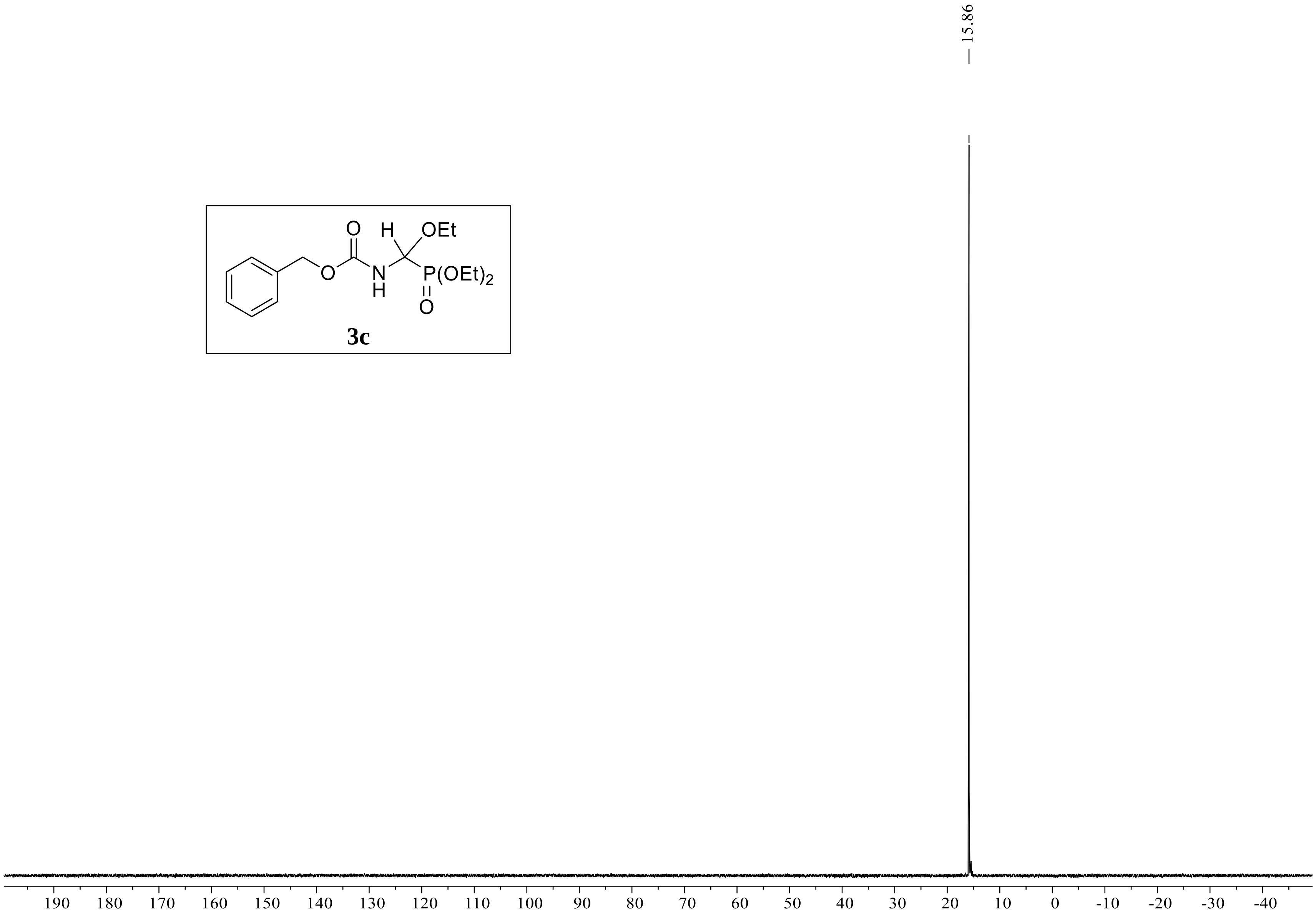

^{31}P NMR spectrum of *diethyl 1-(N-benzyloxycarbonylamino)-1-ethoxymethylphosphonate* (**3c**); 162 MHz/$CDCl_3$/TMS; δ (ppm).

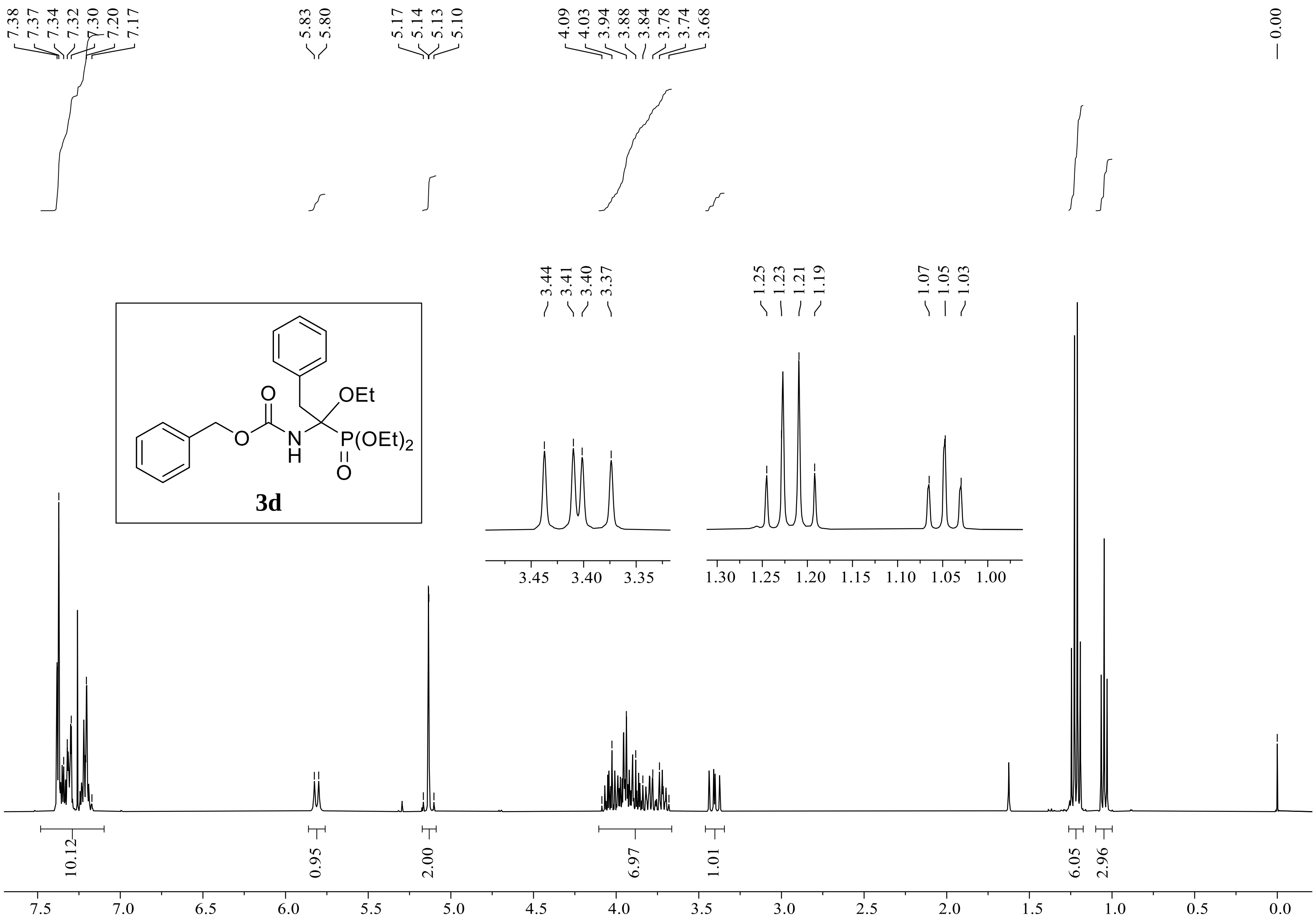

^{1}H NMR spectrum of *diethyl 1-(N-benzyloxycarbonylamino)-1-ethoxy-2-phenylethylphosphonate* (**3d**); 400 MHz/$CDCl_3$/TMS; δ (ppm).

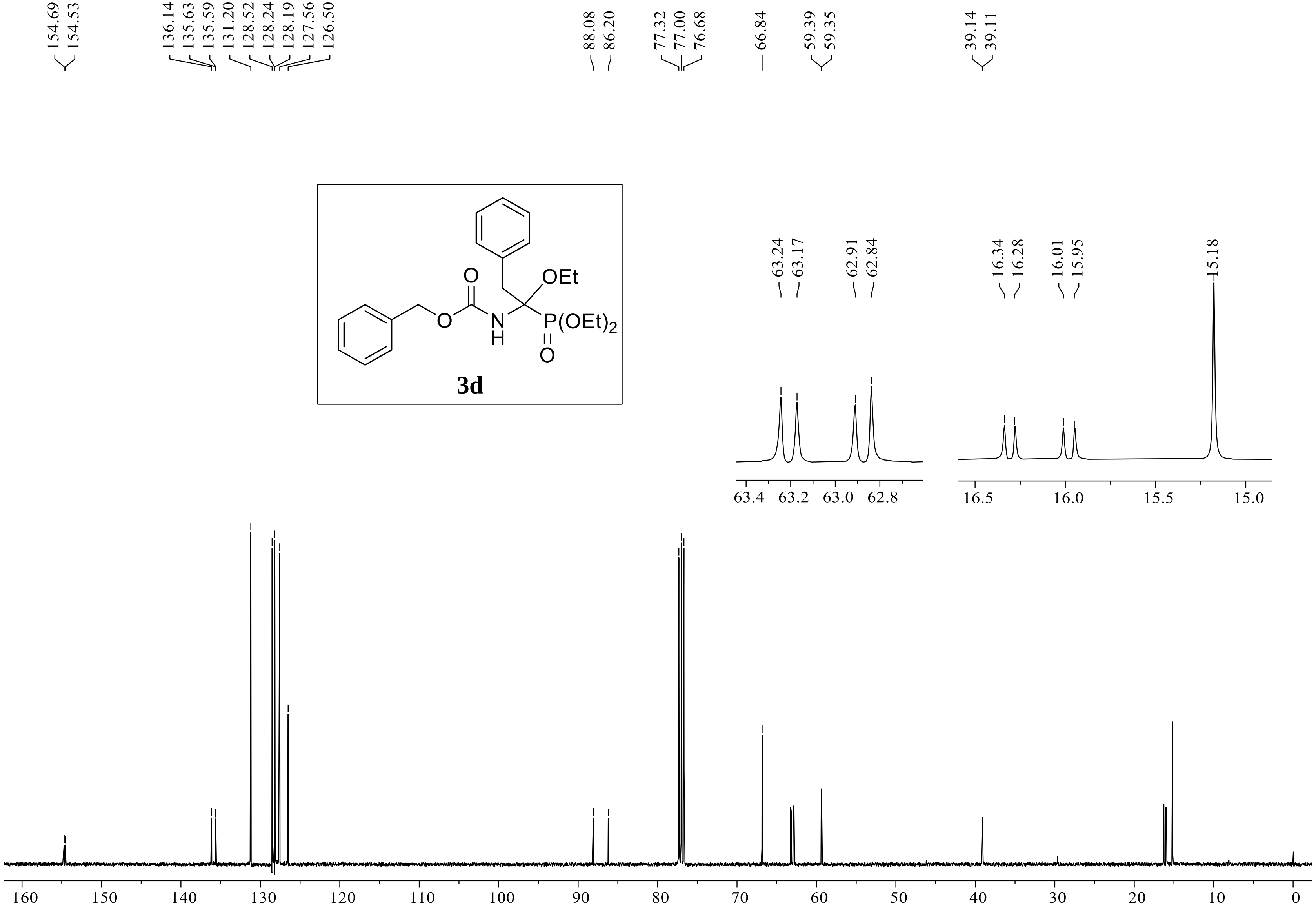

^{13}C NMR spectrum of *diethyl 1-(N-benzyloxycarbonylamino)-1-ethoxy-2-phenylethylphosphonate* (**3d**); 100 MHz/$CDCl_3$/TMS; δ (ppm).

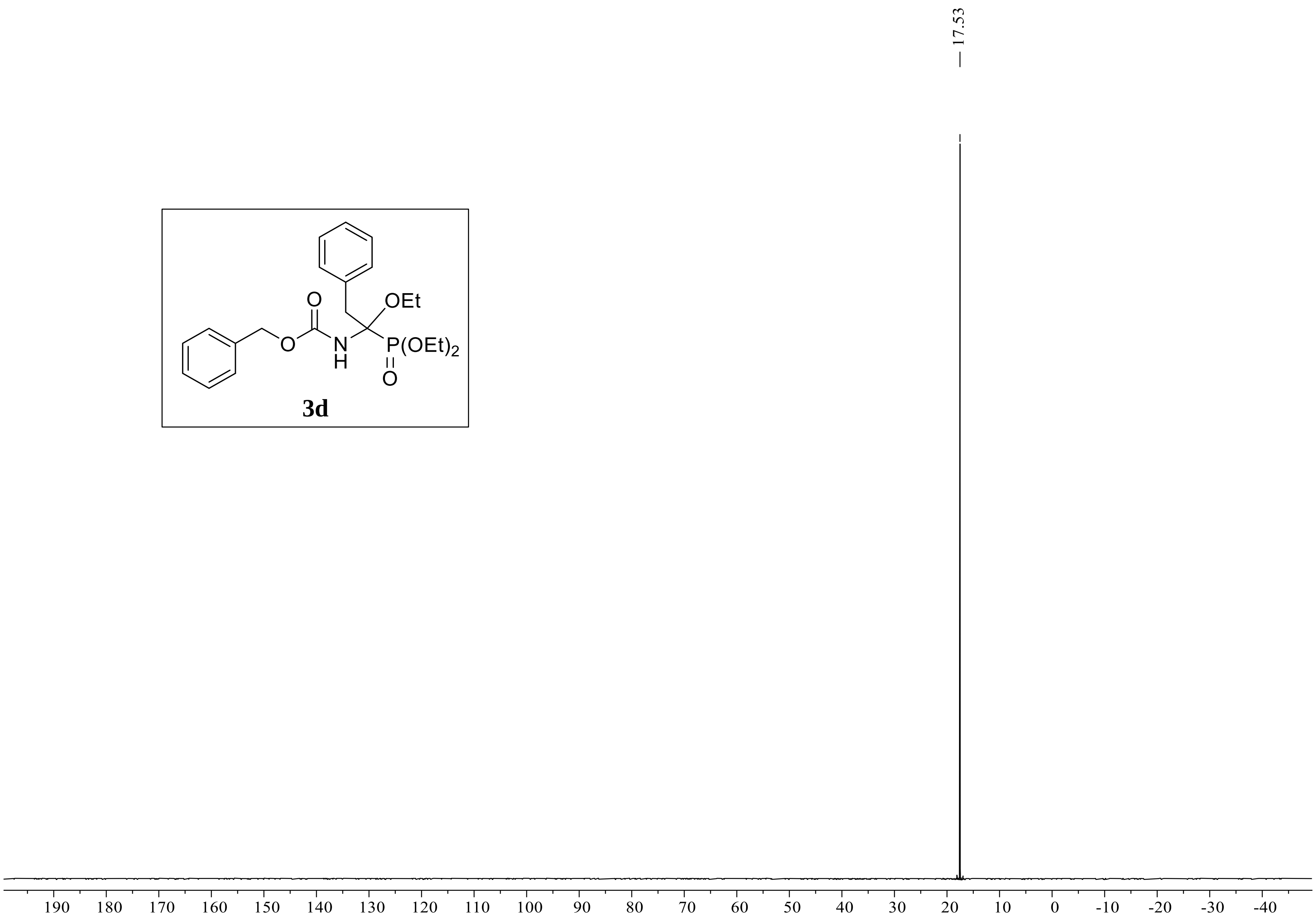

^{31}P NMR spectrum of *diethyl 1-(N-benzyloxycarbonylamino)-1-ethoxy-2-phenylethylphosphonate* (**3d**); 162 MHz/$CDCl_3$/TMS; δ (ppm).

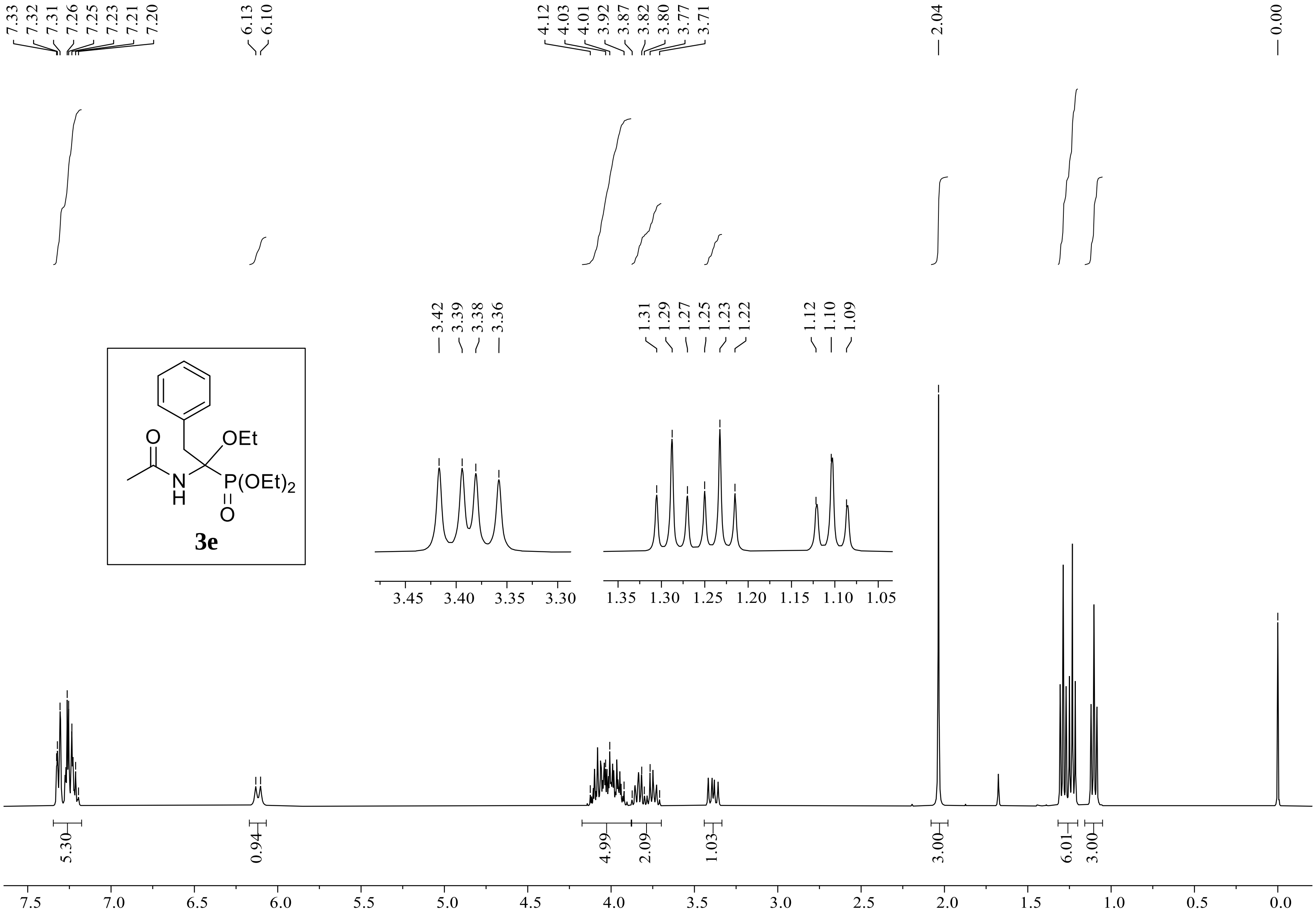

^{1}H NMR spectrum of *diethyl 1-(N-acetylamino)-1-ethoxy-2-phenylethylphosphonate* (**3e**); 400 MHz/$CDCl_3$/TMS; δ (ppm).

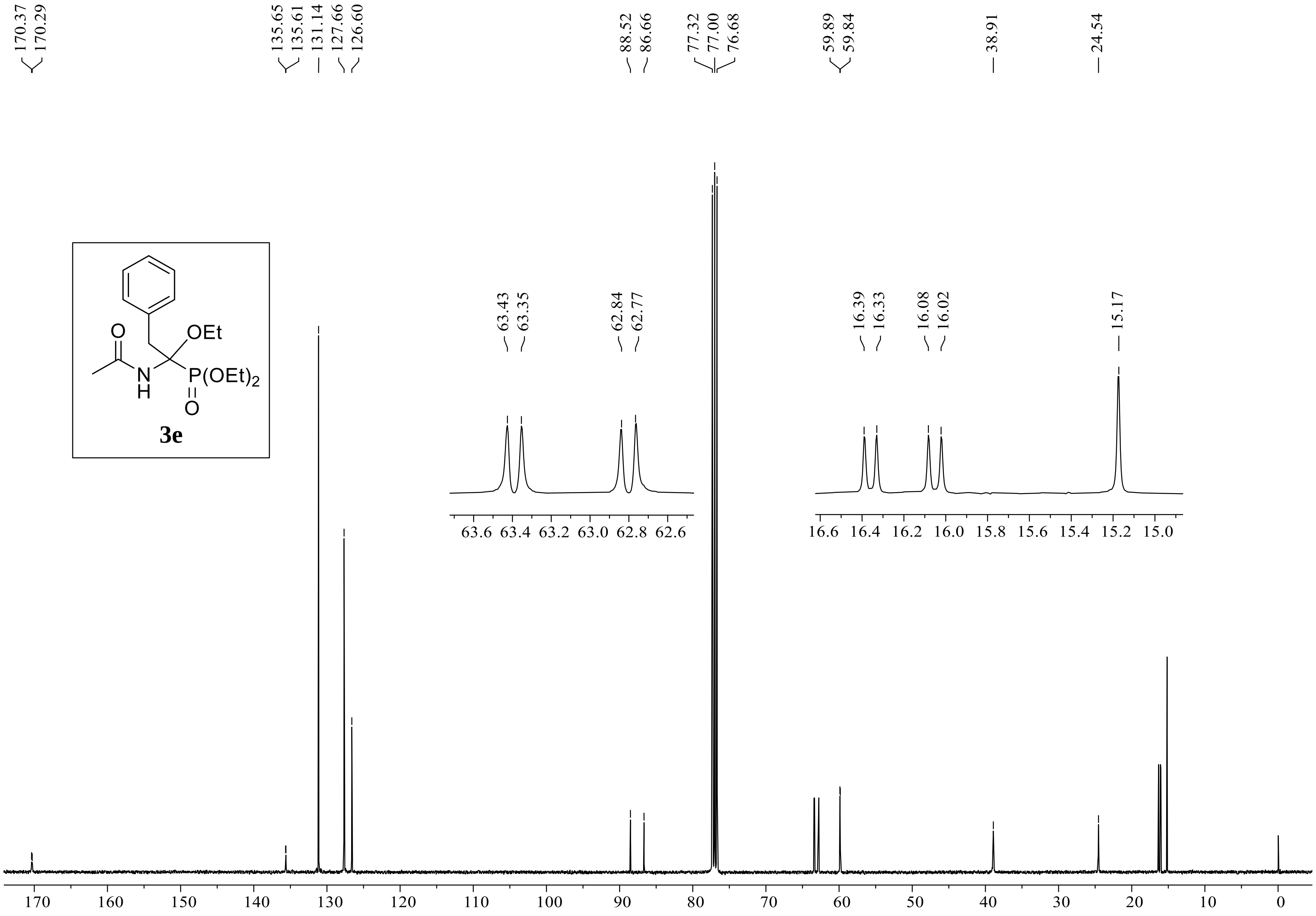

^{13}C NMR spectrum of *diethyl 1-(N-acetylamino)-1-ethoxy-2-phenylethylphosphonate* (**3e**); 100 MHz/$CDCl_3$/TMS; δ (ppm).

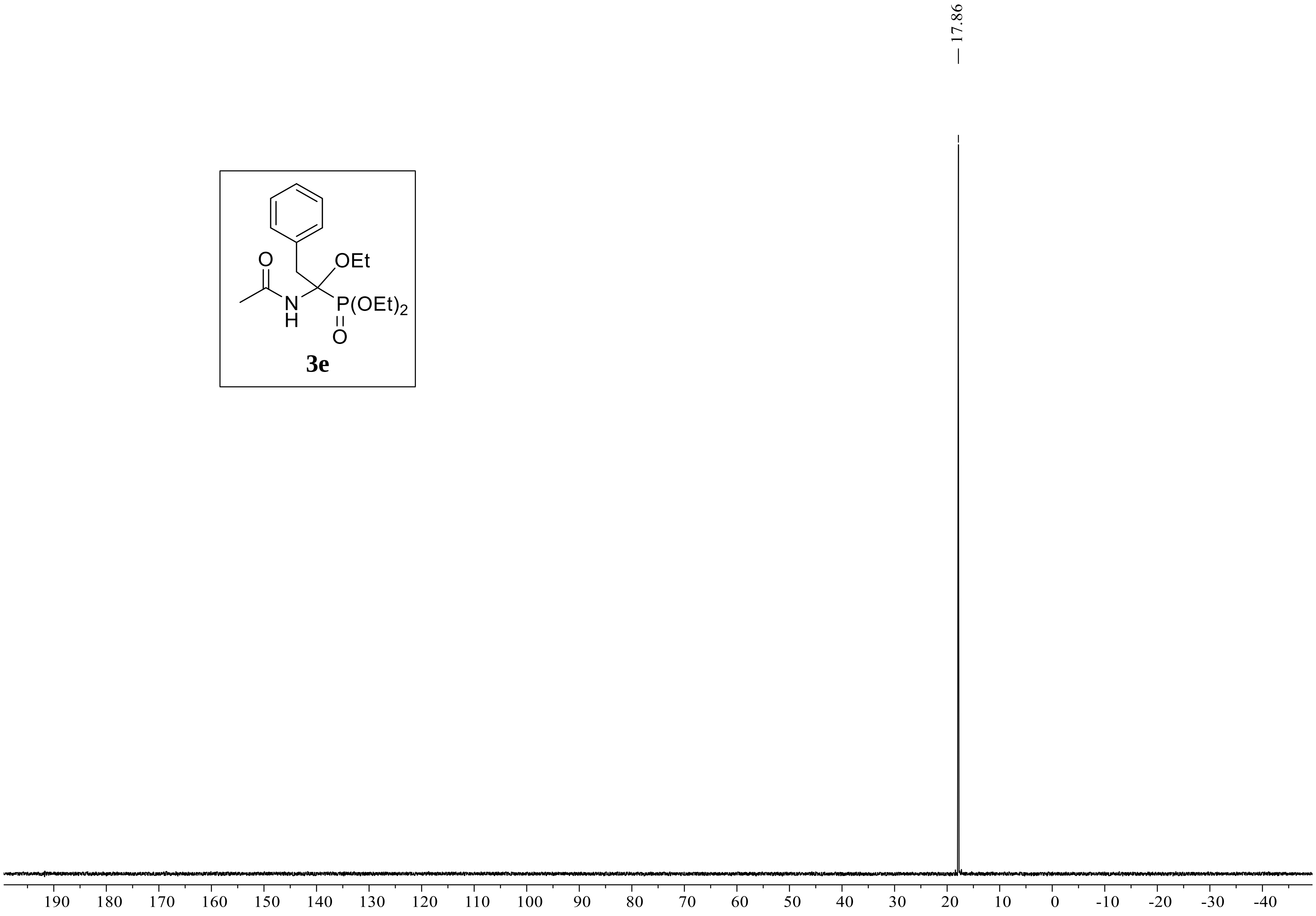

^{31}P NMR spectrum of *diethyl 1-(N-acetylamino)-1-ethoxy-2-phenylethylphosphonate* (**3e**); 162 MHz/$CDCl_3$/TMS; δ (ppm).

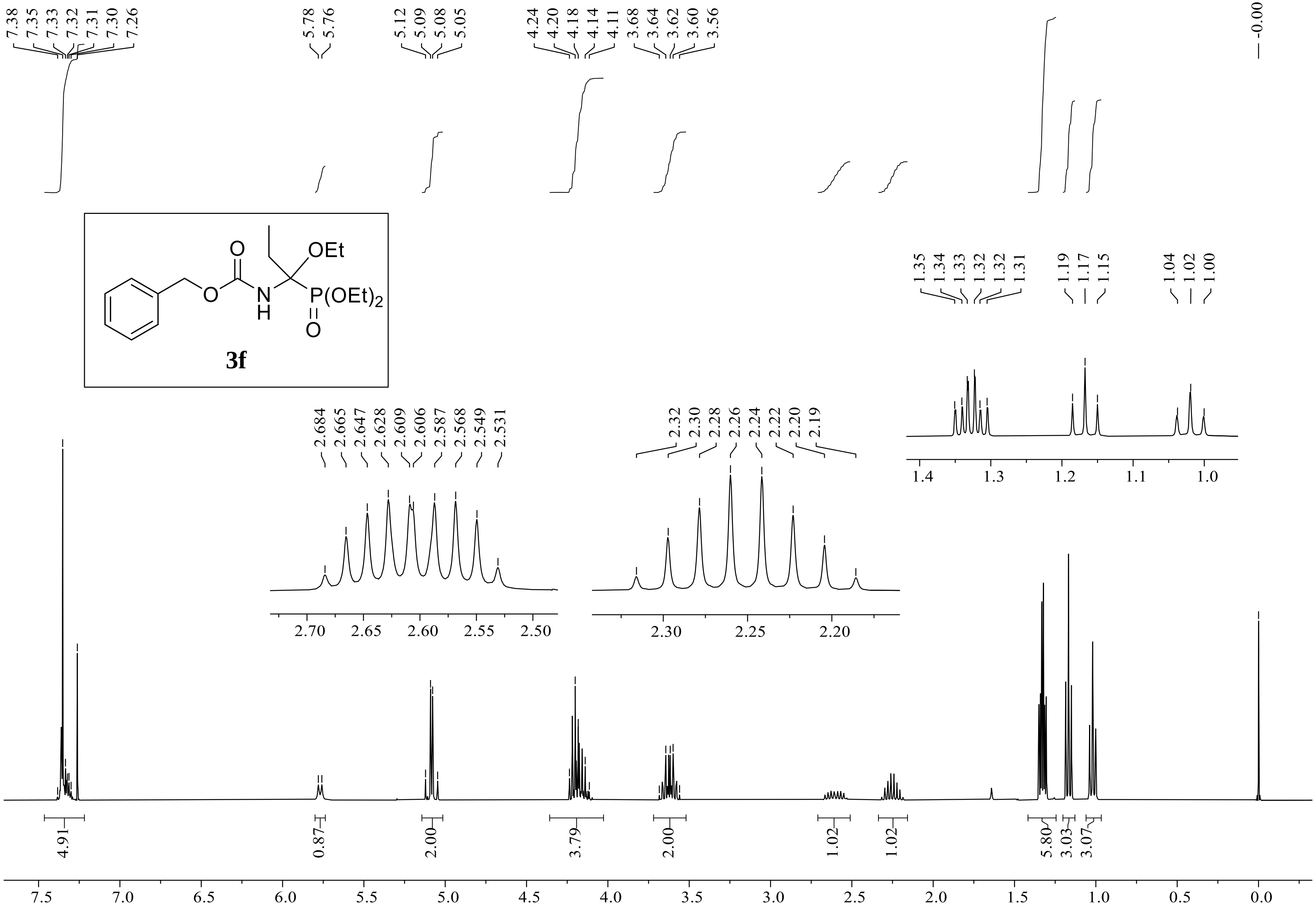

^{1}H NMR spectrum of *diethyl 1-(N-benzyloxycarbonylamino)-1-ethoxypropylphosphonate* (**3f**); 400 MHz/$CDCl_3$/TMS; δ (ppm).

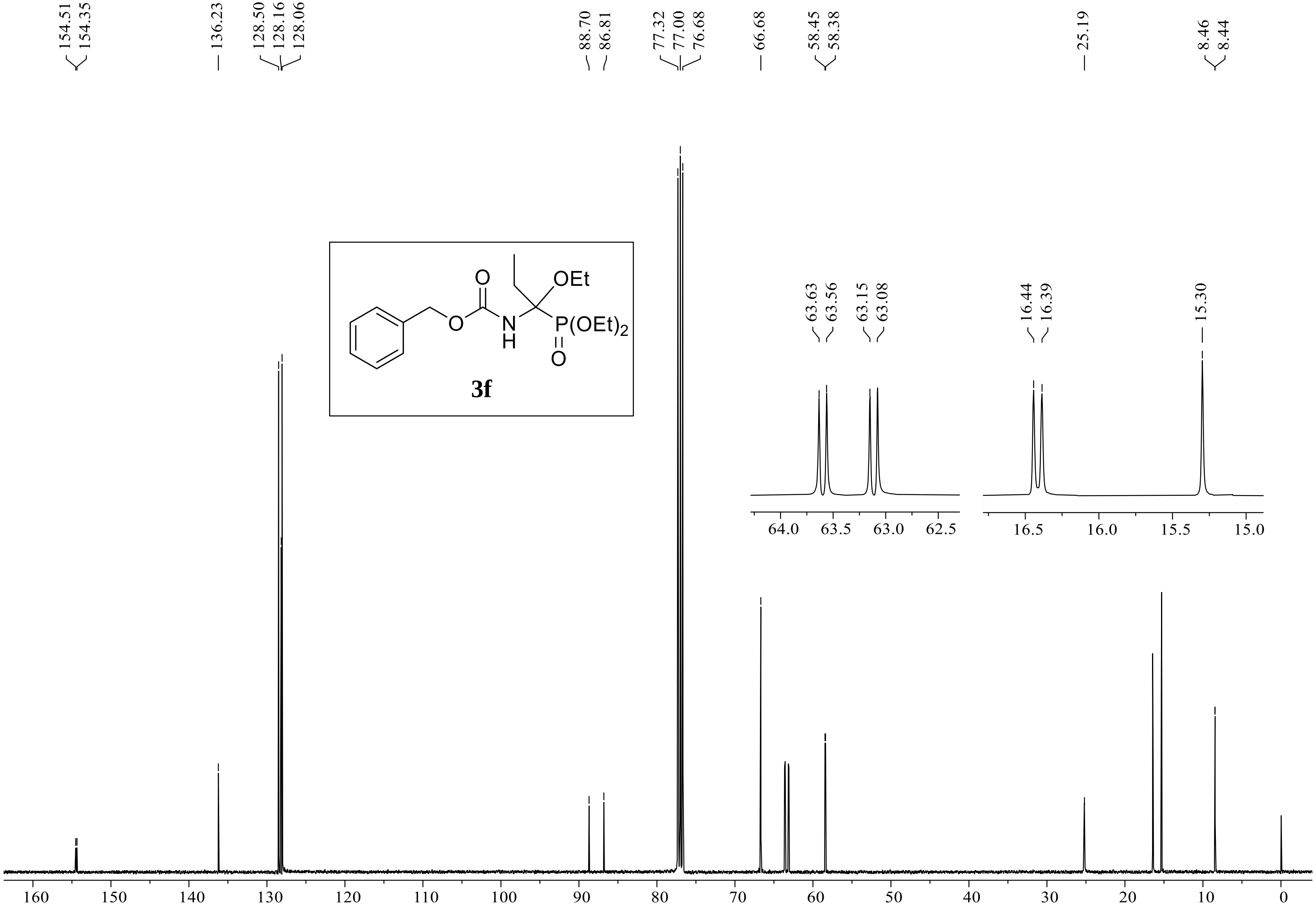

^{13}C NMR spectrum of *diethyl 1-(N-benzyloxycarbonylamino)-1-ethoxypropylphosphonate* (**3f**); 100 MHz/$CDCl_3$/TMS; *δ* (ppm).

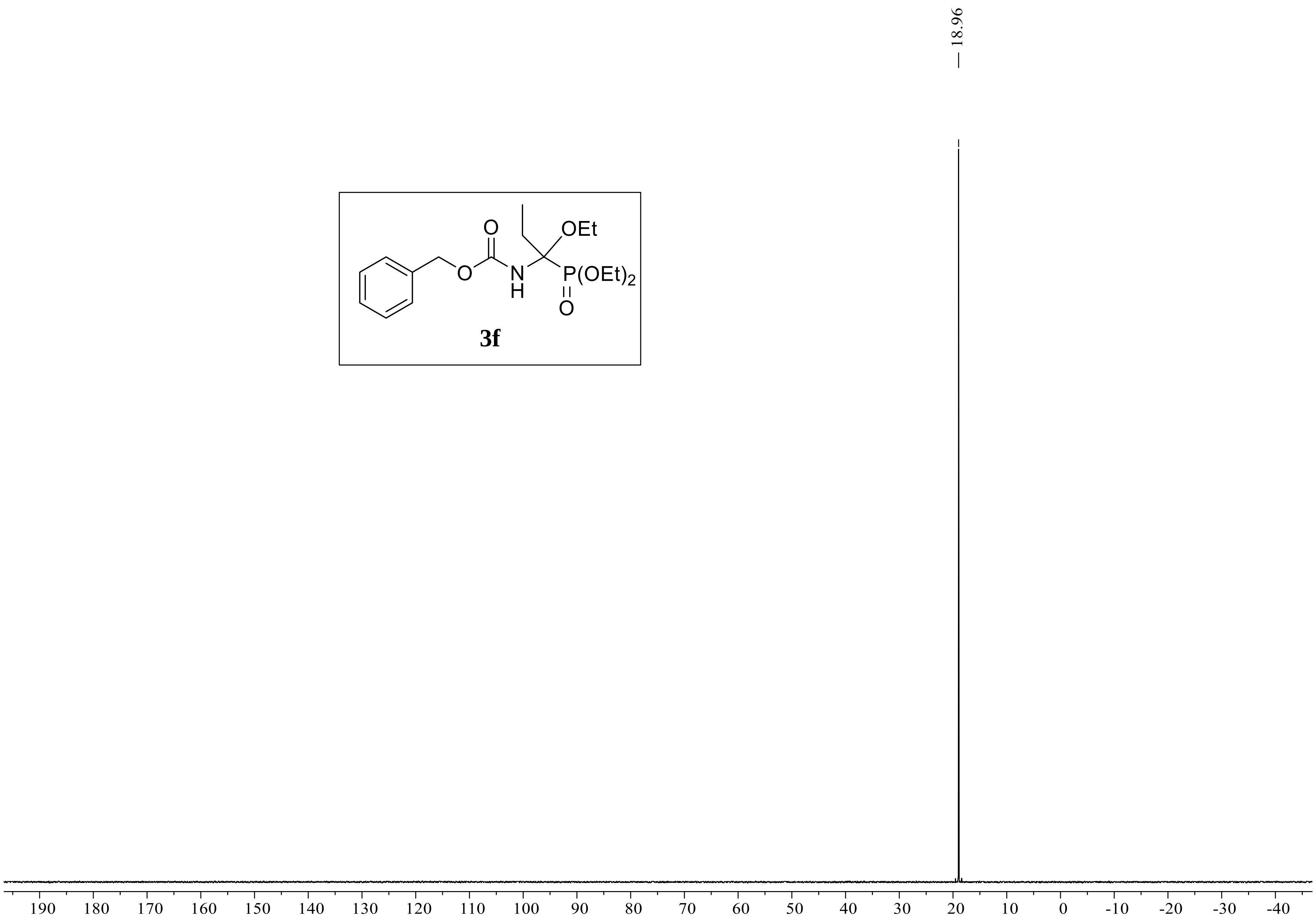

^{31}P NMR spectrum of *diethyl 1-(N-benzyloxycarbonylamino)-1-ethoxypropylphosphonate* (**3f**); 162 MHz/$CDCl_3$/TMS; δ (ppm).

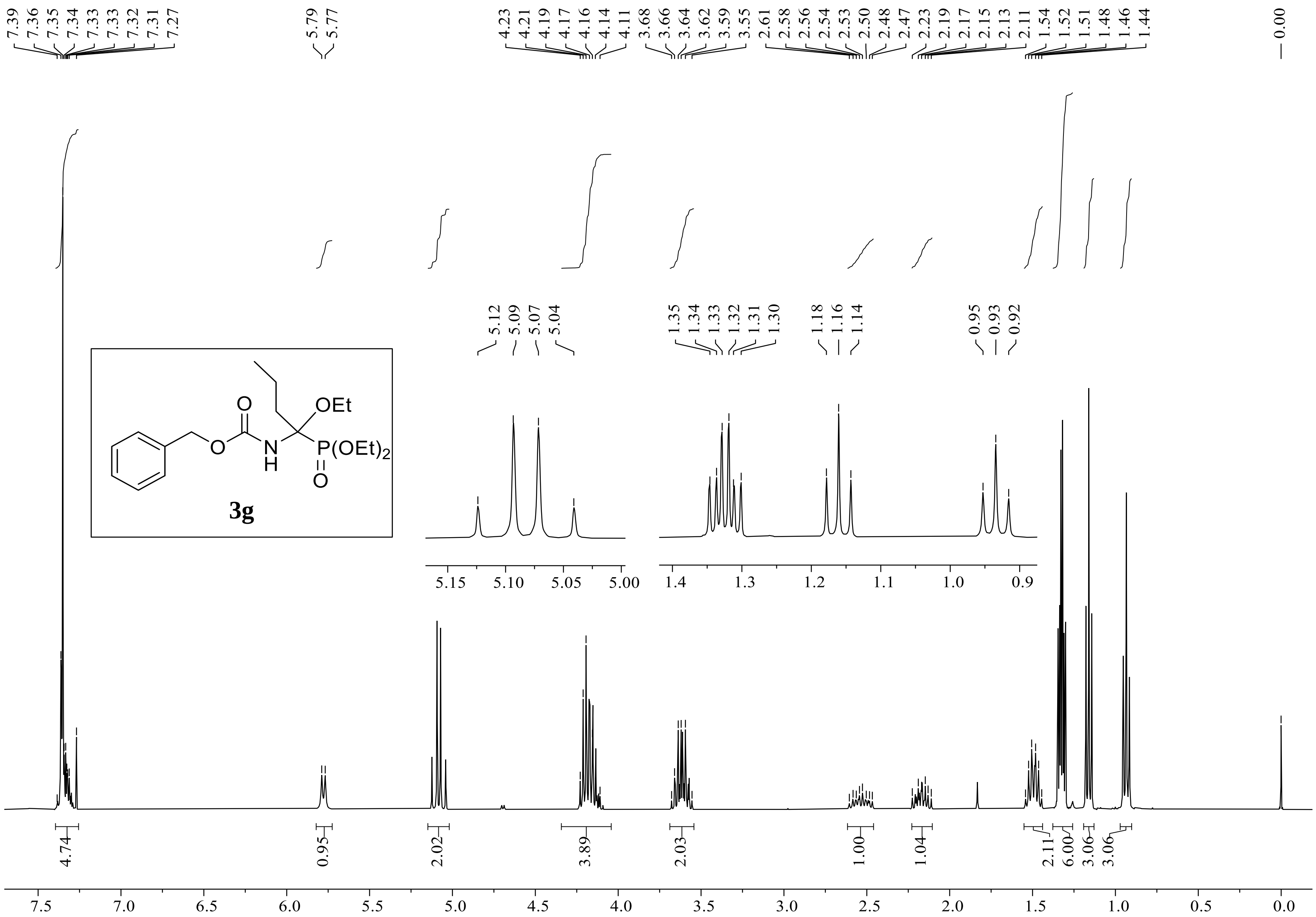

^{1}H NMR spectrum of *diethyl 1-(N-benzyloxycarbonylamino)-1-ethoxybutylphosphonate* (**3g**); 400 MHz/$CDCl_3$/TMS; δ (ppm).

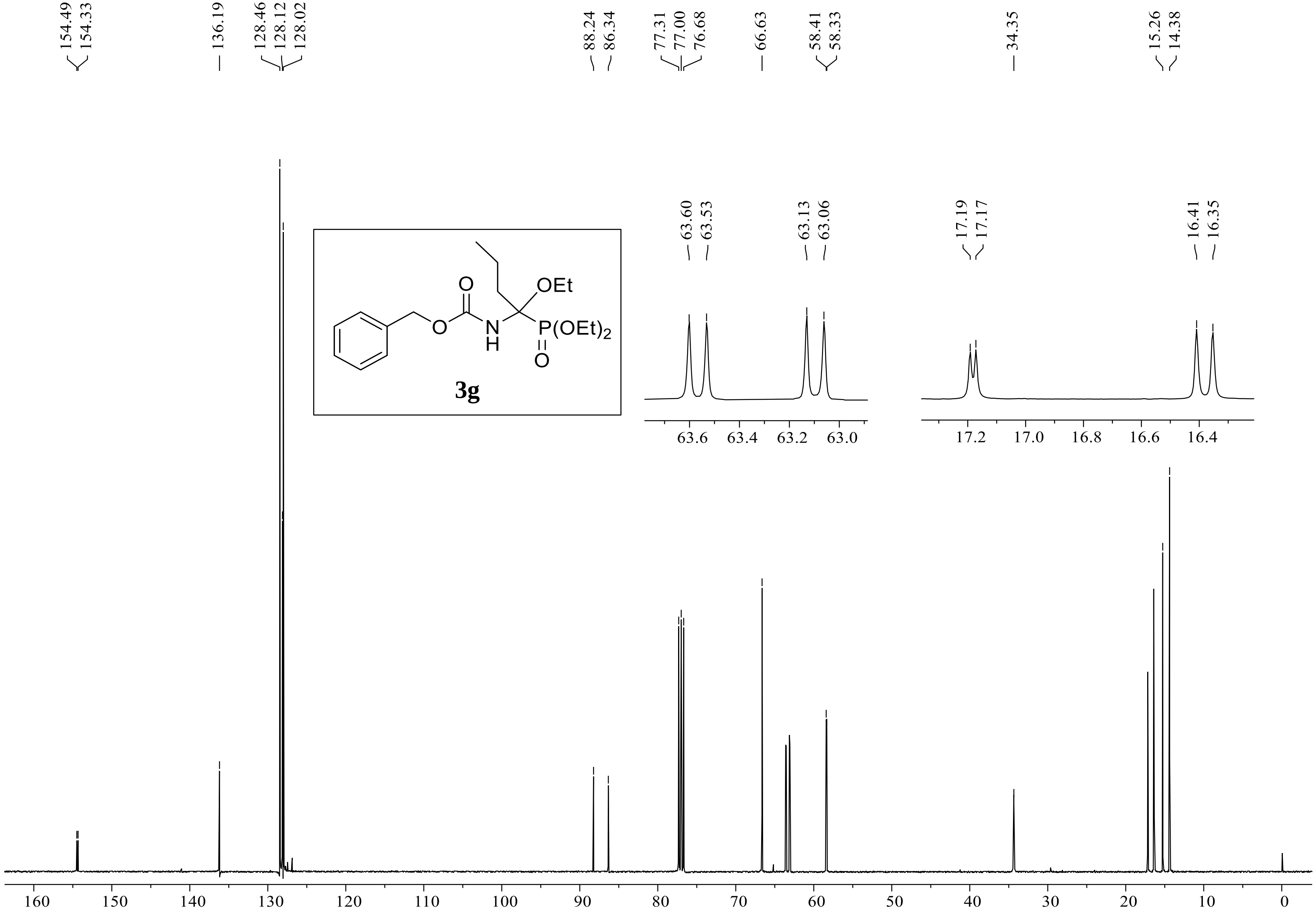

^{13}C NMR spectrum of *diethyl 1-(N-benzyloxycarbonylamino)-1-ethoxybutylphosphonate* (**3g**); 100 MHz/$CDCl_3$/TMS; δ (ppm).

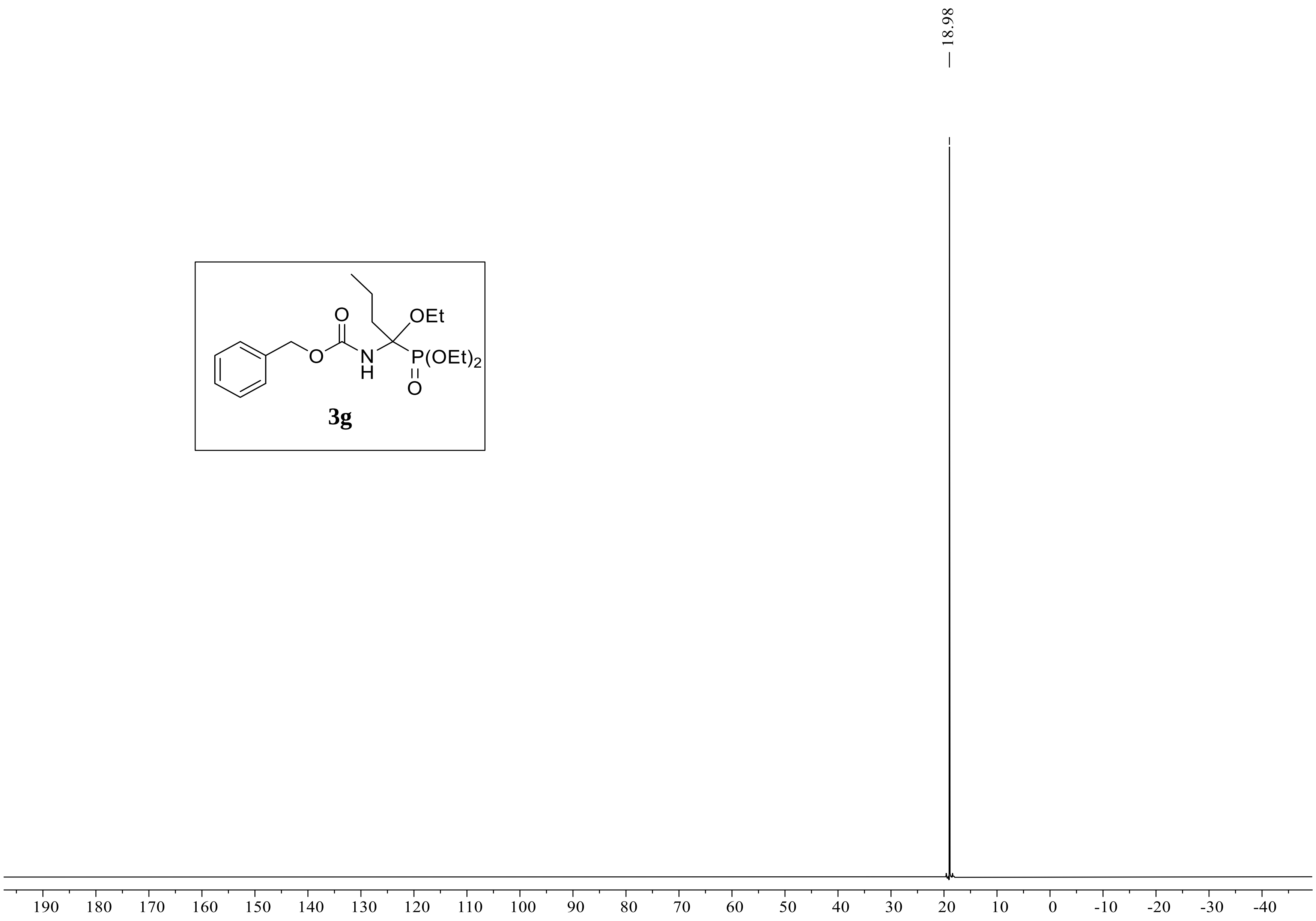

3g

^{31}P NMR spectrum of *diethyl 1-(N-benzyloxycarbonylamino)-1-ethoxybutylphosphonate* (**3g**); 162 MHz/$CDCl_3$/TMS; δ (ppm).

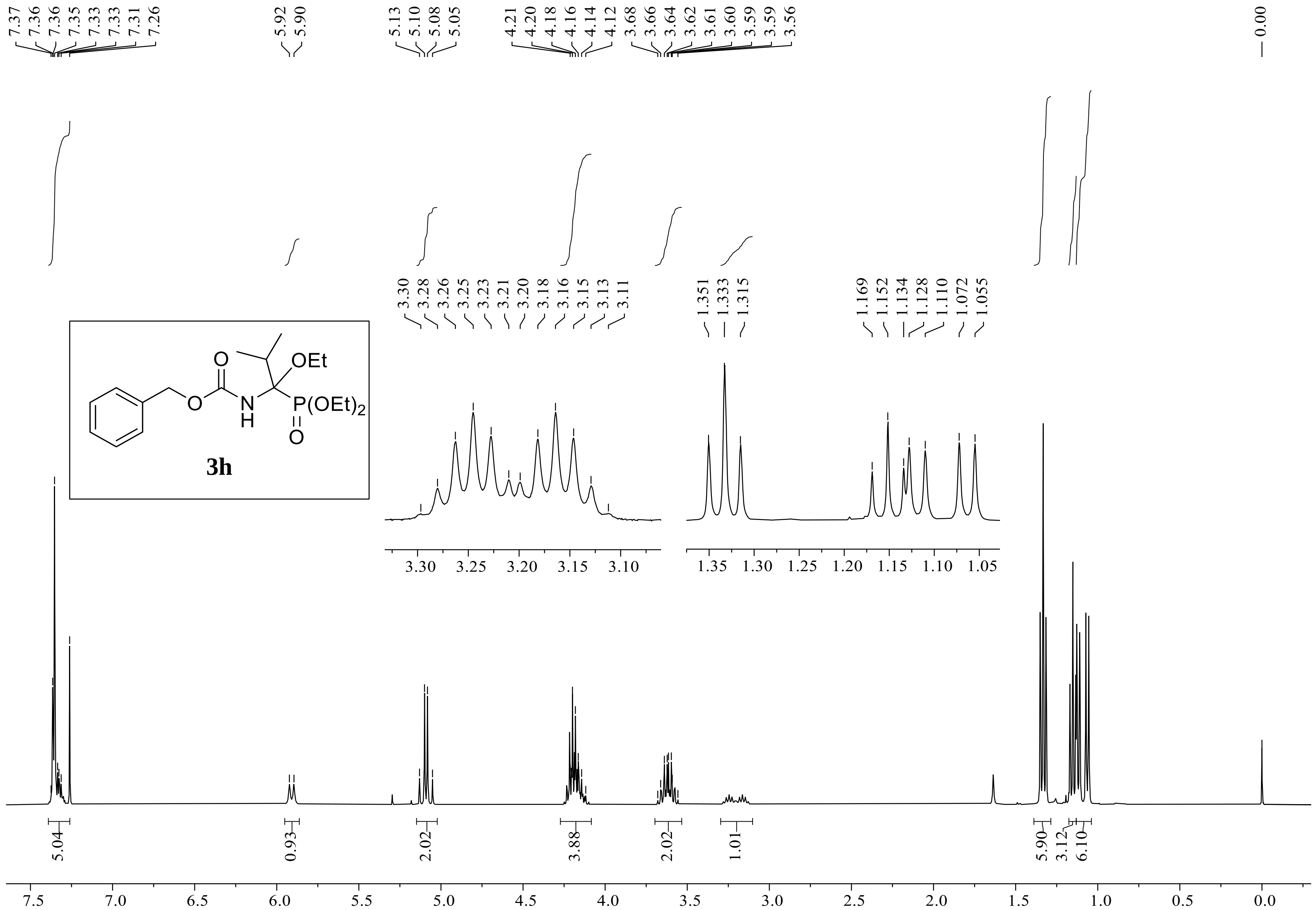

^{1}H NMR spectrum of *diethyl 1-(N-benzyloxycarbonylamino)-1-ethoxy-2-methylpropylphosphonate* (**3h**); 400 MHz/$CDCl_3$/TMS; δ (ppm).

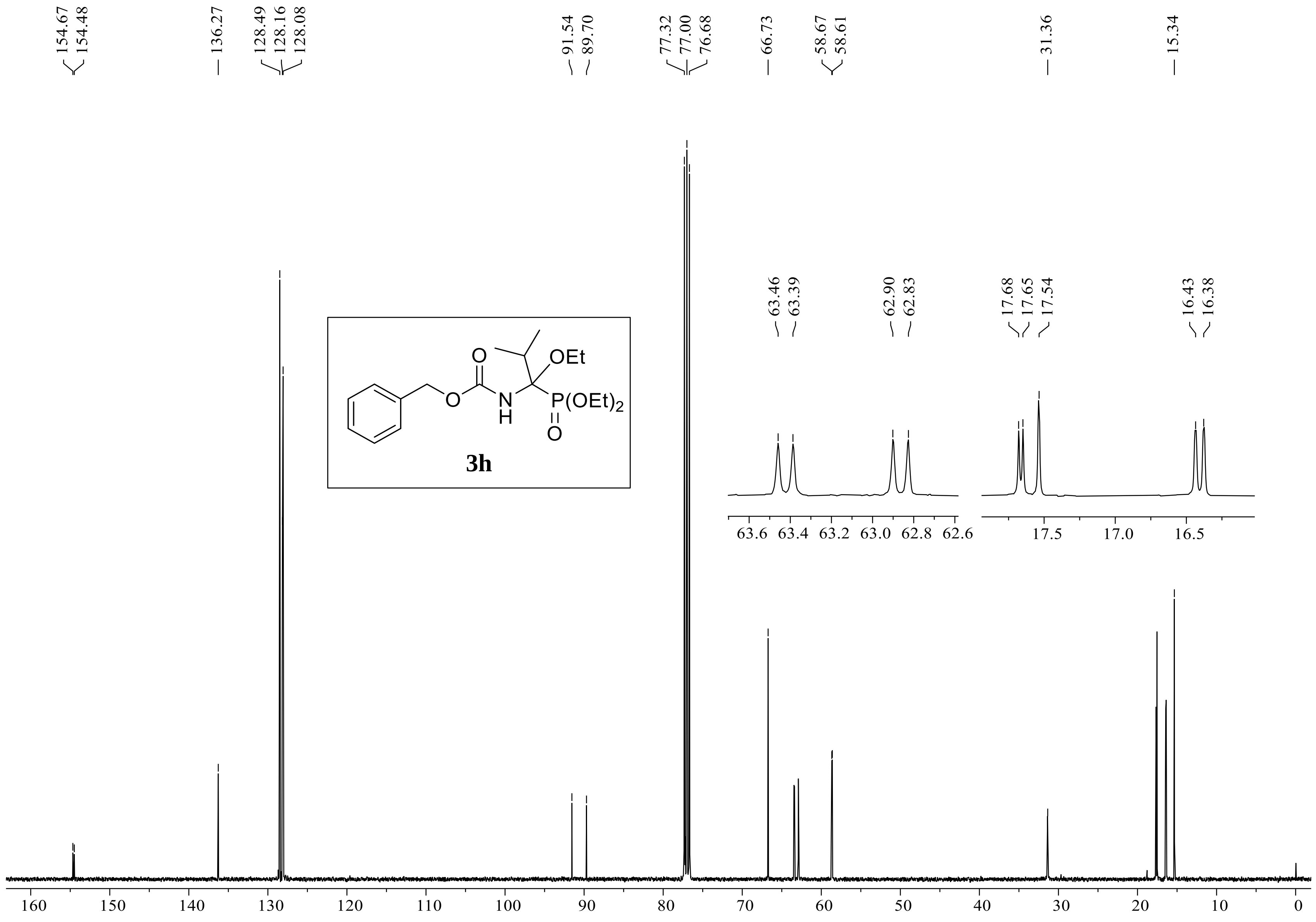

^{13}C NMR spectrum of *diethyl 1-(N-benzyloxycarbonylamino)-1-ethoxy-2-methylpropylphosphonate* (**3h**); 100 MHz/CDCl$_3$/TMS; δ (ppm).

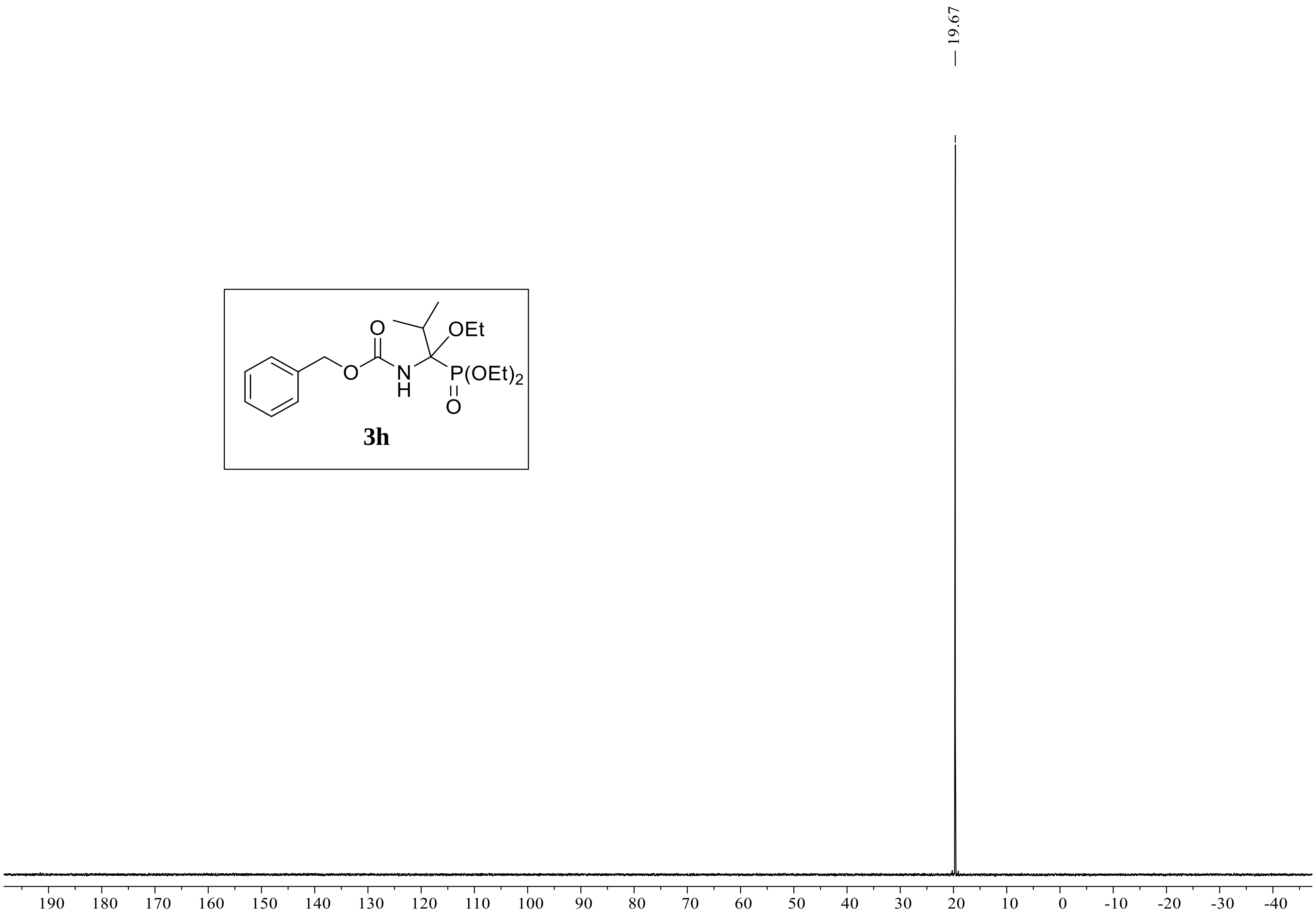

^{31}P NMR spectrum of *diethyl 1-(N-benzyloxycarbonylamino)-1-ethoxy-2-methylpropylphosphonate* (**3h**); 162 MHz/$CDCl_3$/TMS; δ (ppm).

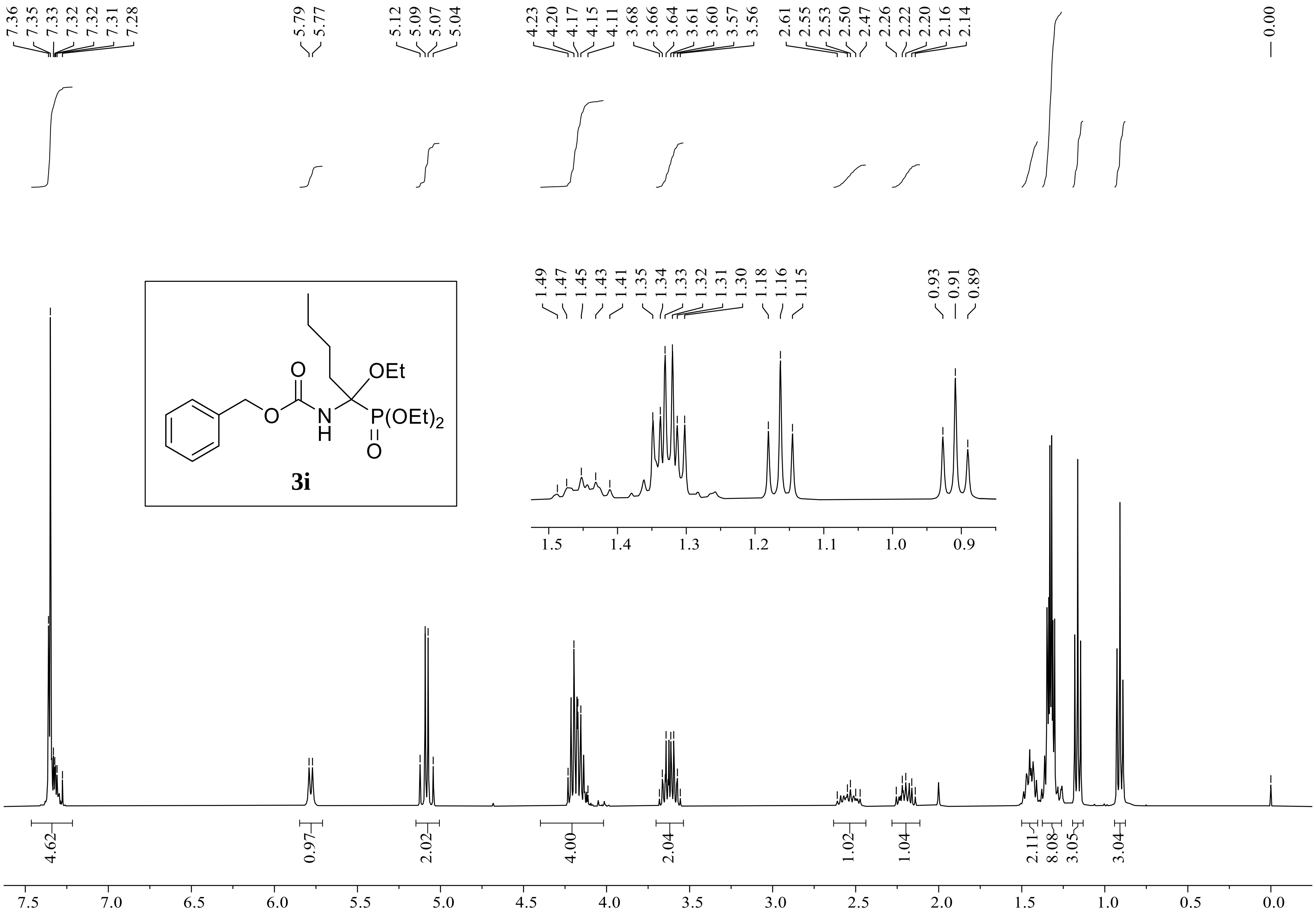

^{1}H NMR spectrum of *diethyl 1-(N-benzyloxycarbonylamino)-1-ethoxypentylphosphonate* (**3i**); 400 MHz/$CDCl_3$/TMS; δ (ppm).

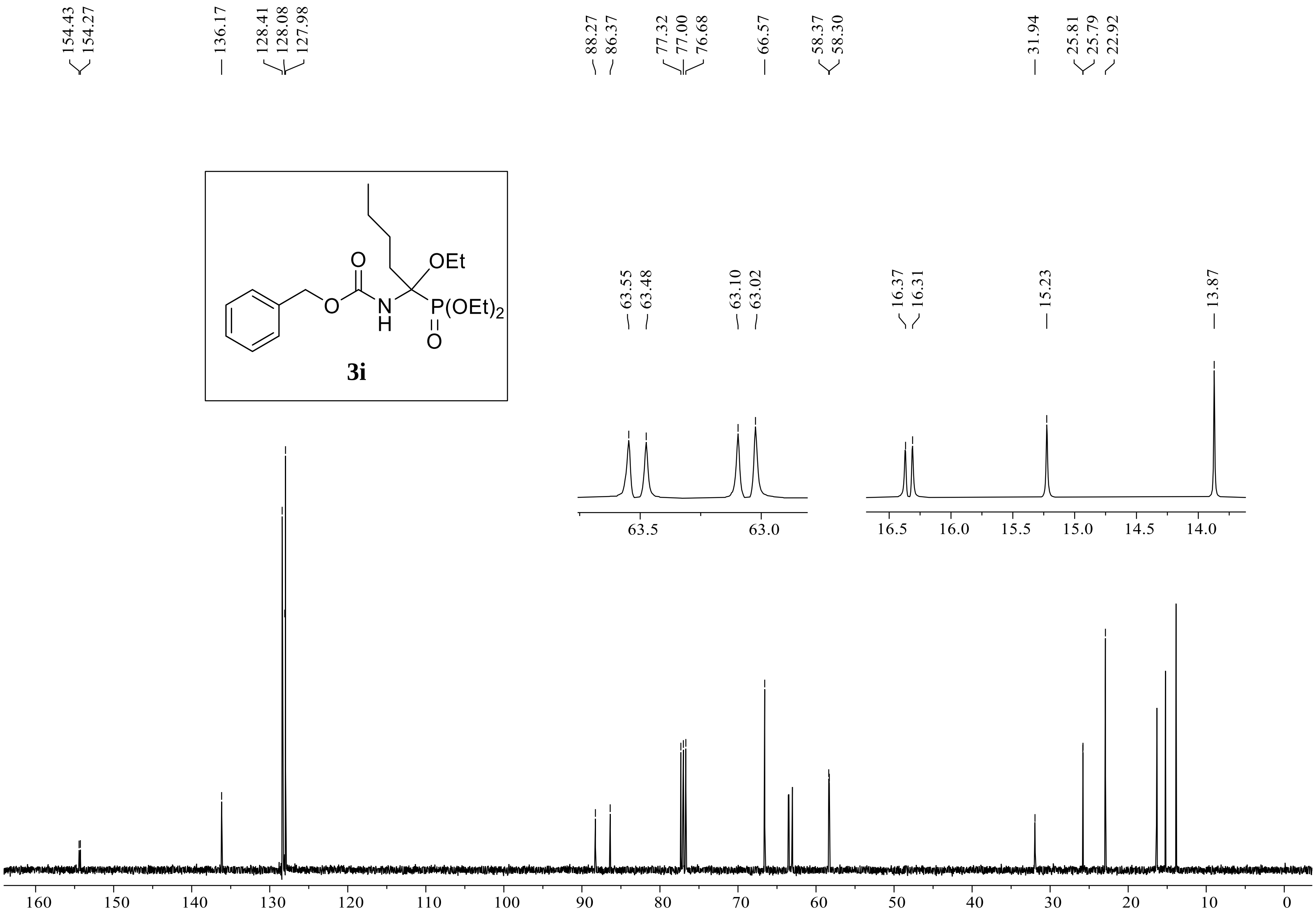

^{13}C NMR spectrum of *diethyl 1-(N-benzyloxycarbonylamino)-1-ethoxypentylphosphonate* (**3i**); 100 MHz/CDCl$_3$/TMS; δ (ppm).

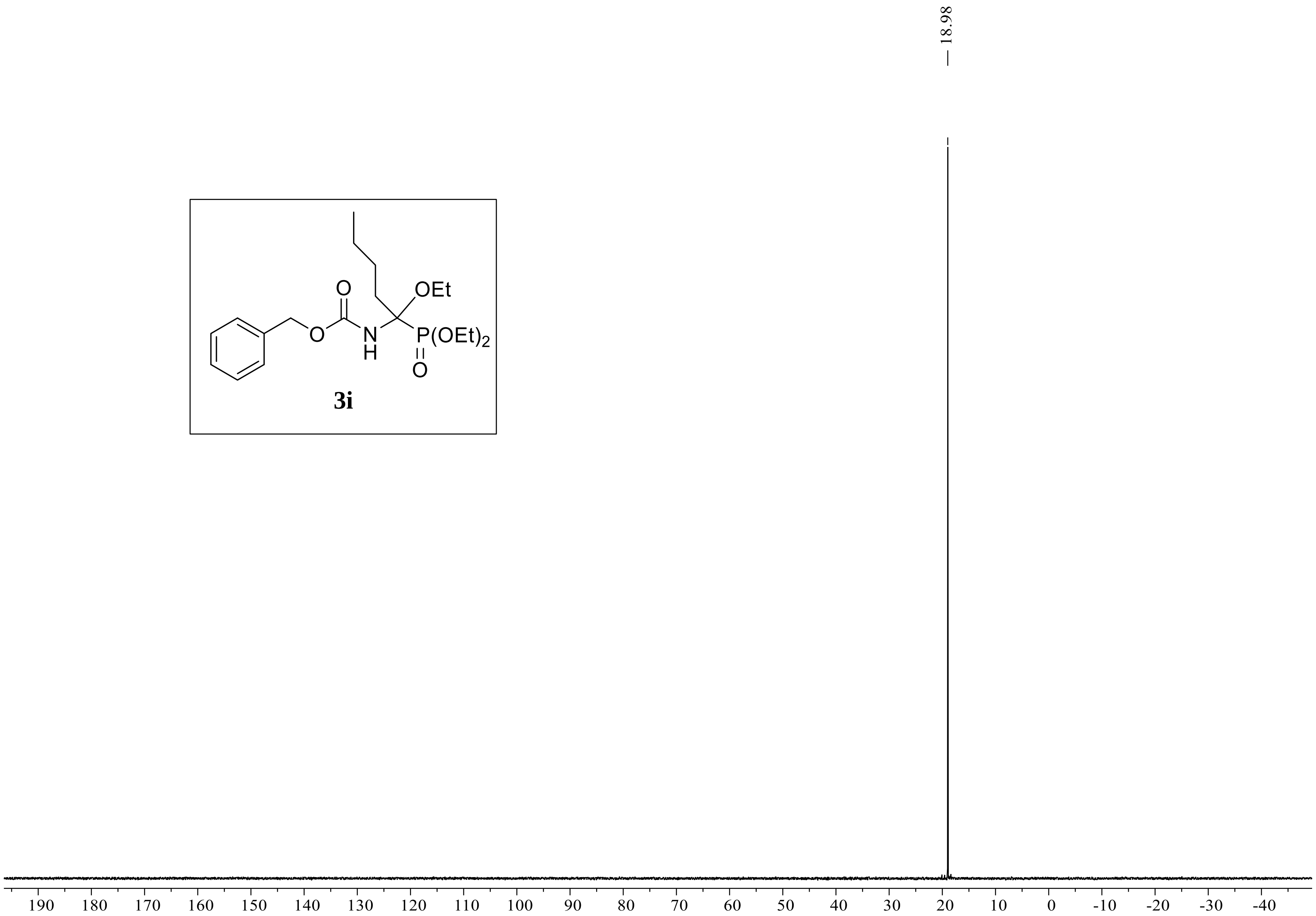

3i

31P NMR spectrum of *diethyl 1-(N-benzyloxycarbonylamino)-1-ethoxypentylphosphonate* (**3i**); 162 MHz/$CDCl_3$/TMS; δ (ppm).

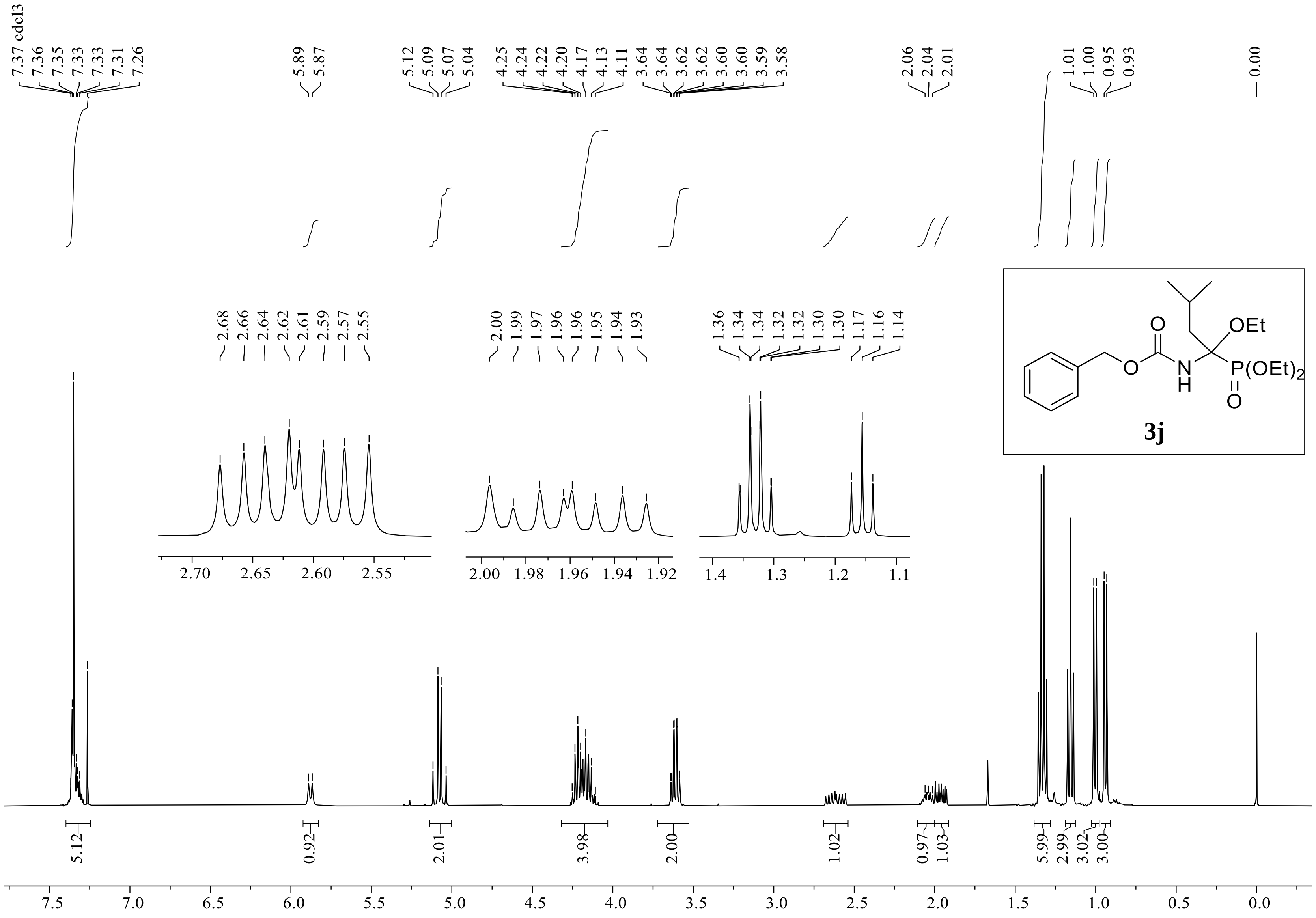

^{1}H NMR spectrum of *diethyl 1-(N-benzyloxycarbonylamino)-1-ethoxy-3-methylbutylphosphonate* (**3j**); 400 MHz/$CDCl_3$/TMS; δ (ppm).

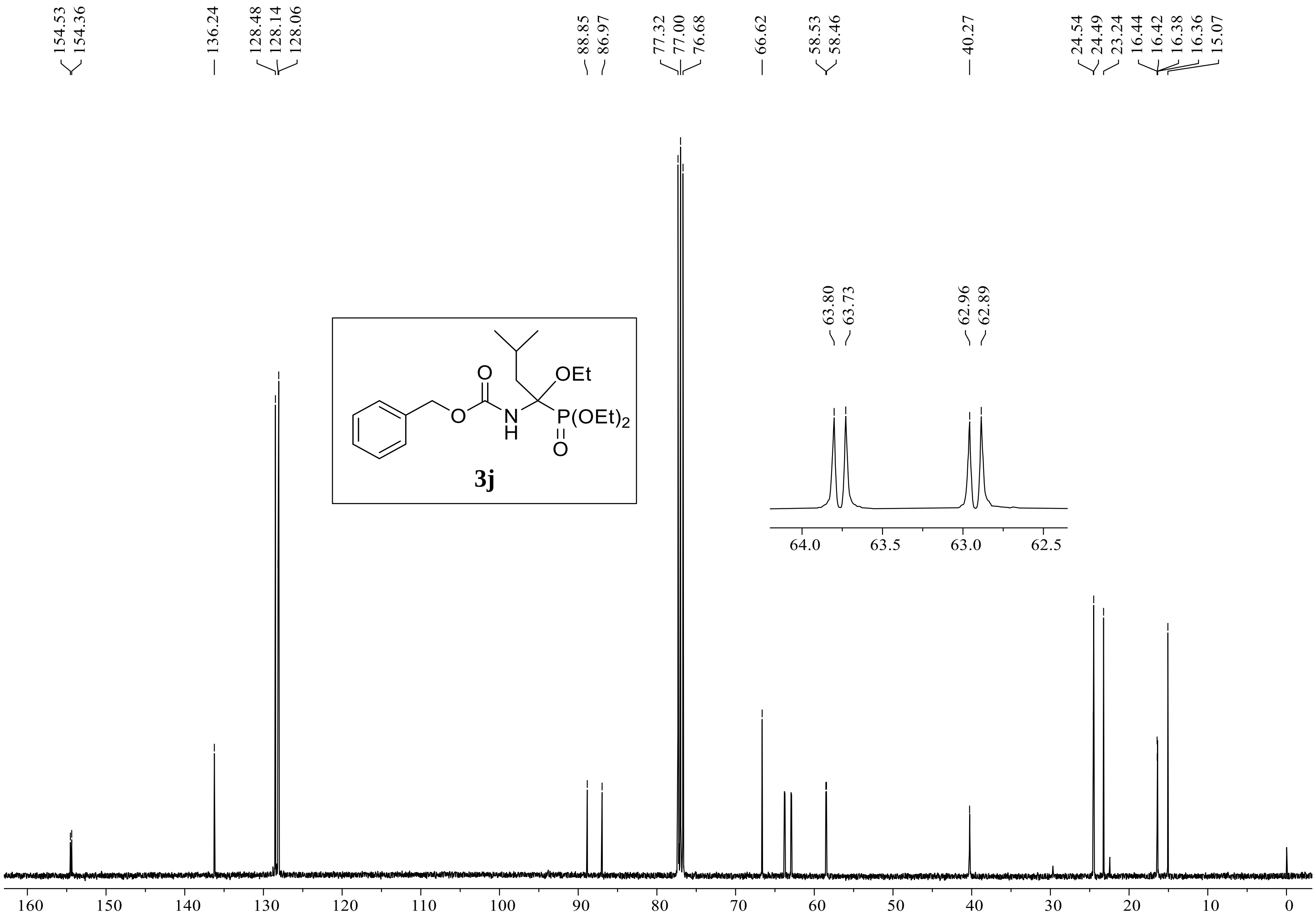

^{13}C NMR spectrum of *diethyl 1-(N-benzyloxycarbonylamino)-1-ethoxy-3-methylbutylphosphonate* (**3j**); 100 MHz/$CDCl_3$/TMS; δ (ppm).

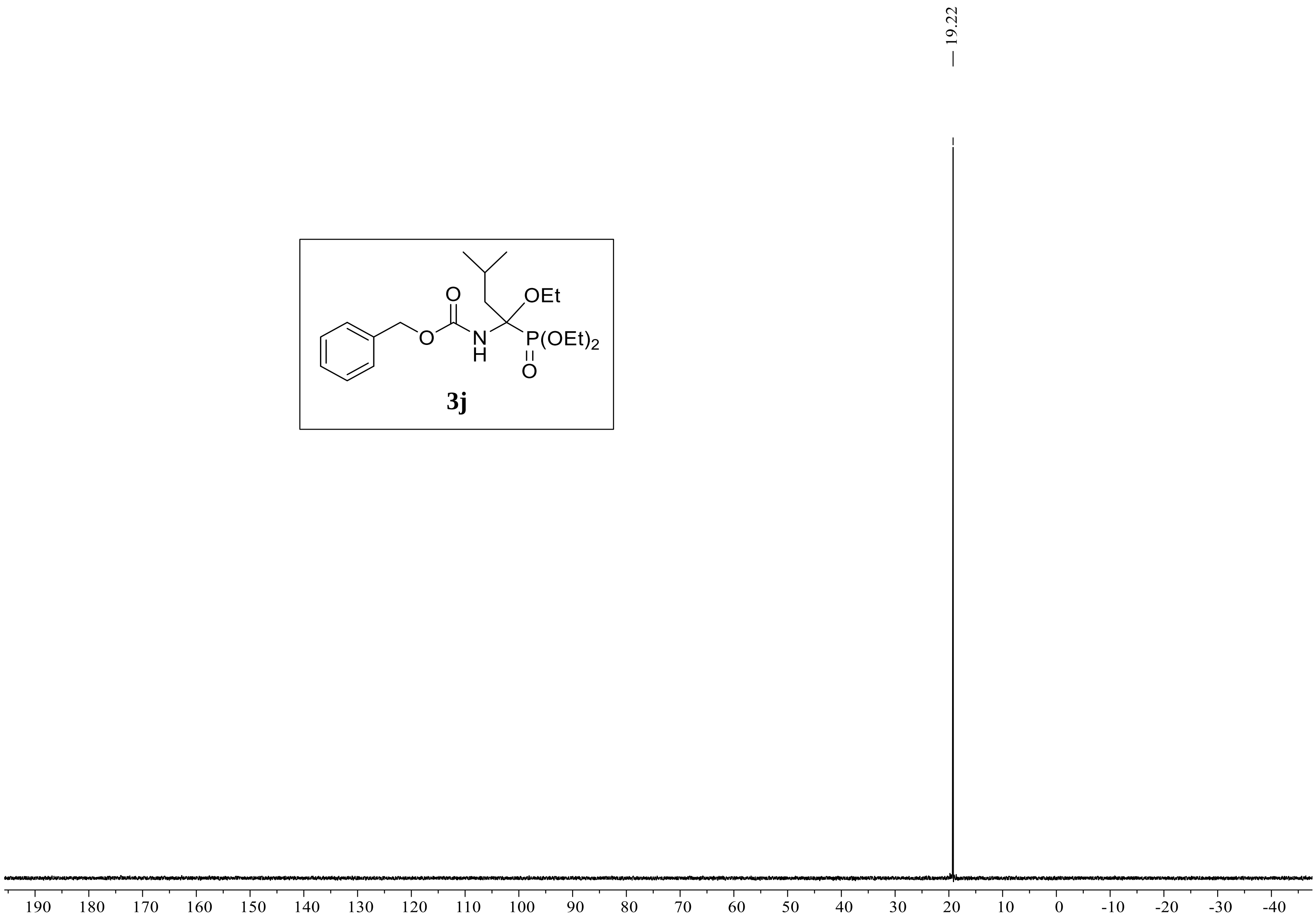

^{31}P NMR spectrum of *diethyl 1-(N-benzyloxycarbonylamino)-1-ethoxy-3-methylbutylphosphonate* (**3j**); 162 MHz/$CDCl_3$/TMS; δ (ppm).

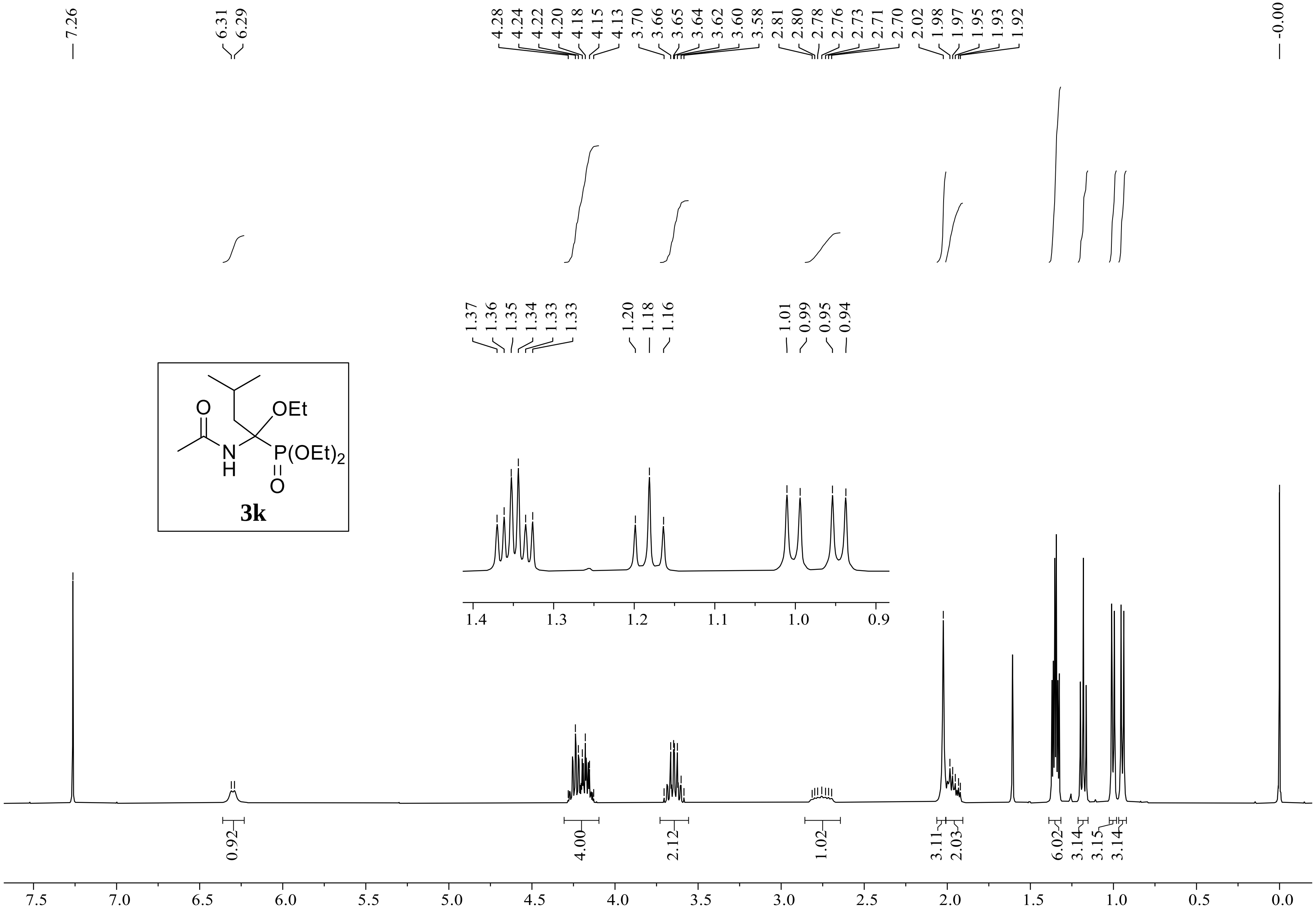

^{1}H NMR spectrum of *diethyl 1-(N-acetylamino)-1-ethoxy-3-methylbutylphosphonate* (**3k**); 400 MHz/$CDCl_3$/TMS; δ (ppm).

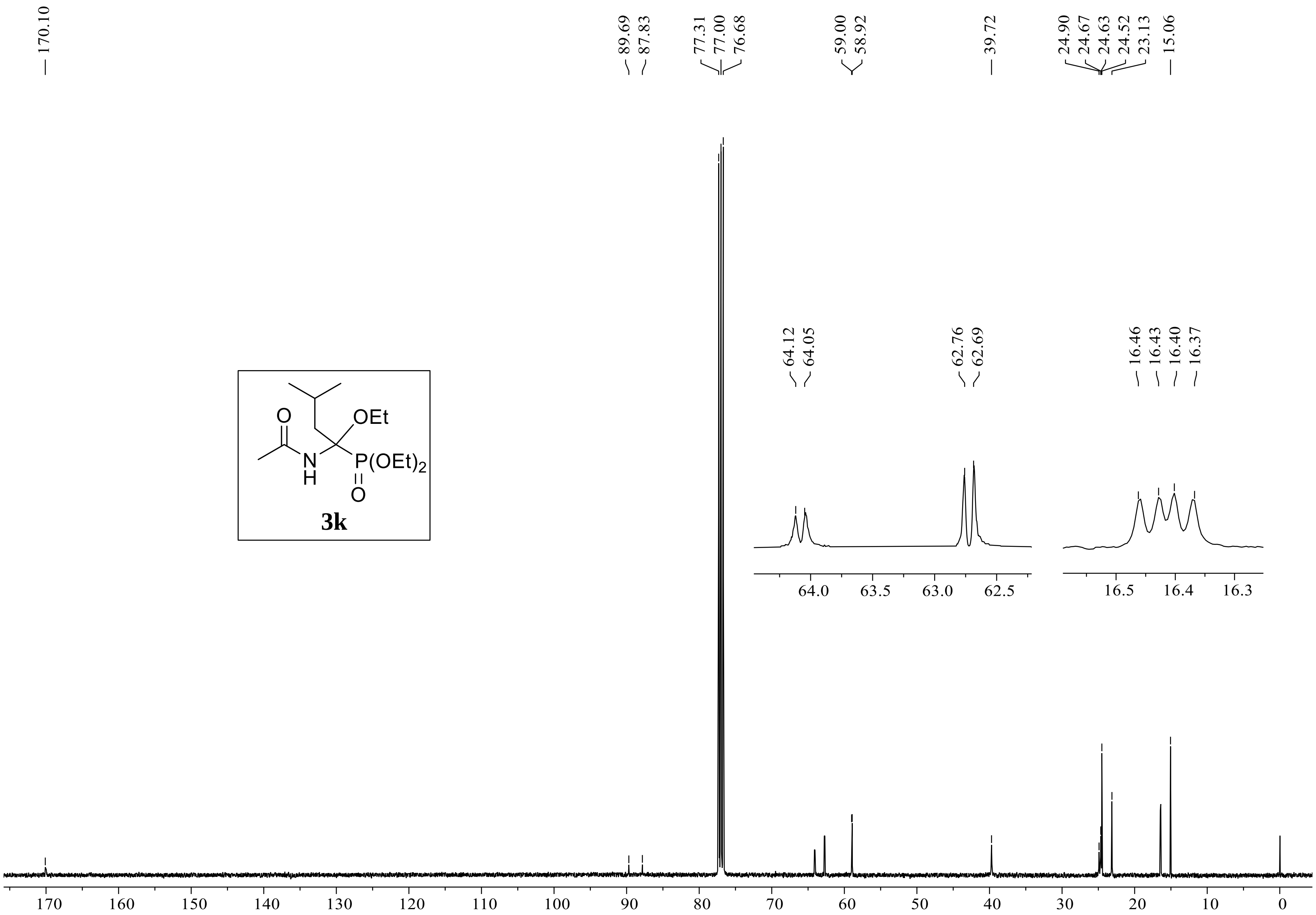

^{13}C NMR spectrum of *diethyl 1-(N-acetylamino)-1-ethoxy-3-methylbutylphosphonate* (**3k**); 100 MHz/$CDCl_3$/TMS; δ (ppm).

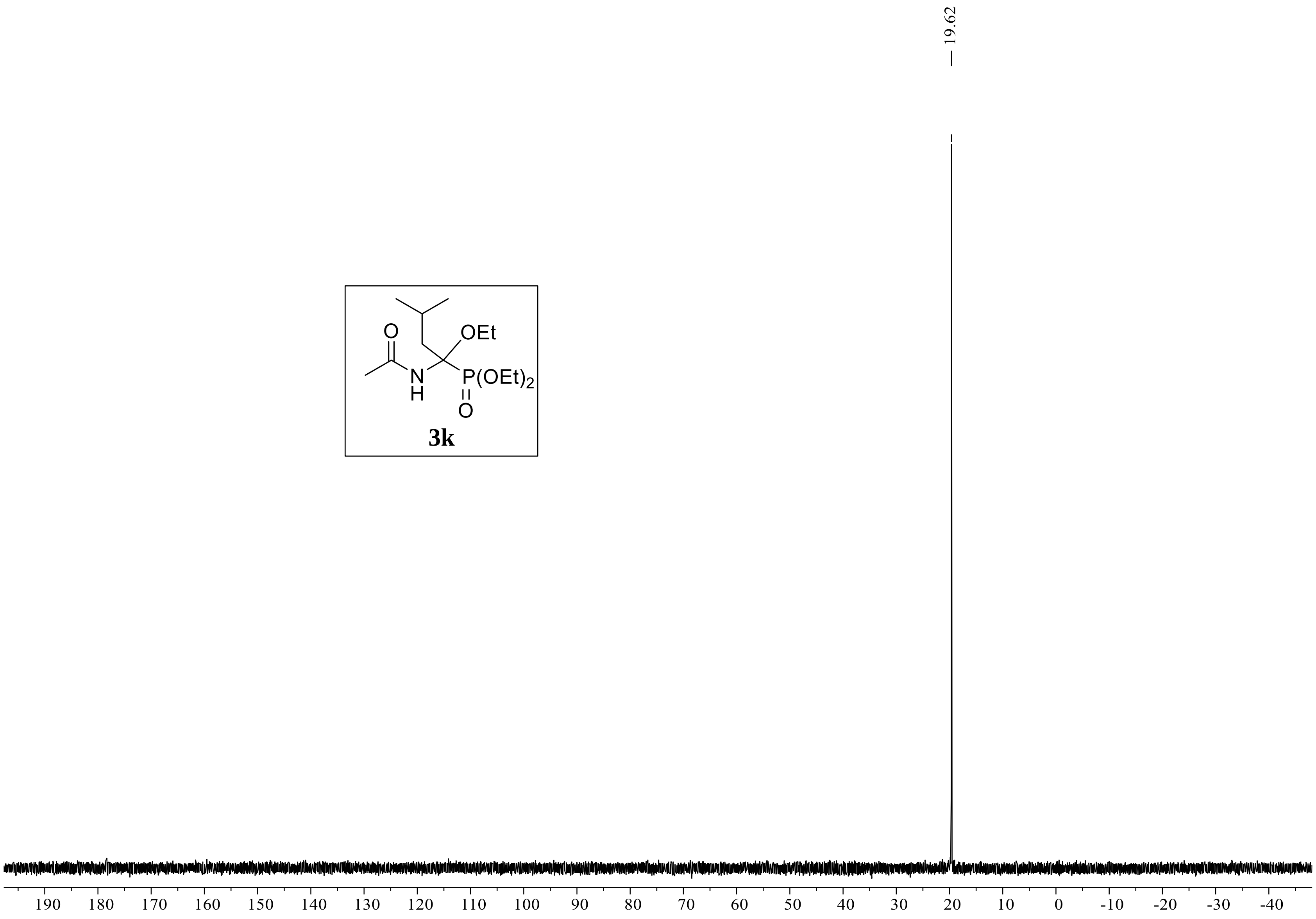

^{31}P NMR spectrum of *diethyl 1-(N-acetylamino)-1-ethoxy-3-methylbutylphosphonate* (**3k**); 162 MHz/$CDCl_3$/TMS; δ (ppm).

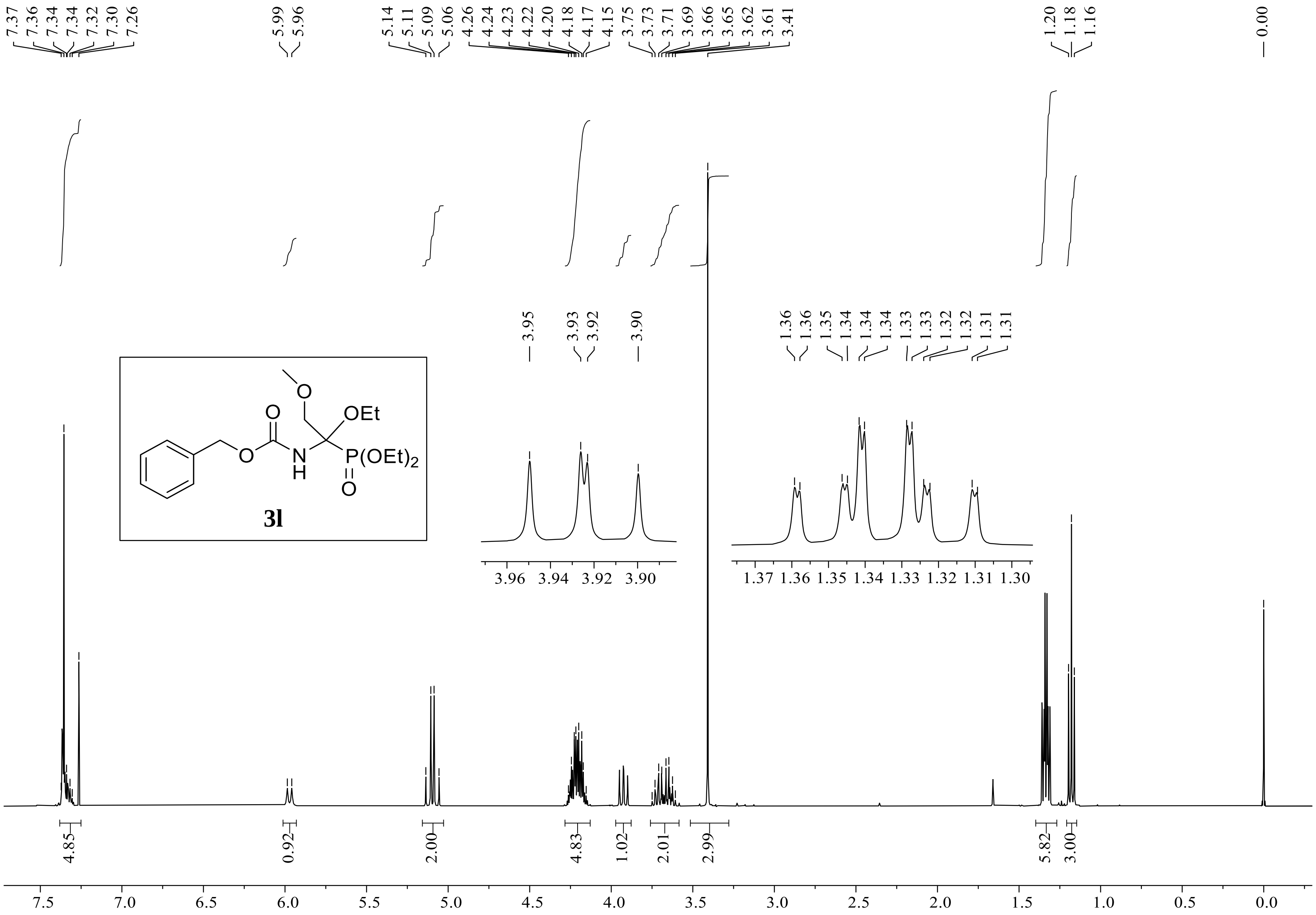

^{1}H NMR spectrum of *diethyl 1-(N-benzyloxycarbonylamino)-1-ethoxy-2-methoxyethylphosphonate* (**3l**); 400 MHz/$CDCl_3$/TMS; δ (ppm).

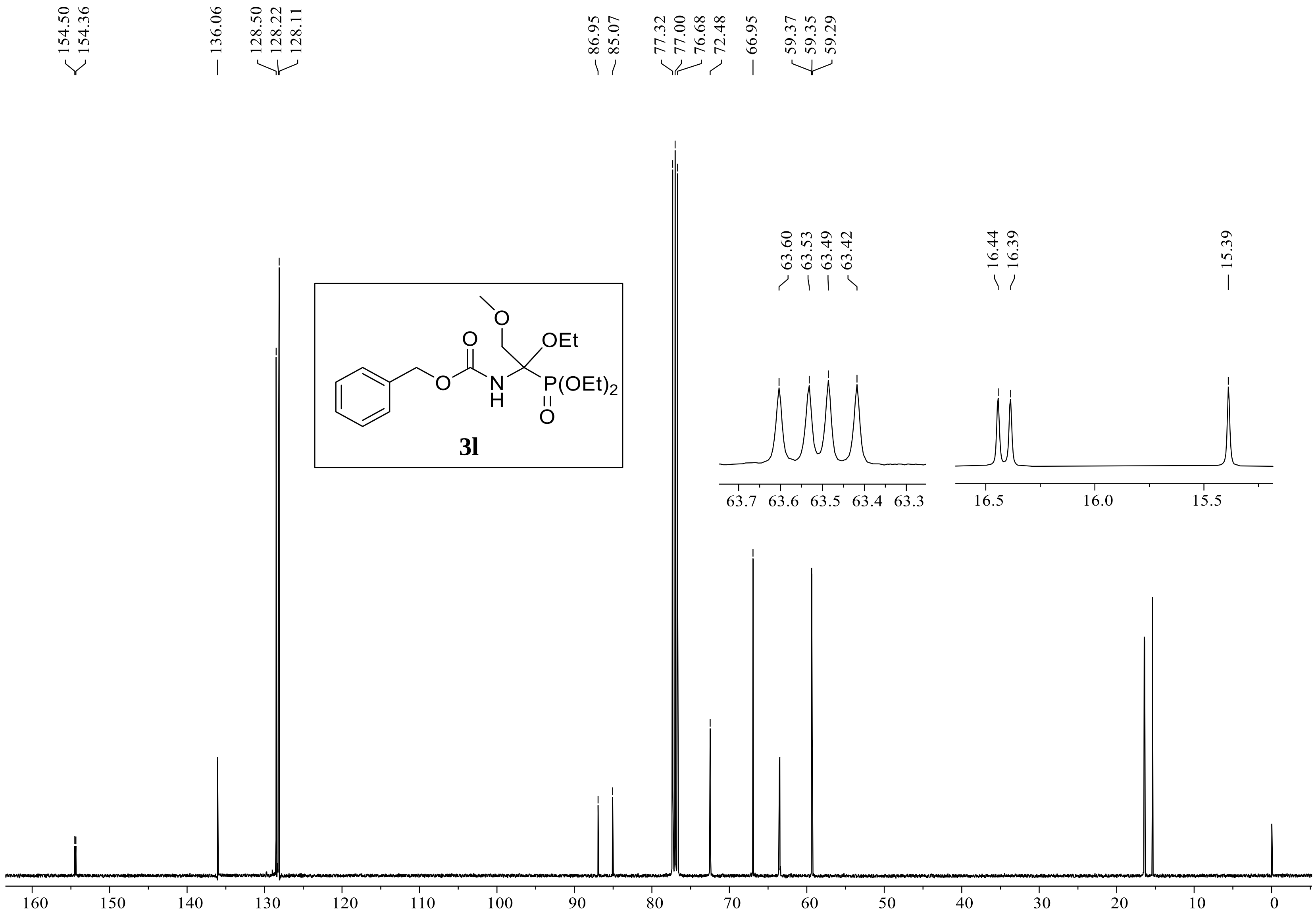

^{13}C NMR spectrum of *diethyl 1-(N-benzyloxycarbonylamino)-1-ethoxy-2-methoxyethylphosphonate* (**3l**); 100 MHz/$CDCl_3$/TMS; δ (ppm).

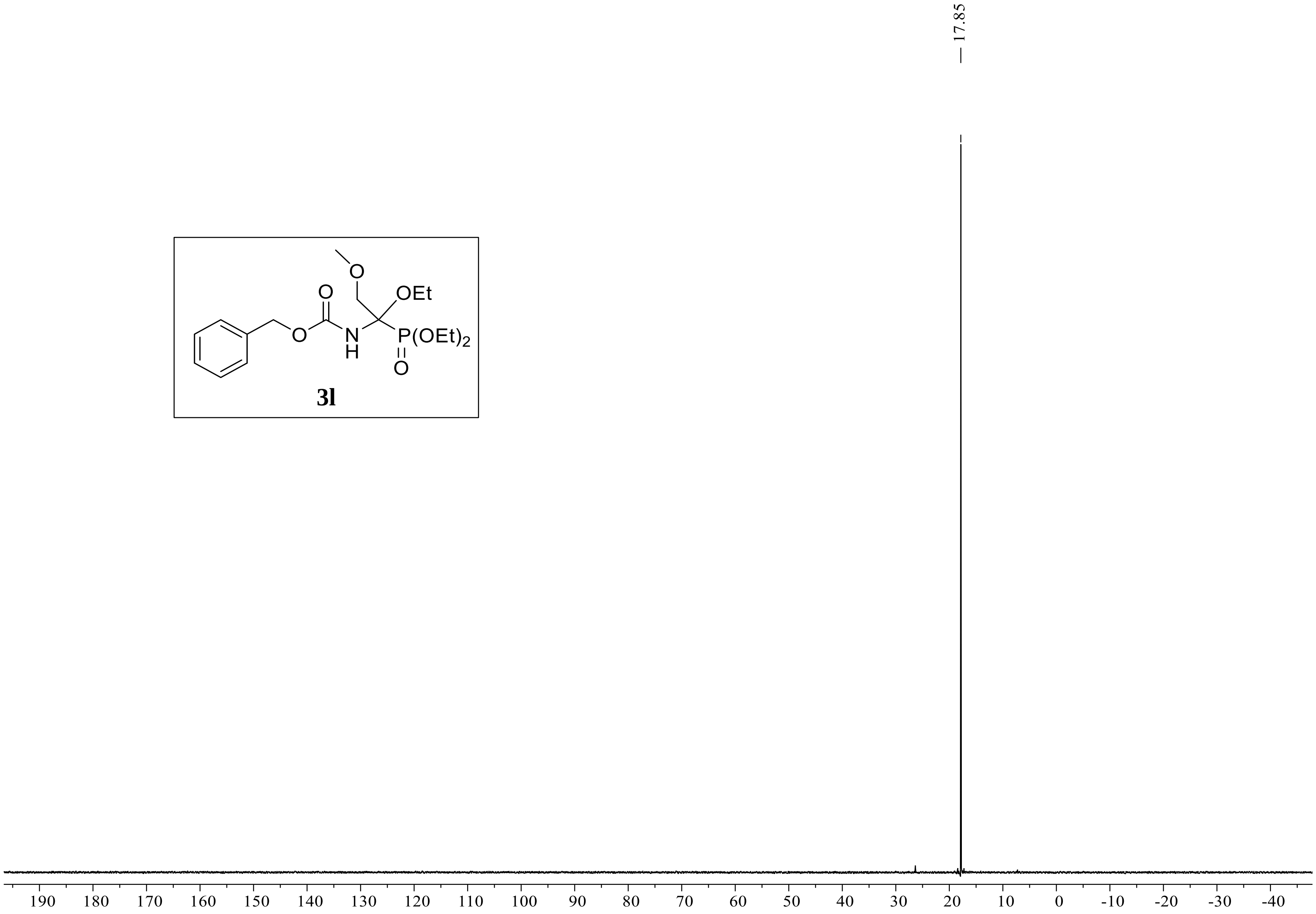

^{31}P NMR spectrum of *diethyl 1-(N-benzyloxycarbonylamino)-1-ethoxy-2-methoxyethylphosphonate* (**3l**); 162 MHz/$CDCl_3$/TMS; δ (ppm).

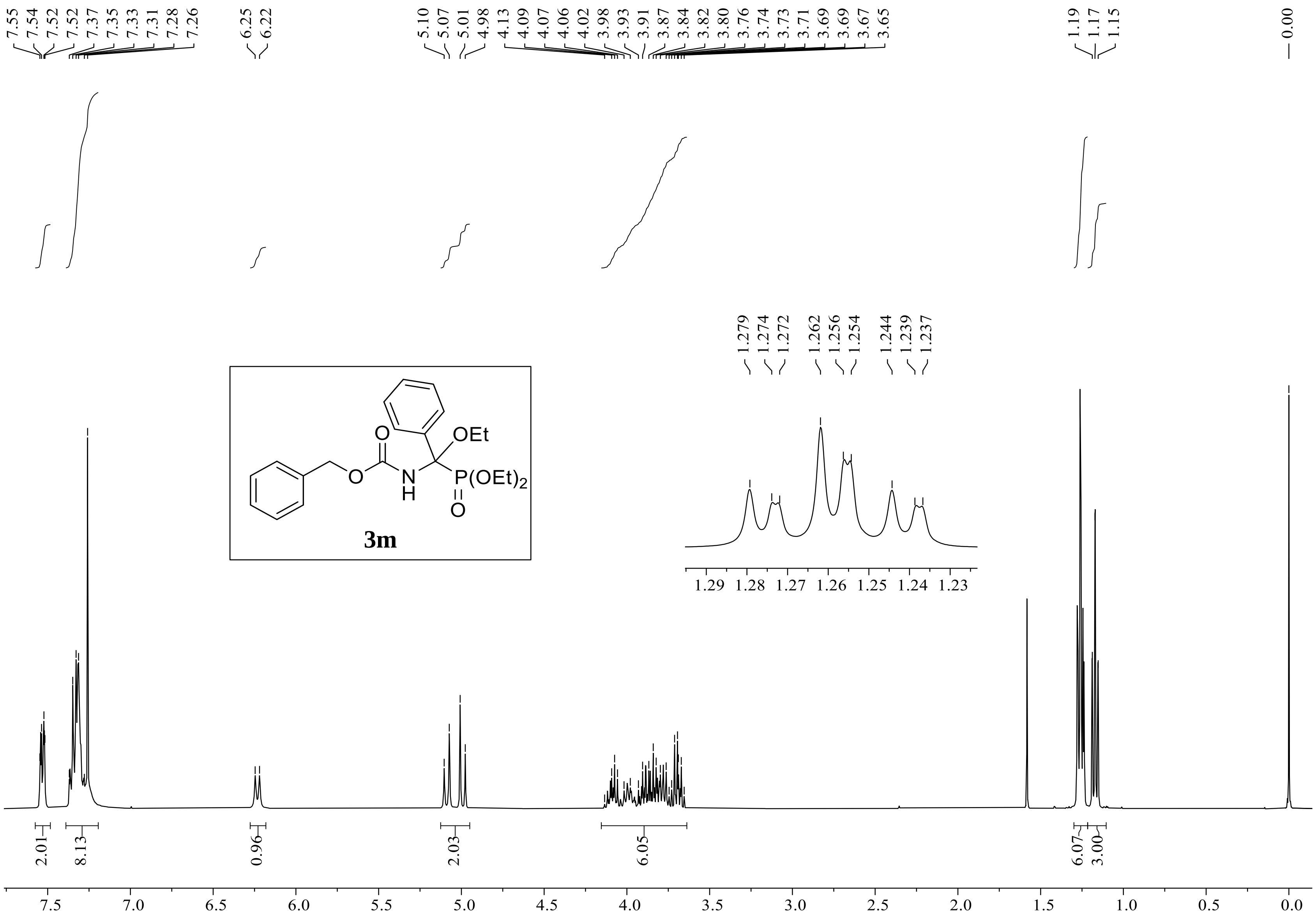

^{1}H NMR spectrum of *diethyl 1-(N-benzyloxycarbonylamino)-1-ethoxy-1-phenylmethylphosphonate* (**3m**); 400 MHz/$CDCl_3$/TMS; δ (ppm).

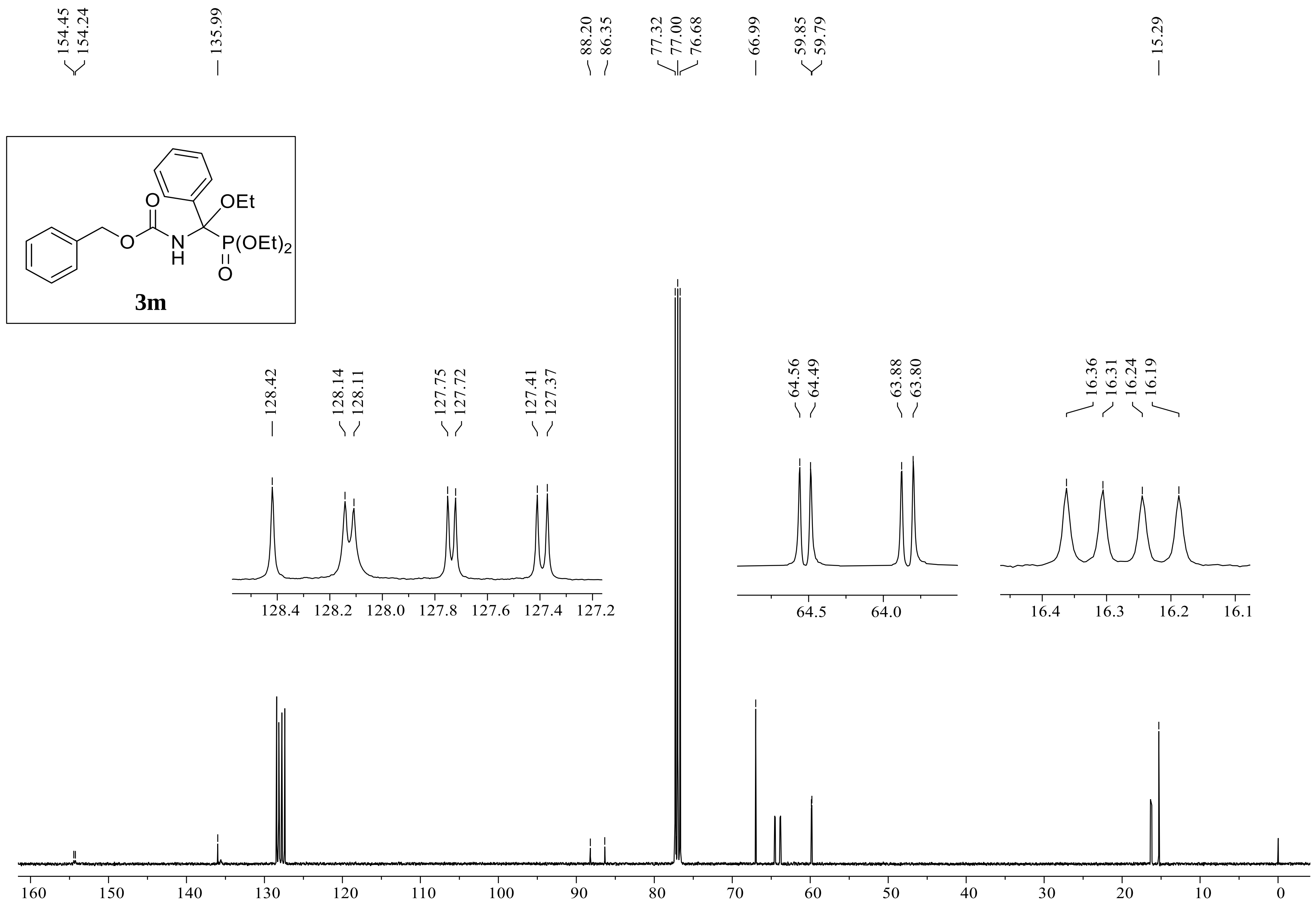

^{13}C NMR spectrum of *diethyl 1-(N-benzyloxycarbonylamino)-1-ethoxy-1-phenylmethylphosphonate* (**3m**); 100 MHz/$CDCl_3$/TMS; δ (ppm).

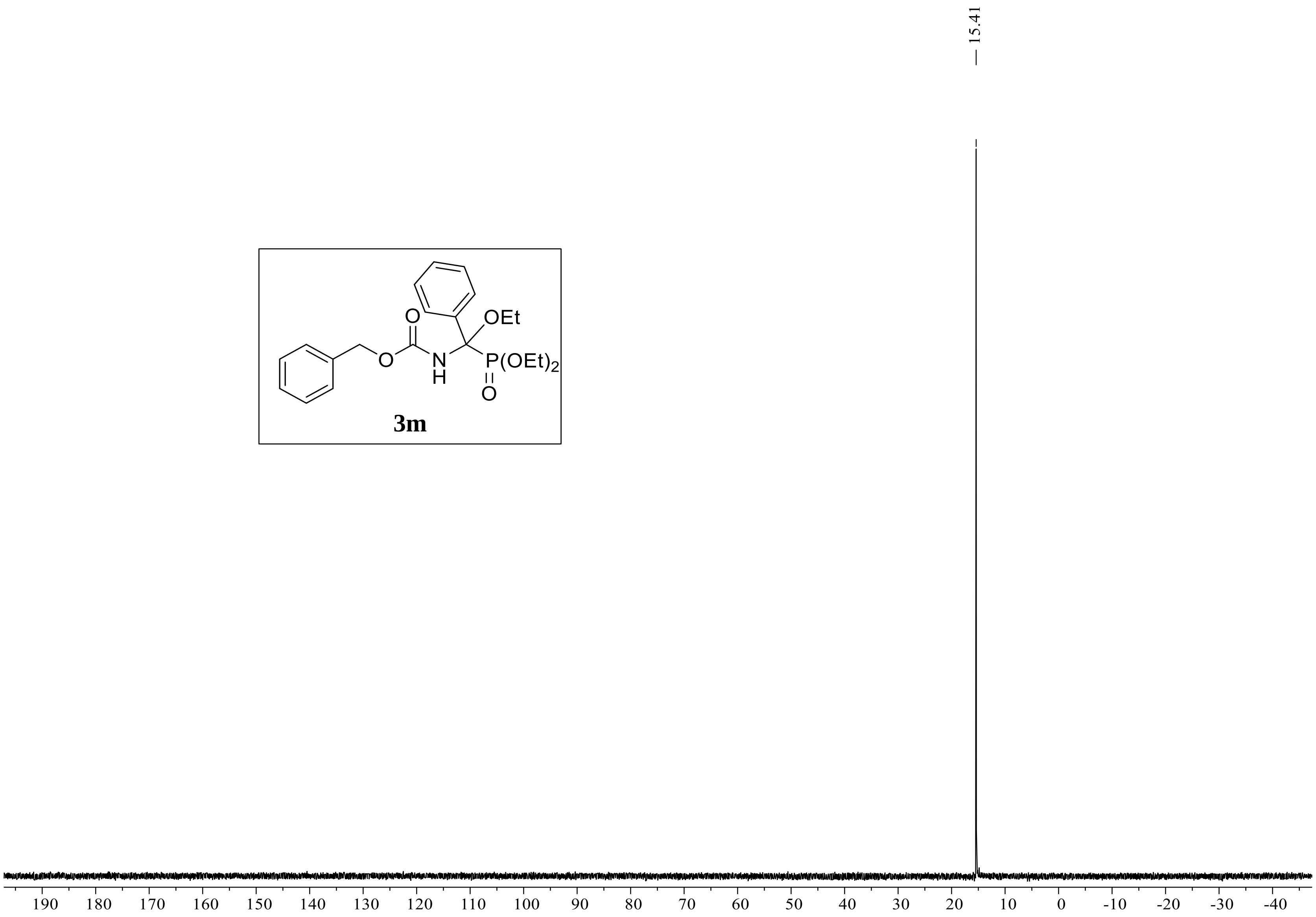

^{31}P NMR spectrum of *diethyl 1-(N-benzyloxycarbonylamino)-1-ethoxy-1-phenylmethylphosphonate* (**3m**); 162 MHz/$CDCl_3$/TMS; δ (ppm).

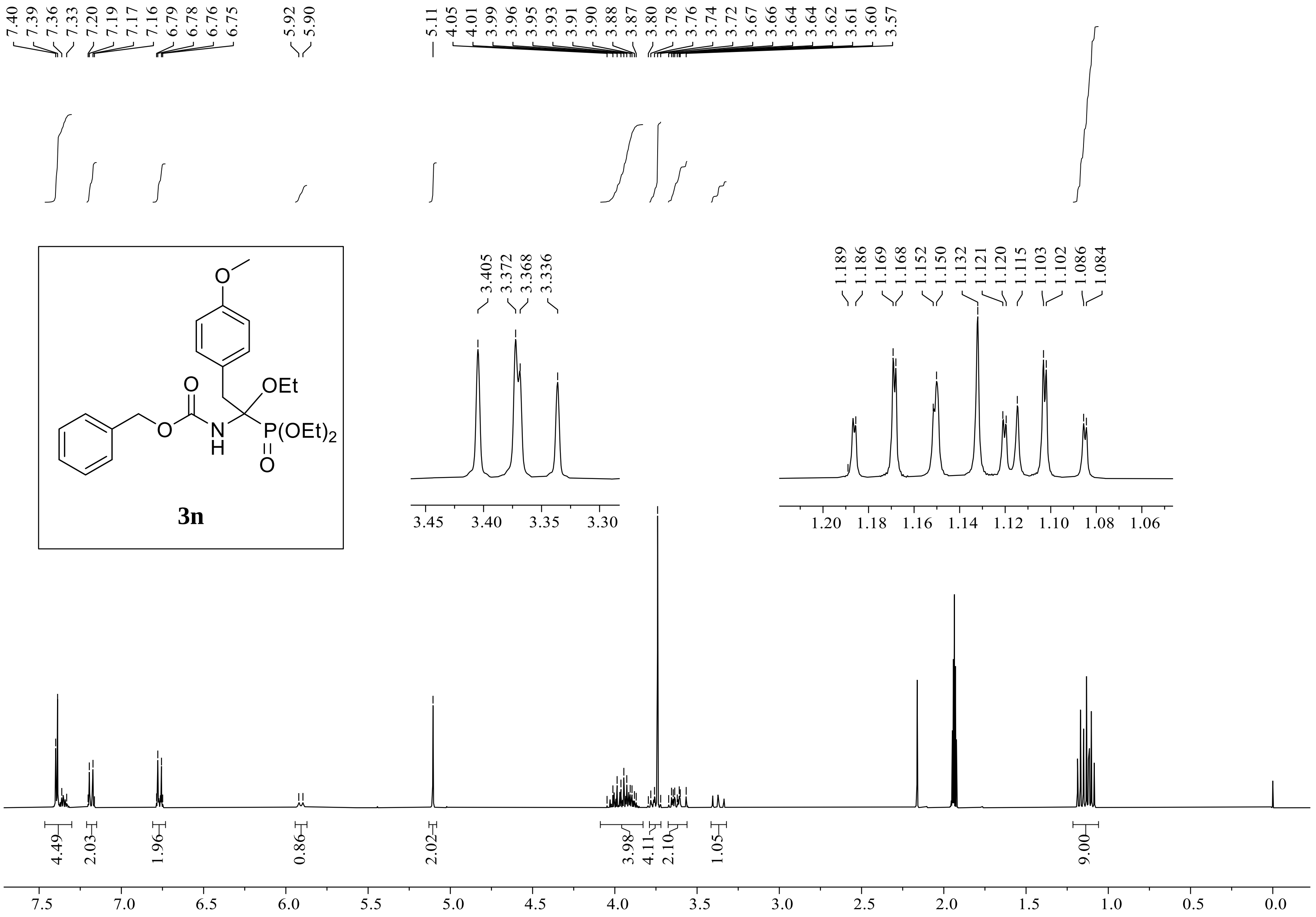

^{1}H NMR spectrum of *diethyl 1-(N-benzyloxycarbonylamino)-1-ethoxy-2-(4-methoxyphenyl)ethylphosphonate* (**3n**); 400 MHz/CD_3CN/TMS; δ (ppm).

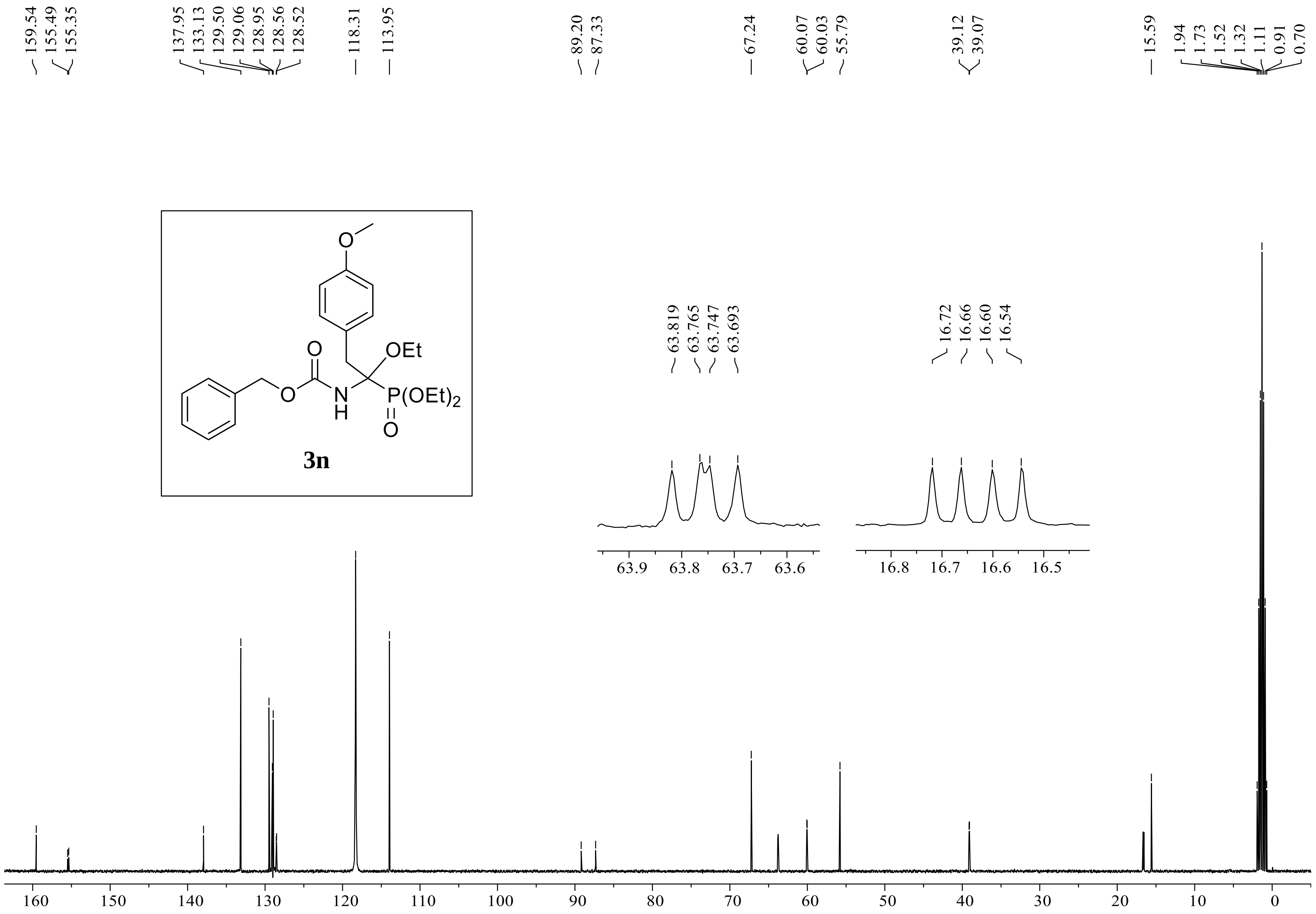

^{13}C NMR spectrum of *diethyl 1-(N-benzyloxycarbonylamino)-1-ethoxy-2-(4-methoxyphenyl)ethylphosphonate* (**3n**); 100 MHz/CD_3CN/TMS; δ (ppm).

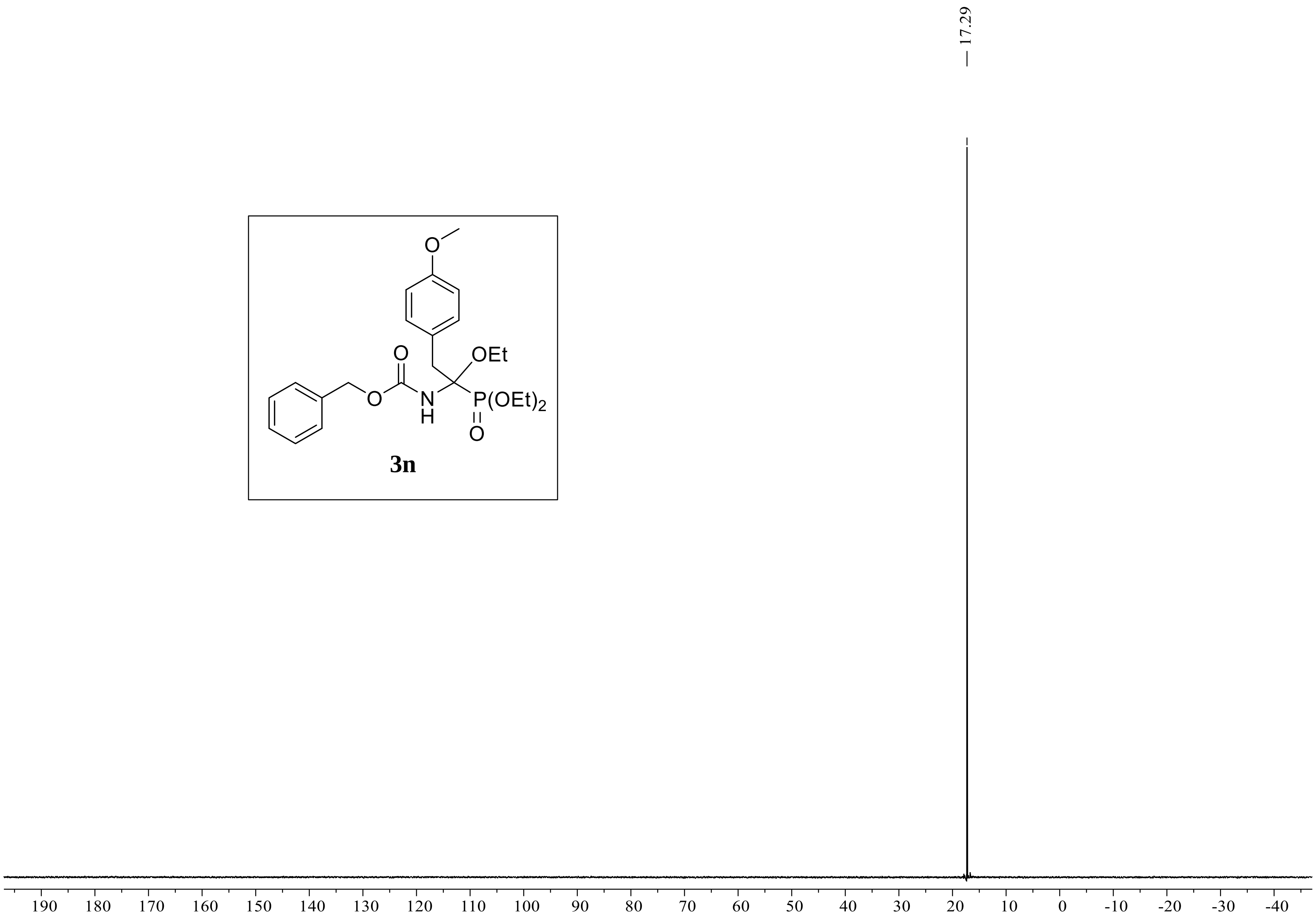

^{31}P NMR spectrum of *diethyl 1-(N-benzyloxycarbonylamino)-1-ethoxy-2-(4-methoxyphenyl)ethylphosphonate* (**3n**); 162 MHz/ CD_3CN/TMS; δ (ppm).

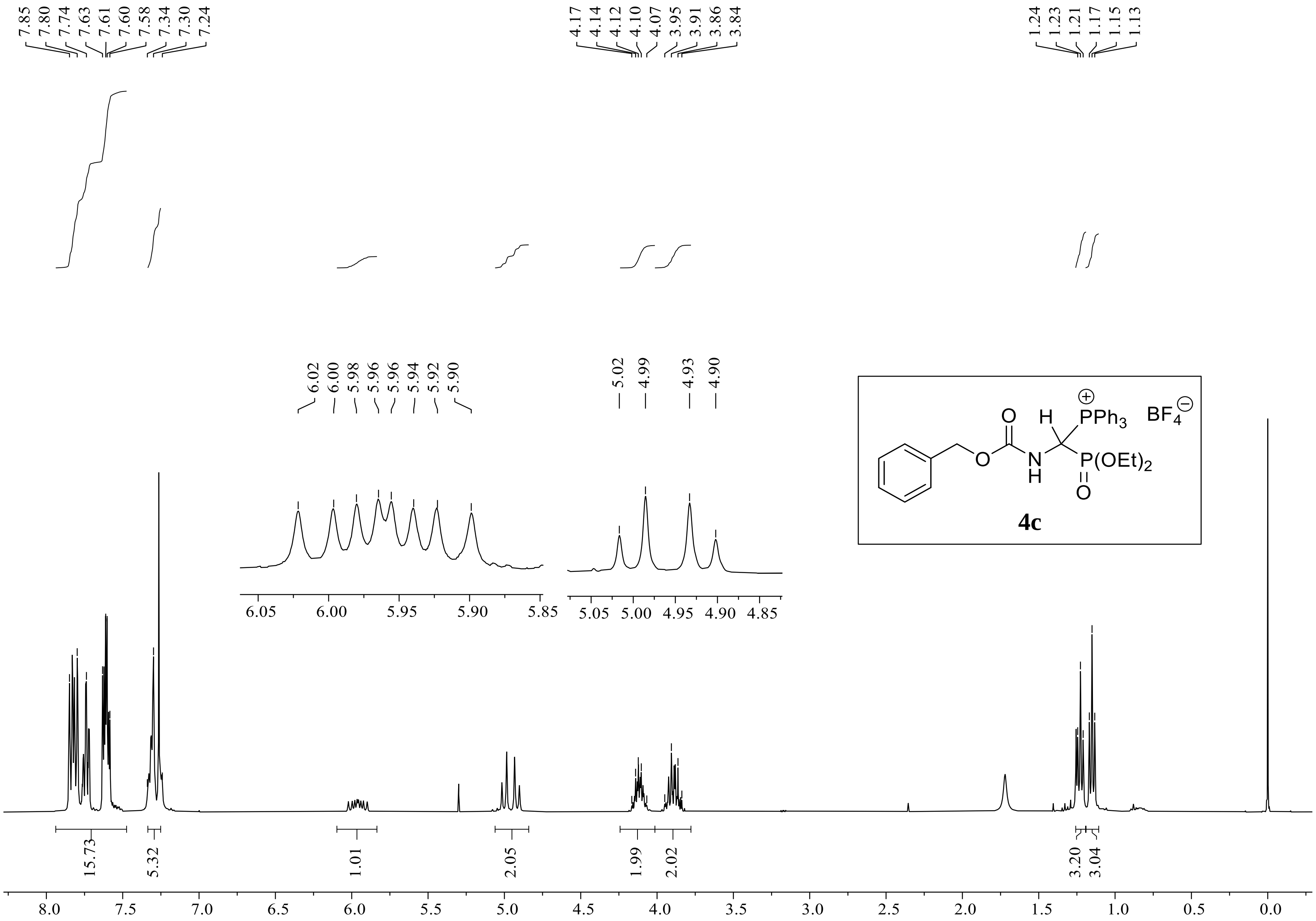

^{1}H NMR spectrum of d*iethyl 1-(N-benzyloxycarbonylamino)-1-triphenylphosphoniummethylphosphonate* (**4c**); 400 MHz/$CDCl_3$/TMS; δ (ppm).

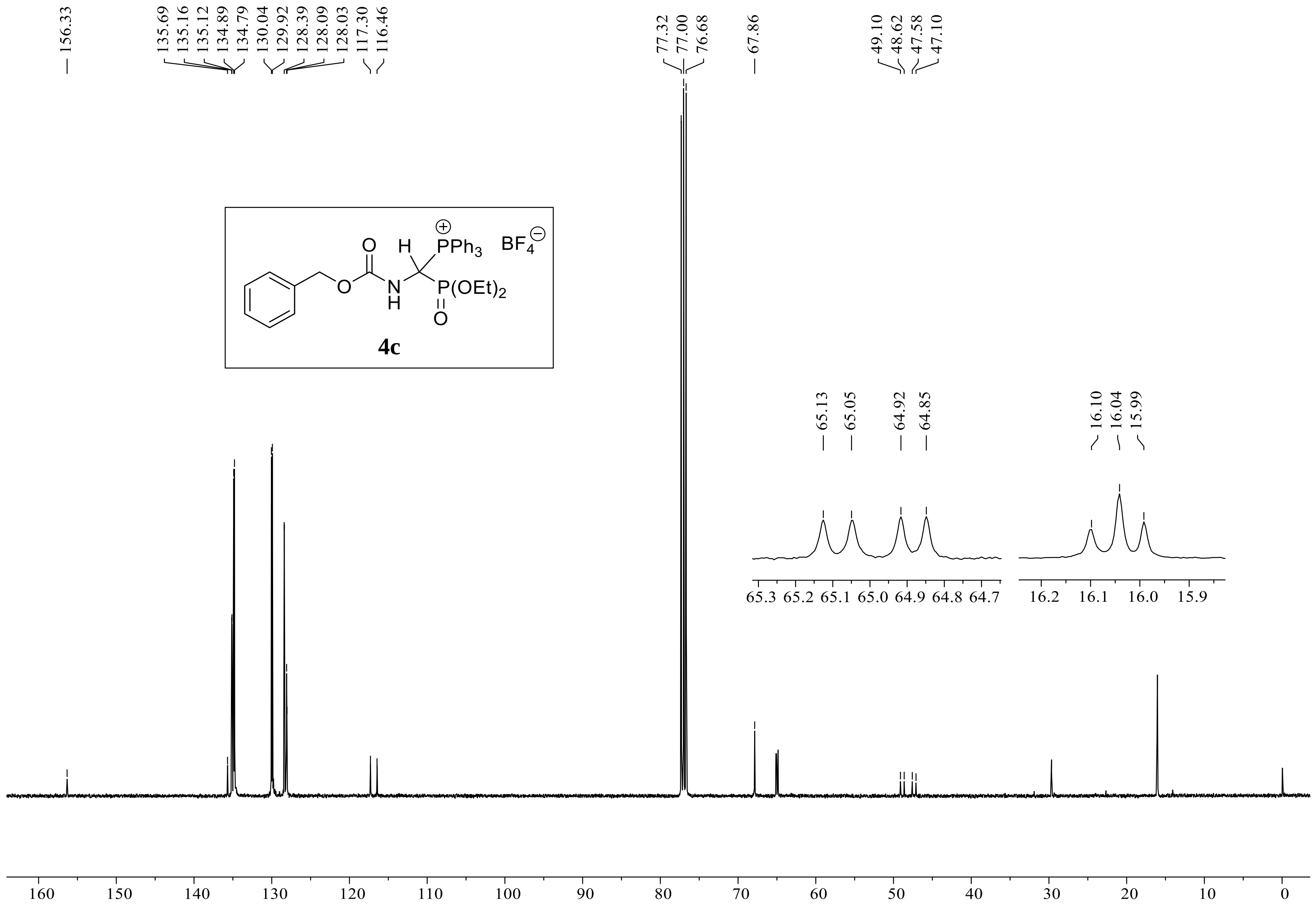

4c

^{13}C NMR spectrum of d*iethyl 1-(N-benzyloxycarbonylamino)-1-triphenylphosphoniummethylphosphonate* (**4c**); 400 MHz/$CDCl_3$/TMS; δ (ppm).

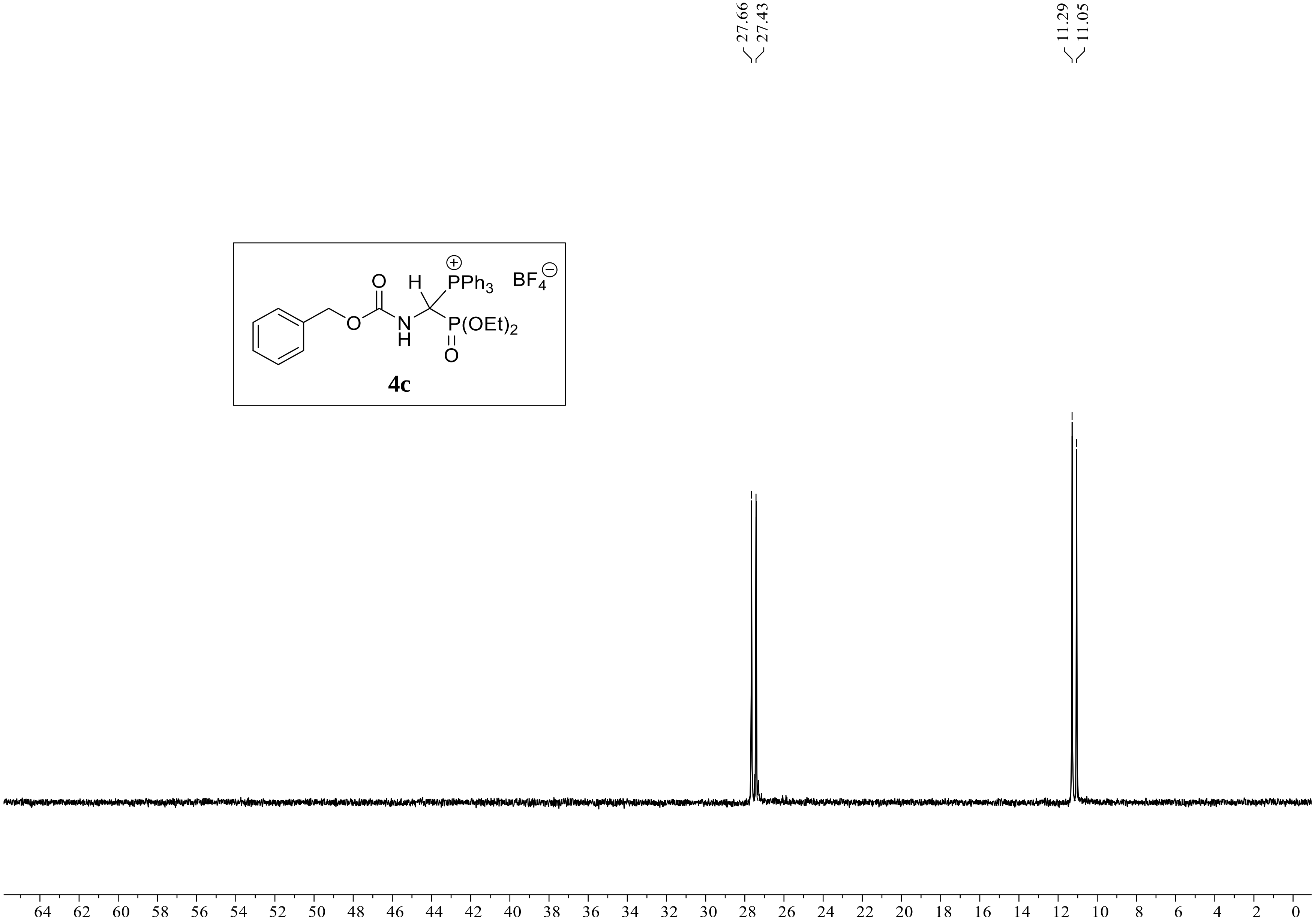

^{31}P NMR spectrum of d*iethyl 1-(N-benzyloxycarbonylamino)-1-triphenylphosphoniummethylphosphonate* (**4c**); 400 MHz/$CDCl_3$/TMS; δ (ppm).

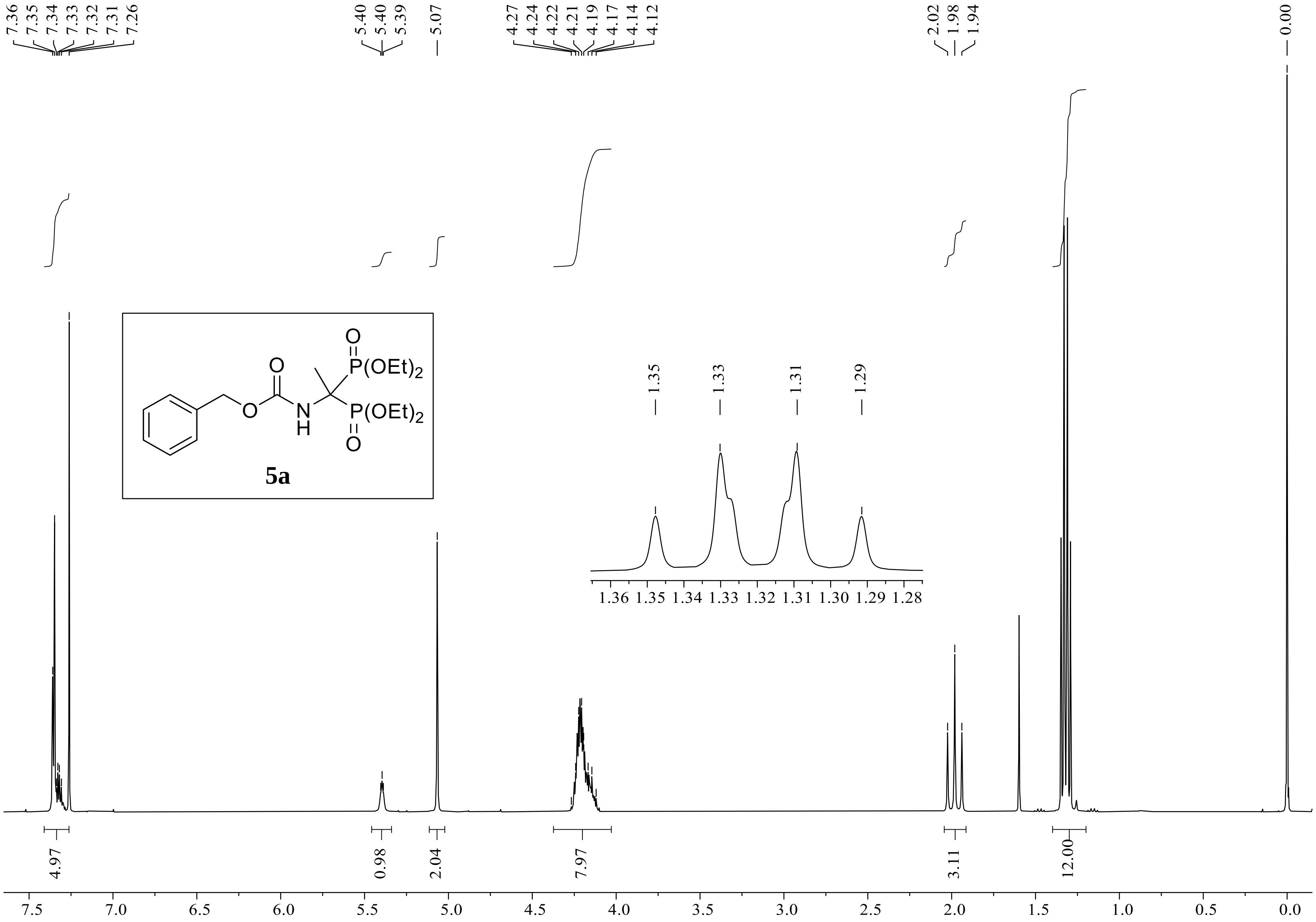

^{1}H NMR spectrum of *tetraethyl 1-(N-benzyloxycarbonylamino)ethylene-1,1-bisphosphonate* (**5a**); 162 MHz/$CDCl_3$/TMS; δ (ppm).

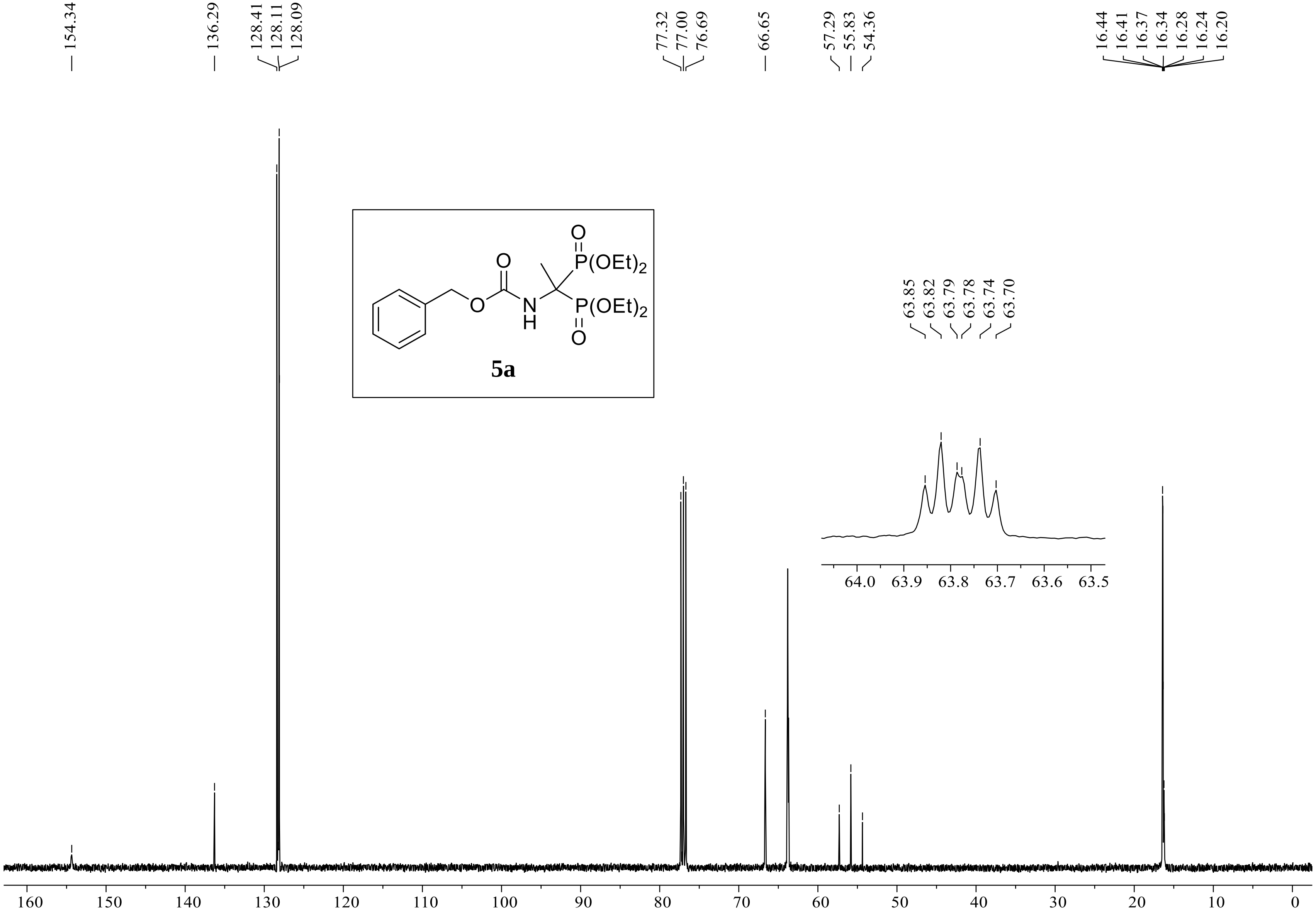

^{13}C NMR spectrum of *tetraethyl 1-(N-benzyloxycarbonylamino)ethylene-1,1-bisphosphonate* (**5a**); 162 MHz/$CDCl_3$/TMS; δ (ppm).

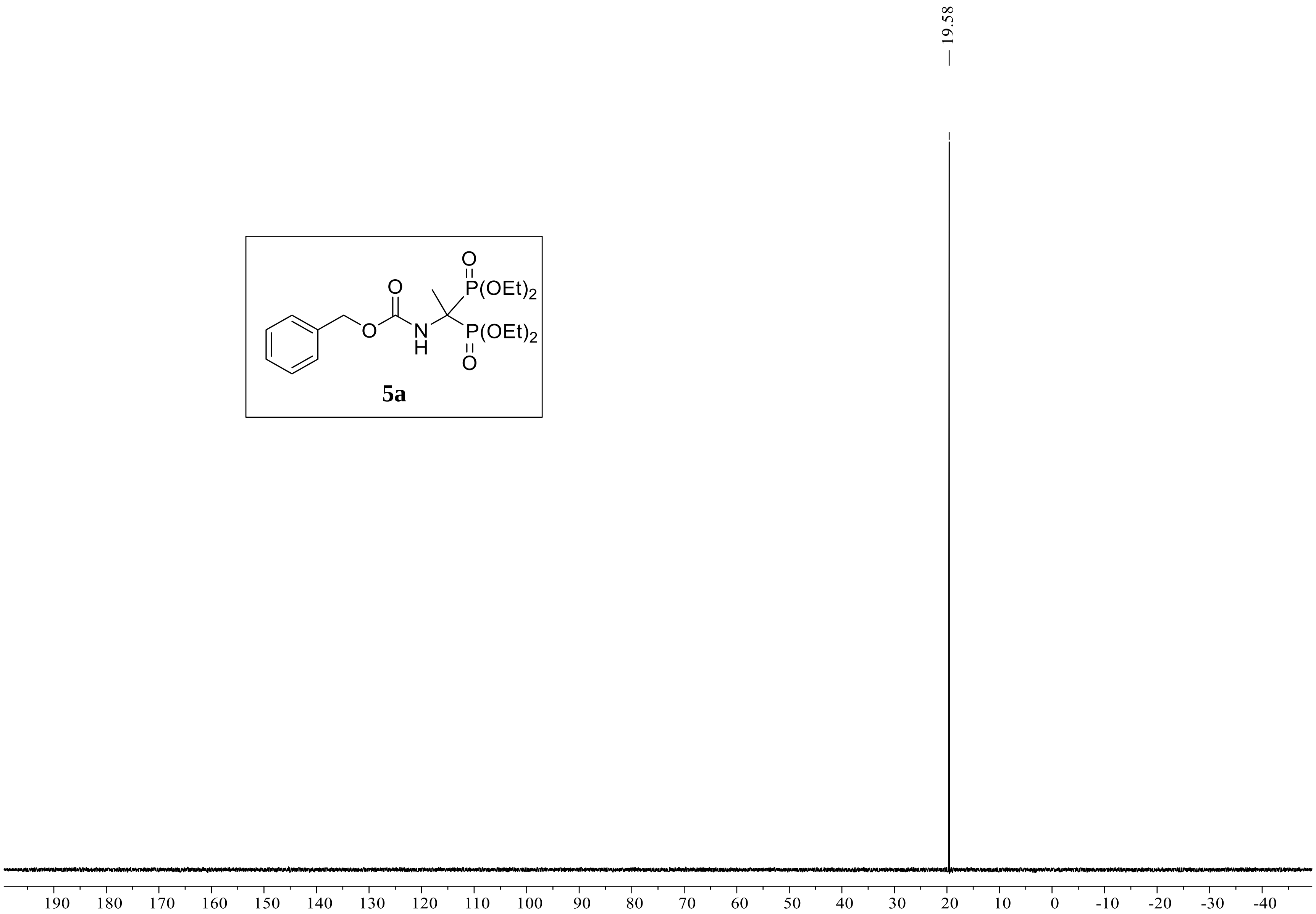

^{31}P NMR spectrum of *tetraethyl 1-(N-benzyloxycarbonylamino)ethylene-1,1-bisphosphonate* (**5a**); 162 MHz/$CDCl_3$/TMS; δ (ppm).

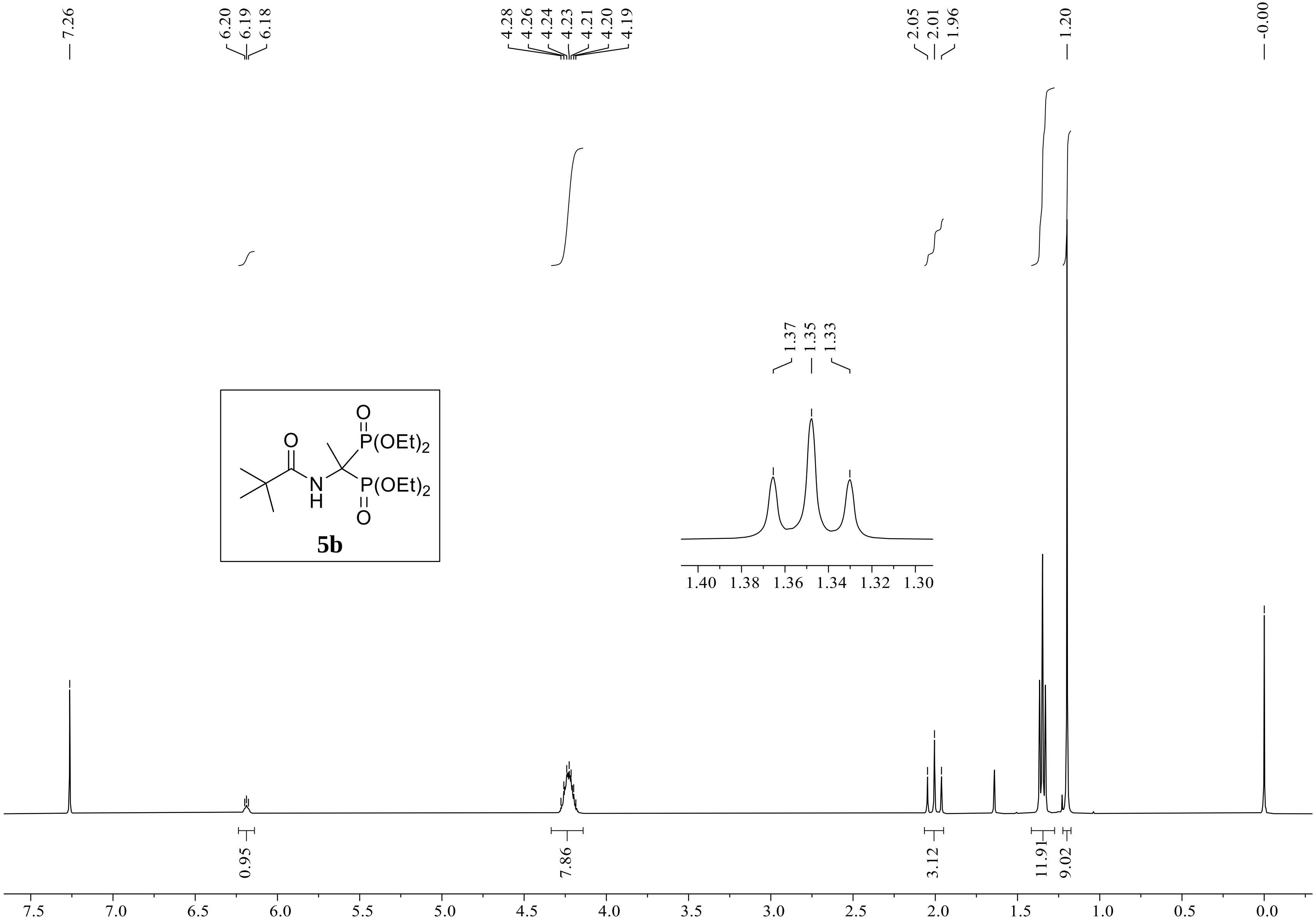

^{1}H NMR spectrum of *tetraethyl 1-(N-pivaloylamino)ethylene-1,1-bisphosphonate* (**5b**); 400 MHz/$CDCl_3$/TMS; δ (ppm).

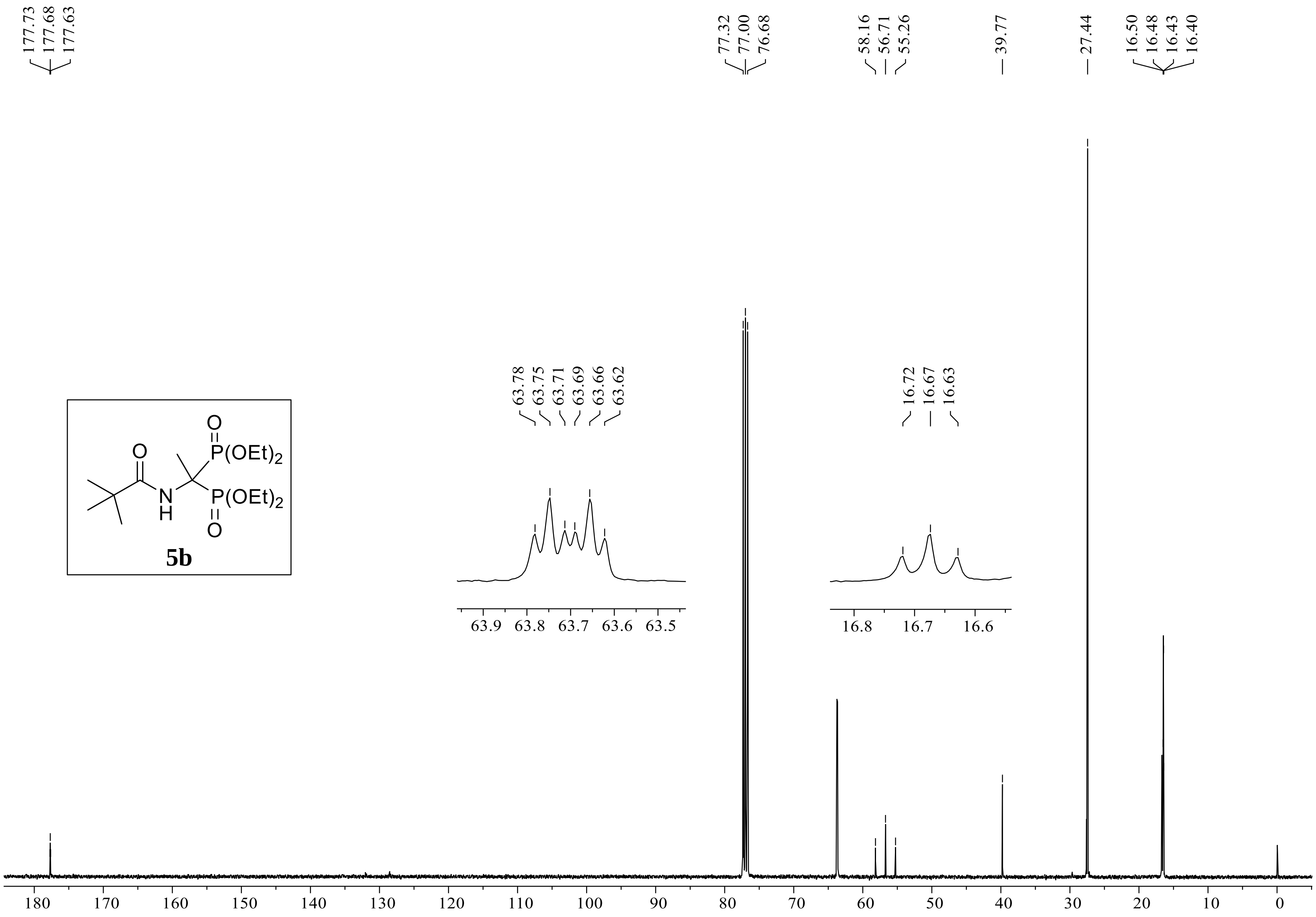

^{13}C NMR spectrum of *tetraethyl 1-(N-pivaloylamino)ethylene-1,1-bisphosphonate* (**5b**); 100 MHz/$CDCl_3$/TMS; δ (ppm).

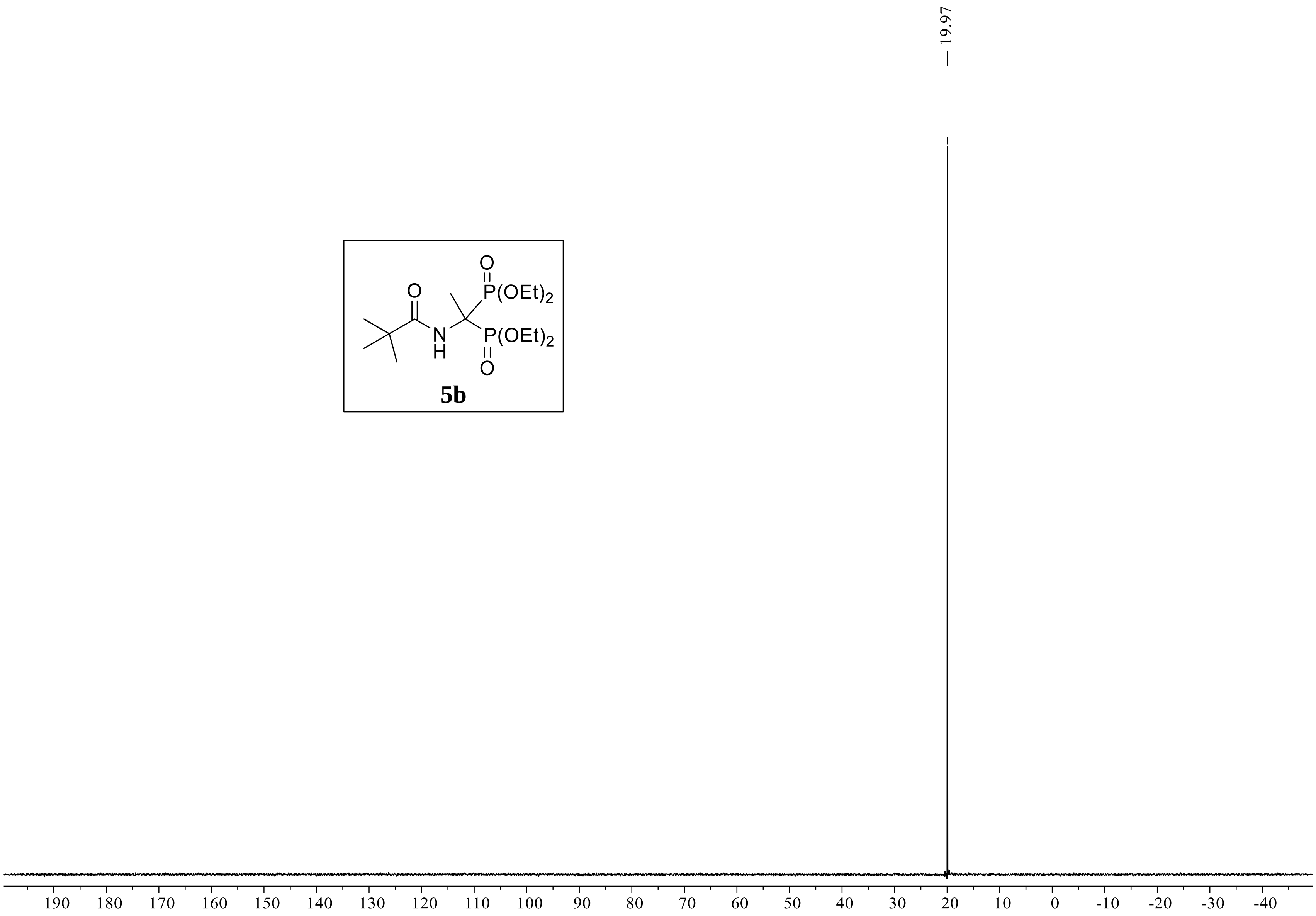

^{31}P NMR spectrum of *tetraethyl 1-(N-pivaloylamino)ethylene-1,1-bisphosphonate* (**5b**); 162 MHz/$CDCl_3$/TMS; δ (ppm).

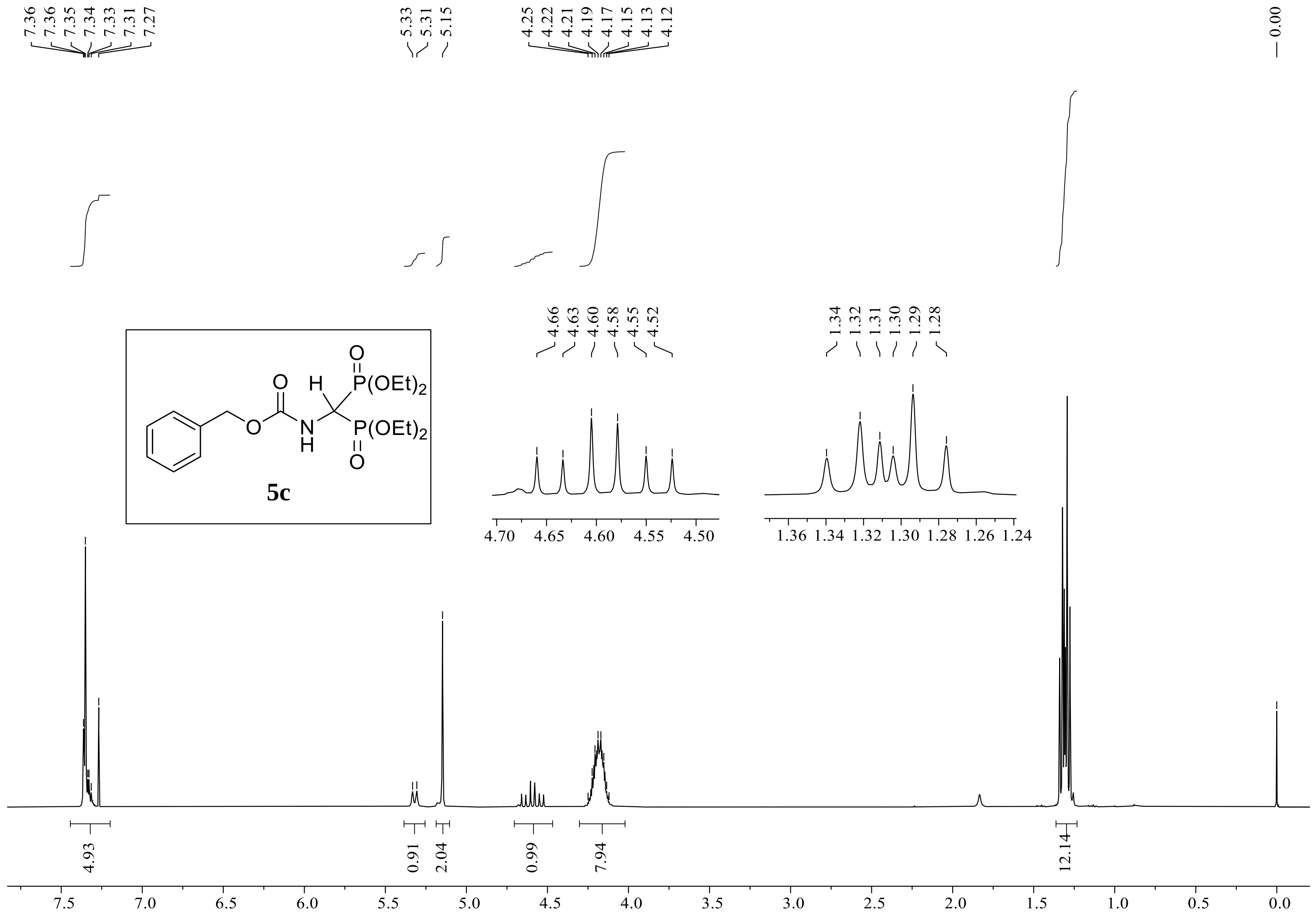

^{1}H NMR spectrum of *tetraethyl 1-(N-benzyloxycarbonylamino)methylene-1,1-bisphosphonate* (**5c**); 400 MHz/$CDCl_3$/TMS; δ (ppm).

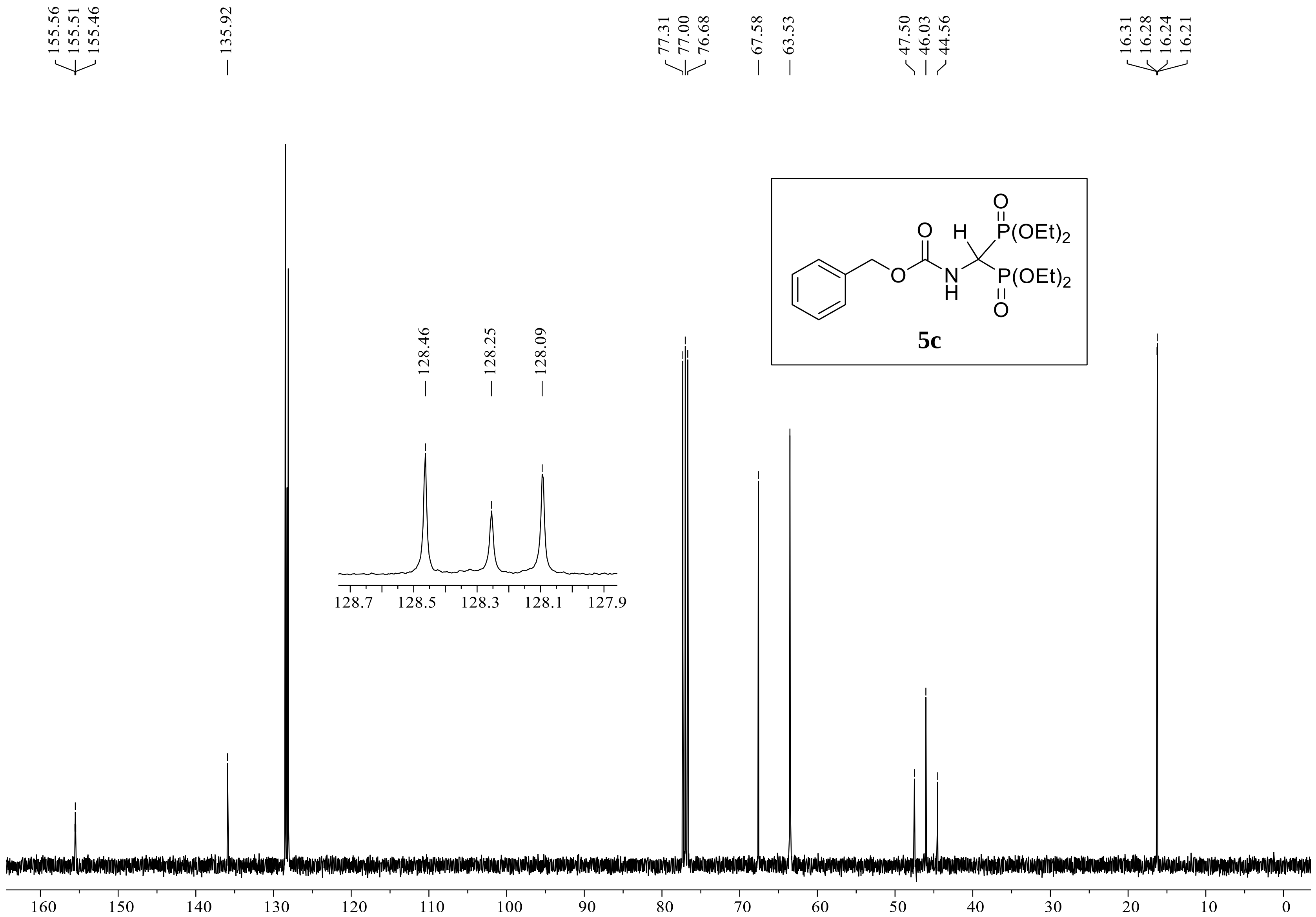

^{13}C NMR spectrum of *tetraethyl 1-(N-benzyloxycarbonylamino)methylene-1,1-bisphosphonate* (**5c**); 100 MHz/$CDCl_3$/TMS; *δ* (ppm).

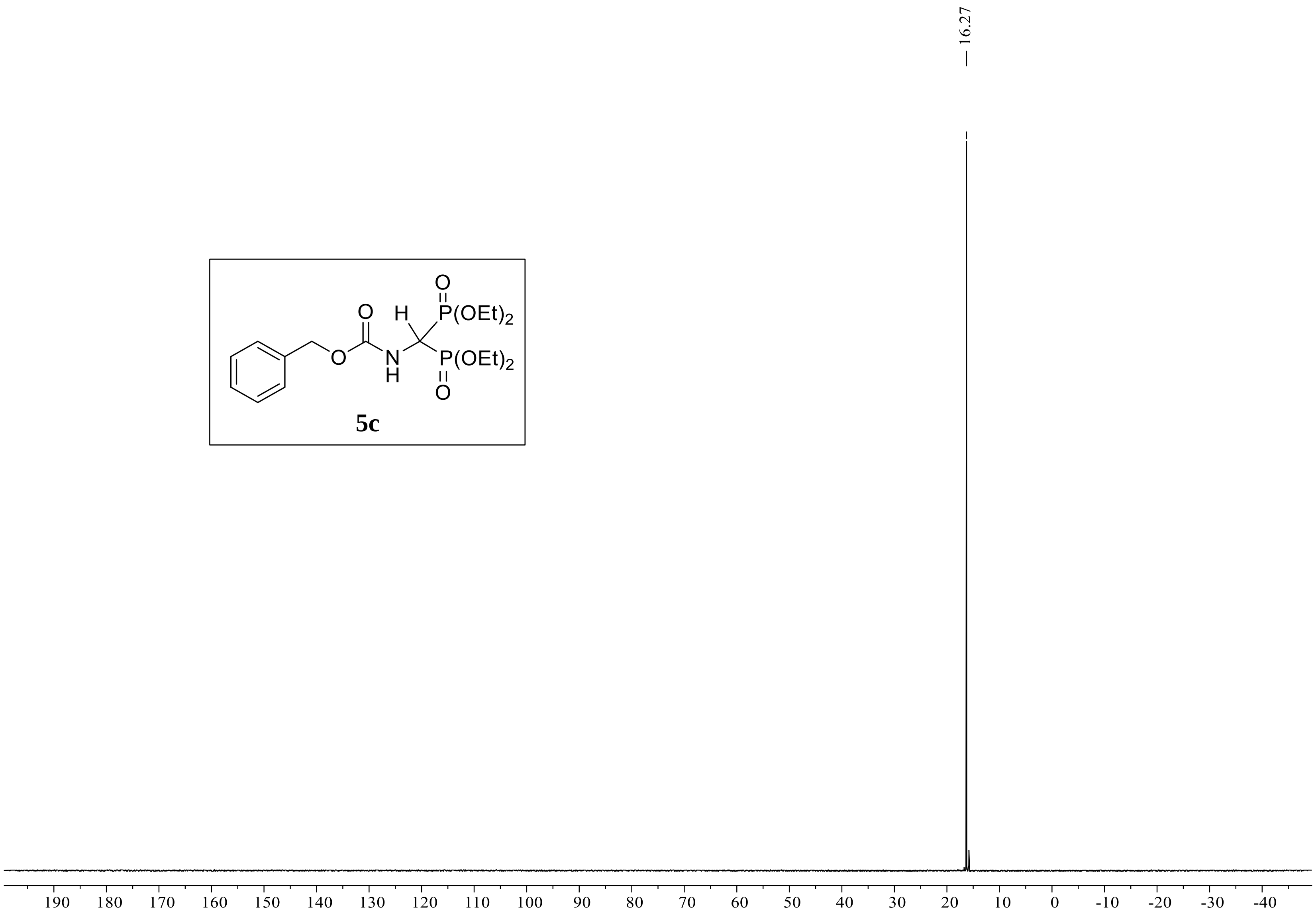

^{31}P NMR spectrum of *tetraethyl 1-(N-benzyloxycarbonylamino)methylene-1,1-bisphosphonate* (**5c**); 162 MHz/$CDCl_3$/TMS; δ (ppm).

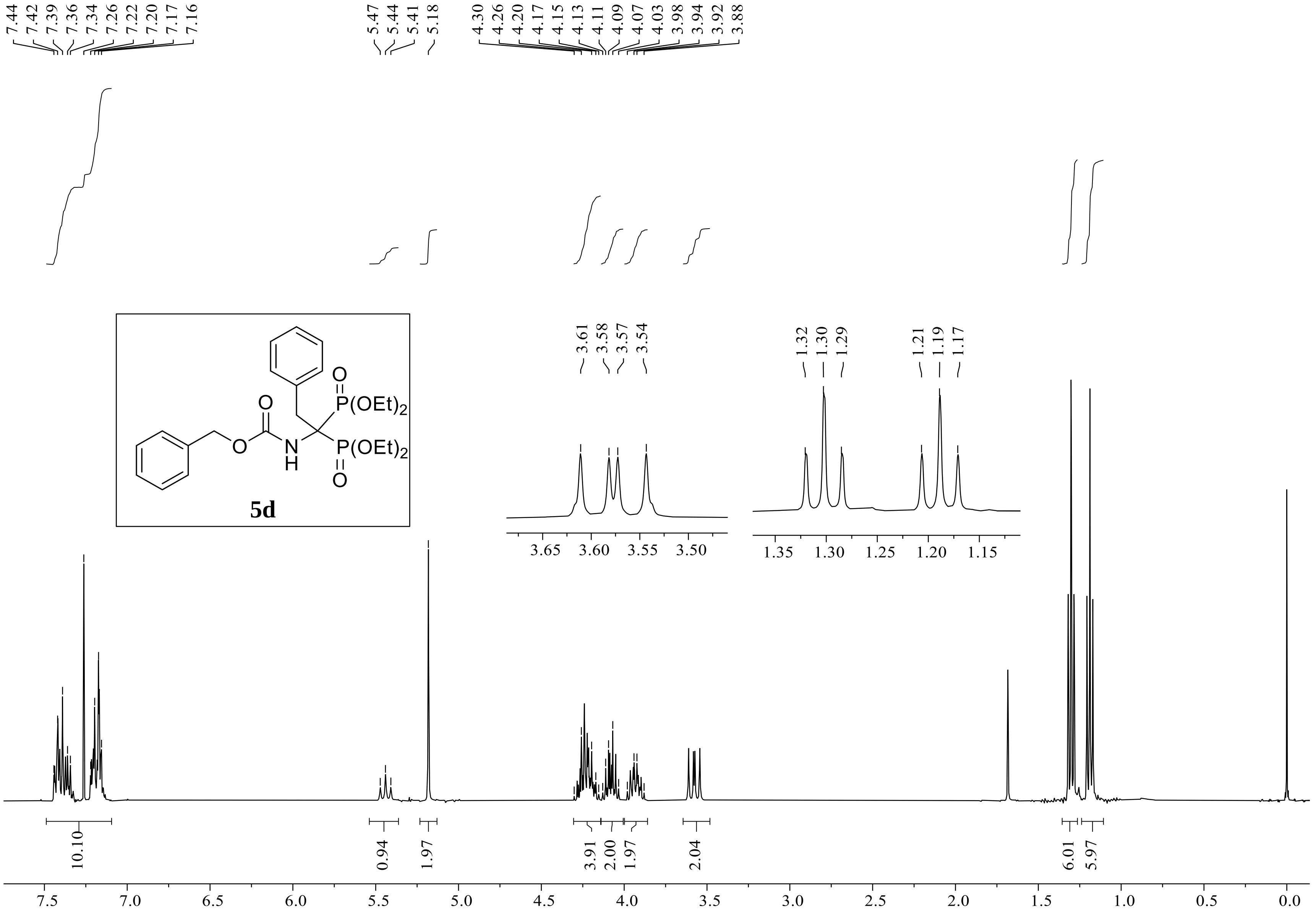

^{1}H NMR spectrum of *tetraethyl 1-(N-benzyloxycarbonylamino)-2-phenylethylene-1,1-bisphosphonate* (**5d**); 400 MHz/$CDCl_3$/TMS; δ (ppm).

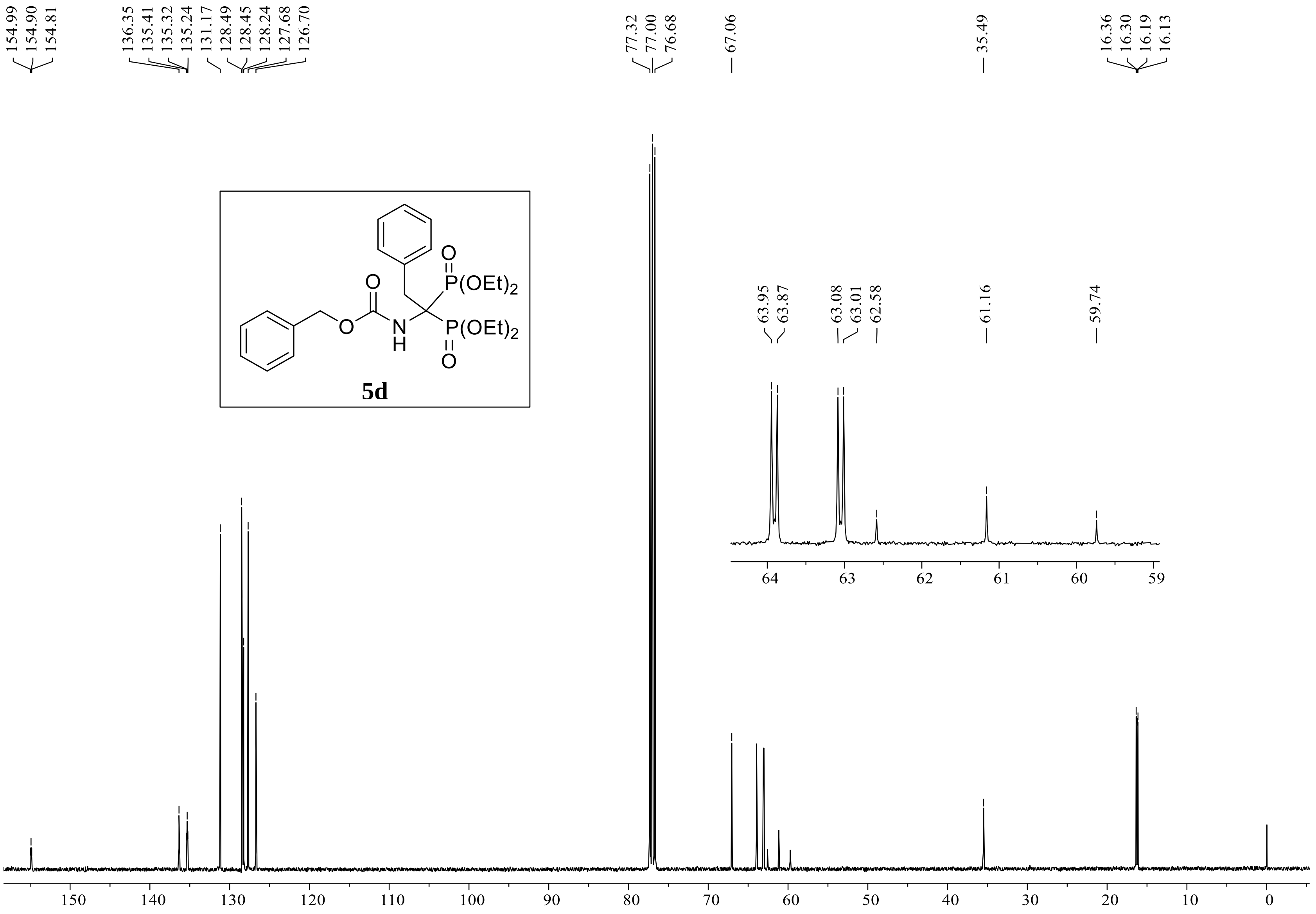

^{13}C NMR spectrum of *tetraethyl 1-(N-benzyloxycarbonylamino)-2-phenylethylene-1,1-bisphosphonate* (**5d**); 100 MHz/$CDCl_3$/TMS; δ (ppm).

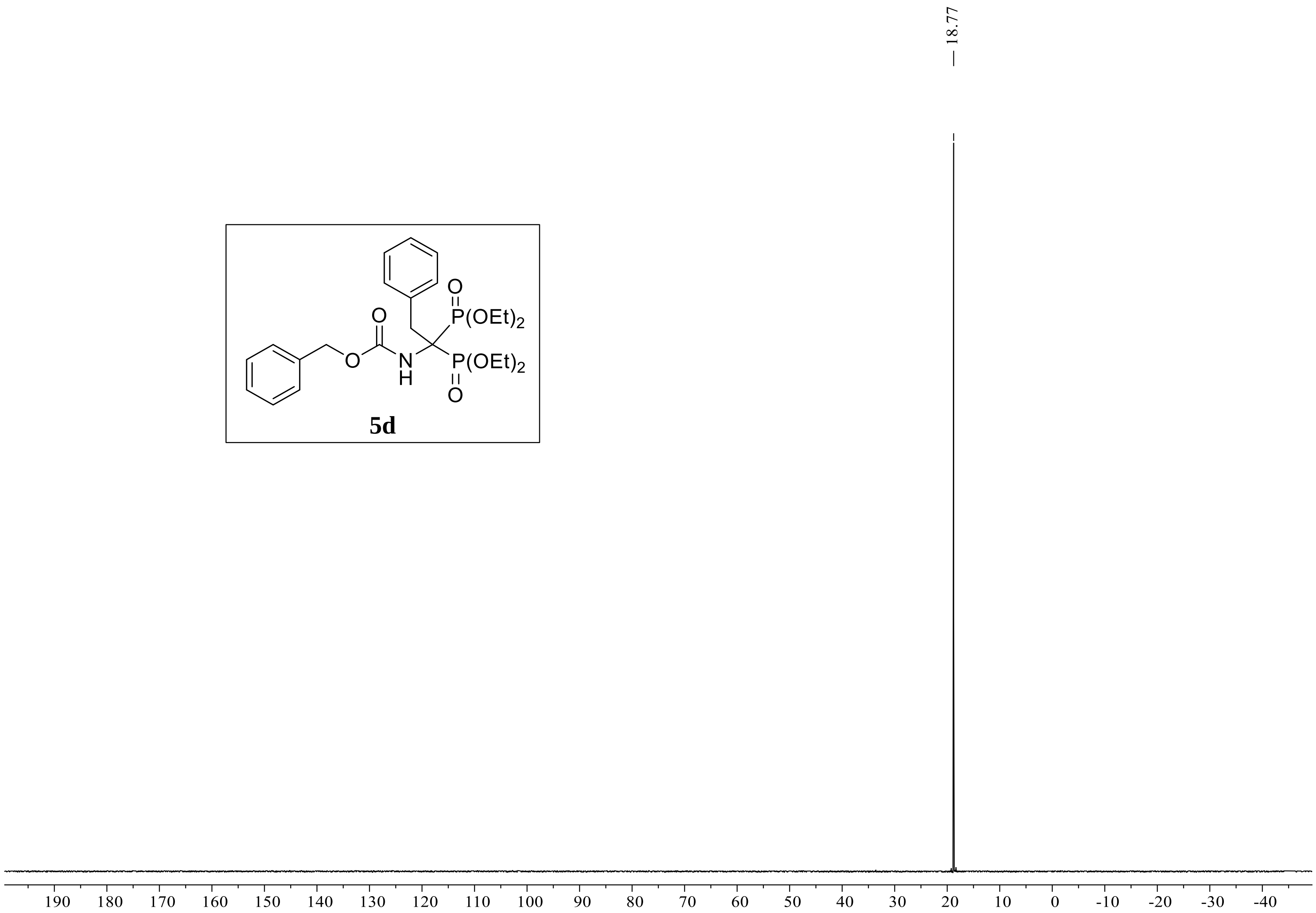

^{31}P NMR spectrum of *tetraethyl 1-(N-benzyloxycarbonylamino)-2-phenylethylene-1,1-bisphosphonate* (**5d**); 162 MHz/$CDCl_3$/TMS; δ (ppm).

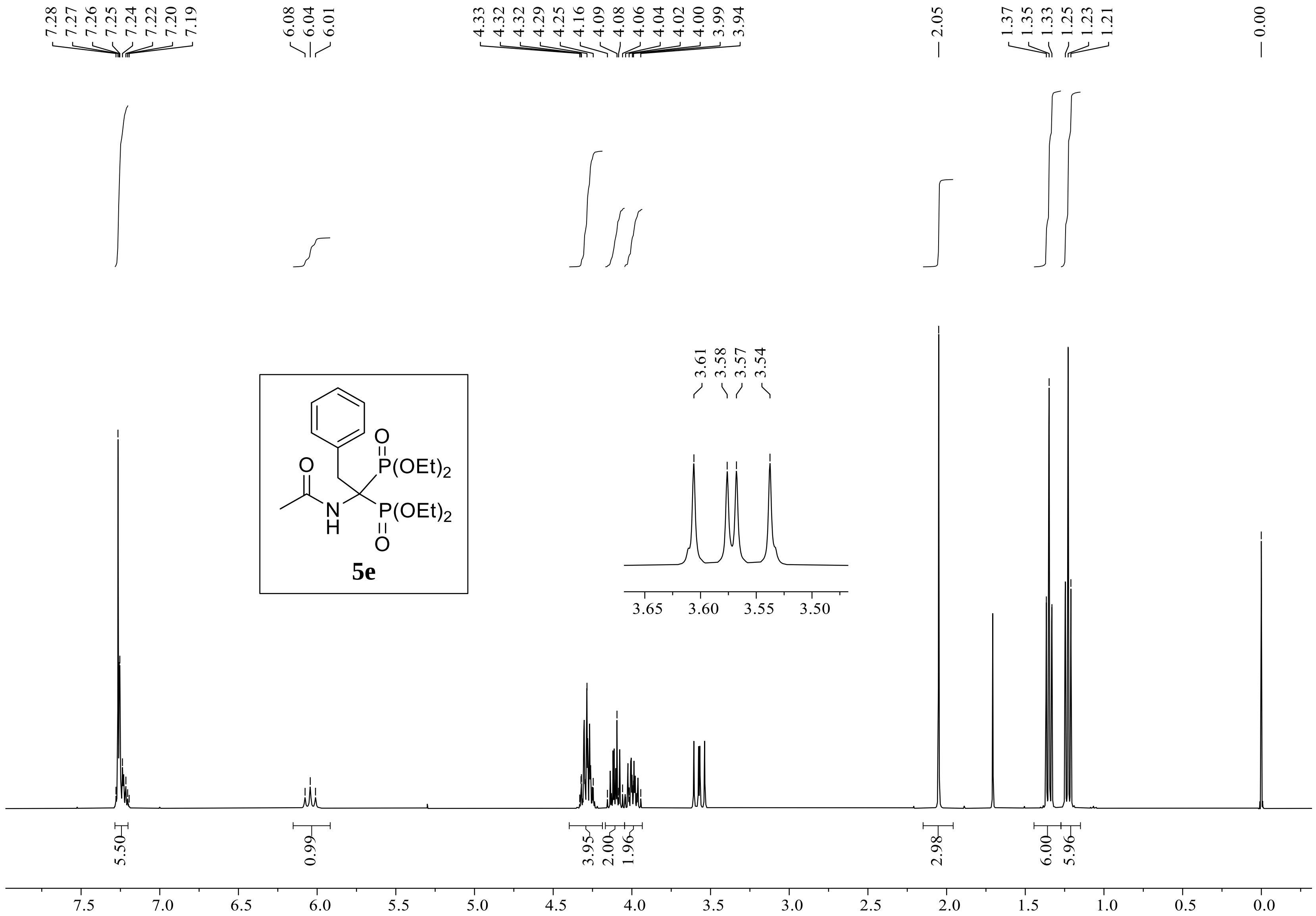

^{1}H NMR spectrum of *tetraethyl 1-(N-acetylamino)-2-phenylethylene-1,1-bisphosphonate* (**5e**); 400 MHz/$CDCl_3$/TMS; δ (ppm).

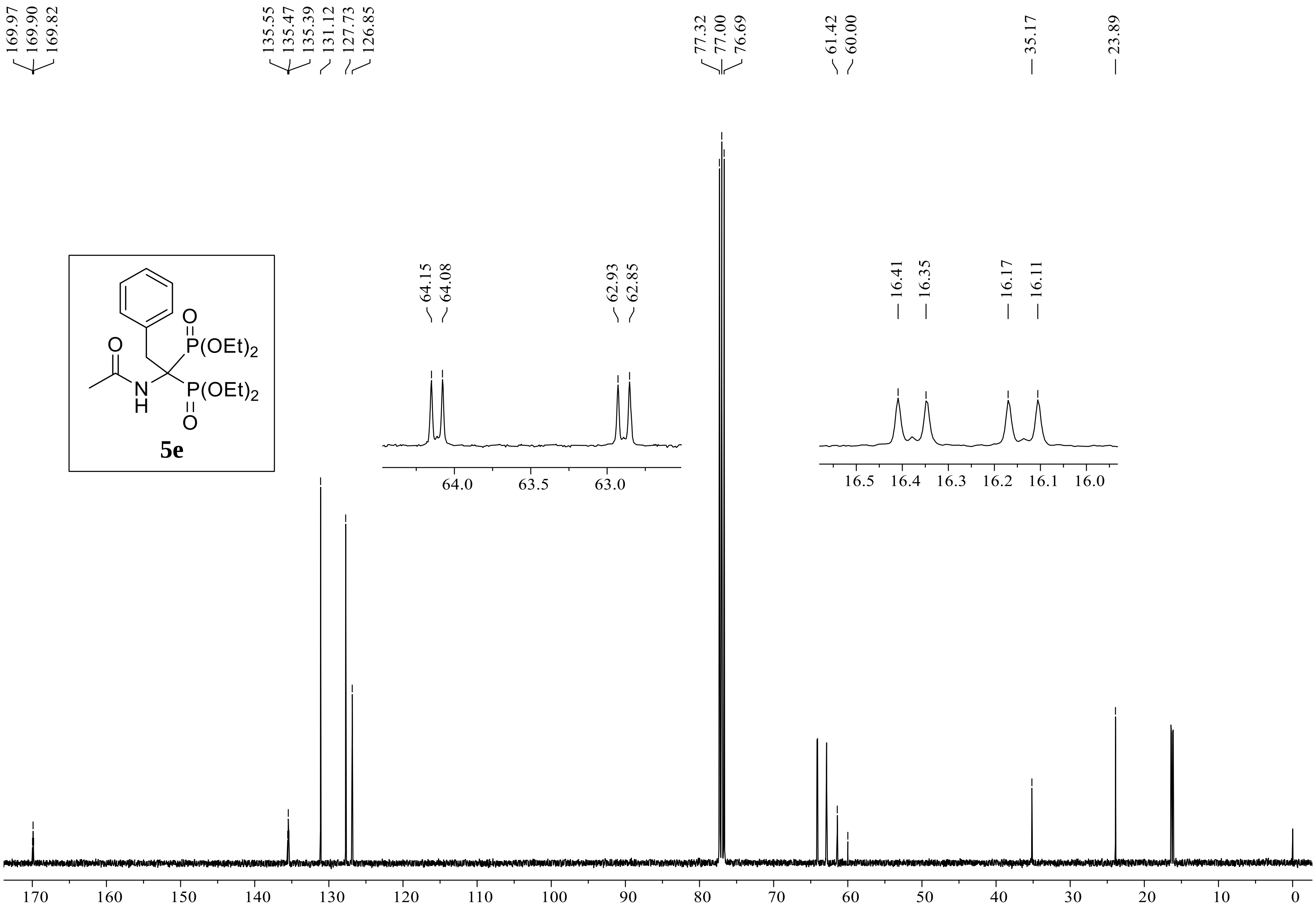

^{13}C NMR spectrum of *tetraethyl 1-(N-acetylamino)-2-phenylethylene-1,1-bisphosphonate* (**5e**); 100 MHz/$CDCl_3$/TMS; δ (ppm).

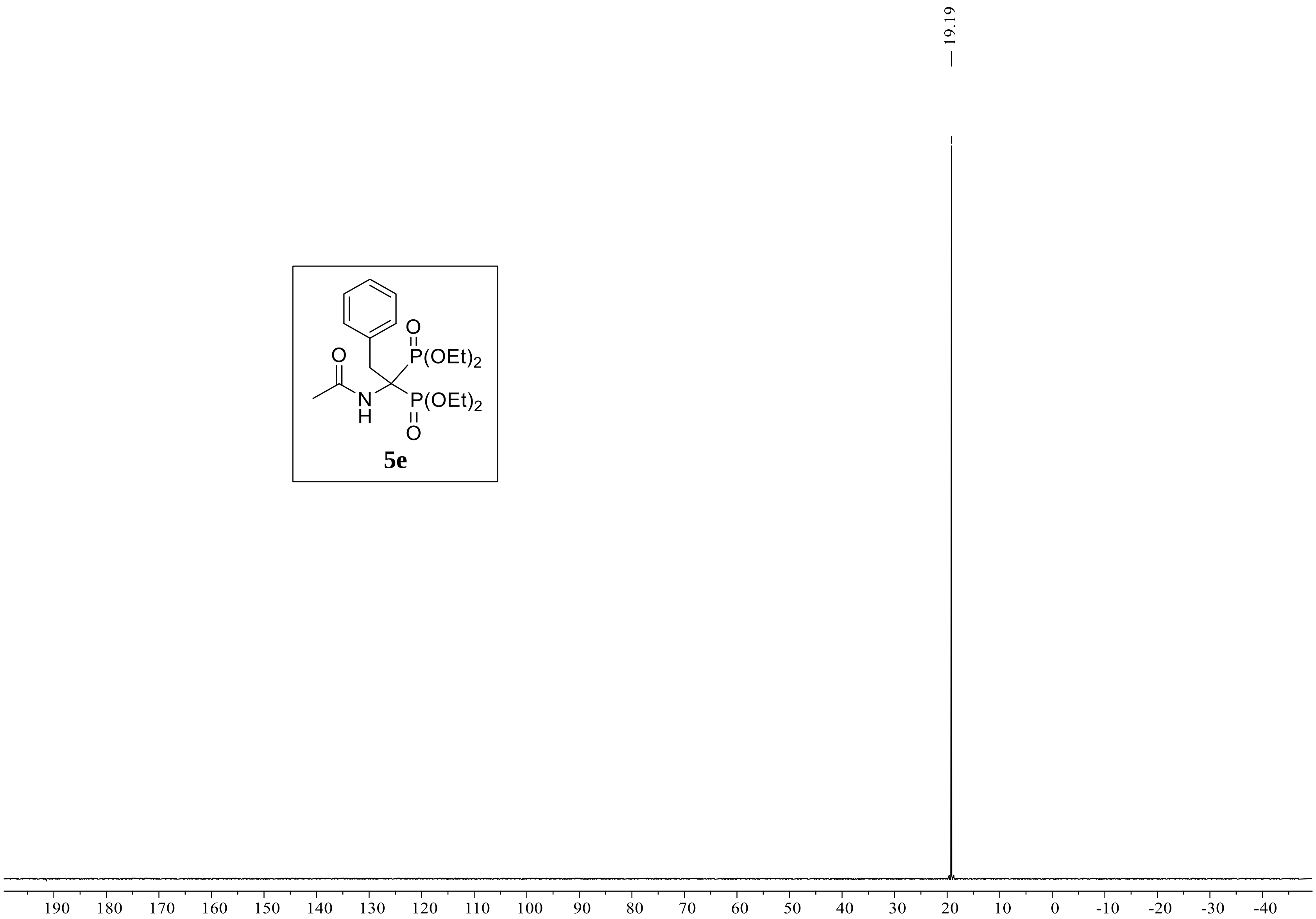

^{31}P NMR spectrum of *tetraethyl 1-(N-acetylamino)-2-phenylethylene-1,1-bisphosphonate* (**5e**); 162 MHz/$CDCl_3$/TMS; *δ* (ppm).

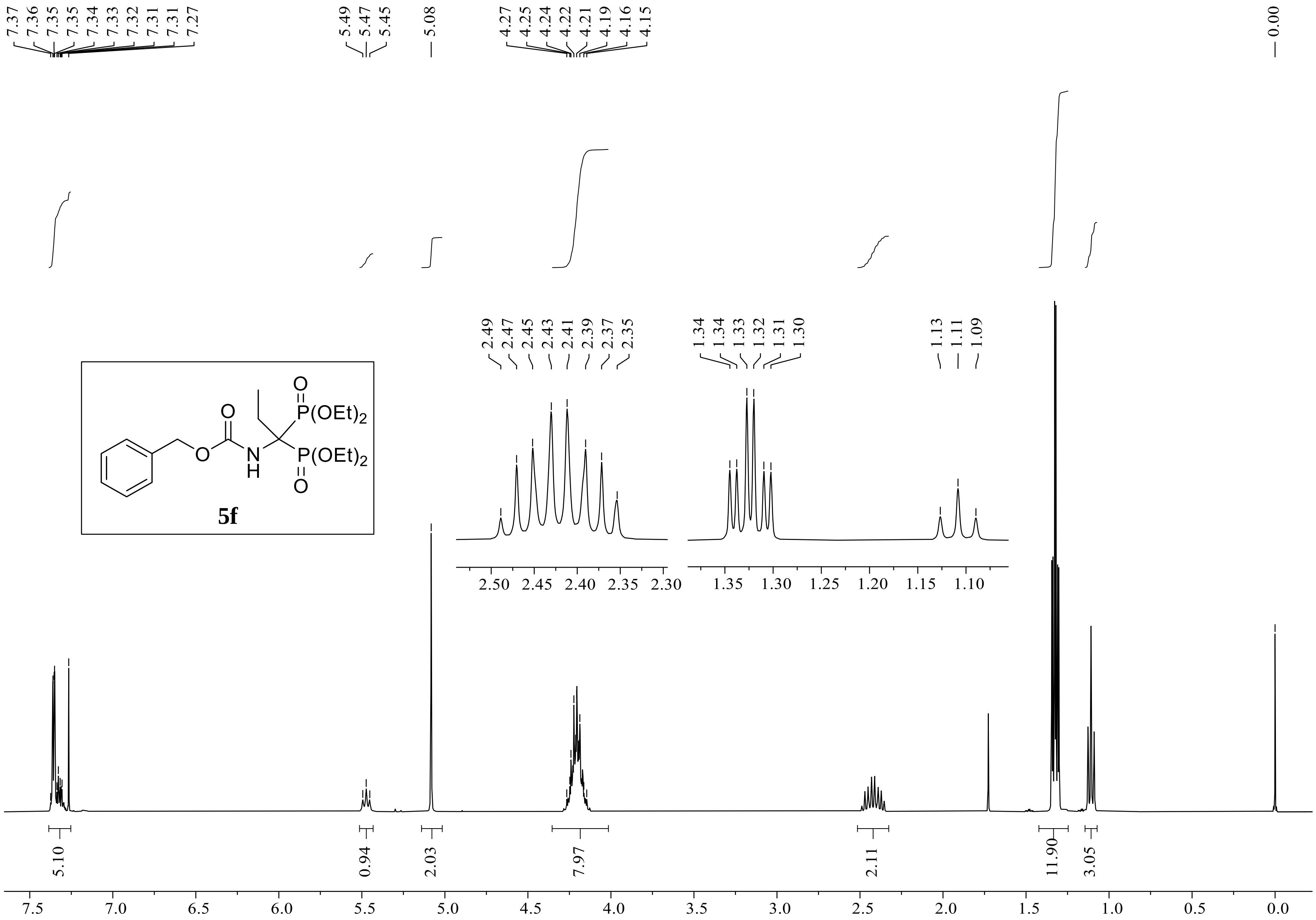

^{1}H NMR spectrum of *tetraethyl 1-(N-benzyloxycarbonylamino)propylene-1,1-bisphosphonate* (**5f**); 400 MHz/$CDCl_3$/TMS; δ (ppm).

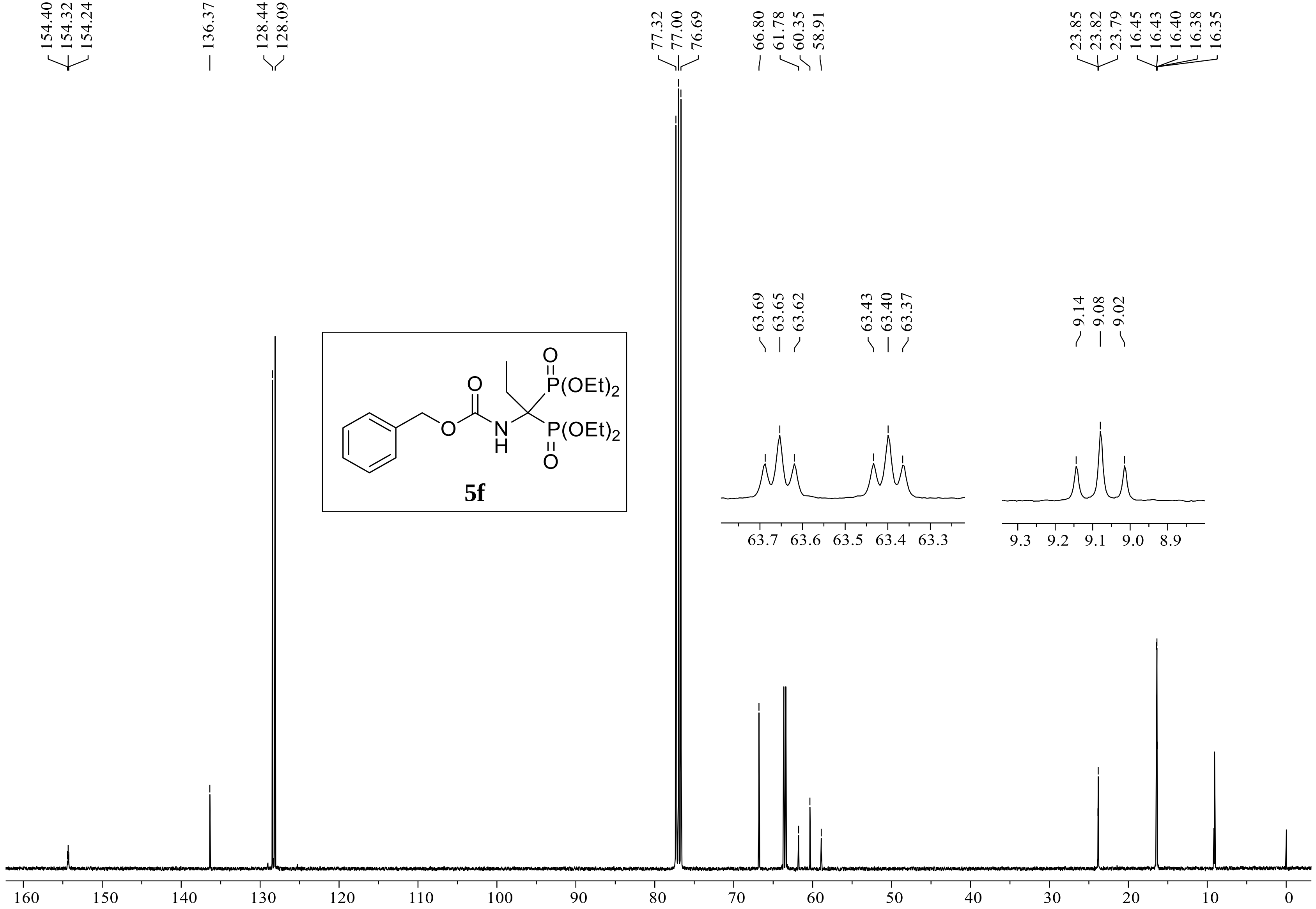

^{13}C NMR spectrum of *tetraethyl 1-(N-benzyloxycarbonylamino)propylene-1,1-bisphosphonate* (**5f**); 100 MHz/$CDCl_3$/TMS; δ (ppm).

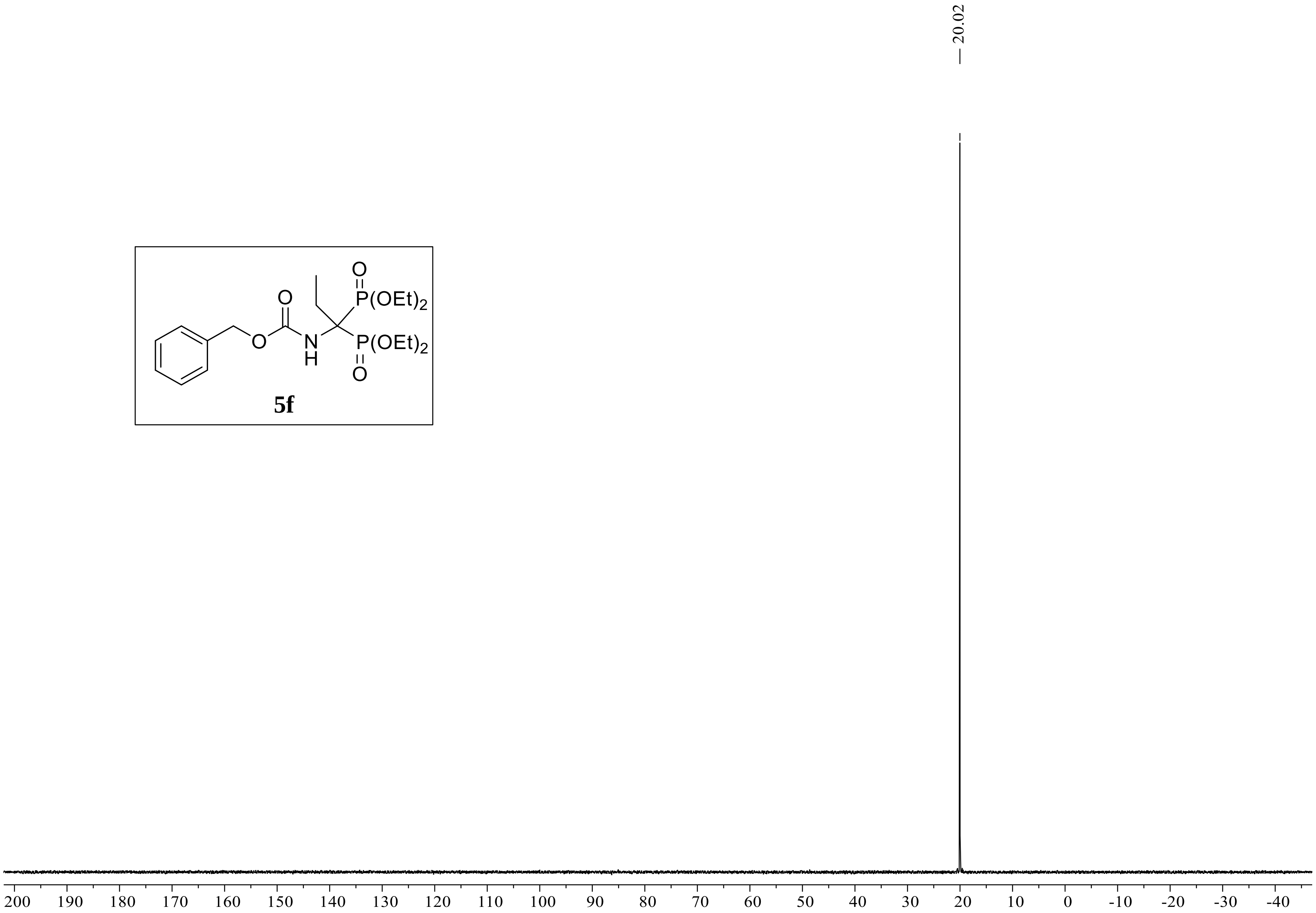

^{31}P NMR spectrum of *tetraethyl 1-(N-benzyloxycarbonylamino)propylene-1,1-bisphosphonate* (**5f**); 162 MHz/$CDCl_3$/TMS; δ (ppm).

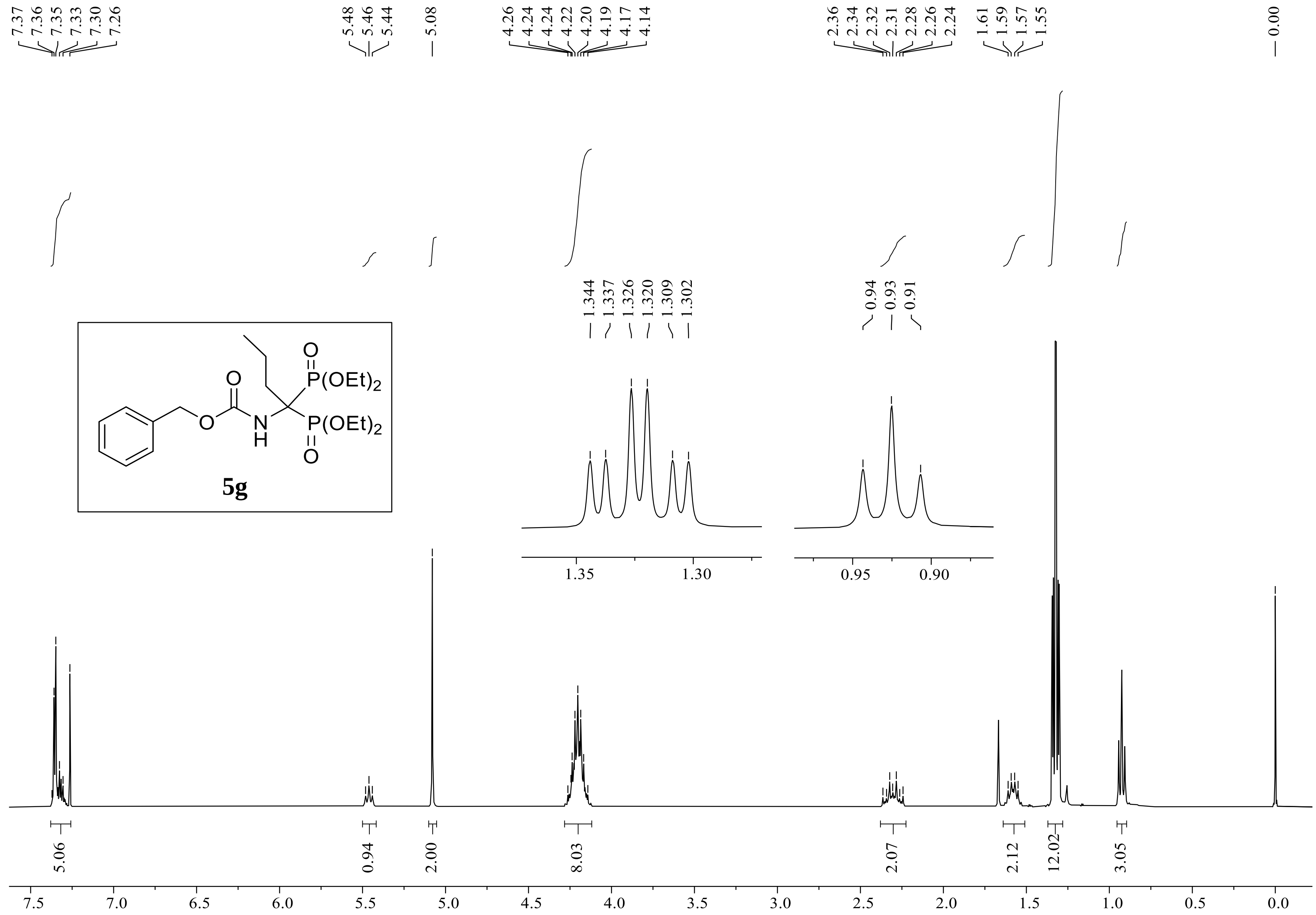

^{1}H NMR spectrum of *tetraethyl 1-(N-benzyloxycarbonylamino)butylene-1,1-bisphosphonate* (**5g**); 400 MHz/$CDCl_3$/TMS; δ (ppm).

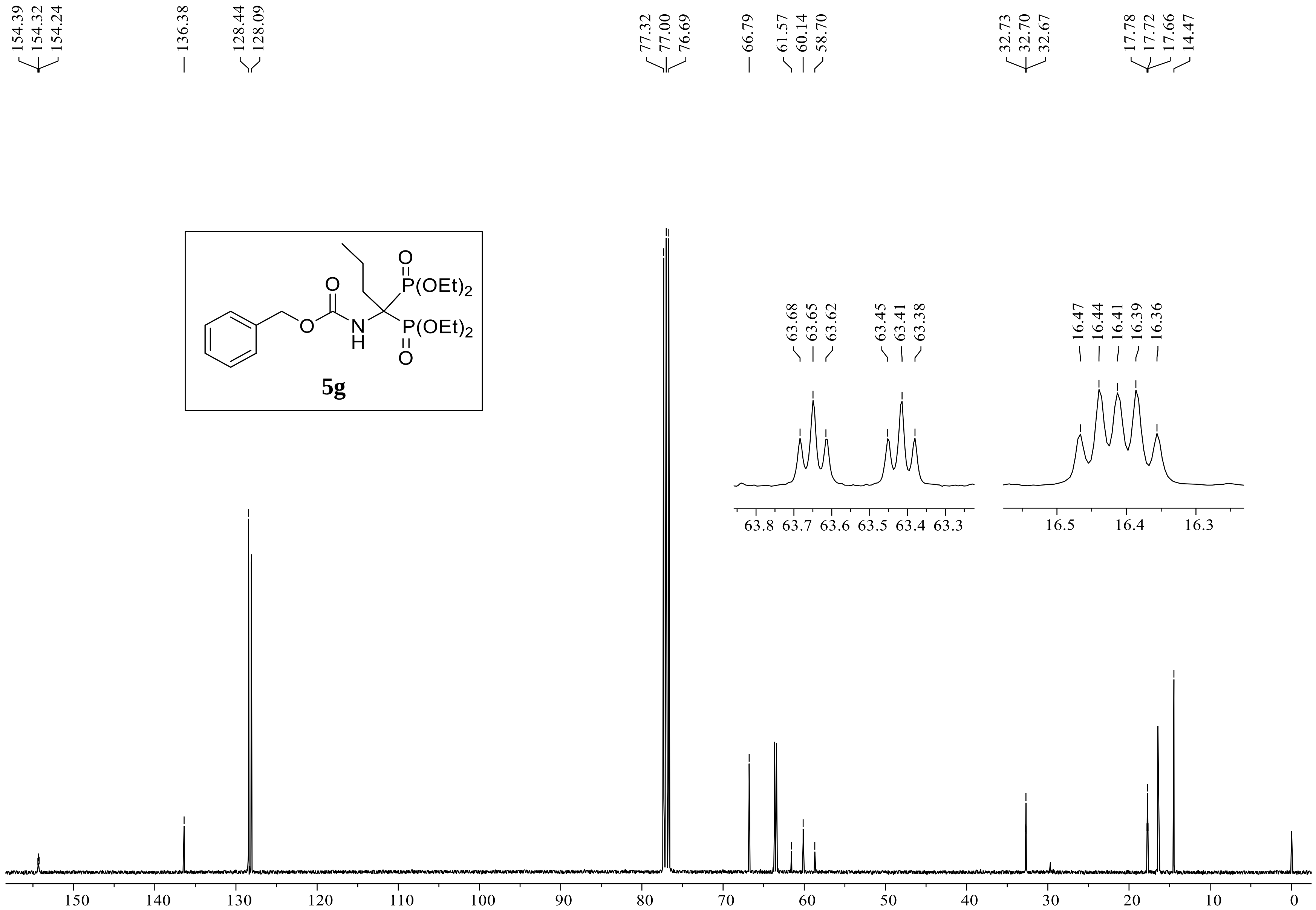

^{13}C NMR spectrum of *tetraethyl 1-(N-benzyloxycarbonylamino)butylene-1,1-bisphosphonate* (**5g**); 100 MHz/$CDCl_3$/TMS; δ (ppm).

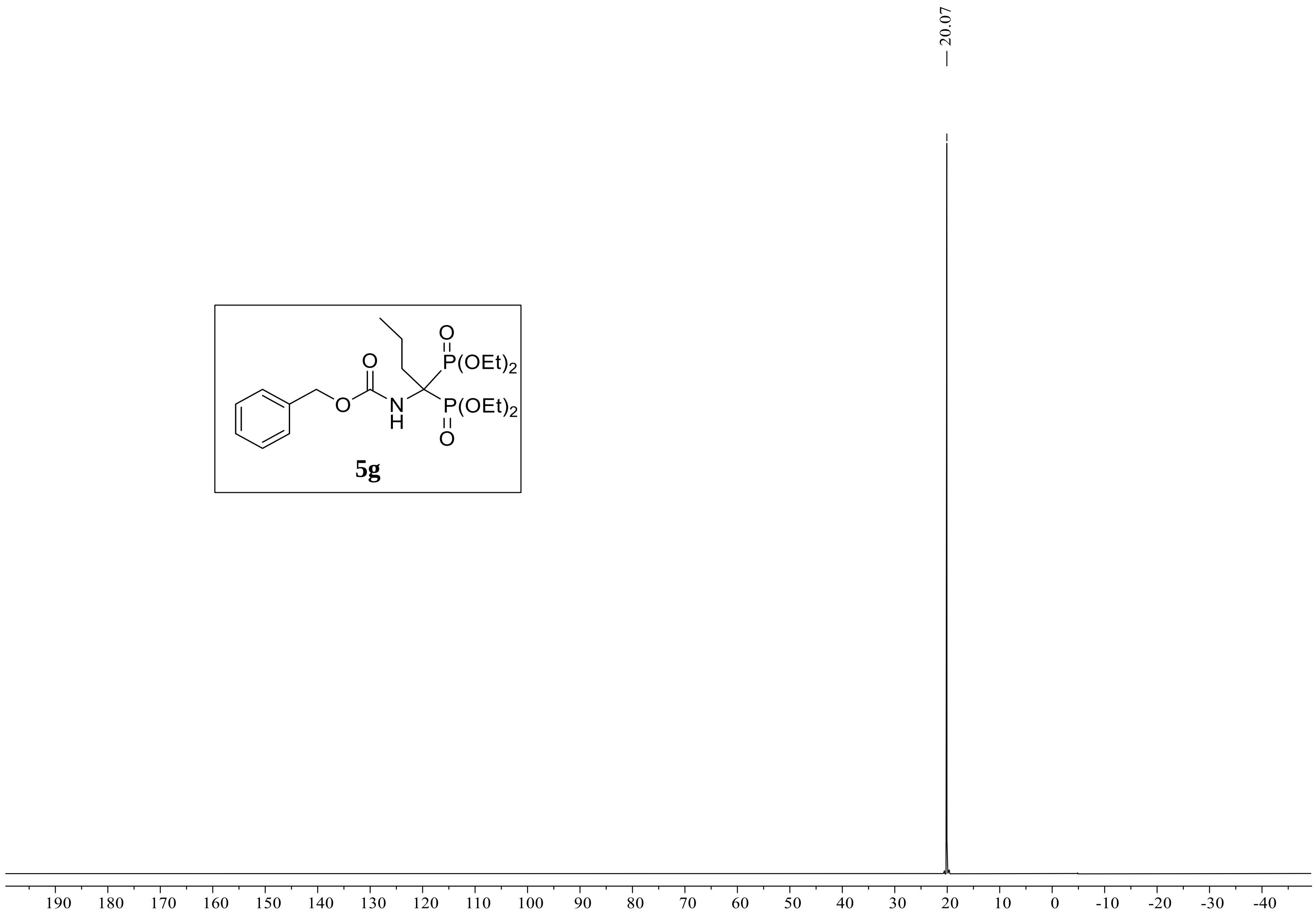

^{31}P NMR spectrum of *tetraethyl 1-(N-benzyloxycarbonylamino)butylene-1,1-bisphosphonate* (**5g**); 162 MHz/$CDCl_3$/TMS; δ (ppm).

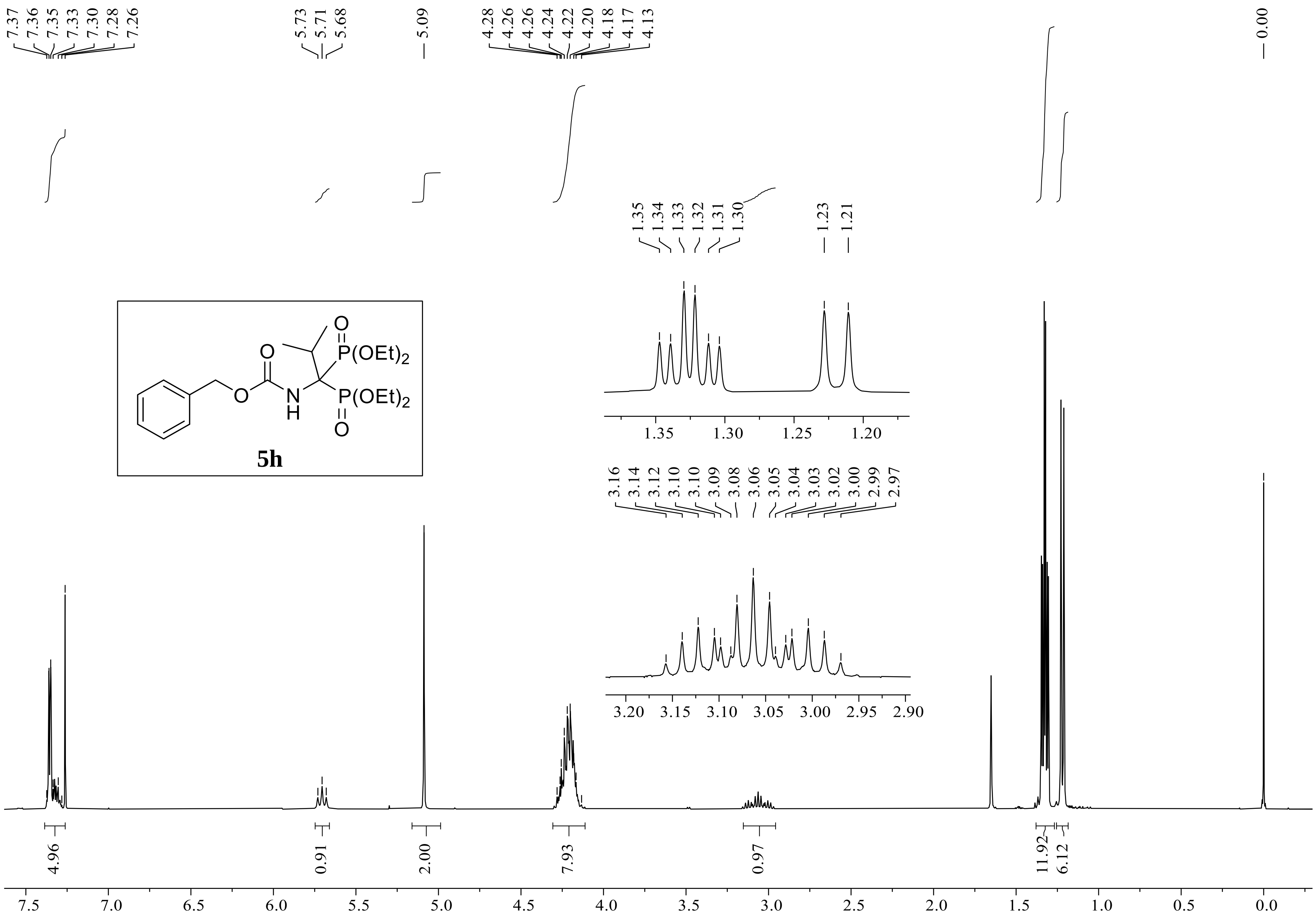

^{1}H NMR spectrum of *tetraethyl 1-(N-benzyloxycarbonylamino)-2-methylpropylene-1,1-bisphosphonate* (**5h**); 400 MHz/$CDCl_3$/TMS; δ (ppm).

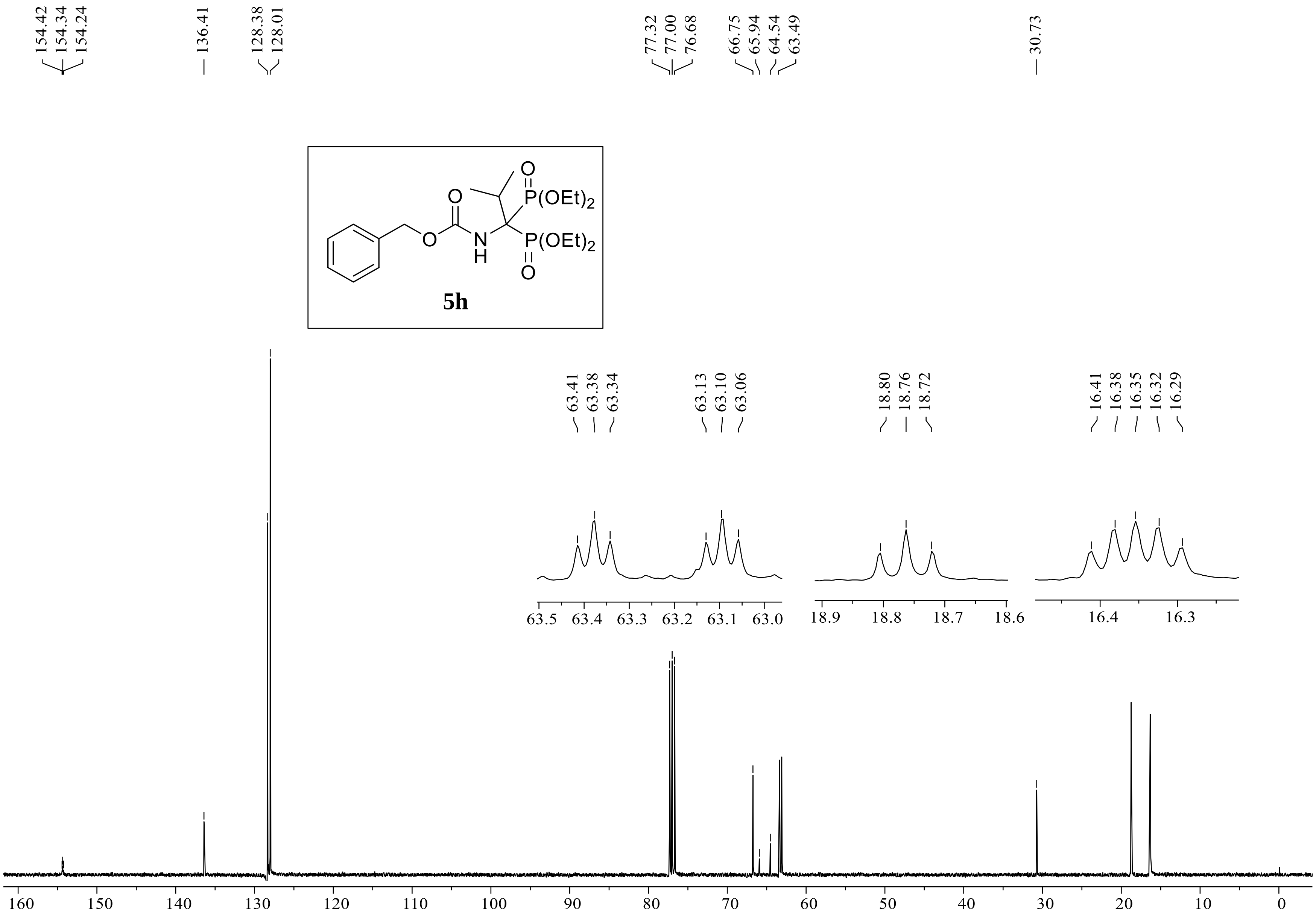

^{13}C NMR spectrum of *tetraethyl 1-(N-benzyloxycarbonylamino)-2-methylpropylene-1,1-bisphosphonate* (**5h**); 100 MHz/$CDCl_3$/TMS; δ (ppm).

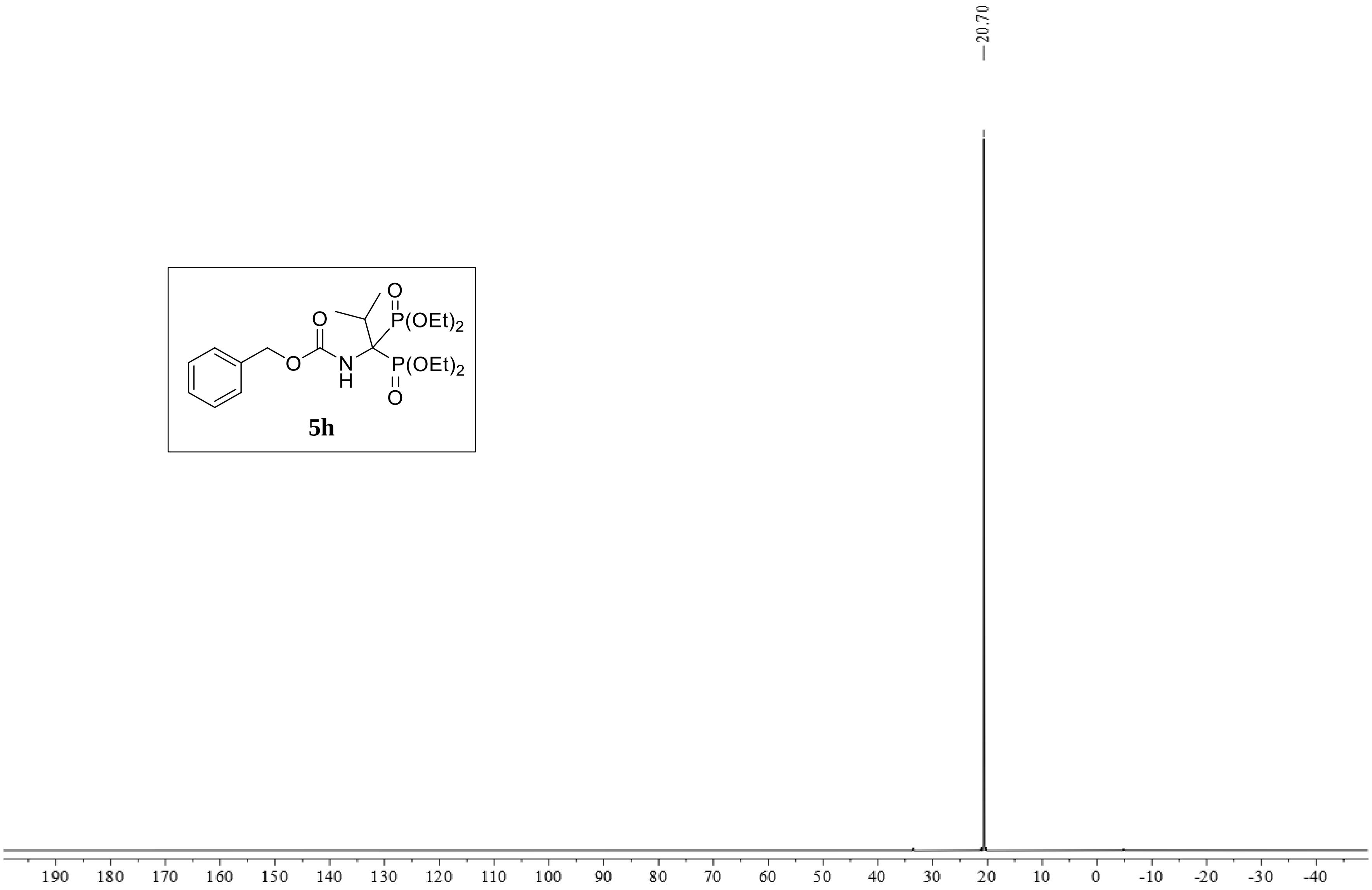

^{31}P NMR spectrum of *tetraethyl 1-(N-benzyloxycarbonylamino)-2-methylpropylene-1,1-bisphosphonate* (**5h**); 162 MHz/$CDCl_3$/TMS; δ (ppm).

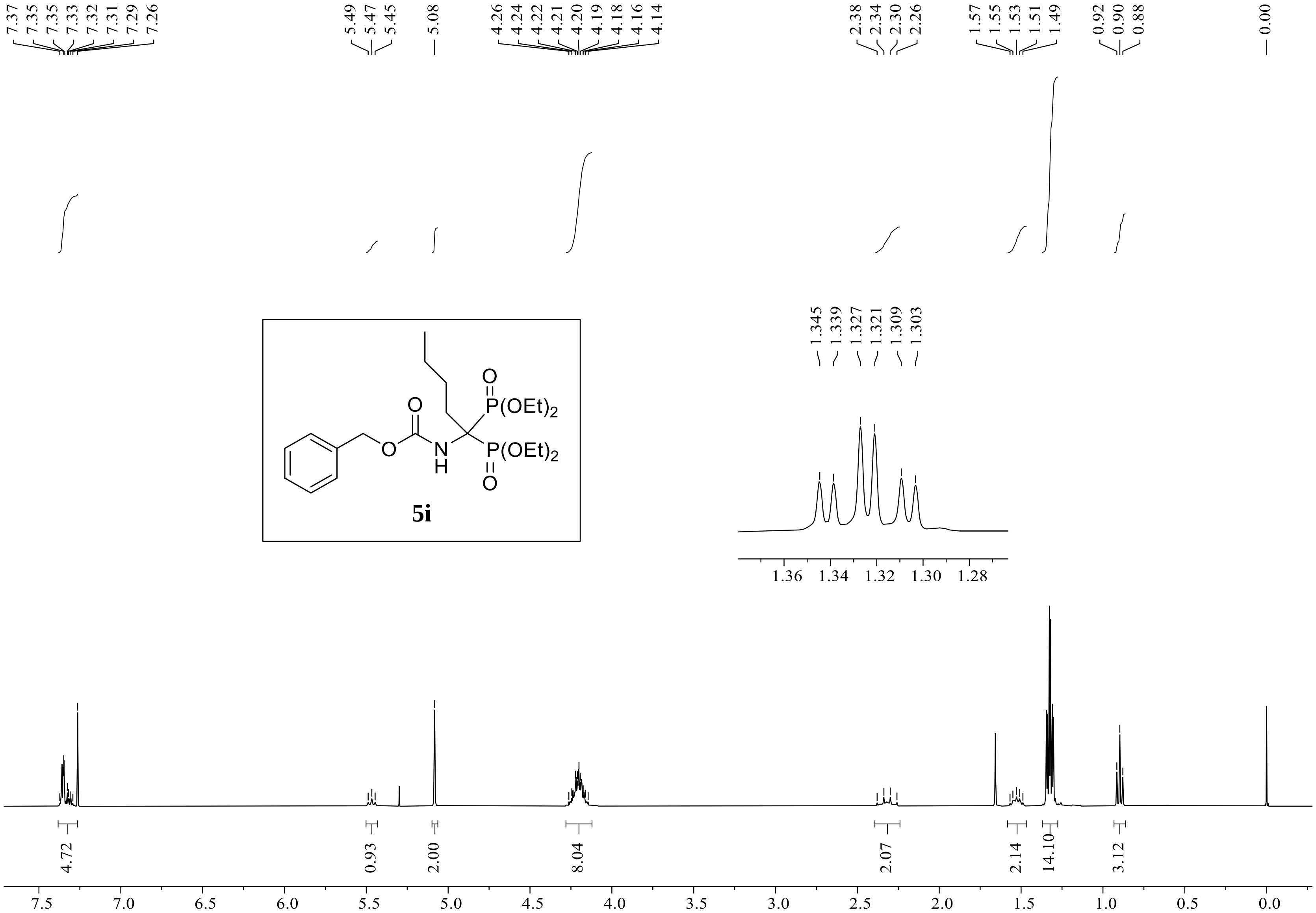

^{1}H NMR spectrum of *tetraethyl 1-(N-benzyloxycarbonylamino)pentylene-1,1-bisphosphonate* (**5i**); 400 MHz/$CDCl_3$/TMS; δ (ppm).

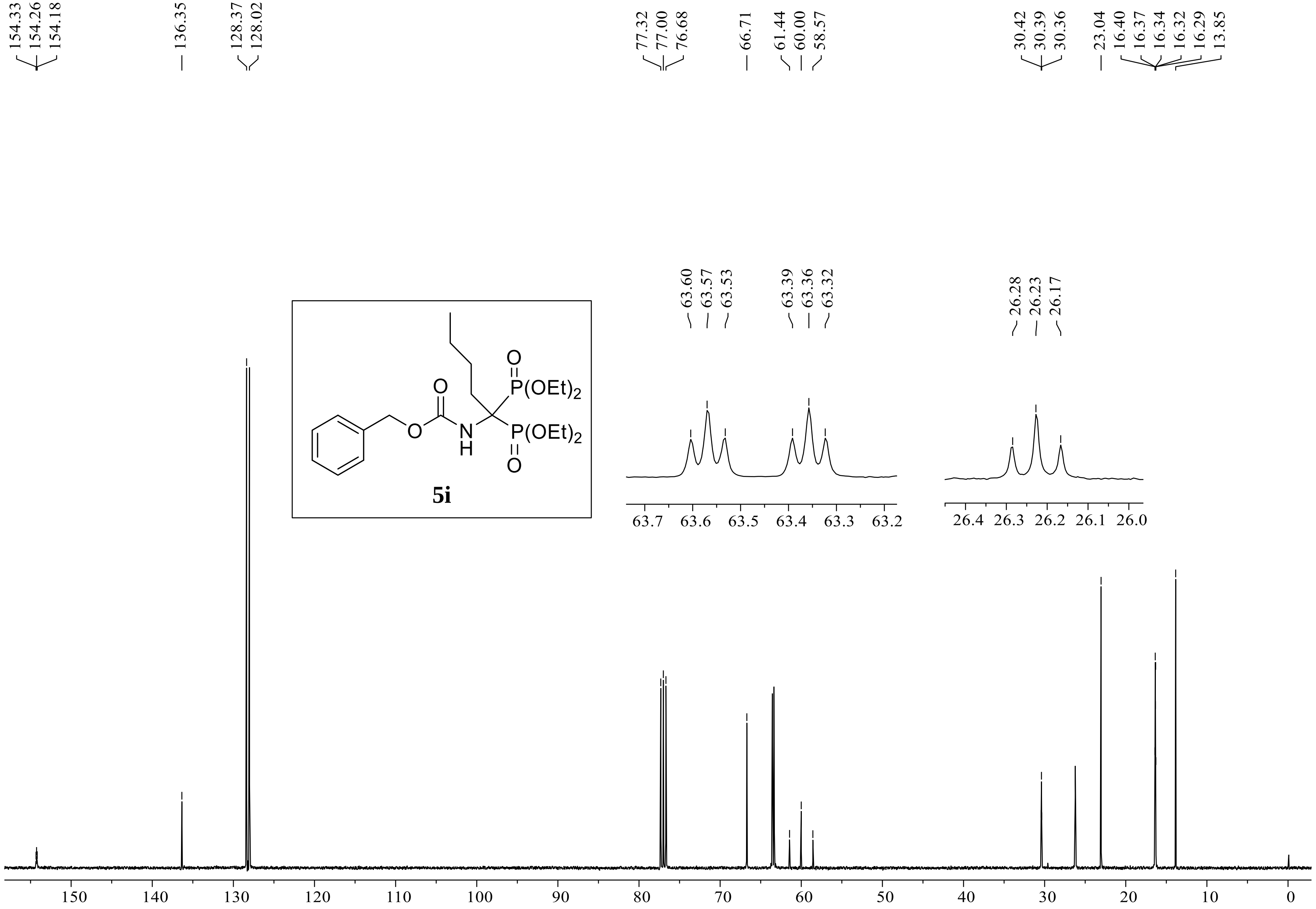

^{13}C NMR spectrum of *tetraethyl 1-(N-benzyloxycarbonylamino)pentylene-1,1-bisphosphonate* (**5i**); 100 MHz/$CDCl_3$/TMS; δ (ppm).

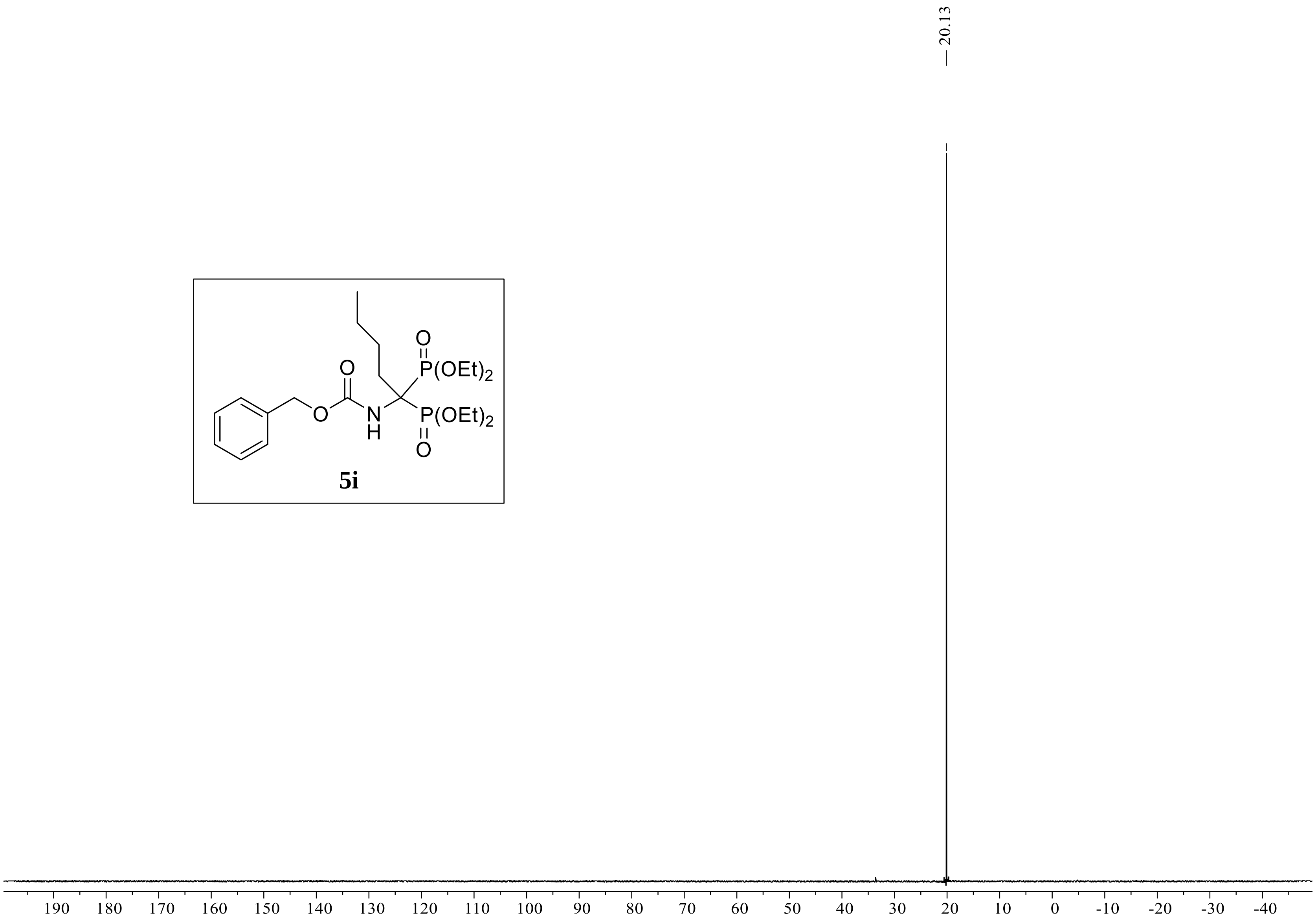

^{31}P NMR spectrum of *tetraethyl 1-(N-benzyloxycarbonylamino)pentylene-1,1-bisphosphonate* (**5i**); 162 MHz/$CDCl_3$/TMS; δ (ppm).

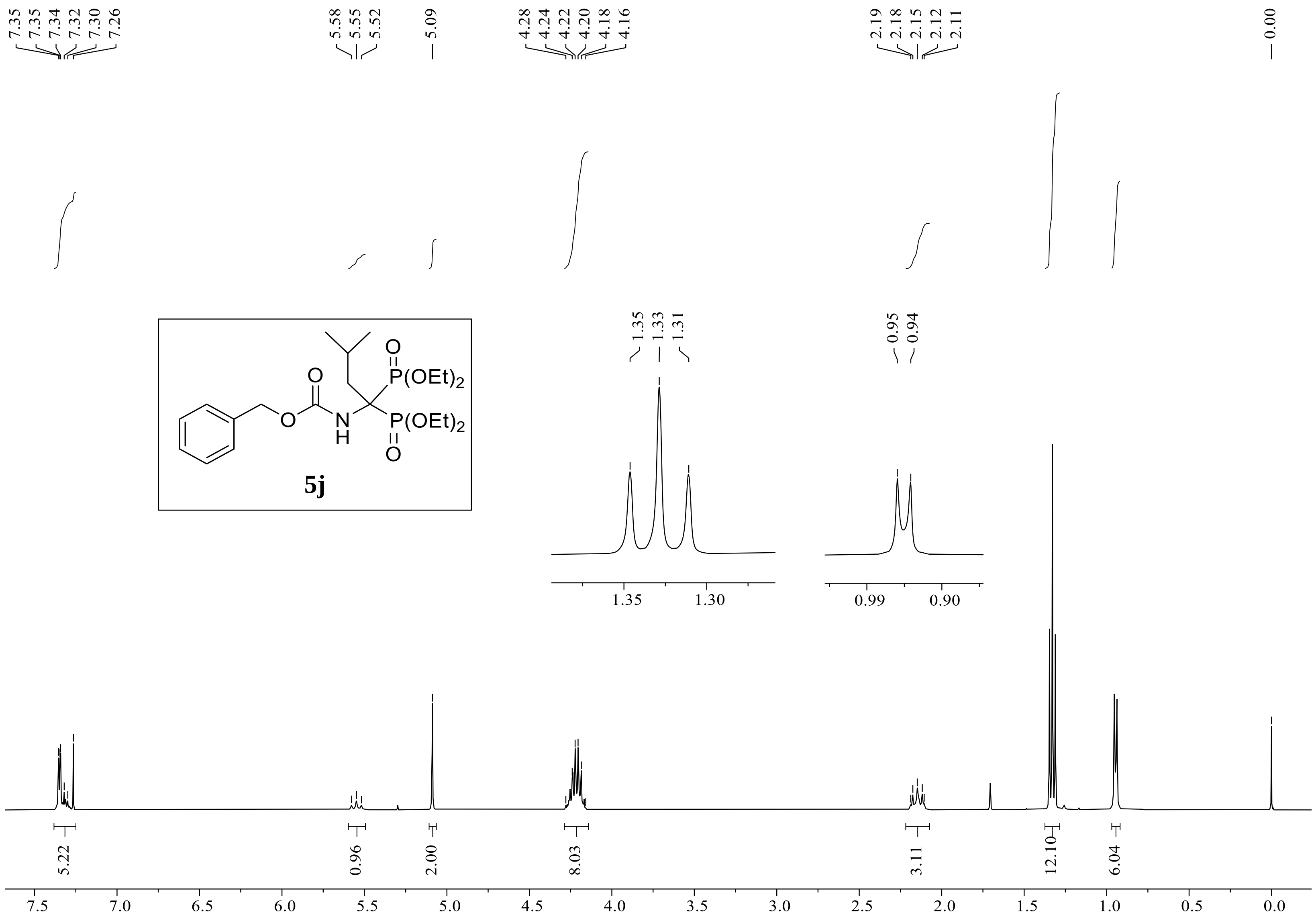

^{1}H NMR spectrum of *tetraethyl 1-(N-benzyloxycarbonylamino)-3-methylbutylene-1,1-bisphosphonate* (**5j**); 400 MHz/$CDCl_3$/TMS; δ (ppm).

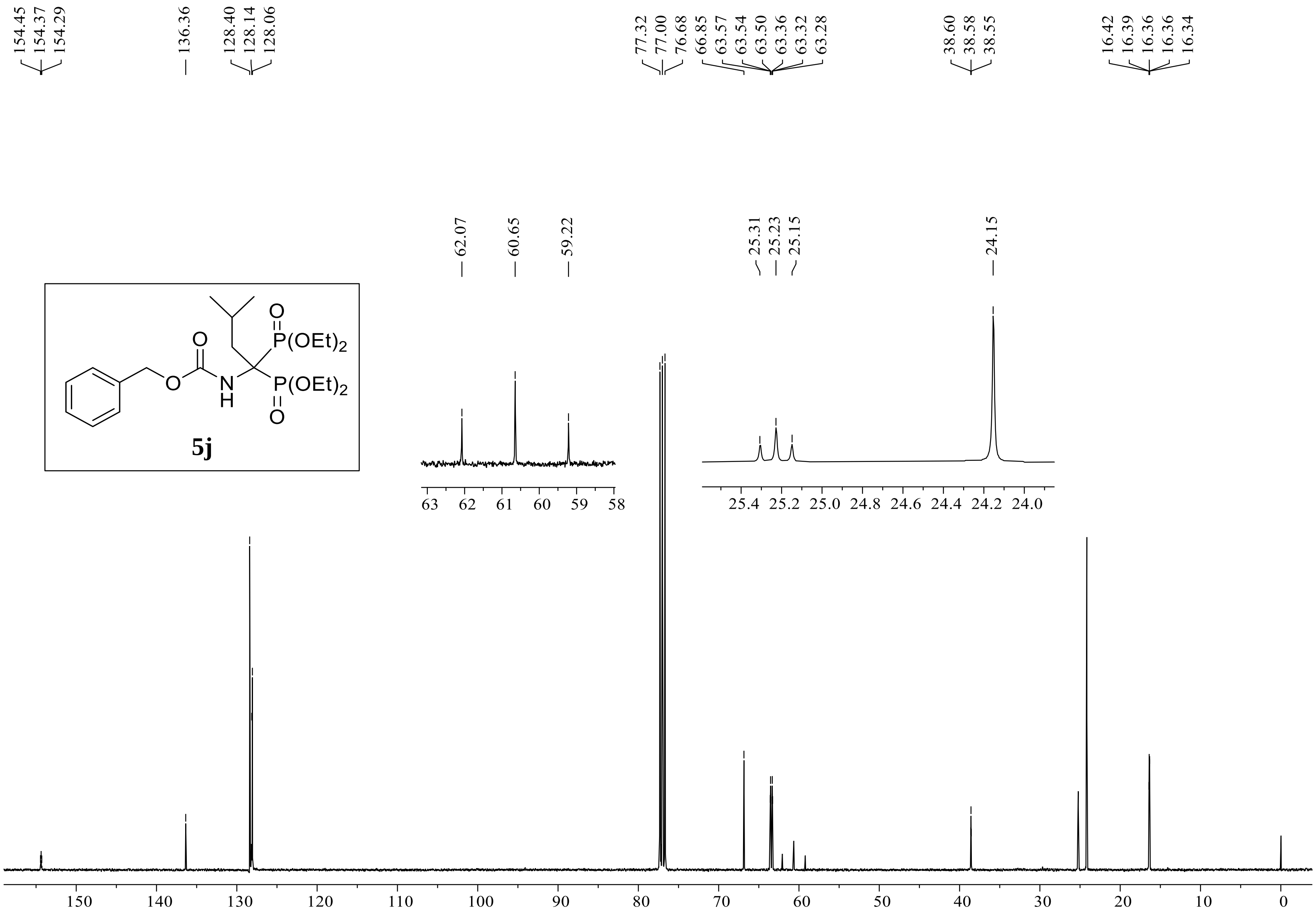

^{13}C NMR spectrum of *tetraethyl 1-(N-benzyloxycarbonylamino)-3-methylbutylene-1,1-bisphosphonate* (**5j**); 100 MHz/$CDCl_3$/TMS; δ (ppm).

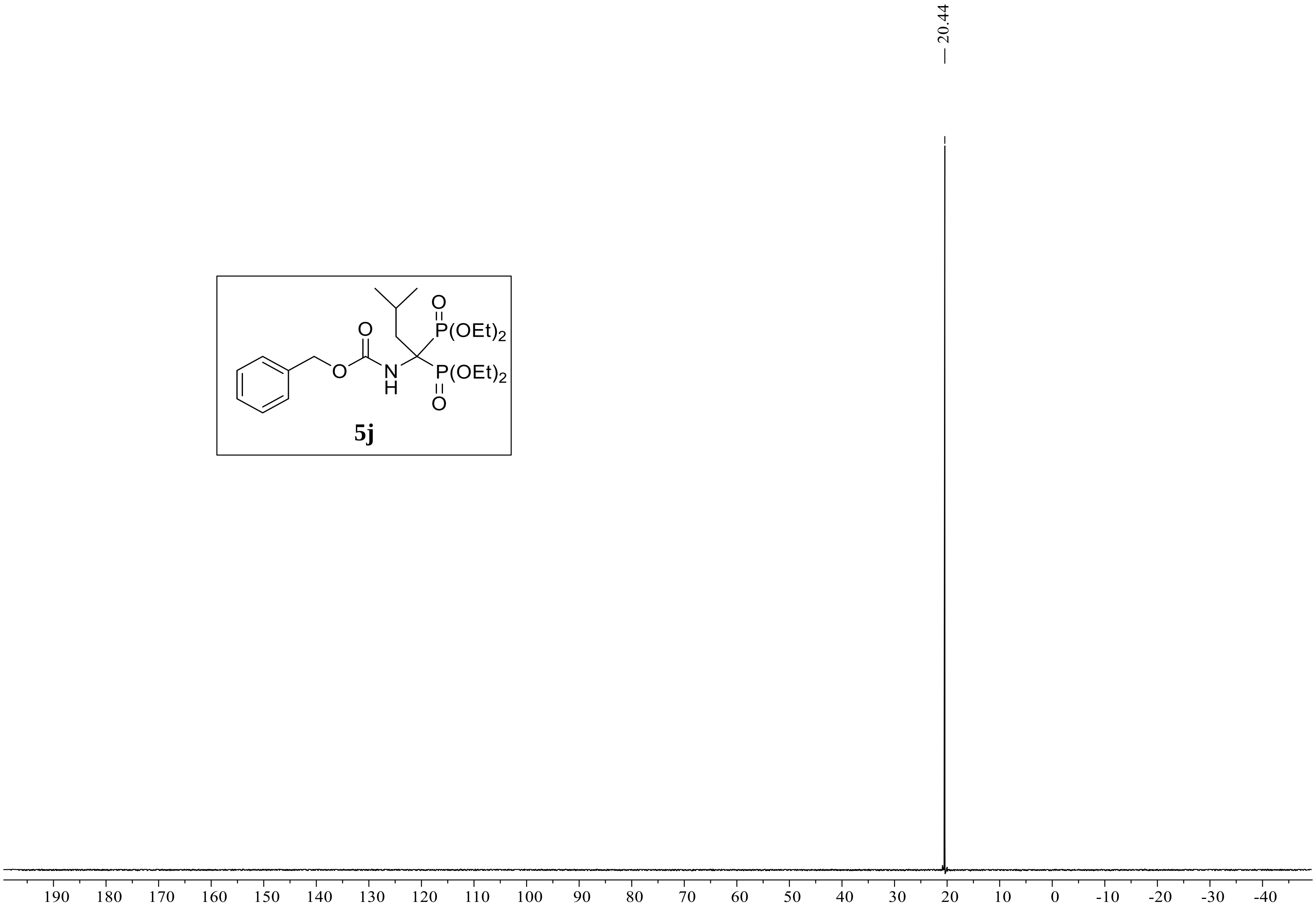

^{31}P NMR spectrum of *tetraethyl 1-(N-benzyloxycarbonylamino)-3-methylbutylene-1,1-bisphosphonate* (**5j**); 162 MHz/$CDCl_3$/TMS; δ (ppm).

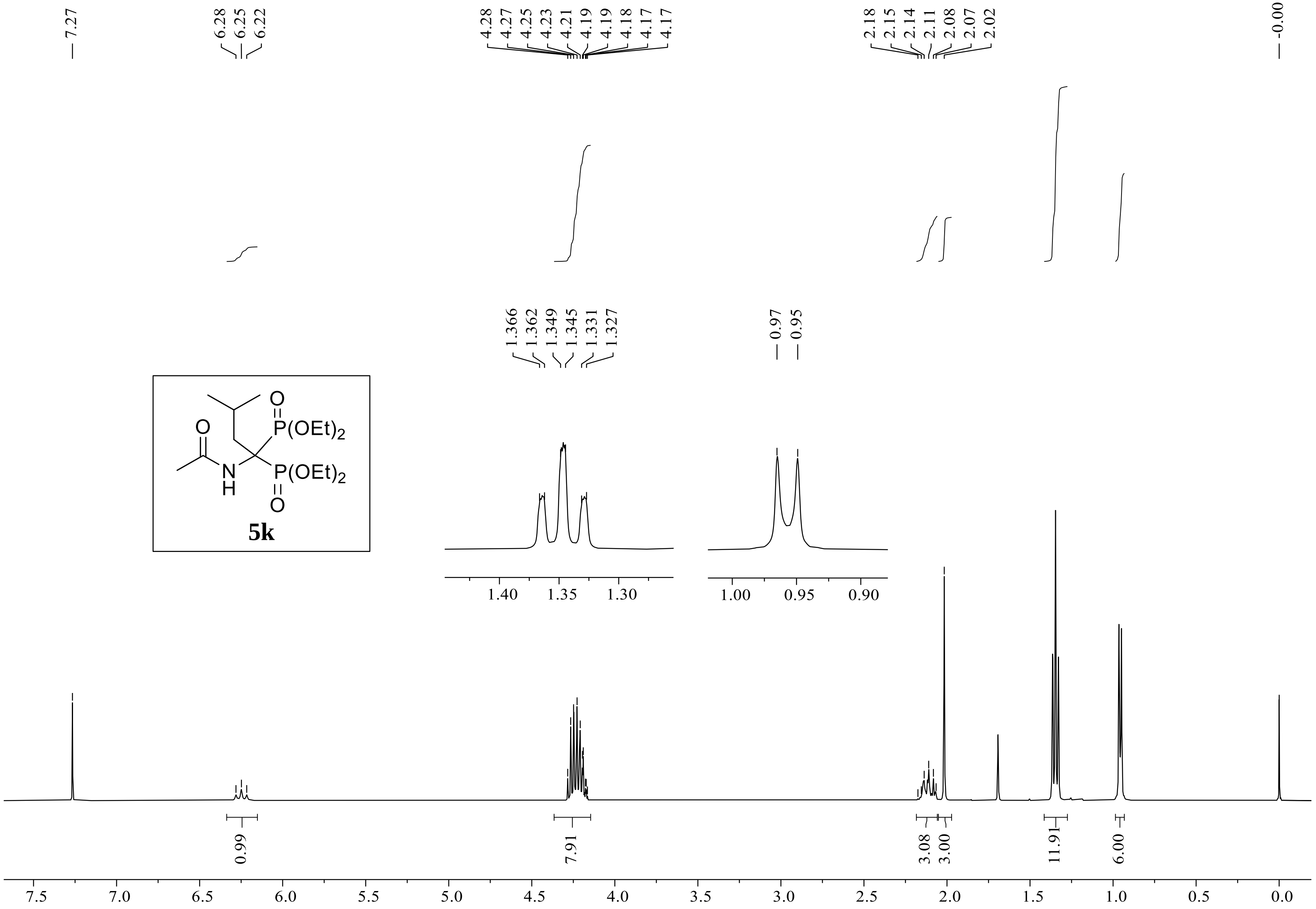

^{1}H NMR spectrum of *tetraethyl 1-(N-acetylamino)-3-methylbutylene-1,1-bisphosphonate* (**5k**); 400 MHz/$CDCl_3$/TMS; δ (ppm).

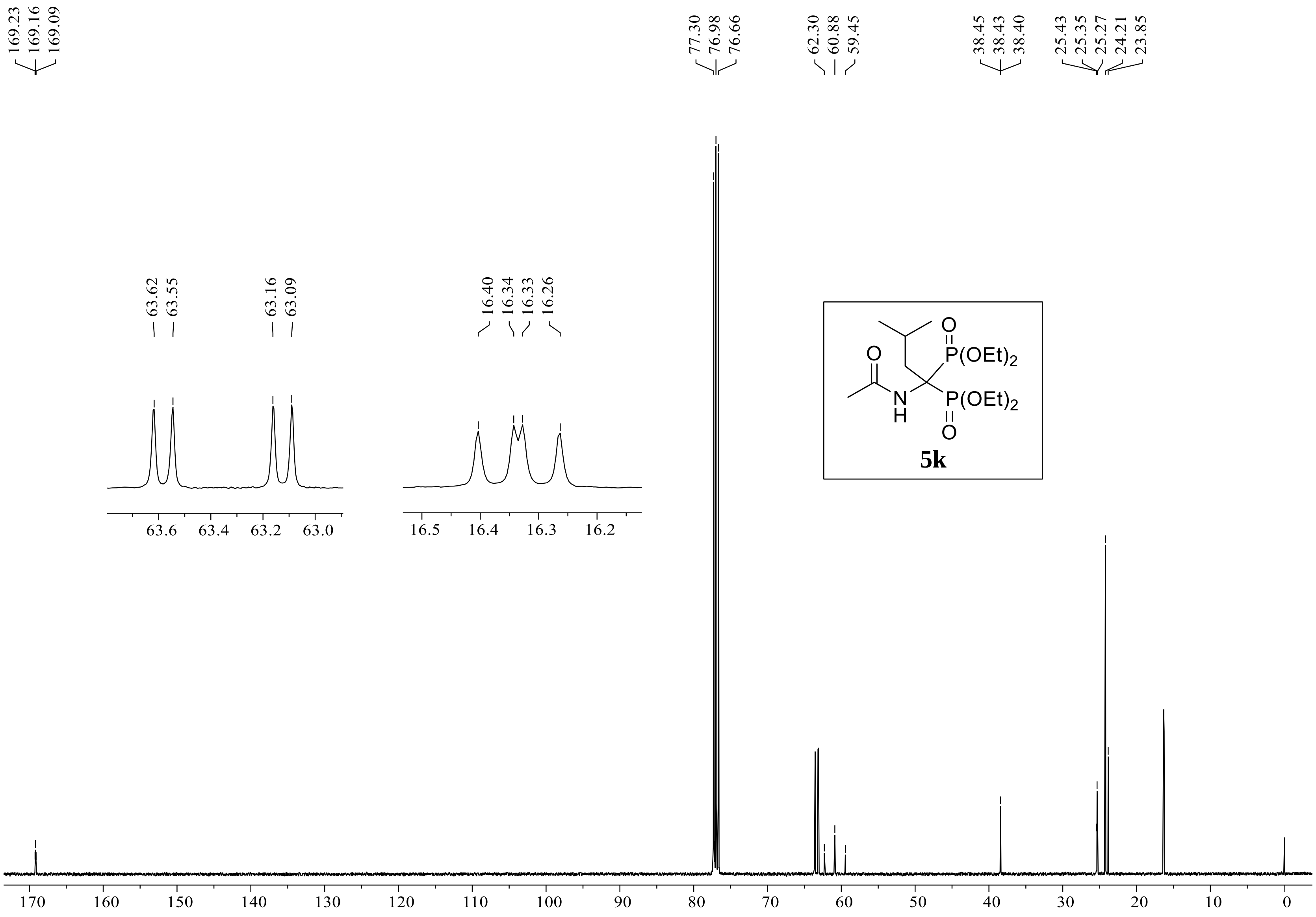

^{13}C NMR spectrum of *tetraethyl 1-(N-acetylamino)-3-methylbutylene-1,1-bisphosphonate* (**5k**); 100 MHz/$CDCl_3$/TMS; δ (ppm).

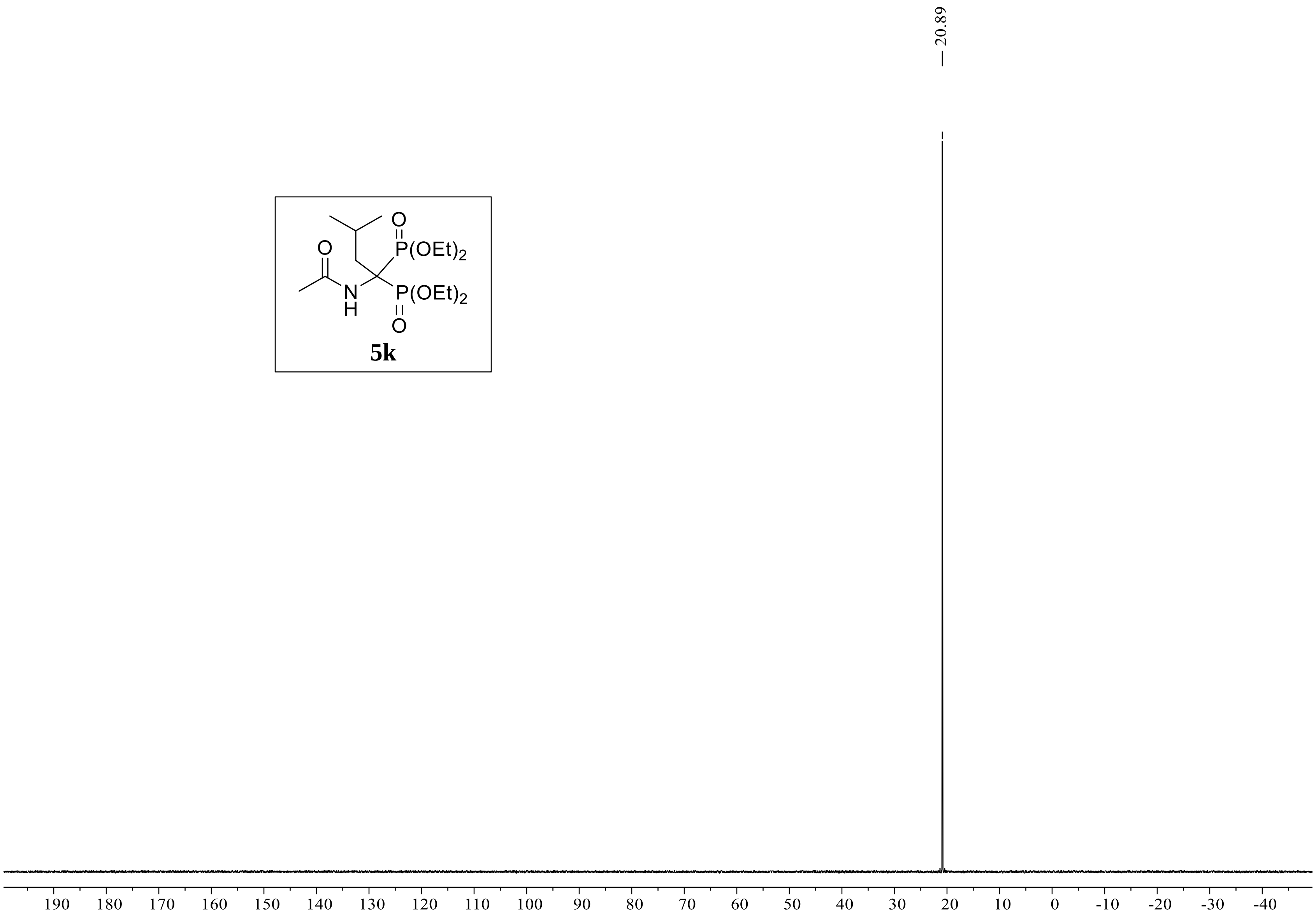

5k

^{31}P NMR spectrum of *tetraethyl 1-(N-acetylamino)-3-methylbutylene-1,1-bisphosphonate* (**5k**); 162 MHz/$CDCl_3$/TMS; δ (ppm).

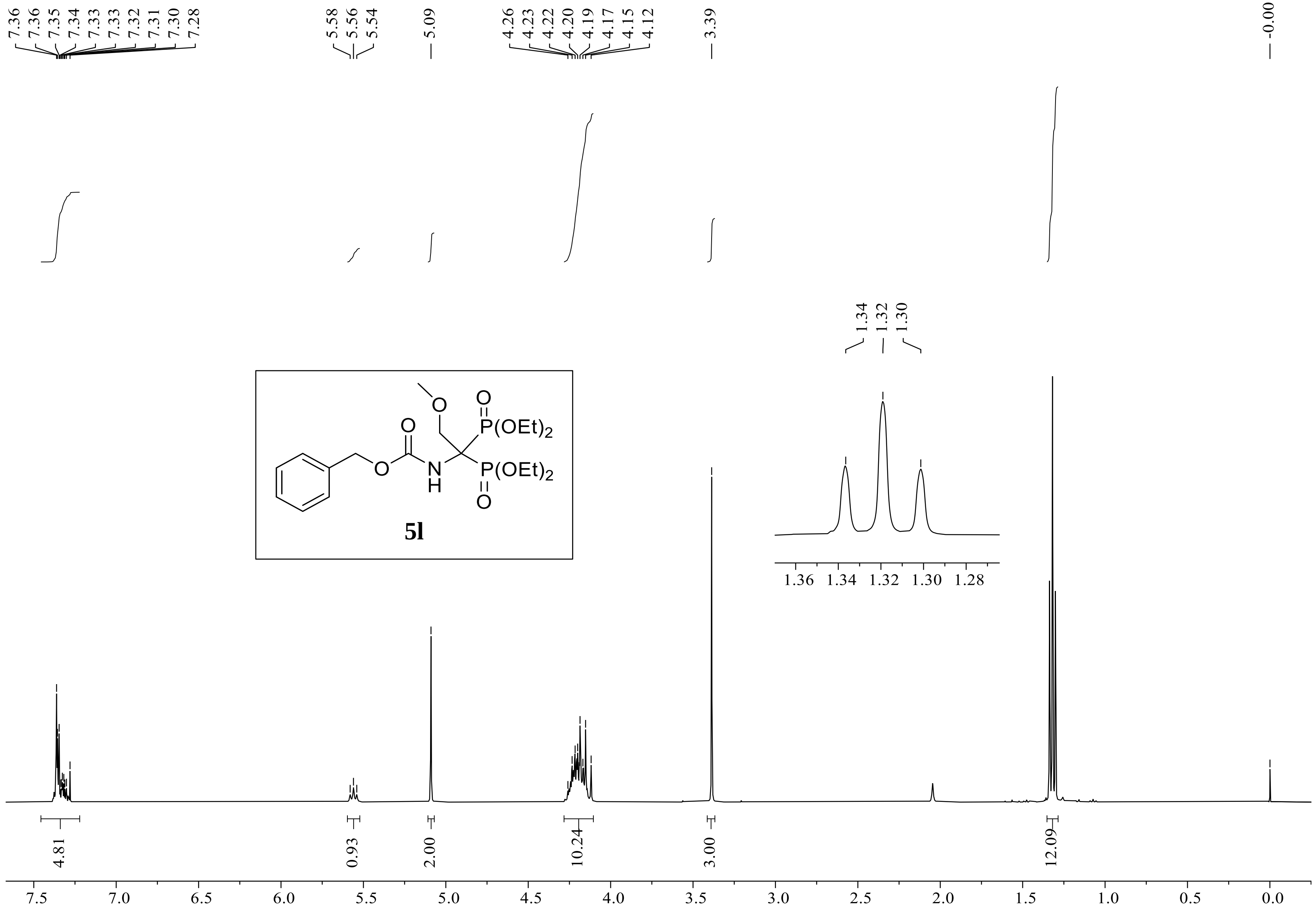

^{1}H NMR spectrum of *tetraethyl 1-(N-benzyloxycarbonylamino)-2-methoxyethylene-1,1-bisphosphonate* (**5l**); 400 MHz/$CDCl_3$/TMS; δ (ppm).

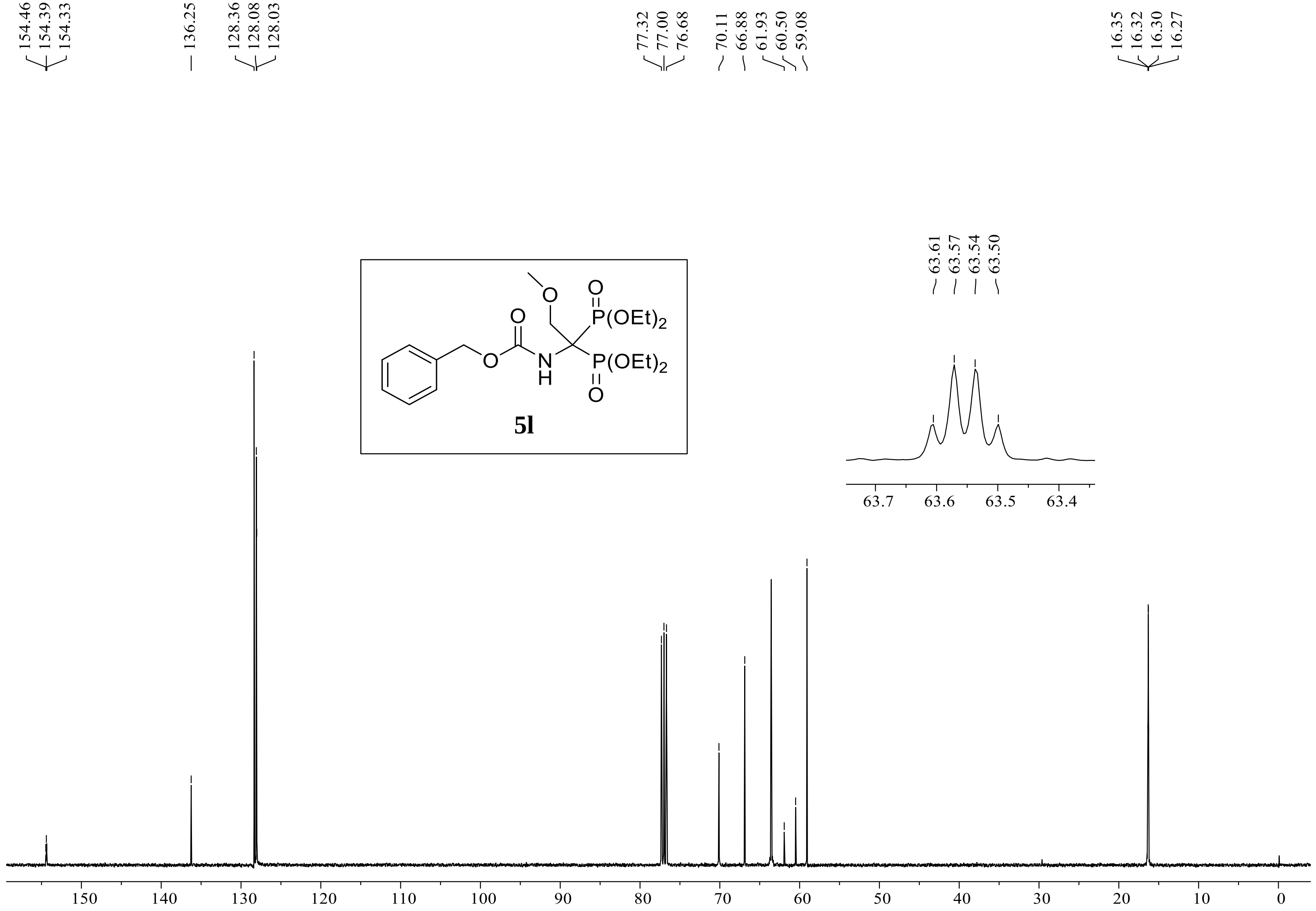

^{13}C NMR spectrum of *tetraethyl 1-(N-benzyloxycarbonylamino)-2-methoxyethylene-1,1-bisphosphonate* (**5l**); 100 MHz/$CDCl_3$/TMS; δ (ppm).

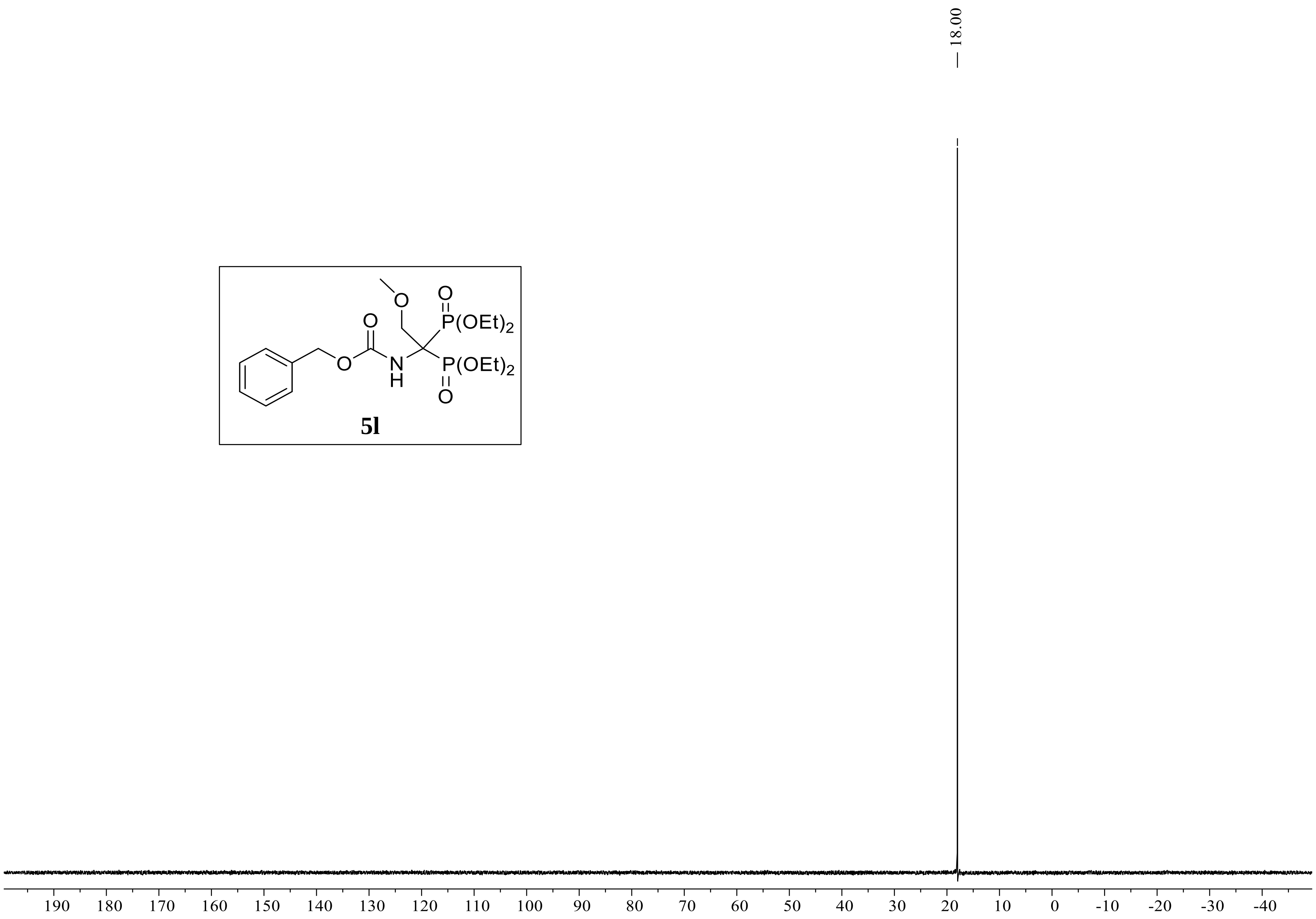

^{31}P NMR spectrum of *tetraethyl 1-(N-benzyloxycarbonylamino)-2-methoxyethylene-1,1-bisphosphonate* (**5l**); 162 MHz/$CDCl_3$/TMS; δ (ppm).

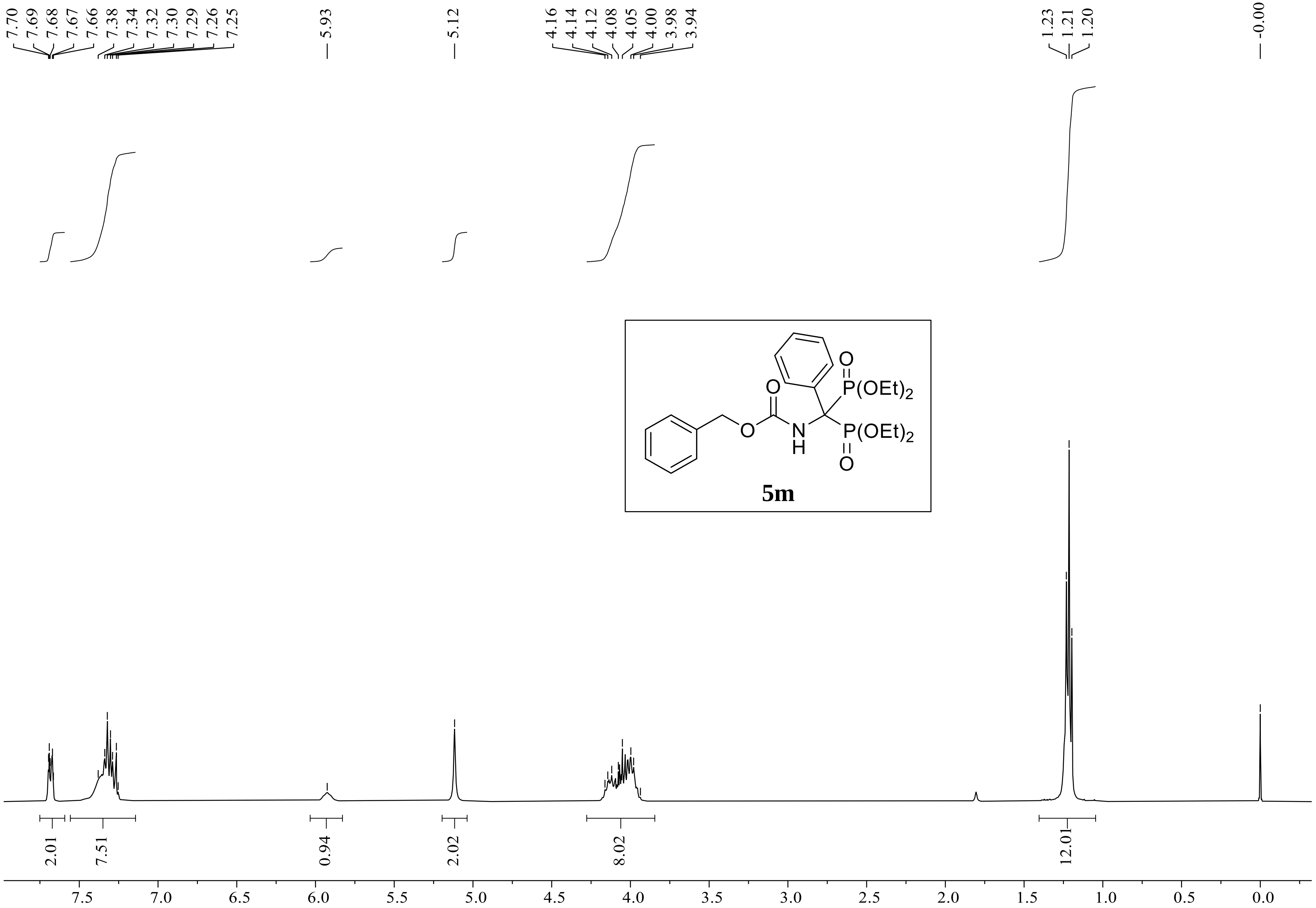

^{1}H NMR spectrum of *tetraethyl 1-(N-benzyloxycarbonylamino)phenylmethylene-1,1-bisphosphonate* (**5m**); 400 MHz/$CDCl_3$/TMS; δ (ppm).

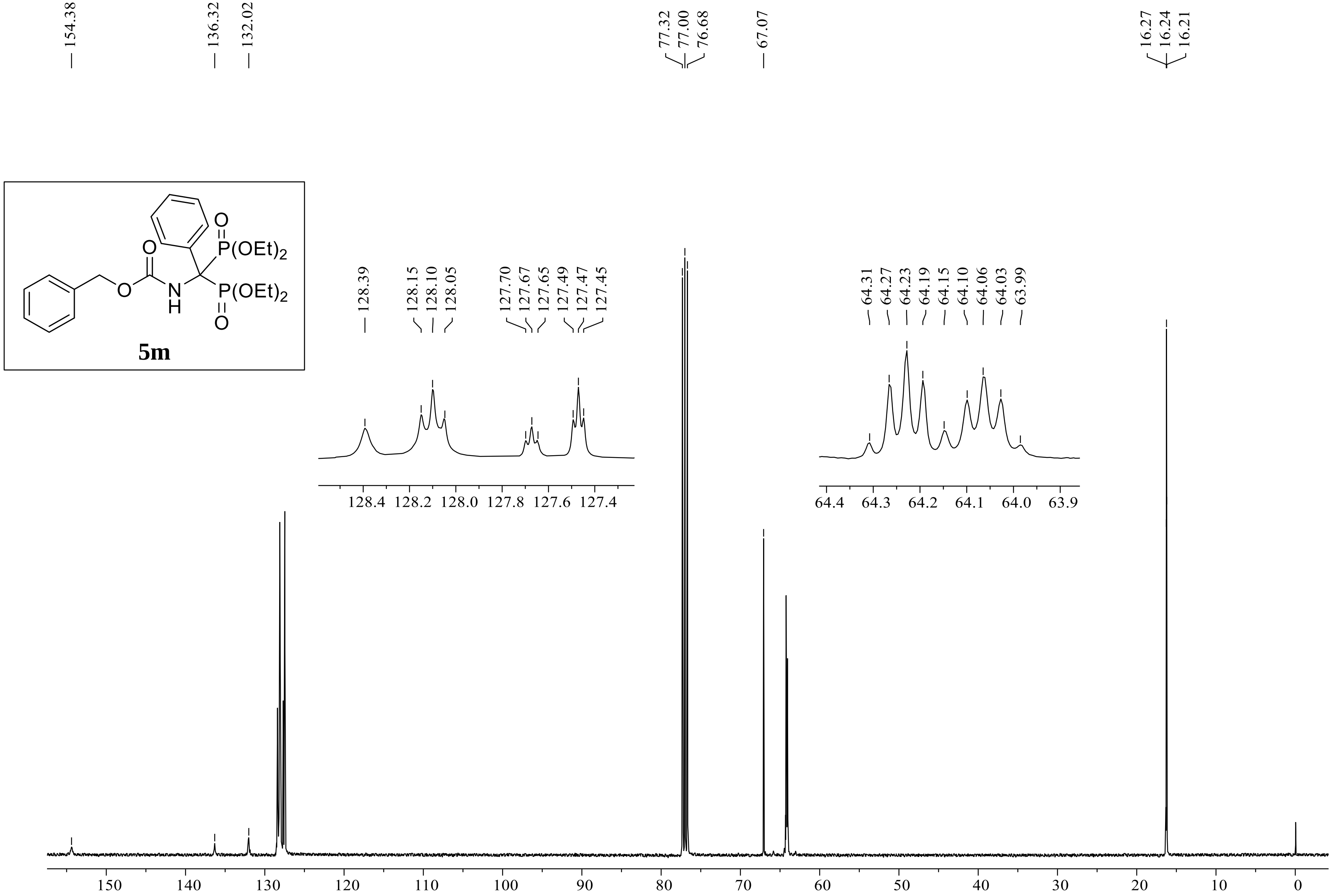

^{13}C NMR spectrum of *tetraethyl 1-(N-benzyloxycarbonylamino)phenylmethylene-1,1-bisphosphonate* (**5m**); 100 MHz/$CDCl_3$/TMS; δ (ppm).

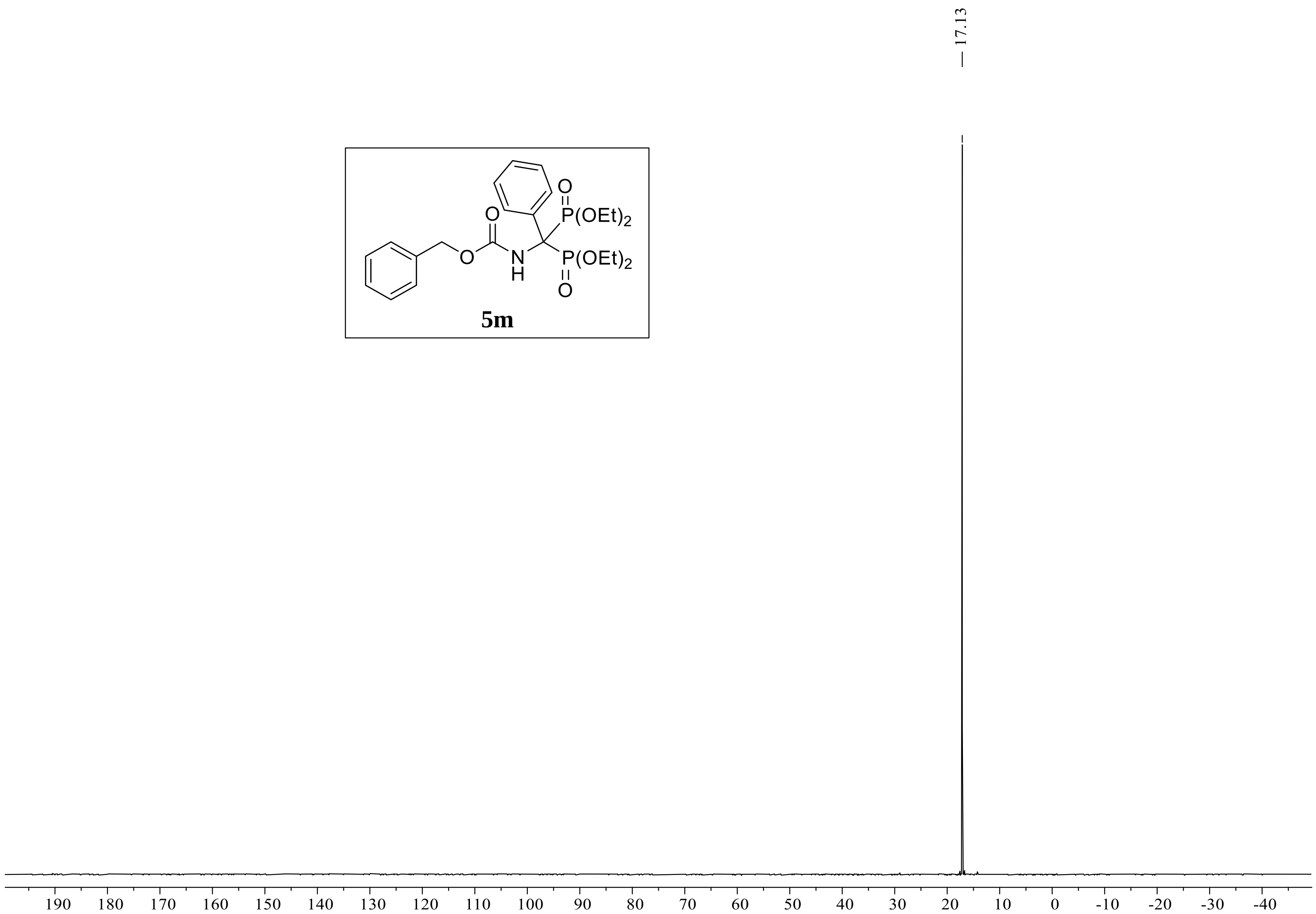

^{31}P NMR spectrum of *tetraethyl 1-(N-benzyloxycarbonylamino)phenylmethylene-1,1-bisphosphonate* (**5m**); 162 MHz/$CDCl_3$/TMS; δ (ppm).

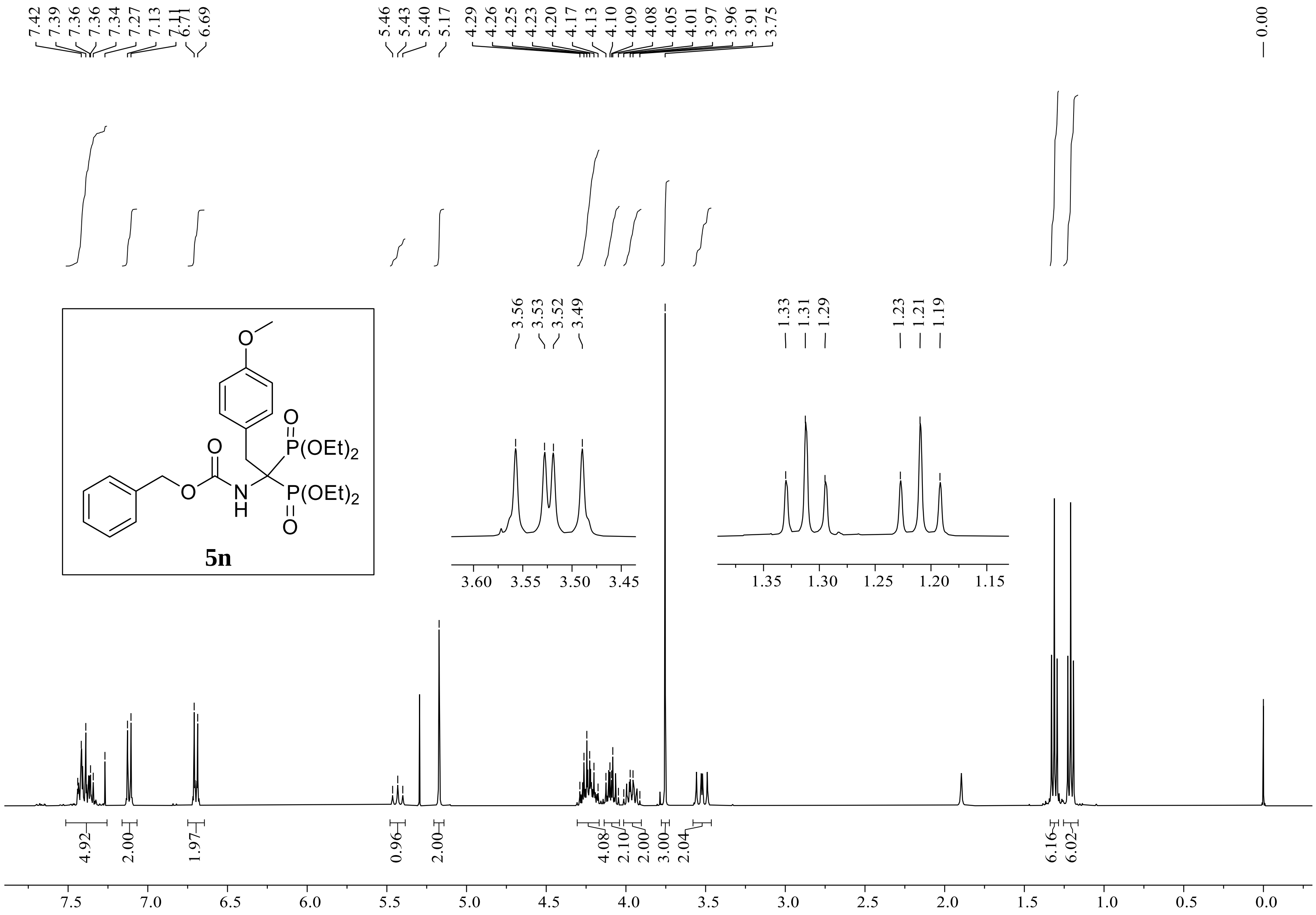

^{1}H NMR spectrum of *tetraethyl 1-(N-benzyloxycarbonylamino)-2-(4-methoxyphenyl)ethylene-1,1-bisphosphonate* (**5n**); 400 MHz/$CDCl_3$/TMS; δ (ppm).

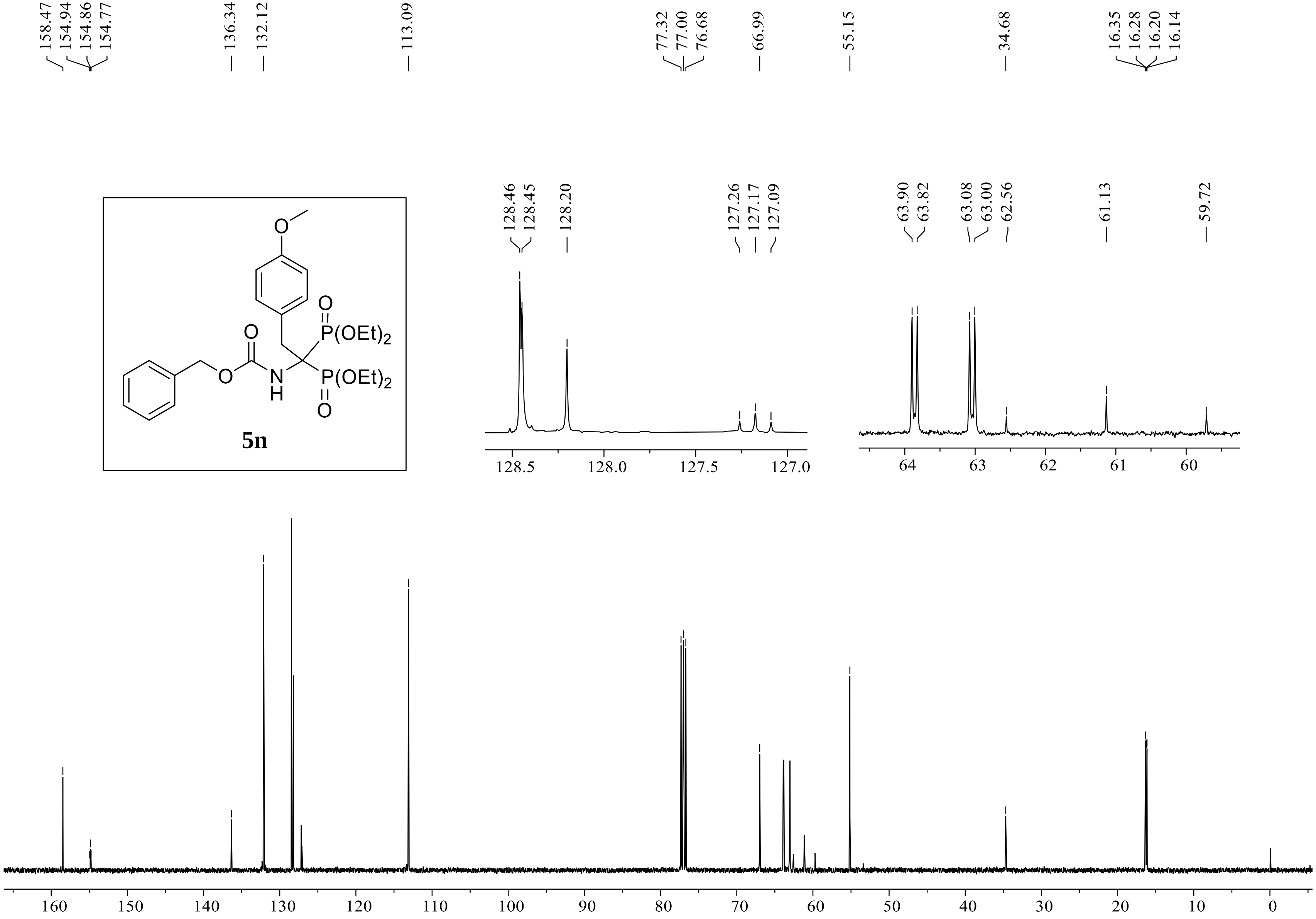

^{13}C NMR spectrum of *tetraethyl 1-(N-benzyloxycarbonylamino)-2-(4-methoxyphenyl)ethylene-1,1-bisphosphonate* (**5n**); 100 MHz/$CDCl_3$/TMS; *δ* (ppm).

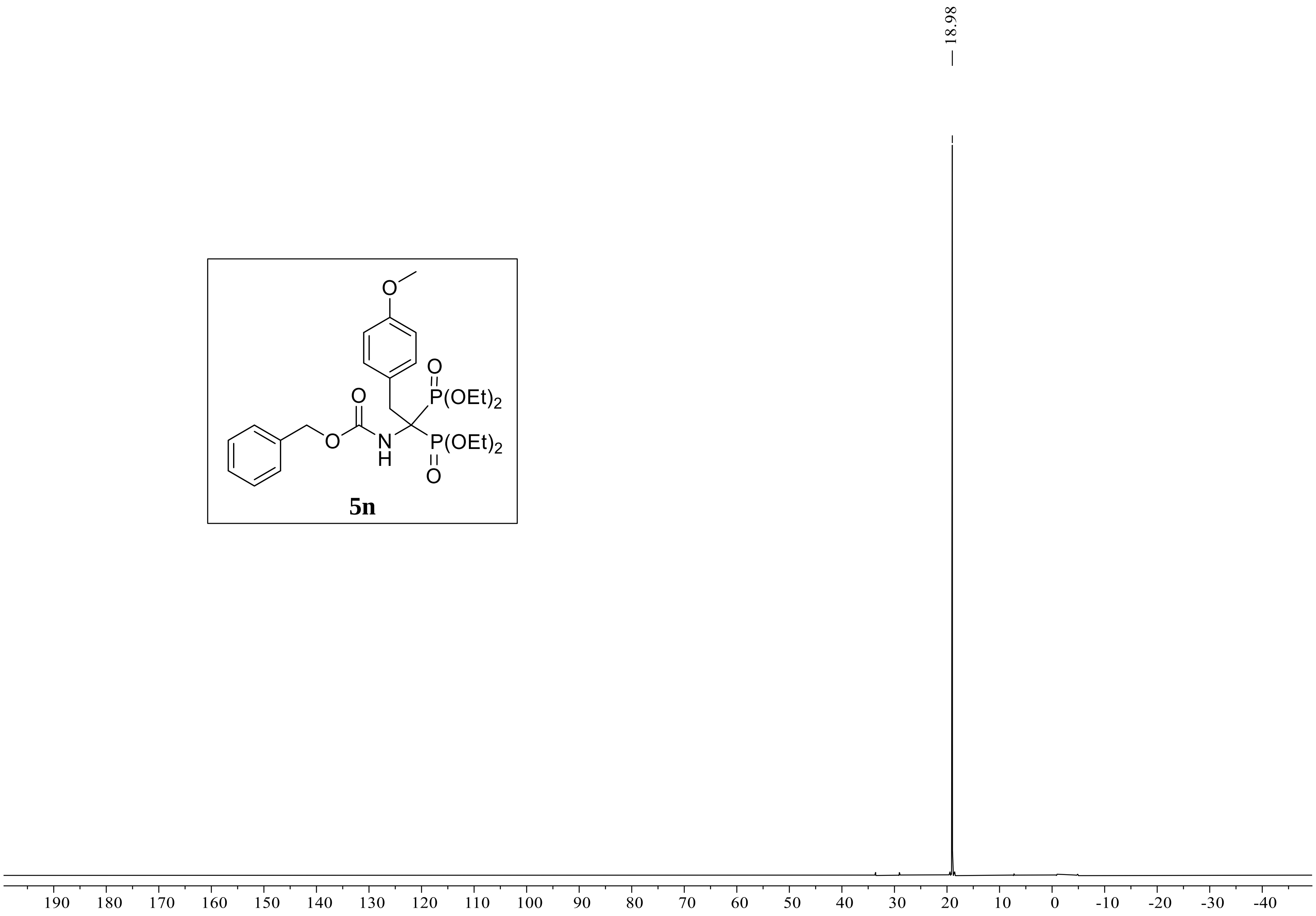

^{31}P NMR spectrum of *tetraethyl 1-(N-benzyloxycarbonylamino)-2-(4-methoxyphenyl)ethylene-1,1-bisphosphonate* (**5n**); 162 MHz/$CDCl_3$/TMS; δ (ppm).

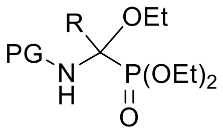

3

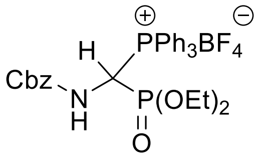

4c

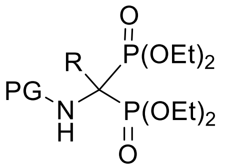

5

Table S1. Summary of characteristic ^{13}C NMR data for all 1-(*N*-acylamino)-1-ethoxyalkylphosphonates **3**.

^{13}C NMR [100 MHz, $CDCl_3$/TMS, δ (ppm) (multiplicity, *J* (Hz))]								
**Comp.**	**PG**		**R**		**Cα**	**OEt**	**P(O)OEt**
**3a**	Cbz	154.6 (d, *J* = 16.4 Hz), 136.2, 128.5, 128.2, 128.1, 66.7	Me	18.9	84.4 (d, *J* = 196.8 Hz)	58.5 (d, *J* = 8.0 Hz), 15.4	63.8 (d, *J* = 6.9 Hz), 63.4 (d, *J* = 6.9 Hz), 16.4 (d, *J* = 5.3 Hz)
**3b**	Piv	178.8 (d, *J* = 9.9 Hz), 39.9, 27.5	Me	18.7	84.9 (d, *J* = 194.5 Hz)	58.6 (d, *J* = 9.5 Hz), 15.5	63.9 (d, *J* = 6.9 Hz), 63.1 (d, *J* = 6.9 Hz), 16.5 (d, *J* = 5.3 Hz), 16.4 (d, *J* = 5.4 Hz)
**3c**	Cbz	156.0 (d, *J* = 12.2 Hz), 135.9, 128.6, 128.3, 128.1, 67.4	H	-	77.4 (d, *J* = 201.1 Hz)	65.4 (d, *J* = 12.9 Hz), 14.9	63.7 (d, *J* = 6.5 Hz), 63.2 (d, *J* = 6.9 Hz), 16.38 (d, *J* = 5.3 Hz) and 16.36 (d, *J* = 5.3 Hz)[b]
**3d**	Cbz	154.6 (d, *J* = 16.4 Hz), 136.1, 128.5, 128.24, 128.19, 66.8	CH_2Ph	135.6 (d, *J* = 3.6 Hz), 131.2, 127.6, 126.5, 39.1 (d, *J* = 2.9 Hz)	87.1 (d, *J* = 186.5 Hz)	59.4 (d, *J* = 4.6 Hz), 15.2	63.2 (d, *J* = 7.2 Hz), 62.9 (d, *J* = 7.2 Hz), 16.3 (d, *J* = 5.8 Hz), 16.0 (d, *J* = 6.1 Hz)
**3e**	Ac	170.3 (d, *J* = 9.2 Hz), 24.5	CH_2Ph	135.6 (d, *J* = 4.0 Hz), 131.1, 127.7, 126.6, 38.9	87.6 (d, *J* = 185.9 Hz)	59.9 (d, *J* = 5.3 Hz), 15.2	63.4 (d, *J* = 7.2 Hz), 62.8 (d, *J* = 7.2 Hz), 16.4 (d, *J* = 6.1 Hz), 16.1 (d, *J* = 6.1 Hz)
**3f**	Cbz	154.4 (d, *J* = 16.2), 136.2, 128.5, 128.3, 128.1, 66.7	Et	25.2, 8.5 (d, *J* = 2.1 Hz)	87.7 (d, *J* = 189.5 Hz)	58.4 (d, *J* = 7.2 Hz), 15.3	63.6 (d, *J* = 7.2 Hz), 63.1 (d, *J* = 7.1 Hz), 16.4 (d, *J* = 5.7 Hz)
**3g**	Cbz	154.4 (d, *J* = 16.1 Hz), 136.2, 128.5, 128.1, 128.0, 66.6	Pr	34.4, 17.2 (d, *J* = 2.0 Hz), 14.4	87.3 (d, *J* = 189.7 Hz)	58.4 (d, *J* = 7.4 Hz), 15.3	63.6 (d, *J* = 7.1 Hz), 63.1 (d, *J* = 7.0 Hz), 16.4 (d, *J* = 5.5 Hz)
**3h**	Cbz	154.6 (d, *J* = 18.2 Hz), 136.3, 128.5, 128.2, 128.1, 66.7	*i*-Pr	31.4, 17.7 (d, *J* = 3.1 Hz), 17.5	90.2 (d, *J* = 185.8 Hz)	58.6 (d, *J* = 6.5 Hz), 15.3	63.4 (d, *J* = 7.2 Hz), 62.9 (d, *J* = 7.6 Hz), 16.4 (d, *J* = 5.3 Hz)

Table S1. Continued.

^{13}C NMR [100 MHz, $CDCl_3$/TMS, δ (ppm) (multiplicity, *J* (Hz))]							
**Comp.**	**PG**		**R**		**C_α**	**OEt**	**P(O)OEt**
**3i**	Cbz	154.3 (d, *J* = 16.1 Hz), 136.2, 128.4, 128.1, 128.0, 66.6	Bu	31.9, 25.8 (d, *J* = 1.9 Hz), 22.9, 13.9	87.3 (d, *J* = 189.5 Hz)	58.3 (d, *J* = 7.2 Hz), 15.2	63.5 (d, *J* = 7.2 Hz) and 63.1 (d, *J* = 7.2 Hz)[a], 16.3 (d, *J* = 5.6 Hz)
**3j**	Cbz	154.4 (d, *J* = 16.7 Hz), 136.2, 128.5, 128.14, 128.06, 66.6	*i*-Bu	40.3, 24.5 (d, *J* = 4.6 Hz), 23.2	87.9 (d, *J* = 188.2 Hz)	58.5 (d, *J* = 7.2 Hz), 15.1	63.8 (d, *J* = 7.2 Hz), 62.9 (d, *J* = 7.2 Hz), 16.40 (d, *J* = 5.7 Hz) and 16.38 (d, *J* = 5.7 Hz)[b]
**3k**	Ac	170.1 (d, *J* = 12.9 Hz), 24.5	*i*-Bu	39.7, 24.9, 24.7 (d, *J* = 3.0 Hz), 23.1	88.8 (d, *J* = 187.3 Hz)	59.0 (d, *J* = 8.0 Hz), 15.1	64.1 (d, *J* = 7.2 Hz), 62.7 (d, *J* = 7.2 Hz), 16.43 (d, *J* = 6.1 Hz) and 16.40 (d, *J* = 5.3 Hz)[b]
**3l**	Cbz	154.4 (d, *J* = 14.3 Hz), 136.1, 128.5, 128.2, 128.1, 67.0	CH_2OMe	72.5, 59.4	86.0 (d, *J* = 188.8 Hz)	59.3 (d, *J* = 6.0 Hz), 15.4	63.6 (d, *J* = 7.2 Hz), 63.5 (d, *J* = 6.9 Hz), 16.4 (d, *J* = 5.7 Hz)
**3m**	Cbz	154.4 (d, *J* = 20.9 Hz), 136.0, 128.4, 128.14, 128.11, 67.0	Ph	127.75, 127.72, 127.41, 127.37	87.3 (d, *J* = 185.8 Hz)	59.8 (d, *J* = 6.1 Hz), 15.3	64.5 (d, *J* = 7.2 Hz), 63.8 (d, *J* = 7.7 Hz), 16.3 (d, *J* = 5.7 Hz), 16.2 (d, *J* = 5.6 Hz)
**^{13}C NMR [100 MHz, CD_3CN/TMS, δ (ppm) (multiplicity, *J* (Hz))]**							
**3n**	Cbz	155.4 (d, *J* = 14.1 Hz), 138.0, 129.5, 129.1, 129.0, 67.2	1,4-CH_2-C_6H_4OMe	159.5, 133.1, 128.5 (d, *J* = 3.8 Hz), 114.0, 55.8, 39.1 (d, *J* = 5.0 Hz)	88.3 (d, *J* = 187.3 Hz),	60.1 (d, *J* = 4.2 Hz), 14.6	63.78 (d, *J* = 7.2 Hz) and 62.73 (d, *J* = 7.2 Hz)[a], 15.7 (d, *J* = 5.7 Hz) and 15.6 (d, *J* = 5.7 Hz)[b]

[a] Overlapping signals of P(O)(O**CH_2**CH_3)$_2$ groups. [b] Overlapping signals of P(O)(OCH_2**CH_3**)$_2$ groups.

Table S2. ^{13}C NMR data of diethyl 1-(*N*-benzyloxycarbonylamino)-1-triphenylphosphoniummethylphosphonate **4c**.

^{13}C NMR [100 MHz, $CDCl_3$/TMS, δ (ppm) (multiplicity, *J* (Hz))]			
**PG**	**C_α**	**$^+PPh_3$**	**P(O)OEt**
156.3, 135.7, 128.4, 128.1, 128.0, 67.9	48.1 (dd, J = 152.8, 48.5 Hz)	135.1 (d, J = 3.1 Hz), 134.8 (d, J = 10.3 Hz), 130.1 (d, J = 13.0 Hz), 116.9 (d, J = 84.7 Hz)	65.1 (d, J = 7.6 Hz), 64.9 (d, J = 6.9 Hz), 16.1 (d, J = 6.1 Hz), 16.0 (d, J = 5.0 Hz)

Table S3. Summary of characteristic ^{13}C NMR data for all 1-(*N*-acylamino)alkylene-1,1-bisphosphonates **5**.

^{13}C NMR [100 MHz, $CDCl_3$/TMS, δ (ppm) (multiplicity, *J* (Hz))]						
**Comp.**	**PG**		**R**		**C$_α$**	**P(O)(OEt)$_2$**
**5a**	Cbz	154.3, 136.3, 128.4, 128.11, 128.09, 66.7	Me	16.2 (br t, *J* = 4.1 Hz)	55.8 (t, *J* = 146.9 Hz)	63.83 (d, *J* = 3.4 Hz) and 63.80 (d, *J* = 3.4 Hz) and 63.75 (d, *J* = 3.4 Hz) and 63.72 (d, *J* = 3.4 Hz)[a], 16.5-16.3 (m)[b]
**5b**	Piv	177.7 (t, *J* = 5.1 Hz), 39.8, 27.4	Me	16.7 (t, *J* = 4.5 Hz)	56.7 (t, *J* = 144.9 Hz)	63.76 (d, *J* = 3.4 Hz) and 63.73 (d, *J* = 3.4 Hz) and 63.67 (d, *J* = 3.4 Hz) and 63.64 (d, *J* = 3.4 Hz)[a], 16.5-16.4 (m)[b]
**5c**	Cbz	155.5 (t, *J* = 4.9 Hz), 135.9, 128.5, 128.3, 128.1, 67.6	H	-	46.0 (t, *J* = 146.8 Hz)	63.5, 16.3-16.2 (m)[b]
**5d**	Cbz	154.9 (t, *J* = 8.8 Hz), 136.4, 128.49, 128.45, 128.2, 67.1	CH_2Ph	135.3 (t, *J* = 8.6 Hz), 131.2, 127.7, 126.7, 35.5	61.2 (t, *J* = 143.1 Hz)	63.9 (d, *J* = 7.5 Hz), 63.0 (d, *J* = 7.4 Hz), 16.3 (d, *J* = 6.3 Hz), 16.2 (d, *J* = 6.2 Hz)
**5e**	Ac	169.9 (t, *J* = 7.3 Hz), 23.9	CH_2Ph	135.5 (t, *J* = 8.2 Hz), 131.1, 127.7, 126.9, 35.2	61.4 (t, *J* = 143.1 Hz)	64.1 (d, *J* = 7.3 Hz), 62.9 (d, *J* = 7.6 Hz), 16.4 (d, *J* = 6.2 Hz), 16.1 (d, *J* = 6.5 Hz)
**5f**	Cbz	154.3 (t, *J* = 8.0 Hz), 136.4, 128.4, 128.1, 66.8	Et	23.8 (t, *J* = 3.0 Hz), 9.1 (t, *J* = 6.5 Hz)	60.4 (t, *J* = 144.4 Hz)	63.67 (d, *J* = 3.5 Hz) and 63.64 (d, *J* = 3.5 Hz)[a], 63.42 (d, *J* = 3.5 Hz) and 63.38 (d, *J* = 3.5 Hz)[a], 16.5-16.3 (m)[b]
**5g**	Cbz	154.3 (t, *J* = 7.6 Hz), 136.4, 128.4, 128.1, 66.8	Pr	32.7 (t, *J* = 3.0 Hz), 17.7 (t, *J* = 6.2 Hz), 14.5	60.1 (t, *J* = 143.5 Hz)	63.66 (d, *J* = 3.5 Hz) and 63.63 (d, *J* = 3.5 Hz)[a], 63.43 (d, *J* = 3.5 Hz) and 63.40 (d, *J* = 3.5 Hz)[a], 16.45 (d, *J* = 2.9 Hz) and 16.43 (d, *J* = 2.6 Hz) and 16.40 (d, *J* = 2.7 Hz) and 16.37 (d, *J* = 2.9 Hz)[b]
**5h**	Cbz	154.3 (t, *J* = 8.2 Hz), 136.4, 128.4, 128.0, 66.8	*i*-Pr	30.7, 18.8 (t, *J* = 4.3 Hz)	64.6 (t, *J* = 139.2 Hz)	63.40 (d, *J* = 3.6 Hz) and 63.36 (d, *J* = 3.6 Hz)[a], 63.11 (d, *J* = 3.5 Hz) and 63.08 (d, *J* = 3.6 Hz)[a], 16.40 (d, *J* = 3.0 Hz) and 16.37 (d, *J* = 2.9 Hz) and 16.34 (d, *J* = 2.9 Hz) and 16.31 (d, *J* = 3.0 Hz)[b]
**5i**	Cbz	154.3 (t, *J* = 7.3 Hz), 136.4, 128.4, 128.0, 66.7	Bu	30.4 (t, *J* = 3.0 Hz), 26.2 (t, *J* = 6.0 Hz), 23.0, 13.9	60.0 (t, *J* = 144.4 Hz)	63.59 (d, *J* = 3.5 Hz) and 63.55 (d, *J* = 3.5 Hz)[a], 63.38 (d, *J* = 3.4 Hz) and 63.34 (d, *J* = 3.5 Hz)[a], 16.39 (d, *J* = 2.7 Hz) and 16.36 (d, *J* = 2.6 Hz) and 16.33 (d, *J* = 2.6 Hz) and 16.31 (d, *J* = 2.7 Hz)[b]
**5j**	Cbz	154.4 (t, *J* = 8.0 Hz), 136.4, 128.4, 128.14, 128.06, 66.9	*i*-Bu	38.6 (t, *J* = 2.2 Hz), 25.2 (t, *J* = 7.8 Hz), 24.2	60.7 (t, *J* = 142.9 Hz)	63.56 (d, *J* = 3.5 Hz) and 63.52 (d, *J* = 3.5 Hz)[a], 63.34 (d, *J* = 3.5 Hz) and 63.30 (d, *J* = 3.5 Hz)[a], 16.44-16.26 (m)[b].

**5k**	Ac	169.2 (t, *J* = 6.9 Hz), 23.9	*i*-Bu	38.4 (t, *J* = 2.6 Hz), 25.4 (t, *J* = 8.0 Hz), 24.2	60.9 (t, *J* = 143.2 Hz)	63.6 (d, *J* = 7.3 Hz), 63.1 (d, *J* = 7.2 Hz), 16.4 (d, *J* = 6.0 Hz) and 16.3 (d, *J* = 6.3 Hz)[b]
**5l**	Cbz	δ 154.4 (t, *J* = 6.2 Hz), 136.3, 128.4, 128.1, 128.0, 66.9	CH_2OMe	70.1, 59.1	60.5 (t, *J* = 142.7 Hz)	63.59 (d, *J* = 3.5 Hz) and 63.55 (d, *J* = 3.6 Hz) and 63.52 (d, *J* = 3.6 Hz)[a], 16.4-16.3 (m)[b]
**5m**	Cbz	154.4 (br s), 136.3, 128.4, 67.1[c]	Ph	132.0, 128.1 (t, *J* = 5.0 Hz), 127.6 (t, *J* = 2.6 Hz), 127.5 (t, *J* = 2.3 Hz)	64.15 (t, *J* = 140.4 Hz)	64.25 (d, *J* = 3.7 Hz) and 64.21 (d, *J* = 3.6 Hz) and 64.08 (d, *J* = 3.6 Hz) and 64.05 (d, *J* = 3.8 Hz)[a], 16.26 (d, *J* = 3.1 Hz) and 16.23 (d, *J* = 3.0 Hz)[b].
**5n**	Cbz	154.9 (t, *J* = 8.8 Hz), 136.3, 128.46, 128.45, 128.2, 67.0	1,4-CH_2-C_6H_4OMe	158.5, 132.1, 127.2 (t, *J* = 8.6 Hz), 113.1, 55.2, 34.7	61.1 (t, *J* = 143.0 Hz)	63.9 (d, *J* = 7.4 Hz), 63.0 (d, *J* = 7.6 Hz), 16.3 (d, *J* = 6.3 Hz), 16.2 (d, *J* = 6.2 Hz)

[a] Overlapping signals of P(O)(O**CH_2**CH_3)$_2$ groups. [b] Overlapping signals of P(O)(OCH_2**CH_3**)$_2$ groups. [c] The lower number of expected signals results from the probable overlap with the signals of the Ph group at the α position.
